# Perioperative mortality rates in low-income and middle-income countries: a systematic review and meta-analysis

**DOI:** 10.1136/bmjgh-2018-000810

**Published:** 2018-06-22

**Authors:** Joshua S Ng-Kamstra, Sumedha Arya, Sarah L M Greenberg, Meera Kotagal, Catherine Arsenault, David Ljungman, Rachel R Yorlets, Arnav Agarwal, Claudia Frankfurter, Anton Nikouline, Francis Yi Xing Lai, Charlotta L Palmqvist, Terence Fu, Tahrin Mahmood, Sneha Raju, Sristi Sharma, Isobel H Marks, Alexis Bowder, Lebei Pi, John G Meara, Mark G Shrime

**Affiliations:** 1 Department of Surgery, University of Toronto, Toronto, Ontario, Canada; 2 Program in Global Surgery and Social Change, Harvard Medical School, Boston, Massachusetts, USA; 3 Department of Plastic and Oral Surgery, Boston Children’s Hospital, Boston, Massachusetts, USA; 4 Department of Medicine, University of Toronto, Toronto, Ontario, Canada; 5 Division of General and Thoracic Surgery, Seattle Children’s Hospital, Seattle, Washington, USA; 6 Department of Pediatric General and Thoracic Surgery, Cincinnati Children’s Hospital Medical Center, Cincinnati, Ohio, USA; 7 Department of Global Health and Population, Harvard T.H. Chan school of Public Health, Boston, Massachusetts, USA; 8 Department of Surgery, Sahlgrenska Academy, Institute of Clinical Sciences, University of Gothenburg, Gothenburg, Sweden; 9 Faculty of Medicine, University of Toronto, Toronto, Ontario, Canada; 10 Division of Emergency Medicine, University of Toronto, Toronto, Ontario, Canada; 11 Skin and Cancer Foundation, Melbourne, Victoria, Australia; 12 Faculty of Medicine, Lund University, Lund, Sweden; 13 Department of Otolaryngology, University of Toronto, Toronto, Ontario, Canada; 14 Department of Surgery, University of Colorado, Denver, Colorado, USA; 15 Imperial College London, London, UK; 16 Department of Surgery, Medical College of Wisconsin, Milwaukee, Wisconsin, USA; 17 Department of Medicine, University of Ottawa, Ottawa, Ontario, Canada; 18 Department of Otolaryngology, Massachusetts Eye and Ear Infirmary, Boston, Massachusetts, USA

**Keywords:** perioperative mortality, surgical outcomes, global surgery, systematic review

## Abstract

**Introduction:**

*The Lancet* Commission on Global Surgery proposed the perioperative mortality rate (POMR) as one of the six key indicators of the strength of a country’s surgical system. Despite its widespread use in high-income settings, few studies have described procedure-specific POMR across low-income and middle-income countries (LMICs). We aimed to estimate POMR across a wide range of surgical procedures in LMICs. We also describe how POMR is defined and reported in the LMIC literature to provide recommendations for future monitoring in resource-constrained settings.

**Methods:**

We did a systematic review of studies from LMICs published from 2009 to 2014 reporting POMR for any surgical procedure. We extracted select variables in duplicate from each included study and pooled estimates of POMR by type of procedure using random-effects meta-analysis of proportions and the Freeman-Tukey double arcsine transformation to stabilise variances.

**Results:**

We included 985 studies conducted across 83 LMICs, covering 191 types of surgical procedures performed on 1 020 869 patients. Pooled POMR ranged from less than 0.1% for appendectomy, cholecystectomy and caesarean delivery to 20%–27% for typhoid intestinal perforation, intracranial haemorrhage and operative head injury. We found no consistent associations between procedure-specific POMR and Human Development Index (HDI) or income-group apart from emergency peripartum hysterectomy POMR, which appeared higher in low-income countries. Inpatient mortality was the most commonly used definition, though only 46.2% of studies explicitly defined the time frame during which deaths accrued.

**Conclusions:**

Efforts to improve access to surgical care in LMICs should be accompanied by investment in improving the quality and safety of care. To improve the usefulness of POMR as a safety benchmark, standard reporting items should be included with any POMR estimate. Choosing a basket of procedures for which POMR is tracked may offer institutions and countries the standardisation required to meaningfully compare surgical outcomes across contexts and improve population health outcomes.

**Key questions**

**What is already known?**

- Previous systematic reviews of anaesthetic mortality and mortality in specific surgical populations have shown decreasing mortality trends over time and differences by world region.
- Geographical differences have similarly been reported in cohort studies such as the GlobalSurg I study, the European Surgical Outcomes Study and the African Surgical Outcomes Study.

**What are the new findings?**

- This is the first systematic review to attempt broad baseline estimation of perioperative mortality rate (POMR) across procedures and describe how low-income and middle-income countries (LMICs) authors define POMR.
- We show here that POMR varies widely by procedure or diagnosis; further, we show significant variation in how POMR is reported, limiting comparisons across contexts.

**What do the new findings imply?**

- POMR is widely used and reported in all contexts; to promote its utility as a standardised surgical safety indicator, greater specificity in the types of procedures assessed and the way in which data are collected, risk adjusted and reported is required.

## Introduction

Over 260 million surgical procedures are performed each year globally, but a further 143 million procedures are required to meet essential surgical needs in low-income and middle-income countries (LMICs).^1^ In addition to increasing surgical access in LMICs, efforts should also focus on improving the quality and safety of surgical care and reducing the risk of death in the perioperative period.

The perioperative mortality rate (POMR), defined as the number of deaths during or after surgery divided by the number of procedures performed, has been championed in the literature as a useful indicator to measure surgical safety at an institutional and national level.^2–4^

*The Lancet* Commission on Global Surgery recommended the national POMR as one of six key indicators to measure the strength of a country’s surgical system.^2^ However, despite its demonstrated utility in high-income settings, few studies have described POMR across LMICs and little research exists on how POMR is used and defined in resource-constrained settings. Bainbridge *et al* showed decreasing perioperative and anaesthetic-related mortality rates in LMICs since 1970, although procedure-specific rates were not studied or reported.^5^ Uribe-Leitz and others quantified mortality after three common procedures in LMICs: caesarean delivery, appendectomies and groin hernia repair.^6^ A prospective cohort study across 58 countries found that emergency abdominal surgery POMR was three times higher in low-Human Development Index (HDI) compared with high-HDI countries. Most recently, the African Surgical Outcomes Study found that despite being younger, with a lower surgical risk profile and undergoing less complex surgeries, patients in Africa are twice as likely to die after surgery when compared with outcomes at the global level.^7^ Nonetheless, to our knowledge, no study has reported procedure-specific POMR across a wide range of surgical conditions across LMICs.

To address this gap, we undertook a systematic review of the perioperative mortality literature for all surgical procedures in LMICs. We reviewed all studies on POMR published in LMICs over a 6-year period between 2009 and 2014. This covers the period roughly between the publication of the WHO Guidelines for Safe Surgery 2009 and *The Lancet* Commission on Global Surgery, providing a modern account of the POMR literature.^2 8^

This study had several aims: (1) to describe POMR across a wide range of surgical procedures in LMICs, (2) to determine whether these rates vary across contexts and (3) to determine how POMR is defined, reported and used in the LMIC literature, including how risk adjustment is undertaken. Finally, we provide recommendations for improving POMR reporting in resource-constrained settings.

## Methods

### Search strategy and selection criteria

The original study protocol was published alongside a preliminary abstract in 2015,^9^ the final version of which is available in the online Supplementary material protocol. We conducted a systematic review and meta-analysis following Preferred Reporting Items for Systematic Reviews and Meta-Analyses and Meta-analysis Of Observational Studies in Epidemiology guidelines.^10 11^ We included published studies primarily reporting facility-based outcomes or mortality for patients who underwent surgery in LMICs (defined according to 2013 World Bank Income Groups).^12^

10.1136/bmjgh-2018-000810.supp1 Supplementary file 1

All study designs (descriptive, case–control, cohort or trial) were eligible for inclusion. We included full-text articles published in English between 1 January 2009 and 31 December 2014. Final searches were performed on 10 January 2015. The perioperative period was defined as the period from entry into the operating theatre to either discharge or 30 days following a surgical procedure, whichever was later. However, studies explicitly discussing surgery-related mortality, but whose shortest reporting interval was 31–90 days after surgery were included. *Surgery* was defined as any procedure performed in an operating theatre. A list of excluded procedure types is available in the attached review protocol. Only studies providing raw mortality data were eligible for inclusion; those in which the numerator (deaths) or denominator (patients or procedures) were estimated or modelled were excluded. We did not impose a large sample size requirement that would exclude literature published in smaller centres with lower surgical volumes.

We searched PubMed, EMBASE, LILACS, Web of Science, African Index Medicus and the WHO Global Health Library. Search terms for all databases were developed in consultation with a medical librarian. Variants of ‘surgery’, ‘operation’, ‘anaesthesia’, ‘intraoperative’, ‘perioperative’, ‘postoperative’ and ‘mortality’ were included in all searches. In addition, we also hand-searched the references of recently published reviews of specific procedures.^6 13–16^ Stand-alone abstracts and unpublished studies were excluded from the review. Full inclusion and exclusion criteria, as well as database-specific searches, are provided in the attached review protocol.

### Data extraction, outcome definition and procedure classification

Titles and abstracts were reviewed independently in duplicate to evaluate for inclusion. Eligibility assessment based on full-text reviews and data abstraction were done by two clinicians. Selected variables including the surgical procedure or diagnosis under consideration, the perioperative mortality rate, case urgency and the definition of POMR were extracted in duplicate. Disagreements in data extraction were resolved by a single physician coder (JNK). A data dictionary describing all variables, codes, assumptions and simplifications is provided in the online Supplementary data dictionary.

10.1136/bmjgh-2018-000810.supp2 Supplementary file 2

The primary outcome of interest was the POMR, and secondary outcomes were the definition of perioperative mortality and the reporting and adjustment for selected preoperative risk factors. When the time frame relative to surgery during which deaths accrued was not clearly defined, it was assumed to be in-hospital mortality. If mortality was reported at multiple postoperative intervals, the longest (up to 30 days) was used.

To describe each patient population accurately and consistently, coding was performed iteratively. First, a clinician identified the broadest procedure or diagnosis group in each study and assigned it a code. A list of such codes was developed and revised by a second clinician. Within each code, we identified studies performed in high-risk populations (eg, restricted to patients with comorbidities such as renal dysfunction or HIV). When possible, we also stratified patients by case urgency.

### Economic variables and risk of bias

We obtained country lending classification data from the World Bank and Human Development Index data from the UN Development Programme.^17 18^ Where data were unavailable for a given country for a given year, the closest available year to the midpoint year of data collection was used. Two potential sources of bias were assessed: selection bias resulting from failure to report on all consecutive cases and detection bias resulting from failure to provide complete follow-up data. The data collected were analysed as case-series outcomes (mortality rates), regardless of the underlying study design.

### Statistical analysis

In order to summarise POMR across procedures, we pooled estimates using random-effects meta-analysis of proportions and the Freeman-Tukey double arcsine transformation to stabilise variances.^19^ This procedure allows for the inclusion of studies with a zero event rate. Meta-analyses were weighted by the inverse variance of the transformed estimates, giving more weight to the more precise rates in the pooled estimate. We used the *metaprop* command in Stata/IC V.13.^19^ In order to determine whether there were differences in procedure-specific POMR across study-country income groups and HDI categories, we used the non-parametric Kruskal-Wallis equality-of-populations test.^20^

### The role of the funder

This study was funded in part by Boston Children’s Hospital, which had no role in the design, conduct, analysis or writing of this study and did not influence the decision to publish.

## Results

**Figure 1.**
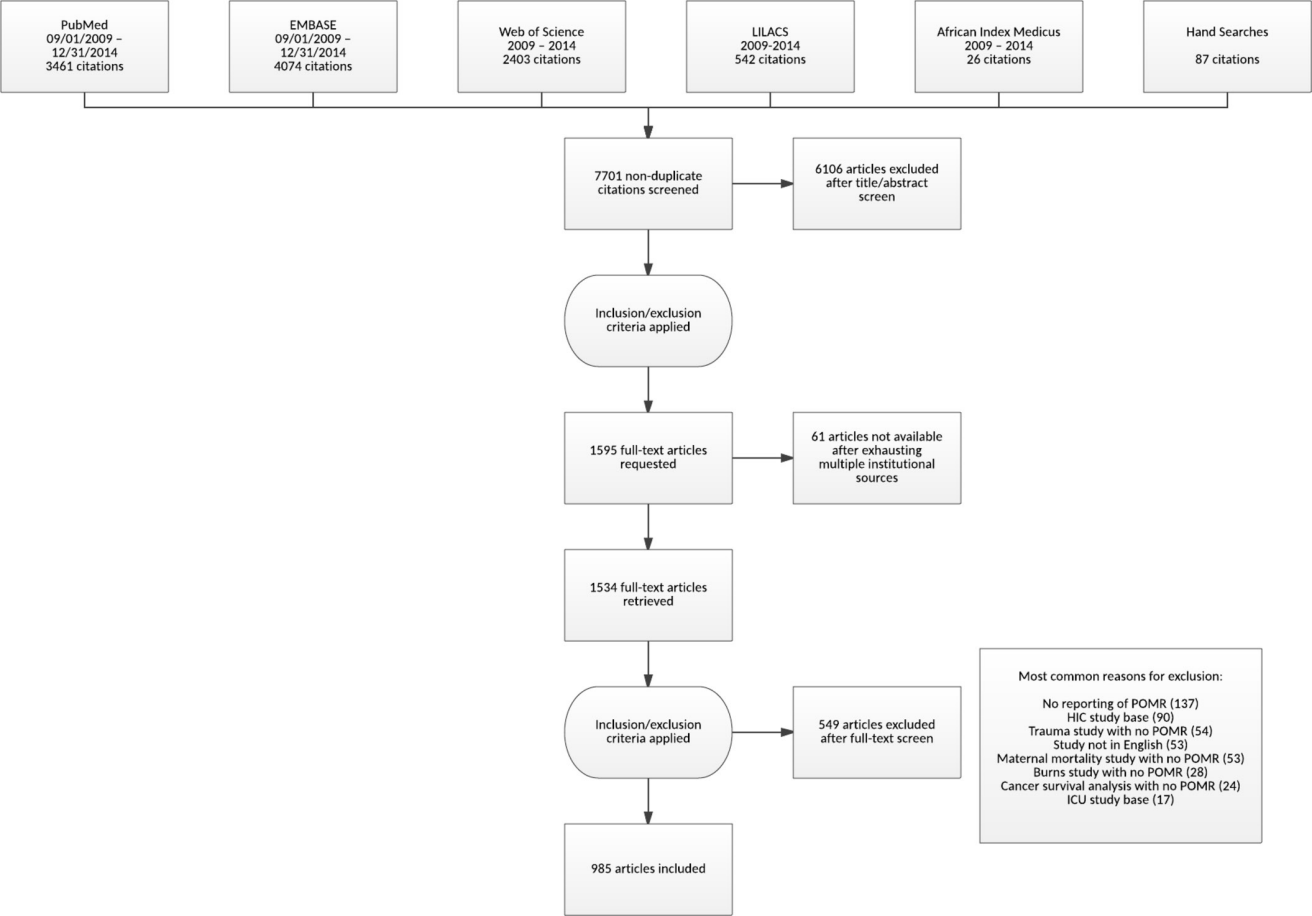
Flow diagram. Preferred Reporting Items for Systematic Reviews and Meta-Analyses flow diagram. HIC, high-income country; ICU, intensive care unit.

**Figure 2.**
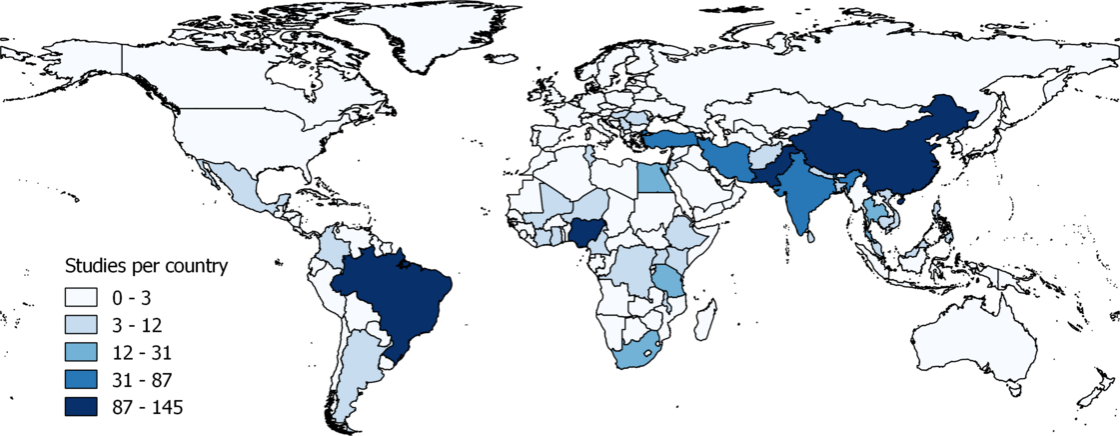
Distribution of the perioperative mortality rate (POMR) literature in low-income and middle-income countries. The number of papers presenting POMR data for each country.

After the removal of duplicate citations, we screened the titles and abstracts of 7701 unique citations. Of these, we requested 1595 full-text articles for further review. A total of 985 articles met our inclusion criteria (figure 1). These studies were conducted across 83 LMICs. The country where the most POMR literature was published was Brazil (145 articles), followed by Nigeria (121), China (111), Pakistan (107) India (87), Turkey (65) and Iran (62) (figure 2; online Supplementary appendix 1 table S1). These studies covered a total of 191 different procedure or diagnostic groups (‘procedures’) in 13 surgical specialties and ranged from small case series of five patients to expansive registries with 152 110 surgical patients (median, 86 patients, IQR 36–234, Supplementary appendix table S2). In total, the surgical outcomes of 1 020 869 patients were included (figure 2). Most studies were conducted in urban environments (n=884, 89.7%) and in academic centres (n=821, 83.4%). The majority of studies were descriptive (n=711, 72.2%) and retrospective (n=685, 69.5%, table 1). Primary data are available in the online Supplementary appendix 2.

10.1136/bmjgh-2018-000810.supp3 Supplementary file 3

10.1136/bmjgh-2018-000810.supp4 Supplementary file 4

**Table 1:** Hospital and study descriptors

Hospital descriptors, n(%)	Academic hospital	821 (83.4%)
	District or community hospital	67 (6.8%)
	Mixed hospital types	74 (7.5%)
	Other	23 (2.4%)
Hospital location	Urban	884 (89.7%)
	Rural	25 (2.5%)
	Mixed locations	76 (7.7%)
Study design	Retrospective	685 (69.5%)
	Prospective	266 (27.0%)
	Ambispective	34 (3.5%)
	Audit	711 (72.2%)
	Non-randomised cohort	225 (22.8%)
	Case–control	24 (2.4%)
	Randomised controlled trial	25 (2.5%)
Urgency	Planned	292 (29.6%)
	Emergent	415 (42.1%)
	Mixed	338 (34.3%)

‘Other’ hospital types include facilities run by Médecins Sans Frontières. ‘Planned’ and ‘Emergent’ rows include studies providing mortality stratified on urgency and therefore totals exceed 100%.

Across the 191 procedures identified, the most commonly reported were caesarean delivery (55 studies) followed by coronary artery bypass grafts (49 studies), emergency peripartum hysterectomies (39 studies) and cardiac valve procedures (35 studies) (table 2). A total of 77 procedures identified were reported in only one study (online Supplementary table s3). Among procedures described in at least four studies (n=67), most demonstrated significant between-study heterogeneity in reported POMR (I^2^>50%, n=55).

**Table 2:** Inverse-variance aggregated perioperative mortality rate (POMR) across the 34 most commonly reported procedures or diagnoses described in the low-income and middle-income countries surgical outcomes literature, 2009–2014

Diagnostic or procedure code	Description	Number of studies	Total number of deaths	Total denominator	Median POMR (range, %)	Inverse-variance aggregated POMR (%, 95 CI)	I-Squared (%)
CAES	Caesarean section	55	805	3 66 501	0.12 (0–15.61)	0.05 (0 to 0.13)	93.52
CABG	Coronary artery bypass graft	49	5807	1 23 513	3.60 (0–52.81)	4.38 (3.47 to 5.37)	97.32
EPH	Emergency peripartum hysterectomy	39	196	2245	10.30 (0–31.03)	7.81 (5.81 to 10.04)	64.27
VALVE	Cardiac valve procedures	35	1197	30 365	4.29 (0–15.07)	4.17 (3.05 to 5.45)	93.61
CARD	Cardiac surgery, not otherwise specified	31	7847	1 23 060	4.79 (0–23.08)	4.96 (3.81 to 6.25)	97.80
COLRES	Colon resection, excluding resection for volvulus	27	356	12 636	1.86 (0–33.64)	2.83 (1.62 to 4.31)	92.35
APPY	Appendicitis	23	28	5237	0 (0–2.78)	0.01 (0 to 0.19)	37.74
LUNGRES	Pulmonary resection, excluding resection for tuberculosis	23	96	5630	1.14 (0–13.76)	1.3 (0.48 to 2.41)	84.46
PERF	Perforated hollow viscus, excluding perforations secondary to salmonella typhi infection	22	264	2427	11.85 (0–40.00)	11.85 (8.35 to 15.83)	84.73
LIVRES	Hepatic resection	20	99	7243	1.38 (0–13.16)	1.04 (0.32 to 2.02)	77.08
MULTI	Multispecialty patient population, usually institution-level surgical mortality	19	1645	1 77 283	1.06 (0.16–7.36)	1.29 (0.77 to 1.94)	99.02
PCARD	Paediatric cardiac procedures, excluding complex congenital heart disease and valve-specific procedures	19	538	6618	7.14 (0–24.24)	6.76 (4.99 to 8.75)	81.36
RIM	Resection of intracranial mass	19	20	814	0 (0–5.88)	1.29 (0.41 to 2.51)	0.00
GASTCA	Gastric cancer	18	267	8250	2.71 (0–18.97)	3.72 (1.92 to 6.01)	94.48
INGHERN	Inguinal hernia	17	71	11 196	0 (0–9.73)	0.38 (0 to 1.22)	93.48
LAPAR	Laparotomy, not meeting other abdominal surgery codes. Includes laparotomy performed for trauma	17	354	3064	11.11 (4.94–42.11)	12.53 (9.39 to 16.04)	83.70
PAED	Paediatric surgical procedures, not otherwise specified	17	355	54 389	3.57 (0–62.22)	6.16 (4.06 to 8.64)	98.64
CHOLE	Cholecystectomy	15	4	6088	0 (0–0.15)	0 (0 to 0)	0.00
UTRUP	Uterine rupture	15	87	1169	8.22 (0–17.50)	7.36 (4.42 to 10.88)	71.74
ESOCA	Esophageal carcinoma	13	134	1802	5.81 (0–24.00)	5.4 (2.28 to 9.54)	89.16
CCHD	Complex congenital heart disease	12	93	596	14.65 (0–61.54)	14.94 (7.03 to 24.75)	83.32
BOBS	Bowel obstruction	10	137	1158	8.79 (2.27–38.10)	12.32 (6.77 to 19.15)	89.13
ICH	Intracranial haemorrhage	10	224	1011	25.48 (3.77–62.22)	24.47 (15.88 to 34.16)	89.05
LIVTRAUM	Hepatic trauma	10	133	909	17.01 (6.80–61.11)	15.84 (10.31 to 22.16)	76.86
WHIP	Whipple pancreaticoduodenectomy	10	90	2065	2.84 (0–9.92)	2.94 (1.61 to 4.57)	52.48
MIS	Minimally invasive surgery, not otherwise specified	9	2	1314	0 (0–4.17)	0 (0 to 0.1)	0.00
RECTAL	Rectal resection	9	6	1032	0 (0–5.88)	0.07 (0 to 0.92)	50.09
SPINE	Spine surgery, excluding trauma	9	11	518	0 (0–8.96)	0.77 (0 to 3.8)	67.18
TIP	Typhoid intestinal perforation	9	134	662	20.73 (4.55–33.33)	20.09 (14.36 to 26.48)	71.46
AABDO	Acute abdomen but not meeting other abdominal surgery codes	8	228	2877	10.42 (4.90–34.88)	11.2 (7.42 to 15.62)	86.27
ACHI	All-comer head injury	8	377	1390	23.08 (10.00–54.58)	27.2 (14.98 to 41.39)	96.32
BILD	Bile duct procedures, excluding Whipple procedure	8	51	714	2.30 (0–21.54)	4.08 (0.1 to 11.63)	91.49
INTUSS	Intussusception	8	43	355	3.66 (0–33.70)	4.8 (0.03 to 14.28)	88.09
TAD	Thoracic aortic disease	8	775	4203	8.66 (0–20.30)	9.5 (3.96 to 16.74)	91.49

While acknowledging the significant between-study heterogeneity within procedures resulting from differences in methodology, outcome definition and patient-level risk, we elected to create pooled POMR estimates as an approximate baseline to inform future research. Procedure-specific POMR pooled by inverse-variance-weighted random-effects meta-analyses for the 34 most commonly reported procedures (ie, reported in at least eight studies) are shown in table 2. Given the high between-study heterogeneity, we also include the median study-level POMR for each procedure and the range across which studies varied.

POMR varied considerably between procedures. For example, pooled POMR ranged from less than 0.1% for cholecystectomy to 20%–27% for typhoid intestinal perforation, intracranial haemorrhage and operative head injury (table 2). While caesarean delivery POMR was a mere 0.05% (95% CI 0 to 0.13), small outlier studies reported rates of up to 16%. After emergency peripartum hysterectomy, however, the risk of dying was 7.81% (95% CI 5.81 to 10.04). Similarly, the risk of dying after appendectomy was 0.01% (95% CI 0 to 0.19) whereas, after surgery for a perforated hollow viscus (excluding typhoid), POMR was 11.85% (95% CI 8.35 to 15.83). Paediatric surgical procedures demonstrated alarmingly high mortality rates: surgery for oesophageal atresia/tracheo-oesophageal fistula carried a 24.41% mortality risk (95% CI 6.76 to 48.04), Hirschsprung’s disease 10.65% (95% CI 0.42% to 29.11%), intestinal atresias 30.95% (95% CI 18.71 to 44.53) and gastroschisis 29.68 (95% CI 10.75 to 53.14).

Mortality rates across all 191 identified procedures are shown in the online Supplementary table s3. As a sensitivity analysis, we excluded all studies performed in high-risk populations (such as studies restricted to patients with specific comorbidities); results are shown in the online Supplementary table s4. There was a little change in pooled POMR estimates after excluding these studies.

We looked at whether procedure-specific POMR varied by study country income group or categories of HDI. For this analysis, procedure-specific POMR estimates for high-income countries were identified through a purposive search of the literature. We found a statistically significant difference in POMR for emergency peripartum hysterectomy across income groups (p<0.05). This relationship was not statistically significant for any other procedure types including caesarean section, appendectomy and colon resection. We found no consistent association between procedure-specific POMR and HDI.

Over half of the studies (n=530, 53.8%, table 3) did not provide a clear POMR definition (ie, of the timeframe during which deaths accrued). In the other studies, a variety of timeframes for calculating POMR were employed. About 20% of studies reported 30-day mortality and a smaller number referred to variants thereof (such as ‘mortality within 30 days of surgery or during the index hospital stay’). Some obstetric surgery studies reported mortality at 42 days after surgery (n=6). Most studies used the number of patients undergoing surgery, rather than the number of procedures performed, as the denominator of POMR (n=969, 98.4%). We also found that studies performed in upper-middle income countries were more likely to provide a clear definition of the POMR described.

**Table 3:** Definitions of perioperative mortality rate

	Number of papers (%)
**Numerator**	
Clearly defined	455 (46.2)
Inpatient/hospital mortality (assumed for all studies lacking clear definition)	703 (71.4)
Inpatient/hospital mortality, within 30 days of procedure	13 (1.3)
30-day mortality	202 (20.5)
Mortality within 30 days or same hospitalisation	32 (3.3)
7-day mortality	3 (0.3)
Intraoperative mortality	14 (1.4)
24 hours mortality	4 (0.4)
Other	14 (1.4)
Multiple	24 (2.4)
**Denominator**	
Number of patients	969 (98.4)
Number of procedures	16 (1.6)

Most studies did not explicitly state the timeframe during which deaths accrued. For those studies lacking clear time definitions, deaths were assumed to accrue during the index hospitalisation alone.

Risk-adjustment methodology varied widely (table 4). Some studies reported gross mortality rates without risk adjustment; by contrast, authors from Brazil and China used detailed registry data to develop sophisticated population-specific scores to determine risk after cardiac surgery. Most studies reported median patient age (95.0%) and case urgency (74.1%), but only 7.5% reported the ASA status (American Society of Anesthesiologists Physical Status Classification). About a third of studies (n=331) reported a clinical risk score, but only 14.3% performed risk adjustment or stratification based on such scores. A summary of the scores used by surgical specialty is included in the online Supplementary appendix 1 table S5.

**Table 4:** Risk factor reporting and adjustment

	Number of studies reporting (n, %)	Number of studies providing adjustment or stratification (n, %)
Patient age	936 (95.0)	145 (14.7)
Comorbidities	402 (40.8)	146 (14.8)
ASA status	74 (7.5)	33 (3.4)
Case urgency	730 (74.1)	693 (70.4)
HIV status	45 (4.6)	25 (2.5)
Clinical Risk Score	331 (33.6)	141 (14.3)

Reporting of case urgency required presentation of the proportion of planned versus emergent cases, or a population consisting exclusively of either planned or emergent cases. The latter group was considered to have reported mortality ‘stratified’ on urgency. ‘Adjustment or stratification’ implies a statistical analysis of the risk factor in relation to mortality, or mortality provided for separate strata.

## Discussion

In LMICs, the POMR literature is as diverse as the institutions and countries that produce it. It spans all surgical specialties and a wide variety of procedures and diagnoses both common and rare. To our knowledge, this is the first systematic review to report procedure-specific POMR across a wide range of surgical conditions in LMICs. This review included data from 985 studies conducted in 83 LMICs and covering 191 types of surgical procedures performed on a population of 1 020 869 patients.

POMR is used for many purposes. In the studies included here, authors used POMR data to argue for increased critical care resources,^21^ quantify the particular surgical risk for the HIV-positive population,^22^ assess the impact of delay in reaching care on outcomes,^23^ raise alarm over high mortality rates in the paediatric population^24 25^ and establish the relative safety of traditionally high-risk procedures in select LMIC environments, among other aims.^26^ The utility of this metric at the institution level is undeniable; with clear outcome definitions, a well-defined population, and robust analysis, perioperative mortality rates can be used to monitor and improve patient safety. To demonstrate patterns in mortality rates beyond the level of the institution requires some standardisation of definitions, methods of data capture and patient-level risk assessment. The studies included here were too heterogeneous on these fronts to demonstrate stable relationships between POMR and macroeconomic variables such as HDI or income groups.

By contrast, the GlobalSurg group demonstrated a clear inverse relationship between POMR and HDI for emergency abdominal surgery, and a previous systematic review by Bainbridge *et al*, showed similar findings for all-comer anaesthesia-related mortality.^5 27^ Assuming this relationship is true, several potential reasons may explain why this study did not demonstrate it. First, unlike Bainbridge *et al*, we report procedure-specific POMR. The small number of studies within each procedure group decreased the power to detect such relationships and prevented us from conducting meta-regression analyses. Second, heterogeneity in POMR definitions across studies introduces significant noise. The impact of differences in POMR definition can be dramatic; in the GlobalSurg analysis, 24 hours mortality was 1.6%, underestimating all-cause 30-day mortality (5.4%) by 70%.^27^ Similarly, in a New Zealand data set, in-hospital mortality underestimated 30-day mortality by about one-third.^28^ Third, most studies included here were retrospective, increasing the risk of information bias or incomplete reporting of mortality data, which may vary by income level. This information bias can be significant. A study in Uganda compared mortality from retrospective chart reviews, surgical logbooks and prospective data collection. Of the 16 deaths identified prospectively, retrospective chart reviews captured only six. Surgical logbooks performed better, capturing 99% of procedures and 15 out of the 16 deaths.^29^ Fourth, individual patient risk was not assessed in this analysis; it is possible that higher-income countries reported on the outcomes of older or more comorbid surgical patients. Fifth, many studies had small sample sizes with few mortalities, resulting in large variances in estimated POMR. Finally, high-income countries were not included in this analysis, narrowing the economic spectrum across which POMR was assessed. We also note that the strength of the relationship between POMR and economic variables is likely to vary by procedure. For procedures with low baseline risk, it may be more difficult to detect a meaningful difference in mortality by level of development.^27^

A clear recommendation to arise from this review is that whenever perioperative mortality data are reported, metadata describing the definition used and the quality of reporting should accompany them (box 1). Because POMR is a proportion (though termed a ‘rate’ in the literature), both the numerator and denominator should be clearly described. The numerator should be described specifically in terms of the time during which deaths accrue, with a preference for *all-cause 30* day deaths where possible. Inpatient mortality misses many deaths.^27 28^ However, it is easier to collect than 30-day mortality, which requires a concerted effort to contact patients at 30 days following surgery. This challenge is the primary reason why *The Lancet* Commission on Global Surgery recommended inpatient perioperative mortality as a key surgical systems indicator.^2^ However, the ubiquity of cellphone technology and its utility in follow-up for surgical site infections in austere environments make it a promising tool for use in collecting POMR data.^30^ Thirty-day POMR seems to be a more robust indicator and it is less sensitive to the varying postsurgery discharge practices around the world. Nonetheless, in some contexts, reporting inpatient mortality will be necessary. In this case, the average length of stay should be reported and authors should specify whether outpatient procedures were included in the denominator. Including outpatient procedures can deflate POMR, as by definition, the outcome (‘inpatient mortality’) is unlikely to occur in an outpatient population (being limited to intraoperative deaths or deaths in the recovery room). In this review, we found that the denominator was most often defined as the number of surgical patients. Alternatively, the denominator can be defined as the number of procedures or number of admissions including a surgical procedure; the impacts on POMR of such subtle changes have been described elsewhere.^28^ Furthermore, the population under study should be clearly described, all consecutive patients included and the completeness of follow-up and reasons for any missing data described.

Box 1 **Critical elements for reporting perioperative mortality rates**

1. Define the surgical population under study.
  - Report all relevant inclusion/exclusion criteria and report if all consecutive patients meeting criteria were included.
  - If multiple procedures are included, report case mix.
2. Report study design.
  - Report study perspective (retrospective, prospective or ambispective).
    - If retrospective, describe how all procedures/patients were identified and whether surgical logbooks, registries, or electronic medical records were used.
  - Describe methodology for any between-group comparisons.
3. State the timeframe during which deaths accrued: in-hospital (during index hospital stay alone), 30-day mortality or other.
  - If in-hospital mortality is used,
    - Report the mean length of hospital stay, standard deviation and range.
    - Report the proportion of included procedures being performed with same-day discharge (‘day surgery’).
4. State the denominator used: surgical patients, surgical procedures or admissions with a surgical procedure.
5. Report any loss to follow-up.
  - If the outcomes of all surgical procedures cannot be identified.
    - Report the proportion of missing data.
    - Report why the data are missing.
6. Report common surgical risk factors including age, comorbidities, functional status, urgency status and ASA.
  - If a validated risk-scoring system is available for the procedure under study, consider using and reporting it.
  - If risk-adjusted mortality is reported, also report crude rates and clearly describe adjustment methodology.

As the scope of analysis of POMR expands from the institutional to the national level, so too does the importance of precision in data collection and reporting. The gross national POMR has been advocated by several groups as a global indicator of surgical safety.^2–4^ The goal of this indicator is to provide a waypost for the improvement of safety in surgical systems. More specifically, it should indicate modifiable operative and postoperative factors that determine mortality, while preoperative factors and data factors are controlled (table 5). Others have argued that while POMR varies with case mix, the operative experience of a country is unlikely to change from year to year; policymakers can therefore monitor POMR over time to assess improvements in surgical safety. This argument relies on two premises: first, that the initial country-level POMR reviewed by a policymaker can be reasonably interpreted to motivate investment in surgical safety, and second, that case mix, definitions and quality of reporting remain stable over time to permit interpretation of changes in reported rates. An effort to collect POMR from ministries of health has been undertaken.^31^ While many countries did provide POMR, these data were difficult to interpret, as they varied in definition and case mix. It was not possible to assess whether a country was performing well, or poorly, compared with others based on the gross national POMR data provided. Even when definitions and methods of data capture are held constant, careful reporting of case mix remains important: an analysis by region in Brazil showed higher all-procedure POMR in wealthier regions, but when caesarean section was analysed in isolation, wealthier regions had lower POMR.^32^ We have shown here that POMR varies by orders of magnitude according to which procedures are being studied. Even within such broad categories as ‘emergency intraperitoneal surgery’ as studied by GlobalSurg, we have demonstrated that mortality rates vary widely according to which specific procedure or diagnosis is under study.

**Table 5:** Known or potential factors influencing perioperative mortality rates

Preoperative factors		Operative factors	
Patient factors	Comorbidities	Urgency	Planned
	Age		Emergent
	Severity and nature of illness		
Health systems factors	Prehospital transport	Surgical approach	Open
	Delay to presentation		Minimally invasive
	Appropriate centre for condition	Intrinsic procedure risk	By specialty
			By procedure
			By complexity score
		Surgeon skill	Specialist versus non-specialist surgeon
			Surgeon versus non-surgeon physician
			Physician versus non-physician surgeon
			Trainee versus fully-trained surgeon
			Inter-surgeon variation
		Anaesthetic modality	General, regional, local
		Anaesthetist skill	Specialist versus non-specialist anaesthetist
			Anaesthetist versus non-anaesthetist physician
			Physician versus non-physician provider
			Trainee versus fully-trained anaesthetist
			Inter-anaesthetist variation

Postoperative factors		Data factors	
Discharge pathway	Outpatient surgery	Database type	Administrative
	Same-day admission		Retrospective collection
	Inpatient surgery		Prospective collection
			Dedicated surgical outcomes registry
Postoperative surveillance for complications	Nursing availability and level of training	Risk adjustment methods	
	Provider: patient ratio		Crude measures reported
	Frequency of physician assessments		Risk-adjusted outcomes reported
	Availability of diagnostic testing		Risk-stratified outcomes reported
Ability to rescue after complications	Availability of preoperative care	Definition of POMR	Intraoperative deaths
	Availability of intravenous antibiotics		Deaths within 24 hours of surgery)
	Availability of blood bank		Inpatient deaths
	Availability of image-guided interventions		30 day deaths
	Availability of critical care beds		>30 day deaths
	Availability of ventilators		Deaths attributable to surgery versus all-cause deaths
	Availability of dialysis		Patients, procedures, or surgical admissions as denominator
	Availability of cardiac interventions		

Based on these findings, we advocate for greater specificity in the standard definition of POMR to be used by hospitals and countries. A selection of indicator procedures might be chosen to cover the lifespan, such as surgery for gastroschisis, caesarean section, colon resection and repair of hip fracture. Each of these procedures is performed at all levels of HDI, is studied widely and has excellent science on how risk adjustment should be performed. The tradeoff encountered by focusing on POMR indicator procedures is wider CIs due to a lower number of events (compared with all-patient nationwide POMR). Again, we argue that wider CIs around indicators that are meaningful to policymakers at face value are preferable to narrower CIs around a gross indicator that is agnostic to case mix. Yet, to abandon the impetus to track all postoperative deaths might be short sighted. Robust surgical registries tracking all procedures have been established in low-income countries.^33^ An expansion of such efforts to all hospitals would allow for tracking of the outcomes of select global index procedures and locally important procedures, in addition to improving nationally representative surgical volume estimates. Other recommendations for the use of POMR to advance surgical safety are included in box 2.

Box 2 **Recommendations for improving surgical safety**

**Clinicians, care providers and hospitals**

1. Promote the implementation of best practices, such as the WHO guidelines for safe surgery and other procedure-specific or context-specific evidence to decrease complications. Develop a local culture of safety with regular quality-of-care discussions.
2. Develop quality improvement networks across settings to work collectively to identify and implement strategies to improve safety and decrease perioperative mortality rate (POMR).
3. Invest in the technology and human resources required for the prospective collection and analysis of POMR data.

**Researchers**

Globally

1. Choose indicator procedures that are commonly performed across all settings, have a significant mortality risk, are representative of the burden of surgical disease across the population and have good existing science for risk adjustment.
2. Determine sample sizes for these procedures required to stably estimate gross and risk-adjusted procedure-specific POMR at the facility and country level.
3. Study these procedures across settings to estimate how differences in data collection methods and the definition of POMR influence estimates.
4. Conduct global studies to establish nationally representative estimates of POMR for each indicator procedure.
5. Advocate for investment in the technology and human resources required for reliable ongoing collection of POMR data.

Locally

1. Study indicator procedures in depth to develop local solutions to patient safety problems that can be scaled regionally, nationally or globally.

**Ministries of Health:**

1. Provide facilities with the administrative and financial support required to collect POMR data prospectively.
2. When higher-than-expected POMR is brought to the attention of the ministry, mobilise additional clinical and financial resources to augment the safety of operative and postoperative care.

While POMR is useful for assessing the safety of operative care, contextualising it within the broader suite of six surgical indicators proposed by *The Lancet* Commission on Global Surgery is critical.^2^ Specifically, juxtaposing surgical volume with POMR allows for an assessment of the quantity of care delivered and the safety thereof. Consider an individual with a typhoid intestinal perforation: our analysis would estimate a POMR of 20% in LMICs. This is still vastly preferable to the reported 70% mortality with non-operative management.^34^ Universal access to the operating room is a dominant determinant of public health, with surgical safety an important secondary determinant of outcome. This was made strikingly clear in the recently published *African Surgical Outcomes Study.*^7^ A sweeping cohort-based study providing complication and mortality data representative at the level of the continent, it showed that surgical patients in Africa have twice the in-hospital POMR of an international cohort despite a favourable risk profile. POMR was particularly elevated after an index complication. However, in the study, hospitals were only able to deliver 212 surgical procedures per 100 000 population per annum, well short of the *5000 per 100 000* target set by *The Lancet* Commission on Global Surgery. While working to improve safety, focus must be maintained on scaling up delivery and bolstering the surgical workforce in low-volume settings.

An important caveat to this discussion is that POMR may be less useful for surgical conditions in which patients do not consistently require a trip to the operating theatre. For example, numerous studies reporting deaths after trauma were excluded from this analysis as a specific operative numerator and denominator were not reported. Finally, measurement alone is not enough.^35^ While measuring POMR is indeed the first step towards reducing mortality rates, clinicians and policymakers must insist on deploying the resources and best practices required to prevent complications and rescue patients who experience them.

This study had limitations. First, given the high between-study heterogeneity within procedure types, the pooled POMR estimates should be interpreted with caution. Second, this review may be subject to publication bias. Studies of particularly high mortality may not have been submitted for publication in the interest of protecting institutional or surgeon reputation. Conversely, studies of low mortality may not have been deemed worthy of publication. Funnel plots are problematic in meta-analyses of proportions where the proportions are small.^36^ We were unsurprised to find that in this group of highly heterogeneous studies, constructing funnel plots, whether conventional funnel plots or those using study size versus log odds, failed to shed light on the file-drawer problem. Further, these results are most representative of mortality at academic medical centres and may not reflect mortality at smaller, more rural sites. Finally, this analysis included only studies published in English over a 6-year period.

Some conditions continue to cause high surgical mortality in LMICs, particularly in the paediatric population. Mortality data are commonly reported in the LMIC surgical literature but the quality of reporting varies widely: more than half of the studies did not provide a clear definition of the outcome. Given that mortality rates differ dramatically by the procedure or diagnosis under study, analysis of mortality rates for a select basket of surgical procedures might add validity to POMR and allow for constructive comparison of outcomes between sites and countries.
