# Supplementary material for: Perioperative mortality rates in low-income and middle-income countries: a systematic review and meta-analysis
Source: BMJ Glob Health. 2018 Jun 22;3(3):e000810. doi: 10.1136/bmjgh-2018-000810 (PMC6035511; doi:10.1136/bmjgh-2018-000810)
Supplement: Supplementary file 1 [file bmjgh-2018-000810supp001.pdf]

Perioperative mortality rates in low- and middle-income countries

A SYSTEMATIC REVIEW

FINAL PROTOCOL, WITH UPDATES JAN 2017

OBJECTIVES

1. To evaluate reported perioperative mortality rates (POMR) across procedures and diagnoses in low- and middle-income countries (LMIC).
2. To determine which definition of POMR is most commonly used in the LMIC literature.
3. To determine how POMR has been used in the LMIC literature, elaborating the types of studies performed and questions answered using POMR as an indicator.
4. To determine how risk adjustment for case mix, patient age, and disease severity have been undertaken.

BACKGROUND

This project is being undertaken as an investigation for the Lancet Commission on Global Surgery. Following on the work of Watters, et al, which recommended the global use of Perioperative Mortality Rate (POMR) as an "indicator of access to and safety of surgery and anesthesia", the need for a systematic review on the state of POMR as a surgical indicator was identified.[1]

METHODS

Selection criteria

Inclusion Criteria:
Any published paper primarily reporting facility-based outcomes or mortality for patients who have undergone surgery in a low- or middle-income country (LMIC) per the 2013 World Bank Country Classification. Any study design (audit, case-control, or cohort) in which such data are presented is eligible for inclusion. The paper must report perioperative mortality rates within a specified patient population.

Filters:

- English
- Publication dates: Jan 1, 2009 to December 31, 2014

For the purposes of this analysis:

- "perioperative" refers to the time period from entry into the operating room to either discharge, or 30 days following a surgical procedure (whichever comes later). This is extended to 42 days for outcomes following caesarean section.
- "surgery" refers to a procedure performed in an operating theatre
- LMIC are defined by World Bank Country and Lending Groups and include upper-middle, lower-middle, and low-income countries.

Exclusion Criteria:
Interventions: Percutaneous vascular interventions, percutaneous cardiovascular interventions, percutaneous nephrolithotomy*, transplantation, robotic operations, ECMO, bariatric surgery, trauma sustained during military engagement, surgery performed in temporary combat hospitals, endoscopic interventions*, HIPEC, cosmetic plastic surgery, short-term medical missions, studies of elective abortion, burr holes without further surgical intervention.

Paper Types: survival analysis for cancer (with no discussion of POMR), survival analysis for pericardial effusion, risk factor model assessment without provision of actual mortality rates, trauma/burn mortality studies without reporting of perioperative mortality, maternal mortality studies without reporting of perioperative mortality, studies whose denominator is primarily drawn from an ICU study base, studies of fetal surgery, dental surgery (studies of maxillofacial surgery were not excluded), studies with denominator <5 patients.

N.B. Aug 22, 2016 added percutaneous nephrolithotomy to exclusion list
N.B. Jan 24, 2017, clarified "colonoscopy" to "endoscopic interventions", referring to procedures performed through an endoscope, gastroscope, nasoscope, or neuroendoscope

Outcome Measures
Primary Outcome: Perioperative mortality rates

Secondary Outcomes:

- Proportion of studies reporting various definitions of POMR, including intraoperative mortality, 24-hour mortality, 7-day perioperative mortality, inpatient mortality, and 30-day mortality.
- Proportion of studies reporting other perioperative complications
- Proportion of studies reporting preoperative risk factors including patient age, comorbidities, ASA status, case urgency, HIV status, and clinical risk scores.

Search Methods
Electronic searches

We will search the following databases:

- PubMed
- EMBASE
- LILACS
- Web of Science
- African Index Medicus
- WHO Global Health Library

Data Collection and Analysis
Selection of Studies

Titles and abstracts will be reviewed in duplicate (SLMG, MK, JNK) to identify studies reporting:

- In-hospital or facility-based mortality or outcomes or results pertaining to surgery or surgically treated diseases
- In-hospital or facility-based mortality pertaining to anesthesia
- In-hospital or facility-based maternal mortality with specific mention of cesarean section

Studies warranting full-text review will be read by trained data extractors to evaluate for inclusion or exclusion, with final oversight by JNK.

Data extraction

The following data will be extracted from all papers using a piloted Excel form

1. Year of publication
2. Study design
3. Start and end dates of study period
4. Country location of participating hospitals
5. Facility type (academic hospital, community hospital, district hospital, mixed hospital types, other)
6. Description of the patient population
7. Type of anesthesia used
8. Definition of POMR employed (including timeframe, numerator, and denominator).
9. Whether or not the study reports HIV status, case urgency, comorbidities, clinical scoring systems, age, and whether or not mortality is adjusted for or stratified on these factors.
10. Names of any clinical scoring systems used.
11. Difficulties raised by authors in data collection, including loss to follow up and other such missing data.
12. Planned versus emergent status of operative cases. A planned surgery is one in which the patient is admitted from his or her place of usual residence at a pre-set date for the purpose of undergoing a surgical procedure. An emergency surgery is one in which the patient undergoes a surgical procedure after being admitted to hospital on an unforeseen date with a potentially life- or limb-threatening disease process.
13. Perioperative mortality rate. Where such a rate is not calculated by authors, but a defined numerator and denominator are reported, it will be imputed as calculated by reviewers. Where studies of operative and nonoperative patients are included, only mortality for patients actually undergoing surgery will be extracted.
14. Surgical specialty. Where studies are limited to a surgical specialty, the single most appropriate specialty will be assigned according to the case mix reported.
15. Procedure or diagnosis name. This is imputed where studies are limited to a single diagnosis or a single procedure.

Specific variables will be extracted in duplicate. These include the procedure or diagnosis name, whether or not the definition of POMR was clearly described, the definition of POMR, case urgency status, whether or not a study was based in a high-risk population (other than age, urgency, or the nature of the procedure or diagnosis), whether the study was based in a specific age category (neonatal, pediatric, or geriatric), and the reported POMR numerator and denominator.

All other variables will be extracted by a single trained data extractor, with regular oversight and review by the lead reviewer (JNK).

The attached data dictionary includes a description of all variables extracted with simplifications and assumptions outlined.

Risk of Bias

On an individual study level, studies risk selection bias (by failing to represent consecutive cases), or detection bias (by failing to provide complete follow-up data). All studies will be assessed for both such biases.

On a review level, publication bias may influence results, however the direction of such bias is unclear. Studies may tend to come from larger centres with more complex patients. This may either overestimate mortality (due to clinical complexity) or underestimate mortality (due to greater resource availability at such centres). Authors may be reticent to publish audit data showing high mortality (for professional or political reasons), or they may publish studies of riskier procedures more frequently (for reasons of academic interest). Such biases will be addressed qualitatively—for example, if studies are primarily identified from urban, academic centres, results may not be generalizable to smaller rural centres.

Data synthesis

The data collected will be analyzed as case-series outcomes (mortality rates), regardless of the underlying study design. We anticipate significant heterogeneity in mortality rates across clinical groups (specific procedures or diagnoses), and even within clinical groups. We will therefore simply provide reported ranges of POMR for a variety of procedures or diagnostic groups.

Analysis

Data analysis will be undertaken using Stata 13/IC

PRISMA REQUIREMENTS FOR REPORTING

Domain	Requirement	Fulfilments in present work
Data collection process	Describe method of data extraction from reports (e.g., piloted forms, independently, in duplicate) and any processes for obtaining and confirming data from investigators.	Used piloted excel-based form. Updated iteratively as deemed appropriate. Data extracted by trained clinician coders.<br><br>Unlikely to seek data/confirmation from primary investigators (too many papers for this to be feasible)
Data items	List and define all variables for which data were sought (e.g., PICOS, funding sources) and any assumptions and simplifications made.	See Data Dictionary for all variables<br><br>PICOS—Primarily descriptive studies.<br>See variable: patient_population<br>See variable: type_of_study.<br>Where specific exposure studied, see variable: exposure

Risk of bias in individual studies	Describe methods used for assessing risk of bias of individual studies (including specification of whether this was done at the study or outcome level), and how this information is to be used in any data synthesis.	Completeness of data were assessed (to ensure no bias due to loss-to-follow-up)<br><br>Subjective validity/reliability assessment<br><br>Variable: correlation_prediction—an assessment of whether POMR correlated with risk strata.
Summary measures	State the principal summary measures (e.g., risk ratio, difference in means).	POMR by procedure or diagnosis, definitions of POMR used, and strategies for risk adjustment

REFERENCES

1. Watters DA, Hollands MJ, Gruen RL, et al. Perioperative Mortality Rate (POMR): A Global Indicator of Access to Safe Surgery and Anaesthesia. *World J Surg* 2014.

APPENDIX 1: PUBMED SEARCH STRATEGY

A search strategy was designed in consultation with a medical librarian to retrieve all relevant articles. Multiple databases will be searched, starting with PubMed.

The PubMed search string is appended below.

FULL PUBMED QUERY

((("Hospital Mortality"[Mesh] OR "Hospital Mortality"[tiab] OR “Hospital Death”[tiab]) AND ("Surgical Procedures, Operative"[Mesh] OR “surgery department, hospital”[MeSH] OR “General Surgery”[Mesh] OR “Anesthesia”[Mesh] OR "Cesarean Section"[Mesh] OR “surgery”[tiab] OR “surgical”[tiab] OR “anesthesia”[tiab] OR “anesthetic”[tiab] OR “anaesthesia”[tiab] OR “anaesthetic”[tiab] OR "cesarean"[tiab] or "caesarean"[tiab])) OR ("Intraoperative Care/mortality"[Mesh] OR ("intraoperative"[tiab] AND (“mortality”[tiab] OR “death”[tiab])) OR "Postoperative Care/mortality"[Mesh] OR "Postoperative Complications/mortality"[Mesh] OR ("postoperative"[tiab] AND (“mortality”[tiab] OR “death”[tiab])) OR "Perioperative Period/mortality"[Mesh] OR (“perioperative”[tiab] AND (“mortality”[tiab] OR “death”[tiab])) OR (“surgery”[tiab] AND (“mortality”[tiab] OR “death”[tiab])) OR (“surgical”[tiab] AND (“mortality”[tiab] OR “death”[tiab])) OR (“operative”[tiab] AND (“mortality”[tiab] OR “death”[tiab])) OR (“operation”[tiab] AND (“mortality”[tiab] OR “death”[tiab])) OR "Anesthesia/mortality"[Mesh] OR (“anesthesia”[tiab] AND (“mortality”[tiab] OR “death”[tiab])) OR (“anesthetic”[tiab] AND (“mortality”[tiab] OR “death”[tiab])) OR (“anaesthesia”[tiab] AND (“mortality”[tiab] OR “death”[tiab])) OR (“anaesthetic”[tiab] AND (“mortality”[tiab] OR “death”[tiab])) OR ((“cesarean”[tiab] OR “caesarean”[tiab]) AND (“mortality”[tiab] OR “death”[tiab])))) AND (("Developing Countries"[Mesh] OR "Africa South of the Sahara"[Mesh] OR "Central America"[Mesh] OR "Afghanistan"[Mesh] OR "Albania"[Mesh] OR "Algeria"[Mesh] OR "American Samoa"[Mesh] OR "Angola"[Mesh] OR "Antigua and Barbuda"[Mesh] OR "Argentina"[Mesh] OR "Armenia"[Mesh] OR “Azerbaijan”[Mesh] OR "Bangladesh"[Mesh] OR "Republic of Belarus"[Mesh] OR "Belize"[Mesh] OR "Benin"[Mesh] OR "Bhutan"[Mesh] OR "Bolivia"[Mesh] OR "Bosnia-Herzegovina"[Mesh] OR "Botswana"[Mesh] OR "Brazil"[Mesh] OR "Bulgaria"[Mesh] OR "Burkina Faso"[Mesh] OR "Burundi"[Mesh] OR "Cape Verde"[Mesh] OR "Cameroon"[Mesh] OR "Cambodia"[Mesh] OR "Central African Republic"[Mesh] OR "Chad"[Mesh] OR "China"[Mesh] OR "Colombia"[Mesh] OR "Comoros"[Mesh] OR "Congo"[Mesh] OR "Democratic Republic of the Congo"[Mesh] OR "Costa Rica"[Mesh] OR "Cote d'Ivoire"[Mesh] OR "Cuba"[Mesh] OR "Djibouti"[Mesh] OR "Dominica"[Mesh] OR "Dominican Republic"[Mesh] OR "Ecuador"[Mesh] OR "Egypt"[Mesh] OR "El Salvador"[Mesh] OR "Eritrea"[Mesh] OR "Ethiopia"[Mesh] OR "Fiji"[Mesh] OR "Gabon"[Mesh] OR "Gambia"[Mesh] OR "Georgia (Republic)"[Mesh] OR "Ghana"[Mesh] OR "Grenada"[Mesh] OR "Guatemala"[Mesh] OR "Guinea"[Mesh] OR "Equatorial Guinea"[Mesh] OR "Guinea-Bissau"[Mesh] OR "Guyana"[Mesh] OR "Haiti"[Mesh] OR "Honduras"[Mesh] OR “Hungary”[Mesh] OR "India"[Mesh] OR "Indonesia"[Mesh] OR "Iran"[Mesh] OR "Iraq"[Mesh] OR "Jamaica"[Mesh] OR "Jordan"[Mesh] OR "Kazakhstan"[Mesh] OR "Kenya"[Mesh] OR “Kosovo”[Mesh] OR "Micronesia"[Mesh] OR "Democratic People's Republic of Korea"[Mesh] OR "Yugoslavia"[Mesh] OR "Kyrgyzstan"[Mesh] OR "Laos"[Mesh] OR "Latvia"[Mesh] OR "Lebanon"[Mesh] OR "Lesotho"[Mesh] OR "Liberia"[Mesh] OR "Libya"[Mesh] OR "Macedonia (Republic)"[Mesh] OR "Madagascar"[Mesh] OR "Malawi"[Mesh] OR "Malaysia"[Mesh] OR "Indian Ocean Islands"[Mesh] OR "Mali"[Mesh] OR "Mauritania"[Mesh] OR “Mauritius”[Mesh] OR "Mexico"[Mesh] OR "Moldova"[Mesh] OR "Mongolia"[Mesh] OR "Montenegro"[Mesh] OR "Morocco"[Mesh] OR "Mozambique"[Mesh] OR "Myanmar"[Mesh] OR "Namibia"[Mesh] OR "Nepal"[Mesh] OR

"Nicaragua"[Mesh] OR "Niger"[Mesh] OR "Nigeria"[Mesh] OR "Pakistan"[Mesh] OR "Palau"[Mesh] OR "Panama"[Mesh] OR "Papua New Guinea"[Mesh] OR "Paraguay"[Mesh] OR "Peru"[Mesh] OR "Philippines"[Mesh] OR "Romania"[Mesh] OR "Siberia"[Mesh] OR "Rwanda"[Mesh] OR "Samoa"[Mesh] OR "Atlantic Islands"[Mesh] OR "Senegal"[Mesh] OR "Serbia"[Mesh] OR "Seychelles"[Mesh] OR "Sierra Leone"[Mesh] OR "Melanesia"[Mesh] OR "Somalia"[Mesh] OR "South Africa"[Mesh] OR "Sri Lanka"[Mesh] OR "Saint Lucia"[Mesh] OR "Saint Vincent and the Grenadines"[Mesh] OR "Sudan"[Mesh] OR "Suriname"[Mesh] OR "Swaziland"[Mesh] OR "Syria"[Mesh] OR "Tajikistan"[Mesh] OR "Tanzania"[Mesh] OR "Thailand"[Mesh] OR "East Timor"[Mesh] OR "Togo"[Mesh] OR "Tonga"[Mesh] OR "Tunisia"[Mesh] OR "Turkey"[Mesh] OR "Turkmenistan"[Mesh] OR "Micronesia"[Mesh] OR "Uganda"[Mesh] OR "Ukraine"[Mesh] OR "Uruguay"[Mesh] OR "Uzbekistan"[Mesh] OR "Vanuatu"[Mesh] OR "Venezuela"[Mesh] OR "Vietnam"[Mesh] OR "Yemen"[Mesh] OR "Zambia"[Mesh] OR "Zimbabwe"[Mesh] OR "Afghanistan"[Tiab] OR "Albania"[Tiab] OR "Algeria"[Tiab] OR "American Samoa"[Tiab] OR "Angola"[Tiab] OR "Antigua and Barbuda"[Tiab] OR "Argentina"[Tiab] OR "Armenia"[Tiab] OR “Azerbaijan”[Tiab] OR "Bangladesh"[Tiab] OR "Republic of Belarus"[Tiab] OR "Belize"[Tiab] OR "Benin"[Tiab] OR "Bhutan"[Tiab] OR "Bolivia"[Tiab] OR "Bosnia-Herzegovina"[Tiab] OR "Botswana"[Tiab] OR "Brazil"[Tiab] OR "Bulgaria"[Tiab] OR "Burkina Faso"[Tiab] OR "Burundi"[Tiab] OR "Cape Verde"[Tiab] OR "Cameroon"[Tiab] OR "Cambodia"[Tiab] OR "Central African Republic"[Tiab] OR "Chad"[Tiab] OR "China"[Tiab] OR "Colombia"[Tiab] OR "Comoros"[Tiab] OR "Congo"[Tiab] OR "Democratic Republic of the Congo"[Tiab] OR "Costa Rica"[Tiab] OR "Cote d'Ivoire"[Tiab] OR "Cuba"[Tiab] OR "Djibouti"[Tiab] OR "Dominica"[Tiab] OR "Dominican Republic"[Tiab] OR "Ecuador"[Tiab] OR "Egypt"[Tiab] OR "El Salvador"[Tiab] OR "Eritrea"[Tiab] OR "Ethiopia"[Tiab] OR "Fiji"[Tiab] OR "Gabon"[Tiab] OR "Gambia"[Tiab] OR "Georgia (Republic)"[Tiab] OR "Ghana"[Tiab] OR "Grenada"[Tiab] OR "Guatemala"[Tiab] OR "Guinea"[Tiab] OR "Equatorial Guinea"[Tiab] OR "Guinea-Bissau"[Tiab] OR "Guyana"[Tiab] OR "Haiti"[Tiab] OR "Honduras"[Tiab] OR “Hungary”[Tiab] OR "India"[Tiab] OR "Indonesia"[Tiab] OR "Iran"[Tiab] OR "Iraq"[Tiab] OR "Jamaica"[Tiab] OR "Jordan"[Tiab] OR "Kazakhstan"[Tiab] OR "Kenya"[Tiab] OR “Kosovo”[Tiab] OR "Micronesia"[Tiab] OR "Democratic People's Republic of Korea"[Tiab] OR "Yugoslavia"[Tiab] OR "Kyrgyzstan"[Tiab] OR "Laos"[Tiab] OR "Latvia"[Tiab] OR "Lebanon"[Tiab] OR "Lesotho"[Tiab] OR "Liberia"[Tiab] OR "Libya"[Tiab] OR "Macedonia"[Tiab] OR "Madagascar"[Tiab] OR "Malawi"[Tiab] OR "Malaysia"[Tiab] OR "Indian Ocean Islands"[Tiab] OR "Mali"[Tiab] OR "Mauritania"[Tiab] OR “Mauritius”[Tiab] OR "Mexico"[Tiab] OR "Moldova"[Tiab] OR "Mongolia"[Tiab] OR "Montenegro"[Tiab] OR "Morocco"[Tiab] OR "Mozambique"[Tiab] OR "Myanmar"[Tiab] OR "Namibia"[Tiab] OR "Nepal"[Tiab] OR "Nicaragua"[Tiab] OR "Niger"[Tiab] OR "Nigeria"[Tiab] OR "Pakistan"[Tiab] OR "Palau"[Tiab] OR "Panama"[Tiab] OR "Papua New Guinea"[Tiab] OR "Paraguay"[Tiab] OR "Peru"[Tiab] OR "Philippines"[Tiab] OR "Romania"[Tiab] OR "Siberia"[Tiab] OR "Rwanda"[Tiab] OR "Samoa"[Tiab] OR "Atlantic Islands"[Tiab] OR "Senegal"[Tiab] OR "Serbia"[Tiab] OR "Seychelles"[Tiab] OR "Sierra Leone"[Tiab] OR "Melanesia"[Tiab] OR "Somalia"[Tiab] OR "South Africa"[Tiab] OR "Sri Lanka"[Tiab] OR "Saint Lucia"[Tiab] OR "Saint Vincent and the Grenadines"[Tiab] OR "Sudan"[Tiab] OR "Suriname"[Tiab] OR "Swaziland"[Tiab] OR "Syria"[Tiab] OR "Tajikistan"[Tiab] OR "Tanzania"[Tiab] OR "Thailand"[Tiab] OR "East Timor"[Tiab] OR "Togo"[Tiab] OR "Tonga"[Tiab] OR "Tunisia"[Tiab] OR "Turkey"[Tiab] OR "Turkmenistan"[Tiab] OR "Micronesia"[Tiab] OR "Uganda"[Tiab] OR "Ukraine"[Tiab] OR "Uruguay"[Tiab] OR "Uzbekistan"[Tiab] OR "Vanuatu"[Tiab] OR "Venezuela"[Tiab] OR "Vietnam"[Tiab] OR "Yemen"[Tiab] OR "Zambia"[Tiab] OR "Zimbabwe"[Tiab]) OR "internationality"[MeSH] OR “World health organization”[MeSH] OR “united nations”[MeSH] OR “Africa”[MeSH] OR “low-income”[tiab]

OR “middle-income”[tiab] OR “low-resource”[tiab] or “developing country”[tiab] or “Africa”[tiab] OR "Central America"[Tiab]))

SEARCH BREAKDOWN

1 AND 2 AND 4

OR

3 AND 4

1. *Specify hospital mortality*

"Hospital Mortality"[Mesh] OR "Hospital Mortality"[tiab] OR “Hospital Death”[tiab]

2. *Delineate by surgery or anesthesia*

"Surgical Procedures, Operative"[Mesh] OR “surgery department, hospital”[MeSH] OR “General Surgery”[Mesh] OR “Anesthesia”[Mesh] OR "Cesarean Section"[Mesh] OR “surgery”[tiab] OR “surgical”[tiab] OR “anesthesia”[tiab] OR “anesthetic”[tiab] OR “anaesthesia”[tiab] OR “anaesthetic”[tiab] OR "cesarean"[tiab] or "caesarean"[tiab]

3. Delineate by surgery or anesthesia and mortality

"Intraoperative Care/mortality"[Mesh] OR ("intraoperative"[tiab] AND (“mortality”[tiab] OR “death”[tiab])) OR "Postoperative Care/mortality"[Mesh] OR "Postoperative Complications/mortality"[Mesh] OR ("postoperative"[tiab] AND (“mortality”[tiab] OR “death”[tiab])) OR "Perioperative Period/mortality"[Mesh] OR (“perioperative”[tiab] AND (“mortality”[tiab] OR “death”[tiab])) OR (“surgery”[tiab] AND (“mortality”[tiab] OR “death”[tiab])) OR (“surgical”[tiab] AND (“mortality”[tiab] OR “death”[tiab])) OR (“operative”[tiab] AND (“mortality”[tiab] OR “death”[tiab])) OR (“operation”[tiab] AND (“mortality”[tiab] OR “death”[tiab])) OR "Anesthesia/mortality"[Mesh] OR (“anesthesia”[tiab] AND (“mortality”[tiab] OR “death”[tiab])) OR (“anesthetic”[tiab] AND (“mortality”[tiab] OR “death”[tiab])) OR (“anaesthesia”[tiab] AND (“mortality”[tiab] OR “death”[tiab])) OR (“anaesthetic”[tiab] AND (“mortality”[tiab] OR “death”[tiab])) OR ((“cesarean”[tiab] OR “caesarean”[tiab]) AND (“mortality”[tiab] OR “death”[tiab]))

4. Delineate by low-resource setting

("Developing Countries"[Mesh] OR "Africa South of the Sahara"[Mesh] OR "Central America"[Mesh] OR "Afghanistan"[Mesh] OR "Albania"[Mesh] OR "Algeria"[Mesh] OR "American Samoa"[Mesh] OR "Angola"[Mesh] OR "Antigua and Barbuda"[Mesh] OR "Argentina"[Mesh] OR "Armenia"[Mesh] OR "Azerbaijan" OR "Bangladesh"[Mesh] OR "Republic of Belarus"[Mesh] OR "Belize"[Mesh] OR "Benin"[Mesh] OR "Bhutan"[Mesh] OR "Bolivia"[Mesh] OR "Bosnia-Herzegovina"[Mesh] OR "Botswana"[Mesh] OR "Brazil"[Mesh] OR "Bulgaria"[Mesh] OR "Burkina Faso"[Mesh] OR "Burundi"[Mesh] OR "Cape Verde"[Mesh] OR "Cameroon"[Mesh] OR "Cambodia"[Mesh] OR "Central African Republic"[Mesh] OR "Chad"[Mesh] OR "China"[Mesh] OR "Colombia"[Mesh] OR "Comoros"[Mesh] OR "Congo"[Mesh] OR "Democratic Republic of the Congo"[Mesh] OR "Costa Rica"[Mesh] OR "Cote d'Ivoire"[Mesh] OR "Cuba"[Mesh] OR "Djibouti"[Mesh] OR "Dominica"[Mesh] OR "Dominican Republic"[Mesh] OR "Ecuador"[Mesh] OR "Egypt"[Mesh] OR "El Salvador"[Mesh] OR "Eritrea"[Mesh] OR "Ethiopia"[Mesh] OR "Fiji"[Mesh] OR "Gabon"[Mesh] OR "Gambia"[Mesh] OR "Georgia (Republic)"[Mesh] OR "Ghana"[Mesh] OR "Grenada"[Mesh] OR "Guatemala"[Mesh] OR "Guinea"[Mesh] OR "Equatorial Guinea"[Mesh] OR "Guinea-Bissau"[Mesh] OR "Guyana"[Mesh] OR "Haiti"[Mesh] OR "Honduras"[Mesh] OR "Hungary"[Mesh] OR "India"[Mesh] OR "Indonesia"[Mesh] OR "Iran"[Mesh] OR "Iraq"[Mesh] OR "Jamaica"[Mesh] OR "Jordan"[Mesh] OR "Kazakhstan"[Mesh] OR "Kenya"[Mesh] OR "Kosovo"[Mesh] OR "Micronesia"[Mesh] OR "Democratic People's Republic of Korea"[Mesh] OR "Yugoslavia"[Mesh] OR "Kyrgyzstan"[Mesh] OR "Laos"[Mesh] OR "Latvia"[Mesh] OR "Lebanon"[Mesh] OR "Lesotho"[Mesh] OR "Liberia"[Mesh] OR "Libya"[Mesh] OR "Macedonia (Republic)"[Mesh] OR "Madagascar"[Mesh] OR "Malawi"[Mesh] OR "Malaysia"[Mesh] OR "Indian Ocean Islands"[Mesh] OR "Mali"[Mesh] OR "Mauritania"[Mesh] OR "Mauritius"[Mesh] OR "Mexico"[Mesh] OR "Moldova"[Mesh] OR "Mongolia"[Mesh] OR "Montenegro"[Mesh] OR "Morocco"[Mesh] OR "Mozambique"[Mesh] OR "Myanmar"[Mesh] OR "Namibia"[Mesh] OR "Nepal"[Mesh] OR "Nicaragua"[Mesh] OR "Niger"[Mesh] OR "Nigeria"[Mesh] OR "Pakistan"[Mesh] OR "Palau"[Mesh] OR "Panama"[Mesh] OR "Papua New Guinea"[Mesh] OR "Paraguay"[Mesh] OR "Peru"[Mesh] OR "Philippines"[Mesh] OR "Romania"[Mesh] OR "Siberia"[Mesh] OR "Rwanda"[Mesh] OR "Samoa"[Mesh] OR "Atlantic Islands"[Mesh] OR "Senegal"[Mesh] OR "Serbia"[Mesh] OR "Seychelles"[Mesh] OR "Sierra Leone"[Mesh] OR "Melanesia"[Mesh] OR "Somalia"[Mesh] OR "South Africa"[Mesh] OR "Sri Lanka"[Mesh] OR "Saint Lucia"[Mesh] OR "Saint Vincent and the Grenadines"[Mesh] OR "Sudan"[Mesh] OR "Suriname"[Mesh] OR "Swaziland"[Mesh] OR "Syria"[Mesh] OR "Tajikistan"[Mesh] OR "Tanzania"[Mesh] OR "Thailand"[Mesh] OR "East Timor"[Mesh] OR "Togo"[Mesh] OR "Tonga"[Mesh] OR "Tunisia"[Mesh] OR "Turkey"[Mesh] OR "Turkmenistan"[Mesh] OR "Micronesia"[Mesh] OR "Uganda"[Mesh] OR "Ukraine"[Mesh] OR "Uruguay"[Mesh] OR "Uzbekistan"[Mesh] OR "Vanuatu"[Mesh] OR "Venezuela"[Mesh] OR "Vietnam"[Mesh] OR "Yemen"[Mesh] OR "Zambia"[Mesh] OR "Zimbabwe"[Mesh]) OR ("internationality"[MeSH Terms] OR World health organization[MeSH Terms] OR united nations[MeSH Terms] OR africa[MeSH Terms]) OR ("low-income"[tiab] OR "middle-income"[tiab] OR "low-resource"[tiab] or "developing country"[tiab] or "Africa"[tiab]) OR ("Central America"[Tiab] OR "Afghanistan"[Tiab] OR "Albania"[Tiab] OR "Algeria"[Tiab] OR "American Samoa"[Tiab] OR "Angola"[Tiab] OR "Antigua and Barbuda"[Tiab] OR "Argentina"[Tiab] OR "Armenia"[Tiab] OR "Azerbaijan"[Tiab] OR "Bangladesh"[Tiab] OR "Republic of Belarus"[Tiab] OR "Belize"[Tiab] OR "Benin"[Tiab] OR "Bhutan"[Tiab] OR "Bolivia"[Tiab] OR "Bosnia-Herzegovina"[Tiab] OR "Botswana"[Tiab] OR "Brazil"[Tiab] OR "Bulgaria"[Tiab] OR "Burkina Faso"[Tiab] OR "Burundi"[Tiab] OR "Cape Verde"[Tiab] OR "Cameroon"[Tiab] OR "Cambodia"[Tiab] OR "Central

African Republic"[Tiab] OR "Chad"[Tiab] OR "Chile"[Tiab] OR "China"[Tiab] OR "Colombia"[Tiab] OR "Comoros"[Tiab] OR "Congo"[Tiab] OR "Democratic Republic of the Congo"[Tiab] OR "Costa Rica"[Tiab] OR "Cote d'Ivoire"[Tiab] OR "Cuba"[Tiab] OR "Djibouti"[Tiab] OR "Dominica"[Tiab] OR "Dominican Republic"[Tiab] OR "Ecuador"[Tiab] OR "Egypt"[Tiab] OR "El Salvador"[Tiab] OR "Eritrea"[Tiab] OR "Ethiopia"[Tiab] OR "Fiji"[Tiab] OR "Gabon"[Tiab] OR "Gambia"[Tiab] OR "Georgia (Republic)"[Tiab] OR "Ghana"[Tiab] OR "Grenada"[Tiab] OR "Guatemala"[Tiab] OR "Guinea"[Tiab] OR "Equatorial Guinea"[Tiab] OR "Guinea-Bissau"[Tiab] OR "Guyana"[Tiab] OR "Haiti"[Tiab] OR "Honduras"[Tiab] OR “Hungary”[Tiab] OR "India"[Tiab] OR "Indonesia"[Tiab] OR "Iran"[Tiab] OR "Iraq"[Tiab] OR "Jamaica"[Tiab] OR "Jordan"[Tiab] OR "Kazakhstan"[Tiab] OR "Kenya"[Tiab] OR “Kosovo”[Tiab] OR "Micronesia"[Tiab] OR "Democratic People's Republic of Korea"[Tiab] OR "Yugoslavia"[Tiab] OR "Kyrgyzstan"[Tiab] OR "Laos"[Tiab] OR "Latvia"[Tiab] OR "Lebanon"[Tiab] OR "Lesotho"[Tiab] OR "Liberia"[Tiab] OR "Libya"[Tiab] OR "Lithuania"[Tiab] OR "Macedonia"[Tiab] OR "Madagascar"[Tiab] OR "Malawi"[Tiab] OR "Malaysia"[Tiab] OR "Indian Ocean Islands"[Tiab] OR "Mali"[Tiab] OR "Mauritania"[Tiab] OR “Mauritius”[Tiab] OR "Mexico"[Tiab] OR "Moldova"[Tiab] OR "Mongolia"[Tiab] OR "Montenegro"[Tiab] OR "Morocco"[Tiab] OR "Mozambique"[Tiab] OR "Myanmar"[Tiab] OR "Namibia"[Tiab] OR "Nepal"[Tiab] OR "Nicaragua"[Tiab] OR "Niger"[Tiab] OR "Nigeria"[Tiab] OR "Pakistan"[Tiab] OR "Palau"[Tiab] OR "Panama"[Tiab] OR "Papua New Guinea"[Tiab] OR "Paraguay"[Tiab] OR "Peru"[Tiab] OR "Philippines"[Tiab] OR "Romania"[Tiab] OR "Siberia"[Tiab] OR "Rwanda"[Tiab] OR "Samoa"[Tiab] OR "Atlantic Islands"[Tiab] OR "Senegal"[Tiab] OR "Serbia"[Tiab] OR "Seychelles"[Tiab] OR "Sierra Leone"[Tiab] OR "Melanesia"[Tiab] OR "Somalia"[Tiab] OR "South Africa"[Tiab] OR "Sri Lanka"[Tiab] OR "Saint Lucia"[Tiab] OR "Saint Vincent and the Grenadines"[Tiab] OR "Sudan"[Tiab] OR "Suriname"[Tiab] OR "Swaziland"[Tiab] OR "Syria"[Tiab] OR "Tajikistan"[Tiab] OR "Tanzania"[Tiab] OR "Thailand"[Tiab] OR "East Timor"[Tiab] OR "Togo"[Tiab] OR "Tonga"[Tiab] OR "Tunisia"[Tiab] OR "Turkey"[Tiab] OR "Turkmenistan"[Tiab] OR "Micronesia"[Tiab] OR "Uganda"[Tiab] OR "Ukraine"[Tiab] OR "Uruguay"[Tiab] OR "Uzbekistan"[Tiab] OR "Vanuatu"[Tiab] OR "Venezuela"[Tiab] OR "Vietnam"[Tiab] OR "Yemen"[Tiab] OR "Zambia"[Tiab] OR "Zimbabwe"[Tiab])

FILTERS

Publication Dates: 1/1/2009 to 12/31/2014

Languages: English

APPENDIX 2: EMBASE SEARCH STRATEGY

EMBASE QUERY

1. Delineate by surgical, obstetric, or anesthetic care:

Surgery/exp OR ‘surgical ward’/exp OR ‘general surgery’/exp OR ‘anesthesia’/exp OR ‘cesarean section’/exp OR ‘obstetric operation’/exp OR ‘intraoperative period’/exp OR ‘postoperative period’/exp OR ‘perioperative period’/exp OR ‘surgery’:ab,ti OR ‘operative’:ab,ti OR ‘surgical’:ab,ti OR ‘anesthesia’:ab,ti OR ‘anaesthesia’:ab,ti OR ‘anesthetic’:ab,ti OR ‘anaesthetic’:ab,ti OR ‘cesarean’:ab,ti OR ‘caesarean’:ab,ti OR ‘intraoperative’:ab,ti OR ‘postoperative’:ab,ti OR ‘perioperative’:ab,ti

2. Delineate by mortality

‘Surgical mortality’/exp OR ‘mortality’/exp OR ‘death’/exp OR ‘mortality’:ab,ti OR ‘death’:ab,ti

3. Delineate by developing country

'developing country'/exp OR ‘africa south of the sahara’/exp OR ‘central america’/exp OR ‘international cooperation’/exp OR ‘world health organization’/exp OR ‘developing country’:ab,ti OR ‘central america’:ab,ti OR ‘international cooperation’:ab,ti OR ‘world health organization’:ab,ti OR ‘low-income’:ab,ti OR ‘middle-income’:ab,ti OR ‘low-resource’:ab,ti

OR

‘Afghanistan’/exp OR ‘Albania’/exp OR ‘Algeria’/exp OR ‘American Samoa’/exp OR ‘Angola’/exp OR ’Antigua and Barbuda’/exp OR ‘Argentina’/exp OR ‘Armenia’/exp OR ‘Azerbaijan’/exp OR ‘Bangladesh’/exp OR ‘Belarus’/exp OR ‘Belize’/exp OR ‘Benin’/exp OR ‘Bhutan’/exp OR ‘Bolivia’/exp OR ‘Bosnia and Herzegovina’/exp OR ‘Botswana’/exp OR ‘Brazil’/exp OR ‘Bulgaria’/exp OR ‘Burkina Faso’/exp OR ‘Burundi’/exp OR ‘Cape Verde’/exp OR ‘Cameroon’/exp OR ‘Cambodia’/exp OR ‘Central African Republic’/exp OR ‘Chad’/exp OR ‘China’/exp OR ‘Colombia’/exp OR ‘Comoros’/exp OR ‘Congo’/exp OR ‘Democratic Republic Congo’/exp OR ‘Costa Rica’/exp OR ‘Cote d Ivoire’/exp OR ‘Cuba’/exp OR ‘Djibouti’/exp OR ‘Dominica’/exp OR ‘Dominican Republic’/exp OR ‘Ecuador’/exp OR ‘Egypt’/exp OR ‘El Salvador’/exp OR ‘Eritrea’/exp OR ‘Ethiopia’/exp OR ‘Fiji’/exp OR ‘Gabon’/exp OR ‘Gambia’/exp OR ‘Georgia (republic)’/exp OR ‘Ghana’/exp OR ‘Grenada’/exp OR ‘Guatemala’/exp OR ‘Guinea’/exp OR ‘Equatorial Guinea’/exp OR ‘Guinea-Bissau’/exp OR ‘Guyana’/exp OR ‘Haiti’/exp OR ‘Honduras’/exp OR ‘Hungary’/exp OR ‘India’/exp OR ‘Indonesia’/exp OR ‘Iran’/exp OR ‘Iraq’/exp OR ‘Jamaica’/exp OR ‘Jordan’/exp OR ‘Kazakhstan’/exp OR ‘Kenya’/exp OR ‘Federated States of Micronesia’/exp OR ‘North Korea’/exp OR ‘Yugoslavia’/exp OR ‘Kiribati’/exp OR ‘Kosovo’/exp OR ‘Kyrgyzstan’/exp OR ‘Laos’/exp OR ‘Latvia’/exp OR ‘Lebanon’/exp OR ‘Lesotho’/exp OR ‘Liberia’/exp OR ‘Libyan Arab Jamahiriya’/exp OR ‘Macedonia (republic)’/exp OR ‘Madagascar’/exp OR ‘Malawi’/exp OR ‘Malaysia’/exp OR ‘Indian Ocean’/exp OR ‘Mali’/exp OR ‘Marshall Islands’/exp OR ‘Mauritania’/exp OR ‘Mauritius’/exp OR ‘Mexico’/exp OR ‘Moldova’/exp OR ‘Mongolia’/exp OR ‘Montenegro (republic)’/exp OR ‘Morocco’/exp OR ‘Mozambique’/exp OR ‘Myanmar’/exp OR ‘Namibia’/exp OR ‘Nepal’/exp OR ‘Nicaragua’/exp OR ‘Niger’/exp OR ‘Nigeria’/exp OR ‘Pakistan’/exp OR ‘Palau’/exp OR ‘Panama’/exp OR ‘Papua New Guinea’/exp OR ‘Paraguay’/exp OR ‘Peru’/exp OR ‘Philippines’/exp OR ‘Romania’/exp OR ‘Rwanda’/exp OR ‘Samoa’/exp OR ‘Atlantic Islands’/exp OR ‘Senegal’/exp OR ‘Serbia’/exp OR ‘Seychelles’/exp OR ‘Sierra Leone’/exp OR ‘Melanesia’/exp OR ‘Somalia’/exp OR ‘Solomon Islands’/exp OR ‘South Africa’/exp OR ‘Sri Lanka’/exp OR ‘Saint Lucia’/exp OR ‘Saint Vincent and the Grenadines’/exp OR ‘Sudan’/exp OR ‘Suriname’/exp OR ‘Swaziland’/exp OR ‘Syrian Arab Republic’/exp OR ‘Tajikistan’/exp OR ‘Tanzania’/exp OR ‘Thailand’/exp OR ‘Timor-Leste’/exp OR ‘Togo’/exp OR ‘Tonga’/exp OR ‘Tunisia’/exp OR ‘Turkey (republic)’/exp OR ‘Turkmenistan’/exp OR ‘Uganda’/exp OR ‘Ukraine’/exp OR ‘Uruguay’/exp OR ‘Uzbekistan’/exp OR ‘Vanuatu’/exp OR ‘Venezuela’/exp OR ‘Viet Nam’/exp OR ‘Yemen’/exp OR ‘Zambia’/exp OR ‘Zimbabwe’/exp OR ‘Afghanistan’:ab,ti OR ‘Albania’:ab,ti OR ‘Algeria’:ab,ti OR ‘American Samoa’:ab,ti OR ‘Angola’:ab,ti OR ’Antigua and Barbuda’:ab,ti OR ‘Argentina’:ab,ti OR

‘Armenia’:ab,ti OR ‘Azerbaijan’:ab,ti OR ‘Bangladesh’:ab,ti OR ‘Belarus’:ab,ti OR ‘Belize’:ab,ti OR ‘Benin’:ab,ti OR ‘Bhutan’:ab,ti OR ‘Bolivia’:ab,ti OR ‘Bosnia and Herzegovina’:ab,ti OR ‘Botswana’:ab,ti OR ‘Brazil’:ab,ti OR ‘Bulgaria’:ab,ti OR ‘Burkina Faso’:ab,ti OR ‘Burundi’:ab,ti OR ‘Cape Verde’:ab,ti OR ‘Cameroon’:ab,ti OR ‘Cambodia’:ab,ti OR ‘Central African Republic’:ab,ti OR ‘Chad’:ab,ti OR ‘China’:ab,ti OR ‘Colombia’:ab,ti OR ‘Comoros’:ab,ti OR ‘Congo’:ab,ti OR ‘Democratic Republic Congo’:ab,ti OR ‘Costa Rica’:ab,ti OR ‘Cote d Ivoire’:ab,ti OR ‘Cuba’:ab,ti OR ‘Djibouti’:ab,ti OR ‘Dominica’:ab,ti OR ‘Dominican Republic’:ab,ti OR ‘Ecuador’:ab,ti OR ‘Egypt’:ab,ti OR ‘El Salvador’:ab,ti OR ‘Eritrea’:ab,ti OR ‘Ethiopia’:ab,ti OR ‘Fiji’:ab,ti OR ‘Gabon’:ab,ti OR ‘Gambia’:ab,ti OR ‘Georgia (republic)’:ab,ti OR ‘Ghana’:ab,ti OR ‘Grenada’:ab,ti OR ‘Guatemala’:ab,ti OR ‘Guinea’:ab,ti OR ‘Equatorial Guinea’:ab,ti OR ‘Guinea-Bissau’:ab,ti OR ‘Guyana’:ab,ti OR ‘Haiti’:ab,ti OR ‘Honduras’:ab,ti OR ‘Hungary’:ab,ti OR ‘India’:ab,ti OR ‘Indonesia’:ab,ti OR ‘Iran’:ab,ti OR ‘Iraq’:ab,ti OR ‘Jamaica’:ab,ti OR ‘Jordan’:ab,ti OR ‘Kazakhstan’:ab,ti OR ‘Kenya’:ab,ti OR ‘Federated States of Micronesia’:ab,ti OR ‘North Korea’:ab,ti OR ‘Yugoslavia’:ab,ti OR ‘Kiribati’:ab,ti OR ‘Kosovo’:ab,ti OR ‘Kyrgyzstan’:ab,ti OR ‘Laos’:ab,ti OR ‘Latvia’:ab,ti OR ‘Lebanon’:ab,ti OR ‘Lesotho’:ab,ti OR ‘Liberia’:ab,ti OR ‘Libyan Arab Jamahiriya’:ab,ti OR ‘Macedonia (republic)’:ab,ti OR ‘Madagascar’:ab,ti OR ‘Malawi’:ab,ti OR ‘Malaysia’:ab,ti OR ‘Indian Ocean’:ab,ti OR ‘Mali’:ab,ti OR ‘Marshall Islands’:ab,ti OR ‘Mauritania’:ab,ti OR ‘Mauritius’:ab,ti OR ‘Mexico’:ab,ti OR ‘Moldova’:ab,ti OR ‘Mongolia’:ab,ti OR ‘Montenegro (republic)’:ab,ti OR ‘Morocco’:ab,ti OR ‘Mozambique’:ab,ti OR ‘Myanmar’:ab,ti OR ‘Namibia’:ab,ti OR ‘Nepal’:ab,ti OR ‘Nicaragua’:ab,ti OR ‘Niger’:ab,ti OR ‘Nigeria’:ab,ti OR ‘Pakistan’:ab,ti OR ‘Palau’:ab,ti OR ‘Panama’:ab,ti OR ‘Papua New Guinea’:ab,ti OR ‘Paraguay’:ab,ti OR ‘Peru’:ab,ti OR ‘Philippines’:ab,ti OR ‘Romania’:ab,ti OR ‘Rwanda’:ab,ti OR ‘Samoa’:ab,ti OR ‘Atlantic Islands’:ab,ti OR ‘Senegal’:ab,ti OR ‘Serbia’:ab,ti OR ‘Seychelles’:ab,ti OR ‘Sierra Leone’:ab,ti OR ‘Melanesia’:ab,ti OR ‘Somalia’:ab,ti OR ‘Solomon Islands’:ab,ti OR ‘South Africa’:ab,ti OR ‘Sri Lanka’:ab,ti OR ‘Saint Lucia’:ab,ti OR ‘Saint Vincent and the Grenadines’:ab,ti OR ‘Sudan’:ab,ti OR ‘Suriname’:ab,ti OR ‘Swaziland’:ab,ti OR ‘Syrian Arab Republic’:ab,ti OR ‘Tajikistan’:ab,ti OR ‘Tanzania’:ab,ti OR ‘Thailand’:ab,ti OR ‘Timor-Leste’:ab,ti OR ‘Togo’:ab,ti OR ‘Tonga’:ab,ti OR ‘Tunisia’:ab,ti OR ‘Turkey (republic)’:ab,ti OR ‘Turkmenistan’:ab,ti OR ‘Uganda’:ab,ti OR ‘Ukraine’:ab,ti OR ‘Uruguay’:ab,ti OR ‘Uzbekistan’:ab,ti OR ‘Vanuatu’:ab,ti OR ‘Venezuela’:ab,ti OR ‘Viet Nam’:ab,ti OR ‘Yemen’:ab,ti OR ‘Zambia’:ab,ti OR ‘Zimbabwe’:ab,ti

AND [english]/lim

AND [embase]/lim

AND [2009-2015]/py

AND ([article]/lim OR [article in press]/lim)

APPENDIX 3: WEB OF SCIENCE SEARCH STRATEGY

1. Delineate by surgery or anesthesia (Topic)

Operation OR Surgery OR Surgical OR Caesarean OR Cesarean OR Anesthesia OR Anaesthesia OR Perioperative OR Postoperative OR Intraoperative OR peri-operative OR post-operative OR intra-operative

2. Delineate by mortality (Topic)

Death OR Deaths OR Mortality OR Mortal*

3. Delineate by low-resource setting (Topic)

"Internationality" OR "World Health Organization" OR "United Nations" OR Africa OR Subsaharan OR Sub-saharan OR "low-income" OR "middle-income" OR "low-resource" or "developing country"

OR

Afghanistan OR Albania OR Algeria OR Angola OR Antigua OR Barbuda OR Argentina OR Armenia OR Azerbaijan OR Bangladesh OR Belarus OR Belize OR Benin OR Bhutan OR Bolivia OR Bosnia OR Herzegovina OR Botswana OR Brazil OR Bulgaria OR "Burkina Faso" OR Burundi OR "Cape Verde" OR "Cabo Verde" OR Cameroon OR Cambodia OR "Central African Republic" OR Chad OR Chile OR China OR Colombia OR Comoros OR Congo OR "Democratic Republic of the Congo" OR "DRC" OR "Costa Rica" OR "Cote d'Ivoire" OR "Ivory Coast" OR Cuba OR Djibouti OR Dominica OR "Dominican Republic" OR Ecuador OR Egypt OR "El Salvador" OR Eritrea OR Ethiopia OR Fiji OR Gabon OR Gambia OR Gaza OR Georgia Republic OR Ghana OR Grenada OR Guatemala OR Guinea OR "Equatorial Guinea" OR "Guinea-Bissau" OR Guyana OR Haiti OR Honduras OR Hungary OR India OR Indonesia OR Iran OR Iraq OR Jamaica OR Jordan OR Kazakhstan OR Kenya OR Kiribati OR Kosovo OR Micronesia OR "Democratic People's Republic of Korea" OR "DPR" OR "DPRK" OR Yugoslavia OR Kyrgyzstan OR "Kyrgyz Republic" OR Laos OR Latvia OR Lebanon OR Lesotho OR Liberia OR Libya OR Lithuania OR Macedonia OR Madagascar OR Malawi OR Malaysia OR Maldives OR Indian Ocean Islands OR Mali OR Marshall Islands OR Mauritania OR Mauritius OR Mexico OR Moldova OR Mongolia OR Montenegro OR Morocco OR Mozambique OR Myanmar OR Namibia OR Nepal OR Nicaragua OR Niger OR Nigeria OR Pakistan OR Palau OR Panama OR "Papua New Guinea" OR Paraguay OR Peru OR Philippines OR Romania OR Siberia OR "Sao tome" OR Principe OR Rwanda OR Samoa OR "Atlantic Islands" OR Senegal OR Serbia OR Seychelles OR "Sierra Leone" OR Melanesia OR Somalia OR "Solomon Islands" OR "South Africa" OR "Sri Lanka" OR "Saint Lucia" OR "St Lucia" OR "Saint Vincent" OR "St Vincent" OR "The Grenadines" OR Sudan OR Suriname OR Swaziland OR Syria OR Tajikistan OR Tanzania OR Thailand OR "East Timor" OR Togo OR Tonga OR Tunisia OR Turkey OR Turkmenistan OR Tuvalu OR Micronesia OR Uganda OR Ukraine OR Uruguay OR Uzbekistan OR Vanuatu OR Venezuela OR Vietnam OR "West Bank" OR Yemen OR Zambia OR Zimbabwe

Publication Dates: 2009 to 2014

Languages: English

Publication Type: Article

Collections:

Science Citation Index Expanded (SCI-EXPANDED) --1900-present

Social Sciences Citation Index (SSCI) --1900-present

Arts & Humanities Citation Index (A&HCI) --1975-present

APPENDIX 4: LILACS SEARCH STRATEGY

1. Delineate by surgery or anesthesia (Words)

Operation OR Surgery OR Surgical OR Caesarean OR Cesarean OR Anesthesia OR Anaesthesia OR Perioperative OR Postoperative OR Intraoperative OR peri-operative OR post-operative OR intra-operative

2. Delineate by mortality (Words)

Death OR Deaths OR Mortality OR Mortalities

3. Delineate by year of publication (Country, Year of Publication)

AND

2009 OR 2010 OR 2011 OR 2012 OR 2013 OR 2014

4. Delineate by English (Language)

English

APPENDIX 5: AIM SEARCH STRATEGY

1. Delineate by surgery or anesthesia (Key Words)

Operation OR Surgery OR Surgical OR Caesarean OR Cesarean OR Anesthesia OR Anaesthesia OR Perioperative OR Postoperative OR Intraoperative OR peri-operative OR post-operative OR intra-operative

2. Delineate by mortality (Key Words)

Death OR Deaths OR Mortality OR Mortalities

3. Delineate by year of publication (Year of Publication)

AND

2009 OR 2010 OR 2011 OR 2012 OR 2013 OR 2014

APPENDIX 6: WHOLIS SEARCH STRATEGY

1. Delineate by surgery or anesthesia (Key Words)

Operation OR Surgery OR Surgical OR Caesarean OR Cesarean OR Anesthesia OR Anaesthesia OR Perioperative OR Postoperative OR Intraoperative

2. Delineate by mortality (Key Words)

Death OR Deaths OR Mortality OR Mortalities
