# Supplementary material for: Perioperative mortality rates in low-income and middle-income countries: a systematic review and meta-analysis
Source: BMJ Glob Health. 2018 Jun 22;3(3):e000810. doi: 10.1136/bmjgh-2018-000810 (PMC6035511; doi:10.1136/bmjgh-2018-000810)
Supplement: Supplementary file 2 [file bmjgh-2018-000810supp002.pdf]

variable_code	Variable	Description	Values	
reviewid	Review ID	Unique ID assigned to each included/excluded paper after reading	XYYZZZ- X- "1" included. "2"excluded. YY-reviewer number. ZZZ- unique paper number	
parent_daughter	Parent or Daughter	For studies with mortality data stratified on country or urgency. Parent are rows cont	"P"- parent, "D"- daughter [urgency-stratified]; "P C"-parent, "D C"-daughter [country-stratified]	
pmid	PMID	PubMed Manuscript ID	Unique ID as string	
first_author	First Author	First author listed on paper	Lastname, I. (first initial)	
corresp_author_email	Contact email	Contact email listed in paper	String	
art_title	Article Title	Title of article	String	
paper_purpose	Purpose of Paper	Ultimate goal of paper	String	
year_published	Year Published	Year of publication	YYYY	
study_time	Retrospective or prospective	Type of study undertaken	"P" (prospective) or "R" (retrospective), or "Mixed R/P"(ambispective)	
study_design	type of study	Type of study undertaken	audit, nonrandomized cohort, case-control, RCT	
exposure	exposure Y/N	Were there two or more groups being compared?	Y/N	
exposure_type	exposure (type)	If so, what was the dependent variable?	String	
dates_assumed	Were dates assumed?	If the study did not provide clear dates during which patient data were collect, we as:	Y/N with description	
study_start	Study Start (date/year)	Start date of study	Enter as mm/dd/yyyy or mm/yyyy or yyyy, excel formatted as mmm/yy	
study_end	Study End (date/year)	End date of study	Enter as mm/dd/yyyy or mm/yyyy or yyyy, excel formatted as mmm/yy	
study_mid_yr	Study midpoint year	Year at the midpoint of study data collection	Year, as YYYY	
country	Country of Focus	Country	String. If multiple, record all countries	
country_code	Alpha-3 country code	Alpha-3 country code	String. As AAA	
country_list	List if multiple	List of countries if multiple countries are included in study base		
hss_midpoint	Health systems spending	Health spending per capita, current US$, at year of study midpoint	Numeric value	
hss_imputed	Imputed from nearest available year?		Y/N	If between two years, take most recent
gdp_midpoint	GDP per capita, current US$, at year of study midpoint	GDP per capita, current US$, at year of study midpoint	Numeric value	
gdp_imputed	Imputed from nearest available year?		Y/N	If between two years, take most recent
popdens_midpoint	Population density, people/km2, at year of study midpoint	Population density at year of study midpoint	Numeric value	
popdens_imputed	Imputed from nearest available year?		Y/N	If between two years, take most recent
hdi_midpoint	UN Human Development Index, at year of study midpoint	HDI at year of study midpoint	Numeric value	
hdi_imputed	Imputed from nearest available year?		Y/N	If between two years, take most recent
number_facilities	Number of Facilities Included	Number	Numeric value	
facility_type	Type of Facilities Included	Any descriptor of hospital type	String. 5 values "MSF"(Medecins Sans Frontieres), "CH" (Community/local Hospital), "DH" (District Hospital), "AH" (Academic/teaching Hospital), . If not described, write NA.	
rural_urban	Facilities Urban/Rural/Mix/NA	Hospital location (rural versus urban)	String. 4 potential values "U" (Urban), "R" (Rural), "M" (Mix), "NA" (unknown)	
patient_population	Patient Population Studied	Type of surgical patients included. Describe inclusion/exclusion	String	
consecutive	Consecutive patients?	Was an effort made to include all consecutive patients meeting inclusion criteria	Y/N	
specialty	Specialty specific study? Y/N	Whether study focused on a specific specialty	Y/N	
specialty_name	Name of specialty	Name of surgical specialty, by 5-letter code.	See Sheet 3	
procedure_dx_name	Name of procedure	Name of procedure being studied, or diagnostic group	See Sheet 2	N.B. When assessing mortality by procedure, remember Beard paper
hrp	Specific HRP (other than age or urgency)?	Compared to other papers with the same procedure code, does this paper have a mo	Y/N	
spec_age	Specific age stratum (NA, N, P, G)	Do authors have an age criterion for inclusion that limits their patient population to n	N (neonates), P(pediatric), NA (general/adult), G(geriatric)	
outpatient	Outpatient procedures included? Y/N	Whether or not outpatient procedures were included in study	Y/N	
anesthesia	Type of anesthesia reported? Y/N	Whether or not type of anesthesia is reported. STRICT (whether or not can assume fr	Y/N	
local_anesthesia	Procedures done under local anesthesia included? Y/N/NA	Whether or not procedures done under local anesthesia were included	Y/N/NA	
regional_anesthesia	Procedures done under regional anesthesia included? Y/N/NA	Whether or not procedures done under regional anesthesia were included	Y/N/NA	
general_anesthesia	Procedures done under general anesthesia included? Y/N/NA	Whether or not procedures done under general anesthesia were included	Y/N/NA	
procedure_location	Procedures done outside of operating room included? Y/N	Whether or not procedures performed in a procedure room or outside of the operati	Y/N/NA	
clear_definition	Was a clear definition of POMR provided? Y/N	Was the POMR definition clearly elaborated?	Y/N	
pomr_defintion	Definition of POMR Used	Descriptor of POMR definition used in the study	String	
time_pomr	Timeframe of POMR	What time of POMR was used (inpatient, 30-day, 5-day, 24h). If not clear, assume inpa	String. Consider "OT" (on-table, operating theatre), "24H", "5D","7D", "IP" (inpatient), "30D", "30_IP" (mortalities within 30 days OR during the same hospitalization), "IP_30D" (mortalities in hospital but within 30days of OR), "IP_1Y" (Mortalities in hospital but within 1Y), AD (after discharge). N.B. often, IP mortality was inferred from methods or discussion without a clear definition provided. This was through phrases such as "information obtained from discharge summaries", discussion of the circumstances of specific deaths, or discussion of survival to discharge, etc. If no mortality, and extensive follow-up, impute 30D time.	
multi_time		Was mortality at more than one time point provided? (e.g. IP and 30D)	Y/N	
pomr_pt_or_pro	POMR based on #patients or #procedures	Whether the POMR calculated was based on the number of patients or number of pr	String. "PRO" (procedures), "PTS" (patients), "ADM" (admissions)	
numerator	Numerator	Description of how deaths were enumerated	String	
denominator	Denominator	Descriptor of denominator	String	
complications	Were other complication rates reported? (Y/N)	Whether or not rates of other complications were reported	Y/N	
hiv_status	Was HIV prevalence in patient population reported?	Whether or not HIV prevalence was reported	Y/N	
hiv_adjustment	Was HIV adjusted for?	Were mortality results adjusted for, statistically related to, or stratified on HIV status?		
urgency	Was case urgency described?	Whether or not proportion of planned/emergent cases were reported	Y/N- "Y" if study restricted to "EME", "PLA", or if "STRAT" on urgency. "MIX" studies may report proportions of planned/emergent (may be "Y) but do not provide stratified mortality.	
urgency_adjustment	Was case urgency adjusted for?	Were mortality results adjusted for, statistically related to, or stratified on case urgen	Y/N- "Y" if study restricted to "EME", "PLA", or if "STRAT" on urgency. "MIX" studies may adjust mortality for urgency through clinical scores, etc (may be "Y") but do not provide stratified mortality.	
planned_eme	Planned versus emergent procedures	Were the patients being assessed planned or emergent cases, or a mix of the two? Se	PLA= planned, EME=emergency, MIX=mix (not stratified), STRAT (mortality strat	**A planned surgery is one in which the patient is admitted from his or her place of usual residence at a pre-set date for the purpose of undergoing a surgical procedure. An emergency surgery is one in which the patient undergoes a surgical procedure after being admitted to the hospital on an unpredicted date with a potentially life- or limb-threatening disease process.**
comorbidities	Were preoperative comorbidities described?	Whether or not rates of preoperative comorbidities were discussed/reported	Y/N	
comorb_adjustment	Were comorbidities adjusted for?	Was mortality adjusted for, statistically related to, or stratified on, preoperative comc	Y/N	
clin_score	Whether or not clinical scoring system was used Y/N	If a preoperative clinical scoring system was used to describe severity of illness or con	Y/N	
score_adjustment	Were clinical scores used to adjust mortality results?	Was mortality adjusted for, statistically related to, or stratified on, a clinical score?	Y/N	
score_name	If yes, which score?	Name of Score	See Sheet2	
asa_report	Was ASA score of patients reported?	Whether or not presenting ASA was reported	Y/N	
asa_adjustment	ASA Adjusted? (Y/N)	Whether or not the paper provided ASA adjustment. Write "Y" if ASA-specific mortali	Y/N	
age_report	Were patient ages reported?	Whether or not mean age/distribution of ages of population was reported	Y/N	
age_adjustment	Age Adjustment (Y/N)	Whether or not age adjustment was provided. Write "Y" if age adjusted for by stratifi	Y/N	
other_risk_adjustments	Note Additional Risk Adjustments	Description of other risk adjustments	String	
data_collection_method	How was Data Collected ?	Description of data collection method	String	
data_problem_description	Problems discussed with collection of data	Any descriptor of problems with data collection	String	
pomr_result	POMR Results	Final result reported. Type in numerator/denominator=mortality (%). For validation/\	String.	
pomr_numerator	Numerator used in calculating POMR			
pomr_denominator	Denominator used in calculating POMR			
total_mortality	Overall POMR result- imputed by paper reviewer (verified *2)	If only stratified results reported in paper, calculate weighted sum of POMR for all grc	Numeric value	
planned_mort	POMR stratum for elective cases	If elective and emergency strata provided, give POMR result for elective cases	Numeric value	
eme_mort	POMR stratum for emergency cases	If elective and emergency strata provided, give POMR result for emergency cases	Numeric value	
mortality_causes	Causes of Mortality	Causes of mortality reported	String	
benchmarking_used	Was any benchmarking used? (Y/N)	Whether or not authors compared results to other studies	Y/N	
benchmarking_description	Benchmarking methods and results	Description of benchmarking	String	
benefits_pomr	BenefitsPertaining to Use	What good came of using POMR in this case?	String	
data_completeness	Data completeness	Description of data completeness/missing data	String	
percent_missing_data	Missing data, as expressed in percentage	Percentage of data incomplete for any reason (loss to follow-up, etc)	Numeric value, expressed as decimal (e.g. 10% is 0.10)	
validity	Validity/Reliability	Face validity of study, as determined by physician coders	Y/N	
correlation_prediction	Correlation/Prediction	Reality check on study validity- whether or not risk strata correlated with mortality, e	String	
further_notes	Any additional Notes	Further comments on study from physician coders	String	
Coder	coder	Clinical reviewer who read the article.	String	

Numeric code	Text code	Descriptor of code	Number of Studies
1025	CAES	Caesarean section	55
1024	CABG	Coronary artery bypass graft	49
1061	EPH	Emergency peripartum hysterectomy	39
1184	VALVE	Cardiac valve procedures	35
1026	CARD	Cardiac surgery, not otherwise specified	31
1043	COLRES	Colon resection, excluding resection for volvulus	27
1010	APPY	Appendicitis	23
1106	LUNGRES	Pulmonary resection, excluding resection for tuberculosis	23
1141	PERF	Perforated hollow viscus, excluding perforations secondary to salmonella	22
1103	LIVRES	Hepatic resection	20
1115	MULTI	Multi-specialty patient population, usually institution-level surgical mortal	19
1138	PCARD	Pediatric cardiac procedures, excluding complex congenital heart disease	19
1158	RIM	Resection of intracranial mass	19
1075	GASTCA	Gastric cancer	18
1094	INGHERN	Inguinal hernia	17
1098	LAPAR	Laparotomy, but not meeting other abdominal surgery codes. Includes lap	17
1133	PAED	Pediatric surgical procedures, not otherwise specified	17
1036	CHOLE	Cholecystectomy	15
1183	UTRUP	Uterine rupture	15
1063	ESOCA	Esophageal carcinoma	13
1030	CCHD	Complex congenital heart disease	12
1019	BOBS	Bowel obstruction	10
1091	ICH	Intracranial hemorrhage	10
1104	LIVTRAUM	Hepatic trauma	10
1191	WHIP	Whipple pancreaticoduodenectomy	10
1113	MIS	Minimally invasive surgery, not otherwise specified	9
1154	RECTAL	Rectal resection	9
1164	SPINE	Spine surgery, excluding trauma	9
1174	TIP	Typhoid intestinal perforation	9
1002	AABDO	Acute abdomen but not meeting other abdominal surgery codes	8
1005	ACHI	All-comer head injury	8
1018	BILD	Bile duct procedures, excluding Whipple procedure	8
1095	INTUSS	Intussusception	8
1168	TAD	Thoracic aortic disease	8
1001	AAA	Abdominal aortic aneurysm	7
1027	CARDAN	Cardiac ventricular aneurysm	7
1028	CARDIACM	Cardiac myxoma	7
1048	CTRAUM	Cardiac trauma	7
1102	LIVHYDAT	Hepatic hydatidosis	7
1008	ANEUR	Intra-cranial aneurysm	6
1060	ENDOCARD	Endocarditis	6
1087	HYDRO	Hydrocephalus	6
1135	PANC_RES	Pancreatic resection, excluding Whipple procedures or trauma	6
1150	PVI	Peripheral vascular injury	6
1171	THORHYDA	Thoracic hydatidosis	6
1186	VASCU	Vascular procedures, not otherwise specified	6
1020	BRES	Bowel resection, not otherwise specified	5
1072	FOURN	Fournier's gangrene	5
1083	HIPFRAC	Hip fracture	5
1089	HYST	Hysterectomy, excluding emergency peripartum hysterectomy	5
1123	NEURO	Neurosurgical procedures, not otherwise specified	5
1145	PROST	Prostatectomy	5

1165	SPLEEN	Splenic procedures	5
1172	THORTRAU	Thoracic trauma, not otherwise specified	5
1188	VOLV	Colonic volvulus	5
1004	ABDOTB	Abdominal tuberculosis	4
1014	AWH	Abdominal wall hernia	4
1023	BYPASS	Peripheral arterial bypass	4
1031	CDC	Choledochal cyst	4
1032	CDH	Congenital diaphragmatic hernia	4
1076	GASTROSCI	Gastroschisis	4
1079	GOITRE	Goitre	4
1086	HYDAT	Hydatid disease, not otherwise specified	4
1109	MCHD	Mixed (pediatric and adult) congenital heart disease	4
1152	RADCYS	Radical cystectomy	4
1166	STRAUM	Spinal trauma	4
1175	TRACHEOST	Tracheostomy	4
1007	AMPUT	Amputation	3
1009	ANOMAL	Anorectal malformation	3
1015	BABS	Intracranial abscess	3
1022	BURN	Burn injuries	3
1034	CGSW	Cranial gunshot wound	3
1035	CHD	Congenital heart disease, adult population	3
1041	CLEFT	Cleft lip or palate surgery	3
1050	DCL	Damage control laparotomy	3
1052	DIABINF	Diabetic limb infection	3
1055	EATEF	Esophageal atresia or tracheo-esophageal fistula	3
1057	ECTOP	Ectopic pregnancy	3
1064	ESPERF	Esophageal perforation	3
1084	HIRSCH	Hirschprung's disease	3
1097	IATRES	Intestinal atresia	3
1100	LIVABS	Hepatic abscess	3
1126	OBGYNE	Obstetric and gynecologic procedures, not otherwise specified	3
1129	ONCOL	Oncologic diagnoses, not otherwise specified	3
1148	PTRAUM	Pancreatic trauma	3
1169	TDH	Traumatic diaphragmatic hernia	3
1173	THYROID	Thyroid surgery, excluding goitre	3
1177	TRAUM	Trauma, not otherwise specified	3
1179	UGIB	Upper gastrointestinal bleed	3
1190	VVF	Vesicovaginal fistula	3
1011	APR	Abdomino-perineal resection	2
1013	ASO	Arterial Switch Operation	2
1029	CBT	Carotid body tumor	2
1033	CEA	Carotid endarterectomy	2
1039	CIRCUM	Male circumcision	2
1062	EPIL	Epilepsy	2
1080	GSW	Gunshot wound	2
1088	HYPOS	Hypospadias	2
1090	ICBX	Intracranial biopsy	2
1107	MALRO	Malrotation	2
1116	MYOMEC	Myomectomy for uterine fibroids	2
1117	NEC	Necrotising enterocolitis	2
1118	NECFASC	Necrotising fasciitis	2
1122	NEPHREC	Nephrectomy	2
1134	PALL	Palliative procedures for intra-abdominal malignancy	2
1136	PANCRE	Pancreatitis	2
1139	PECTUS	Pectus excavatum	2

1142	PERICAR	Pericardiectomy	2
1146	PTCATH	Peritoneal dialysis catheter placement	2
1156	RELAPCS	Relaparotomy after caesarean section	2
1160	RSS	Renal stone surgery	2
1162	SDH	Subdural hemorrhage	2
1176	TRACHSTEI	Tracheal stenosis	2
1178	TTHR	Tumor thrombus secondary to renal or adrenal malignancy	2
1003	ABDOPR	Abdominal pregnancy	1
1006	AFIB	Atrial fibrillation	1
1012	ASCAR	Ascariasis	1
1016	BIAL	Bilateral internal iliac artery ligation	1
1017	BILAT	Biliary atresia	1
1021	BTL	Bilateral tubal ligation	1
1037	CHOLESTEA	Cholesteatoma	1
1038	CHYST	Ceasarean hysterectomy	1
1040	CLE	Congenital lobar emphysema	1
1042	COLOST	Colostomy, not otherwise specified	1
1044	CORANOM	Coronary vessel anomalies	1
1045	CRANHYDA	Intracranial hydatitosis	1
1046	CROHN	Bowel resection for Crohn's disease	1
1047	CRUPT	Cardiac rupture	1
1049	CWR	Chest wall resection	1
1051	DECORT	Decortication for empyema	1
1053	DISC	Discectomy	1
1054	DPSS	Placement of diaphragmatic pacer	1
1056	ECF	Enterocutaneous fistula	1
1058	EDH	Epidural hematoma	1
1059	EGYNE	Emergency gynecologic surgery, excluding ectopic pregnancy	1
1065	EVISC	Evisceration	1
1066	EXENT	Pelvic exenteration	1
1067	FASC	Fasciotomy	1
1068	FEME	Frontoethmoidal meningoencephalocele	1
1069	FEMFRAC	Femur fracture	1
1070	FIA	Fistula-in-ano	1
1071	FORBOD	Airway foreign body	1
1073	FTI	Flexor tendon injury	1
1074	GAST	Gastric procedure, not otherwise specified	1
1077	GASTROJ	Gastrojejunostomy, not otherwise specified	1
1078	GENS	General surgery	1
1081	HELLER	Heller myotomy	1
1082	HEMOR	Hemorrhoidectomy	1
1085	HNC	Head and neck cancer	1
1092	ILEALC	Ileal conduit	1
1093	ILEOST	Ileostomy, not otherwise specified	1
1096	IPAA	Ileal pouch-anal anastomosis	1
1099	LAPORCH	Laparoscopic orchidecomy	1
1101	LIVAMOEB	Hepatic amoebiasis	1
1105	LLU	Lower extremity ulcer, not otherwise specified	1
1108	MASTOID	Mastoidectomy	1
1110	MEDIAST	Mediastinitis	1
1111	MEDMASS	Mediastinal mass	1
1114	MOYA	Moyamoya disease	1
1119	NECKDIS	Neck dissection	1
1120	NECKMA	Neck mass	1

1121	NEOPNEUI	Pneumothorax in neonates	1
1124	NISSN	Nissen fundoplication	1
1125	NPCR	Nasopharyngeal carcinoma resection	1
1127	OMD	Omphalomesenteric duct remnant	1
1128	OMPHAL	Omphalocele	1
1130	ORBTRAUI	Orbital trauma	1
1131	OTRAUM	Orthopedic trauma, not otherwise specified	1
1132	OVACA	Ovarian malignancy	1
1137	PARATHYR	Parathyroidectomy	1
1140	PEMB	Pulmonary embolectomy	1
1143	PLACENTA	Placenta accreta	1
1144	PNECK	Penetrating neck trauma	1
1147	PTM	Post-traumatic meningitis	1
1149	PULC	Pressure ulcer	1
1151	PYLORSTEI	Pyloric stenosis	1
1153	RCFUN	Rhinocerebral fungal infection	1
1155	RECTO	Rectopexy	1
1157	RHEPAT	Ruptured hepatoma	1
1159	ROHS	Reoperation after open heart surgery	1
1161	SACRO	Sacrococcygeal teratoma	1
1163	SKINCA	Dermatologic malignancy	1
1167	STROKE	Neurosurgical intervention for stroke	1
1170	THAL	Thal procedure	1
1180	URETEROS	Ureterostomy	1
1181	URETHRAL	Urethral stricture	1
1182	UROL	Urological procedures, not otherwise specified	1
1185	VALVEOBS	Reoperation for obstructed mechanical cardiac valve	1
1187	VENOM	Surgery for snake evenomation	1
1189	VULVCA	Vulvar carcinoma	1
1192	XDRTB	Pulmonary resection for XDR-TB	1
			985

CARDI Cardiac surgery

All cardiac surgery procedure types, excluding cardiac trauma, which is classified with trauma. See right for included procedure codes.

ENT Otorhinolaryngology- Head and Neck Surgery

All ENT procedure types. Includes thyroid/goitre and parathyroid surgery. See right for included procedure codes.

GENSX General surgery

All general surgery procedure types, excluding HPB, pediatrics, trauma, or thoracics, which are classified separately. See right for included procedure codes.

HEPAT Hepatobiliary and pancreatic surgery

All HPB procedure types, excluding pancreatic and liver trauma, which are classified with trauma. See right for included procedure codes.

MULTI Multi-specialty patient population

Imputed when a multi-specialty patient population was studied, for example, all surgical mortalities at a single institution.

NEURO Neurological surgery

All neurological surgery procedure types. See right for included procedure codes.

OBGYN Obstetrics and gynecology

All obstetrics and gynecology surgery procedure types. See right for included procedure codes.

ORTHO Orthopedic surgery

All orthopedic surgery procedure types. See right for included procedure codes.

PAEDS Pediatric surgery

All pediatric surgery procedure types, excluding cardiac, cleft surgery, urology, or neurosurgery, which are classified separately. See right for included procedure codes

PLAST Plastic surgery

All plastic surgery procedure types. See right for included procedure codes.

THORA Thoracic surgery

All thoracic surgery procedure types, excluding pediatric thoracic surgery and thoracic trauma, which are classified with pediatric surgery and trauma surgery, respectively. See right for included procedure codes.

TRAUM Trauma surgery

All trauma surgery procedure types. Includes burn surgery. See right for included procedure codes.

UROL Urological surgery

All urological surgery procedure types. Includes Fournier's Gangrene. See right for included procedure codes.

VASCU Vascular surgery

All vascular surgery procedure types. Excludes peripheral vascular injury, which is classified with trauma surgery. See right for included procedure codes

CARDI	ENT	GENSX	HEPAT	MULTI	NEURO	OBGYN	ORTHO	PAEDS	PLAST	THORA	TRAUM	UROL	VASCU
AFIB	CBT	AABDO	BILAT	MULTI	ACHI	ABDOPR	AMPUT	AABDO	CLEFT	CWR	BURN	CIRCUM	AAA
ASO	GOITRE	ABDOTB	BILD		ANEUR	BIAL	FEMFRAC	ABDOTB	FTI	DECORT	CTRAUM	FOURN	BYPASS
CABG	HNC	APPY	CDC		BABS	BTL	HIPFRAC	ANOMAL	LLU	DPSS	DCL	HYPOS	CARDAN
CARD	MASTOID	APR	LIVABS		CGSW	CAES	NECFASC	ASCAR	PULC	ESOCA	FASC	ILEALC	CEA
CARDAN	NECKDIS	AWH	LIVAMOEB		CRANHYD	CHYST	OTRAUM	AWH		ESPERF	GSW	LAPORCH	DIABINF
CARDIACM	PARATHYR	BOBS	LIVHYDAT		DISC	ECTOP		BOBS		LUNGRES	LAPAR	NEPHREC	VASCU
CCHD	RCFUN	BRES	LIVRES		EDH	EGYNE		BRES		MEDMASS	LIVTRAUM	PROST	
CHD	THYROID	CHOLE	PALL		EPIL	EPH		CDH		NEOPNEU	ORBTRAUM	RADCYS	
CORANOM	TRACHEO	COLRES	PANC_RES		FEME	HYST		CHOLESTEA		PEMB	PNECK	RSS	
CRUPT		CROHN	PANCRE		HYDRO	MIS		CLE		THORHYDA	PTRAUM	TTHR	
CTRAUM		DCL	RHEPAT		ICBX	MYOMEC		EATEF		TRACHSTE	PVI	URETEROST	
ENDOCARD		ECF	WHIP		ICH	OBGYNE		FORBOD		XDRTB	SPLEEN	URETHRAL	
MCHD		EVISC			MOYA	OVACA		GASTROSCH			STRAUM	UROL	
PCARD		EXENT			NEURO	PALL		HIRSCH			TDH		
PERICAR		FIA			NPCR	PERF		INGHERN			THORTRAUM		
ROHS		GAST			PTM	PLACENTAACC		INTUSS			TRAUM		
TAD		GASTCA			RIM	RELAPCS		JIATRES					
VALVE		GENS			SACRO	UTRUP		LAPAR					
VALVEOBS		HELLER			SDH	VULVCA		MALRO					
		HEMOR			SPINE	VVF		MEDIAST					
		HYDAT			STRAUM			MIS					
		ILEOST			STROKE			NEC					
		INGHERN						NECKMA					
		INTUSS						OMD					
		IPAA						ONCOL					
		LAPAR						PAED					
		LIVRES						PECTUS					
		LLU						PERF					
		MIS						PYLORSTEN					
		NECFASC						RSS					
		NISSN						TIP					
		ONCOL						TRACHEOST					
		PERF						VENOM					
		PTCATH											
		RECTAL											
		RECTO											
		SKINCA											
		SPLEEN											
		THAL											
		THYROID											
		TIP											
		UGIB											
		VOLV											

Exclusion reason	Exclusion code
Burn mortality study without perioperative mortality reporting	BURN
Trauma mortality without perioperative mortality reporting	TRAUMA
Endoscopic intervention	ENDOS
No perioperative mortality reporting (other than trauma, burns, or maternal mortality)	NOPOMR
Survival analysis for cancer without POMR reporting	CANCER
Bariatric Surgery	BARIATRIC
Not in LMIC	HIC
Maternal mortality studies without reporting of perioperative mortality	MATERNAL
Fewer than 5 pts	FEWER
ICU study base	ICU
Robotic surgery	ROBOT
Same patient population included in other study	DUPLICATE
Full text not in English	LANGUAGE
Burr hole study	BURR
Review study with no new primary data	REVIEW
Short term surgical mission	MISSION
Percutaneous interventions	PERCU
Study unavailable	UNAVAIL
Termination of pregnancy	TERMIN
Editorial	EDITOR
Surgery performed in temporary combat hospitals	MILITARY
Survival analysis for pericardial effusion	PERIC
Article not in academic journal	NONAC
Fetal surgery	FETAL
Patients transported to HIC	THIC
Study of transplantation	TRANSPLANT
Retracted article	RETRACT
Heated IntraPeritoneal Chemotherapy	HIPEC
No surgical procedure (performed in OR) reported	NOSURG

Code	Risk Score
PCS	Procedure Complexity Score
WHO_HIV	WHO HIV stage
ONC_STG	Oncologic stage
BMI	Body Mass Index
ASA	American Society of Anesthesiologists physical status classification
NYHA	New York Heart Association
CHILD	Child's score
GCS	Glasgow Coma Scale
ICH	Intracranial hemorrhage score
EURO_	EuroSCORE
TBSA	Total Body Surface Area
LA50	Lethal Area-50
POTI	Perforation-Operation Time Interval
ABC_	Aristotle Basic Complexity Score
AIS	Abbreviated Injury Scale
OIS	AAST Organ Injury Scale
SINO	SinoSCORE
CHARL	Charlson Comorbidity Index
EPASS	E-PASS system
RACHS	RACHS-1 score
STSEACTS	STS-EACTS score
BOEY	Boey score
MPI	Mannheim Peritonitis Index
BALT	Balthazar Score
GUARA	GuaragnaSCORE
POSSUM	Possum score
PPOSS	P-Possum score
CRPOSS	Colorectal-Possum score
BELL	Bell Classification
JPS	Jabalpur Peritonitis Score
APGAR	Surgical APGAR score
KPI	Karnofski Performance Index
BOAS	Brazil Old Age Schedule
APACHE2	Acute Physiology and Chronic Health Evaluation-2 score
MODS	Multiple Organ Dysfunction Score
SAPS3	SAPS3 score
PARSON	Parsonnet Score
CAROSELLA	Carosella score
WATERS	Waterston classification
MONTREAL	Montreal classification
SPITZ	Spitz classification
BREMEN	Bremen classification
FRANKEL	Frankel classification
BENED	Benedetti score
10GROUP	10-group classification

HUNT	Hunt-Hess SAH scale
WAGNER	Wagner diabetic foot classification
KAPLAN	Kaplan-Feinstein comorbidity index
KRICKEN	Krickenbeck anorectal malformations classification
VA	Veteran's Administration risk score- Cardiac valve replacement
NNE	Northern New England risk score
AMBLER	Ambler risk score (heart valve surgery)
NYC	NYC cardiac valve scoring system
VMCP	Cardiac valve scoring system
RTS	Revised Trauma Score
GOITRE	WHO Goitre grade
EURO2	EuroSCOREII
ISS_	Injury Severity Score
NURICK	Nurick grade of functional disability in patients with cervical spondylotic myelopathy
MELD	Model for End-Stage Liver Disease
MESS	Mangled Extremity Severity Score
TODANI	Todani classification of choledochal cysts
NEVES	Neves classification of renal tumor thrombus extension
ASIA	ASIA impairment scale for neurological classification of spinal cord injury
TASH	Trauma-Associated Severe Hemorrhage
MCLAU	McLaughlin risk model for trauma
NISS	New Injury Severity Score
ABCD	New scoring system for trauma(Ordonez et al)
FGSI	Fournier’s Gangrene Severity Index
WFNS	World Federation of Neurological Surgeons grade
TRISS	Trauma and Injury Severity Score
AOCLASS	Arbeitsgemeinschaft fur Osteosynthesefragen Class for femur fractures
ISHAK	Ishak liver fibrosis score
CCS	CCS Functional Classification of Angina
SHAMBLIN	Shamblin classification of carotid body tumors
GOLDMAN	Goldman cardiac risk index
LENKE	Lenke classification of scoliosis
IAHTN	Intra-abdominal hypertension grade
TEXAS	University of Texas wound classification
ALSFRS	ALS Functional Rating Score
BRITT	Brain abscess staging
MRS	Modified Rankin Scale
ACCP	American College of Chest Physicians risk strata for lung resection
FISHER	Fisher subarachnoid hemorrhage scale
APGAR_N	APGAR score (relevant to neonatal surgical patients)
