# Supplementary material for: Perioperative mortality rates in low-income and middle-income countries: a systematic review and meta-analysis
Source: BMJ Glob Health. 2018 Jun 22;3(3):e000810. doi: 10.1136/bmjgh-2018-000810 (PMC6035511; doi:10.1136/bmjgh-2018-000810)
Supplement: Supplementary file 3 [file bmjgh-2018-000810supp003.pdf]

	Country (number of articles providing POMR data)		
Upper-Middle Income	Albania (2)	Cuba (1)	Peru (2)
	Algeria (1)	Ecuador (1)	Romania (10)
	Angola (1)	Hungary (5)	Serbia (12)
	Argentina (7)	Iran (62)	South Africa (27)
	Bosnia and Herzegovina (1)	Jamaica (6)	Thailand (23)
	Botswana (1)	Jordan (8)	Tunisia (6)
	Brazil (145)	Malaysia (8)	Turkey (65)
	China (111)	Mexico (6)	Venezuela (1)
	Colombia (4)	Montenegro (1)	
	Costa Rica (1)	Panama (1)	
Low-Middle Income	Bolivia (1)	India (87)	Senegal (2)
	Cameroon (4)	Indonesia (3)	South Sudan (2)
	Congo, Rep. (1)	Kosovo (3)	Sri Lanka (5)
	Côte D'Ivoire (4)	Mongolia (1)	Sudan (2)
	Egypt (29)	Morocco (1)	Uzbekistan (1)
	El Salvador (2)	Nicaragua (2)	Vietnam (6)
	Georgia (1)	Nigeria (121)	Yemen (2)
	Ghana (11)	Pakistan (107)	Zambia (1)
	Guatemala (3)	Paraguay (1)	
	Honduras (1)	Philippines (4)	
Low-Income	Afghanistan (4)	Ethiopia (9)	Myanmar (1)
	Bangladesh (12)	Gambia, The (1)	Nepal (11)
	Benin (2)	Guinea (1)	Niger (5)
	Burkina Faso (1)	Haiti (2)	Sierra Leone (3)
	Burundi (2)	Kenya (6)	Somalia (2)
	Cambodia (4)	Liberia (1)	Tanzania (31)
	Central African Republic (1)	Malawi (6)	Uganda (10)
	Chad (1)	Mali (6)	Zimbabwe (1)
	Dem. Rep. of the Congo (5)	Mozambique (1)	

Table s1. Number of articles reporting POMR data for each country. Countries classified by 2013 World Bank Income Classification

	Number of papers (%)
By surgical specialty:	
General	202 (20.5%)
Cardiac	191 (19.4%)
Obstetrics and Gynecology	140 (14.2%)
Pediatric	92 (9.3%)
Neurological	86 (8.7%)
Hepatopancreaticobiliary	63 (6.4%)
Trauma (including burns)	55 (5.6%)
Thoracic	54 (5.5%)
Urologic	27 (2.7%)
Vascular	23 (2.3%)
Otorhinolaryngology- HNS	16 (1.6%)
Orthopedic	11 (1.1%)
Plastic	6 (0.6%)
Multiple Specialties	19 (1.9%)

Table s2: Number of papers reporting POMR, by specialty. Each study attributed to the single most relevant specialty where appropriate. *HNS*- Head and Neck Surgery

Table s3. Inverse-variance aggregated POMR by procedure or diagnosis. All studies included, regardless of definition, urgency, study design, or population-specific risk factors.

Diagnostic or procedure code	Description	Number of studies	Total number of deaths	Total denominator	Gross POMR (%)	Median POMR (%)	Range (min, max, %)		Inverse-variance aggregated POMR (%)	95% Confidence Interval		Degrees of Freedom	I-Squared (%)
CAES	Caesarean section	55	805	366501	0.22	0.12	0	15.61	0.05	0	0.13	54	93.52
CABG	Coronary artery bypass graft	49	5807	123513	4.70	3.60	0	52.81	4.38	3.47	5.37	48	97.32
EPH	Emergency peripartum hysterectomy	39	196	2245	8.73	10.30	0	31.03	7.81	5.81	10.04	38	64.27
VALVE	Cardiac valve procedures	35	1197	30365	3.94	4.29	0	15.07	4.17	3.05	5.45	34	93.61
CARD	Cardiac surgery, not otherwise specified	31	7847	123060	6.38	4.79	0	23.08	4.96	3.81	6.25	30	97.80
COLRES	Colon resection, excluding resection for volvul	27	356	12636	2.82	1.86	0	33.64	2.83	1.62	4.31	26	92.35
APPY	Appendicitis	23	28	5237	0.53	0	0	2.78	0.01	0	0.19	22	37.74
LUNGRES	Pulmonary resection, excluding resection for tu	23	96	5630	1.71	1.14	0	13.76	1.3	0.48	2.41	22	84.46
PERF	Perforated hollow viscus, excluding perforatio	22	264	2427	10.88	11.85	0	40.00	11.85	8.35	15.83	21	84.73
LIVRES	Hepatic resection	20	99	7243	1.37	1.38	0	13.16	1.04	0.32	2.02	19	77.08
MULTI	Multi-specialty patient population, usually insti	19	1645	177283	0.93	1.06	0.16	7.36	1.29	0.77	1.94	18	99.02
PCARD	Pediatric cardiac procedures, excluding comple	19	538	6618	8.13	7.14	0	24.24	6.76	4.99	8.75	18	81.36
RIM	Resection of intracranial mass	19	20	814	2.46	0	0	5.88	1.29	0.41	2.51	18	0.00
GASTCA	Gastric cancer	18	267	8250	3.24	2.71	0	18.97	3.72	1.92	6.01	17	94.48
INGHERN	Inguinal hernia	17	71	11196	0.63	0	0	9.73	0.38	0	1.22	16	93.48
LAPAR	Laparotomy, not meeting other abdominal surg	17	354	3064	11.55	11.11	4.94	42.11	12.53	9.39	16.04	16	83.70
PAED	Pediatric surgical procedures, not otherwise sp	17	355	54389	0.65	3.57	0	62.22	6.16*	4.06	8.64	16	98.64
CHOLE	Cholecystectomy	15	4	6088	0.07	0	0	0.15	0	0	0	14	0.00
UTRUP	Uterine rupture	15	87	1169	7.44	8.22	0	17.50	7.36	4.42	10.88	14	71.74
ESOCA	Esophageal carcinoma	13	134	1802	7.44	5.81	0	24.00	5.4	2.28	9.54	12	89.16
CCHD	Complex congenital heart disease	12	93	596	15.60	14.65	0	61.54	14.94	7.03	24.75	11	83.32
BOBS	Bowel obstruction	10	137	1158	11.83	8.79	2.27	38.10	12.32	6.77	19.15	9	89.13
ICH	Intracranial hemorrhage	10	224	1011	22.16	25.48	3.77	62.22	24.47	15.88	34.16	9	89.05
LIVTRAUM	Hepatic trauma	10	133	909	14.63	17.01	6.80	61.11	15.84	10.31	22.16	9	76.86
WHIP	Whipple pancreaticoduodenectomy	10	90	2065	4.36	2.84	0	9.92	2.94	1.61	4.57	9	52.48
MIS	Minimally invasive surgery, not otherwise spec	9	2	1314	0.15	0	0	4.17	0	0	0.1	8	0.00
RECTAL	Rectal resection	9	6	1032	0.58	0	0	5.88	0.07	0	0.92	8	50.09
SPINE	Spine surgery, excluding trauma	9	11	518	2.12	0	0	8.96	0.77	0	3.8	8	67.18
TIP	Typhoid intestinal perforation	9	134	662	20.24	20.73	4.55	33.33	20.09	14.36	26.48	8	71.46
AABDO	Acute abdomen but not meeting other abdomi	8	228	2877	7.92	10.42	4.90	34.88	11.2	7.42	15.62	7	86.27
ACHI	All-comer head injury	8	377	1390	27.12	23.08	10.00	54.58	27.2	14.98	41.39	7	96.32
BILD	Bile duct procedures, excluding Whipple proce	8	51	714	7.14	2.30	0.00	21.54	4.08	0.1	11.63	7	91.49
INTUSS	Intussusception	8	43	355	12.11	3.66	0.00	33.70	4.8	0.03	14.28	7	88.09
TAD	Thoracic aortic disease	8	775	4203	18.44	8.66	0.00	20.30	9.5	3.96	16.74	7	91.49
AAA	Abdominal aortic aneurysm	7	166	1692	9.81	10.53	8.50	50.00	10.9	7.42	14.87	6	66.75
CARDAN	Cardiac ventricular aneurysm	7	41	925	4.43	5.00	2.01	14.29	5.57	2.28	9.98	6	74.97
CARDIACMYX	Cardiac myxoma	7	17	500	3.40	4.17	0	5.41	2.24	0.34	5.22	6	53.25
CTRAUM	Cardiac trauma	7	53	285	18.60	18.27	0	23.29	17	12.46	21.99	6	0.00
LIVHYDAT	Hepatic hydatidosis	7	29	2050	1.41	0	0	2.82	0.46	0	1.54	6	57.80
ANEUR	Intra-cranial aneurysm	6	93	881	10.56	7.28	2.05	16.77	7.99	3.57	13.84	5	85.62
ENDOCARD	Endocarditis	6	124	471	26.33	23.01	15.52	38.71	25.23	15.53	36.27	5	79.98
HYDRO	Hydrocephalus	6	18	1067	1.69	2.51	0	4.17	1.6	0.22	3.8	5	65.10
PANC_RES	Pancreatic resection, excluding Whipple proce	6	5	288	1.74	1.32	0	4.23	0.77	0	3.17	5	29.35
PVI	Peripheral vascular injury	6	23	458	5.02	2.89	0	11.29	3.52	0.63	8.09	5	74.72
THORHYDAT	Thoracic hydatidosis	6	4	1375	0.29	0.10	0	1.15	0	0	0.08	5	0.00
VASCU	Vascular procedures, not otherwise specified	6	110	1284	8.57	6.02	1.85	9.93	6.43	3.78	9.62	5	53.27
BRES	Bowel resection, not otherwise specified	5	12	192	6.25	1.00	0.00	31.82	4.92	0	17.39	4	79.25
FOURN	Fournier's gangrene	5	34	162	20.99	20.00	0.00	30.77	14.22	3.31	29.65	4	73.65
HIPFRAC	Hip fracture	5	108	1380	7.83	8.94	4.31	10.76	7.73	5.15	10.77	4	72.87
HYST	Hysterectomy, excluding emergency peripartu	5	7	460	1.52	0	0	7.02	0.98	0	4.16	4	74.66
NEURO	Neurosurgical procedures, not otherwise speci	5	78	2430	3.21	5.68	2.05	16.67	5.78	1.87	11.3	4	88.22
PROST	Prostatectomy	5	7	1050	0.67	1.01	0	1.75	0.4	0.04	1	4	0.00
SPLEEN	Splenic procedures	5	36	278	12.95	10.00	0	21.90	8.86	2.01	18.84	4	76.20
THORTRAUM	Thoracic trauma, not otherwise specified	5	38	343	11.08	10.19	6.67	15.38	10.07	6.83	13.77	4	0.00
VOLV	Colonic volvulus	5	39	811	4.81	10.42	0.84	14.63	6.39	1.84	13.03	4	86.25
ABDOTB	Abdominal tuberculosis	4	73	440	16.59	14.65	3.05	38.78	15.66	2.66	35.53	3	94.48
AWH	Abdominal wall hernia	4	5	1307	0.38	1.89	0.00	4.88	0.84	0	3.92	3	82.72
BYPASS	Peripheral arterial bypass	4	16	424	3.77	2.85	2.30	18.18	4.24	0.98	9.22	3	70.13
CDC	Choledochal cyst	4	3	1147	0.26	0.10	0	0.88	0	0	0.18	3	0.00
CDH	Congenital diaphragmatic hernia	4	16	65	24.62	32.67	0	42.86	21.72	1.56	52.18	3	82.21
GASTROSCH	Gastroschisis	4	113	455	24.84	27.64	10.29	62.96	29.68	10.75	53.14	3	96.11
GOITRE	Goitre	4	0	223	0.00	0.00	0	0	0	0	0.88	3	0.00
HYDAT	Hydatid disease, not otherwise specified	4	3	207	1.45	1.64	0	5.88	0.66	0	4.92	3	50.97
MCHD	Mixed (pediatric and adult) congenital heart di	4	2	695	0.29	0.96	0	2.50	0.19	0	2.04	3	60.36
RADCYS	Radical cystectomy	4	8	482	1.66	9.07	1.00	20.00	0.02	0	2.67	3	55.26
STRAUM	Spinal trauma	4	17	135	12.59	10.35	4.76	20.41	11.25	4.38	20.32	3	46.81
TRACHEOST	Tracheostomy	4	43	540	7.96	15.47	0	20.00	9.56	0.24	27.7	3	95.95
AMPUT	Amputation	3	55	382	14.40	16.03	4.69	16.67	12.6	6.61	20.1	2	-
ANOMAL	Anorectal malformation	3	3	115	2.61	2.50	2.17	3.45	2.54	0.14	6.75	2	-
BABS	Intracranial abscess	3	23	136	16.91	16.67	5.88	40.00	18.09	4.29	37.78	2	-
BURN	Burn injuries	3	34	495	6.87	4.46	1.54	14.11	5.79	0.9	14.13	2	-
CGSW	Cranial gunshot wound	3	70	575	12.17	13.33	10.63	19.32	13.52	8.18	19.89	2	-
CHD	Congenital heart disease, adult population	3	14	479	2.92	2.96	1.31	4.19	2.77	1.29	4.73	2	-
CLEFT	Cleft lip or palate surgery	3	0	292	0.00	0	0	0	0	0	0.68	2	-
DCL	Damage control laparotomy	3	45	375	12.00	11.18	5.56	26.92	13.05	4.78	24.11	2	-
DIABINF	Diabetic limb infection	3	25	205	12.20	13.79	5.56	15.05	11.54	6.27	18.04	2	-
EATEF	Esophageal atresia or tracheo-esophageal fistul	3	42	153	27.45	23.40	8.70	45.00	24.41	6.76	48.04	2	-
ECTOP	Ectopic pregnancy	3	0	200	0.00	0.00	0.00	0	0	0	0.95	2	-
ESPERF	Esophageal perforation	3	7	76	9.21	9.68	6.67	10.00	9.02	3.06	17.11	2	-
HIRSCH	Hirschprung's disease	3	28	159	17.61	16.00	0	21.82	10.65	0.42	29.11	2	-
IATRES	Intestinal atresia	3	17	54	31.48	33.33	21.74	40.91	30.95	18.71	44.53	2	-
LIVABS	Hepatic abscess	3	17	144	11.81	9.45	0	62.50	15.86	0	50.47	2	-
OBGYNE	Obstetric and gynecologic procedures, not oth	3	157	9747	1.61	0.83	0	1.78	0.8	0.13	1.92	2	-
ONCOL	Oncologic diagnoses, not otherwise specified	3	8	297	2.69	2.56	0	7.14	1.97	0.05	5.58	2	-
PTRAUM	Pancreatic trauma	3	56	275	20.36	21.00	11.54	23.33	19.87	15.21	24.95	2	-
TDH	Traumatic diaphragmatic hernia	3	7	64	10.94	11.11	0	13.79	9.59	2.77	18.96	2	-
THYROID	Thyroid surgery, excluding goitre	3	0	345	0.00	0	0	0	0	0	0.19	2	-
TRAUM	Trauma, not otherwise specified	3	9	263	3.42	2.88	0	4.72	2.93	0.99	5.62	2	-
UGIB	Upper gastrointestinal bleed	3	31	97	31.96	18.75	0	70.37	27.24	0.03	71.41	2	-
VVF	Vesicovaginal fistula	3	1	773	0.13	0.00	0	0.20	0	0	0.33	2	-
APR	Abdomino-perineal resection	2	1	50	2.00	1.39	0	2.78	1.33	0	7.88	1	-
ASO	Arterial Switch Operation	2	12	154	7.79	7.90	6.98	8.82	7.76	3.91	12.66	1	-
CBT	Carotid body tumor	2	0	75	0.00	0	0	0	0	0	2.3	1	-
CEA	Carotid endarterectomy	2	1	177	0.56	0.42	0	0.84	0.39	0	2.26	1	-
CIRCUM	Male circumcision	2	0	3204	0.00	0	0	0	0	0	0	1	-
EPIL	Epilepsy	2	2	402	0.50	2.91	0.26	5.56	0	0	0.38	1	-
GSW	Gunshot wound	2	11	76	14.47	14.55	13.95	15.15	14.45	7.16	23.52	1	-
HYPOS	Hypospadias	2	0	179	0.00	0	0	0	0	0	0.96	1	-
ICBX	Intracranial biopsy	2	3	252	1.19	1.60	0.57	2.63	0.96	0.02	2.76	1	-
MALRO	Malrotation	2	4	40	10.00	14.34	6.45	22.22	8.41	0.89	20.3	1	-
MYOMEC	Myomectomy for uterine fibroids	2	0	329	0.00	0	0	0	0	0	0.54	1	-
NEC	Necrotising enterocolitis	2	47	137	34.31	39.70	31.58	47.83	34	26.13	42.31	1	-
NECFASC	Necrotising fasciitis	2	11	129	8.53	7.18	5.26	9.09	7.91	3.51	13.58	1	-
NEPHREC	Nephrectomy	2	1	574	0.17	0.09	0	0.18	0	0	0.01	1	-
PALL	Palliative procedures for intra-abdominal malig	2	15	70	21.43	23.75	7.50	40.00	19.19	10.55	29.51	1	-
PANCRE	Pancreatitis	2	11	94	11.70	22.08	5.26	38.89	9.11	3.7	16.22	1	-
PECTUS	Pectus excavatum	2	0	528	0.00	0.00	0	0	0	0	0.29	1	-
PERICAR	Pericardiectomy	2	18	139	12.95	9.80	5.56	14.05	12.23	6.99	18.52	1	-
PTCATH	Peritoneal dialysis catheter placement	2	5	398	1.26	4.46	0	8.93	0.09	0	0.9	1	-
RELAPCS	Relaparotomy after caesarean section	2	9	50	18.00	18.27	11.54	25.00	17.54	7.81	29.74	1	-
RSS	Renal stone surgery	2	2	542	0.37	0.19	0	0.38	0	0	0	1	-
SDH	Subdural hemorrhage	2	50	330	15.15	14.38	11.20	17.56	15.01	11.32	19.1	1	-
TRACHSTEN	Tracheal stenosis	2	3	57	5.26	15.29	2.00	28.57	2.02	0	9.14	1	-
TTHR	Tumor thrombus secondary to renal or adrenal	2	2	19	10.53	7.14	0	14.29	8.11	0	27.57	1	-
ABDOPR	Abdominal pregnancy	1	0	9	0.00	0	0	0	0	0	33.63	0	-
AFIB	Atrial fibrillation	1	0	10	0.00	0	0	0	0	0	30.85	0	-
ASCAR	Ascariasis	1	1	79	1.27	1.27	1.27	1.27	1.27	0.03	6.85	0	-
BIAL	Bilateral internal iliac artery ligation	1	1	8	12.50	12.50	12.50	12.50	12.5	0.32	52.65	0	-
BILAT	Biliary atresia	1	2	15	13.33	13.33	13.33	13.33	13.33	1.66	40.46	0	-
BTL	Bilateral tubal ligation	1	0	78	0.00	0	0	0	0	0	4.62	0	-
CHOLESTEA	Cholesteatoma	1	1	61	1.64	1.64	1.64	1.64	1.64	0.04	8.8	0	-
CHYST	Ceasarean hysterectomy	1	2	28	7.14	7.14	7.14	7.14	7.14	0.88	23.5	0	-
CLE	Congenital lobar emphysema	1	4	26	15.38	15.38	15.38	15.38	15.38	4.36	34.87	0	-
COLOST	Colostomy, not otherwise specified	1	2	38	5.26	5.26	5.26	5.26	5.26	0.64	17.75	0	-
CORANOM	Coronary vessel anomalies	1	1	9	11.11	11.11	11.11	11.11	11.11	0.28	48.25	0	-
CRANHYDAT	Intracranial hydatitosis	1	2	97	2.06	2.06	2.06	2.06	2.06	0.25	7.25	0	-
CROHN	Bowel resection for Crohn's disease	1	3	46	6.52	6.52	6.52	6.52	6.52	1.37	17.9	0	-
CRUPT	Cardiac rupture	1	6	19	31.58	31.58	31.58	31.58	31.58	12.58	56.55	0	-
CWR	Chest wall resection	1	0	19	0.00	0	0	0	0	0	17.65	0	-
DECORT	Decortication for empyema	1	31	206	15.05	15.05	15.05	15.05	15.05	10.46	20.68	0	-
DISC	Discectomy	1	1	95	1.05	1.05	1.05	1.05	1.05	0.03	5.73	0	-
DPSS	Placement of diaphragmatic pacer	1	0	11	0.00	0	0	0	0	0	28.49	0	-
ECF	Enterocutaneous fistula	1	0	22	0.00	0	0	0	0	0	15.44	0	-
EDH	Epidural hematoma	1	2	18	11.11	11.11	11.11	11.11	11.11	1.38	34.71	0	-
EGYNE	Emergency gynecologic surgery, excluding ect	1	6	105	5.71	5.71	5.71	5.71	5.71	2.13	12.02	0	-
EVISC	Evisceration	1	14	31	45.16	45.16	45.16	45.16	45.16	27.32	63.97	0	-
EXENT	Pelvic exenteration	1	2	59	3.39	3.39	3.39	3.39	3.39	0.41	11.71	0	-
FASC	Fasciotomy	1	8	70	11.43	11.43	3.39	11.43	11.43	5.07	21.28	0	-
FEME	Frontoethmoidal meningoencephalocele	1	4	200	2.00	2.00	2.00	2.00	2	0.55	5.04	0	-

FEMFRAC	Femur fracture	1	0	96	0.00	0	0	0	0	0	3.77	0	-
FIA	Fistula-in-ano	1	0	8	0.00	0	0	0	0	0	36.94	0	-
FORBOD	Airway foreign body	1	0	7	0.00	0	0	0	0	0	40.96	0	-
FTI	Flexor tendon injury	1	0	31	0.00	0	0	0	0	0	11.22	0	-
GAST	Gastric procedure, not otherwise specified	1	0	5	0.00	0	0	0	0	0	52.18	0	-
GASTROJ	Gastrojejunostomy, not otherwise specified	1	0	32	0.00	0	0	0	0	0	10.89	0	-
GENS	General surgery	1	6	100	6.00	6.00	6.00	6.00	6	2.23	12.6	0	-
HELLER	Heller myotomy	1	0	60	0.00	0	0	0	0	0	5.96	0	-
HEMOR	Hemorrhoidectomy	1	0	43	0.00	0	0	0	0	0	8.22	0	-
HNC	Head and neck cancer	1	2	184	1.09	1.09	1.09	1.09	1.09	0.13	3.87	0	-
ILEALC	Ileal conduit	1	0	16	0.00	0.00	0.00	0.00	0	0	20.59	0	-
ILEOST	Ileostomy, not otherwise specified	1	0	68	0.00	0	0	0	0	0	5.28	0	-
IPAA	Ileal pouch-anal anastomosis	1	2	49	4.08	4.08	4.08	4.08	4.08	0.5	13.98	0	-
LAPORCH	Laparoscopic orchidecomy	1	0	48	0.00	0	0	0	0	0	7.4	0	-
LIVAMOEB	Hepatic amoebiasis	1	6	16	37.50	37.50	37.50	37.50	37.5	15.2	64.57	0	-
LLU	Lower extremity ulcer, not otherwise specified	1	10	100	10.00	10.00	10.00	10.00	10	4.9	17.62	0	-
MASTOID	Mastoidectomy	1	0	6	0.00	0	0	0	0	0	45.93	0	-
MEDIAST	Mediastinitis	1	1	16	6.25	6.25	6.25	6.25	6.25	0.16	30.23	0	-
MEDMASS	Mediastinal mass	1	17	105	16.19	16.19	16.19	16.19	16.19	9.72	24.65	0	-
MOYA	Moyamoya disease	1	2	25	8.00	8.00	8.00	8.00	8	0.98	26.03	0	-
NECKDIS	Neck dissection	1	1	14	7.14	7.14	7.14	7.14	7.14	0.18	33.87	0	-
NECKMA	Neck mass	1	12	148	8.11	8.11	8.11	8.11	8.11	4.26	13.73	0	-
NEOPNEUMO	Pneumothorax in neonates	1	6	10	60.00	60.00	60.00	60.00	60	26.24	87.84	0	-
NISSN	Nissen fundoplication	1	0	25	0.00	0	0	0	0	0	13.72	0	-
NPCR	Nasopharyngeal carcinoma resection	1	0	18	0.00	0	0	0	0	0	18.53	0	-
OMD	Omphalomesenteric duct remnant	1	2	29	6.90	6.90	6.90	6.90	6.9	0.85	22.77	0	-
OMPHAL	Omphalocele	1	20	82	24.39	24.39	24.39	24.39	24.39	15.58	35.12	0	-
ORBTRAUM	Orbital trauma	1	1	9	11.11	11.11	11.11	11.11	11.11	0.28	48.25	0	-
OTRAUM	Orthopedic trauma, not otherwise specified	1	6	248	2.42	2.42	2.42	2.42	2.42	0.89	5.19	0	-
OVACA	Ovarian malignancy	1	0	60	0.00	0	0	0	0	0	5.96	0	-
PARATHYROID	Parathyroidectomy	1	0	43	0.00	0	0	0	0	0	8.22	0	-
PEMB	Pulmonary embolectomy	1	2	16	12.50	12.50	12.50	12.50	12.5	1.55	38.35	0	-
PLACENTAACC	Placenta accreta	1	1	41	2.44	2.44	2.44	2.44	2.44	0.06	12.86	0	-
PNECK	Penetrating neck trauma	1	11	98	11.22	11.22	11.22	11.22	11.22	5.74	19.2	0	-
PTM	Post-traumatic meningitis	1	19	52	36.54	36.54	36.54	36.54	36.54	23.62	51.04	0	-
PULC	Pressure ulcer	1	2	55	3.64	3.64	3.64	3.64	3.64	0.44	12.53	0	-
PYLORSTEN	Pyloric stenosis	1	0	63	0.00	0	0	0	0	0	5.69	0	-
RCFUN	Rhinocerebral fungal infection	1	12	28	42.86	42.86	42.86	42.86	42.86	24.46	62.82	0	-
RECTO	Rectopexy	1	0	12	0.00	0.00	0.00	0.00	0	0	26.46	0	-
RHEPAT	Ruptured hepatoma	1	1	20	5.00	5.00	5.00	5.00	5	0.13	24.87	0	-
ROHS	Reoperation after open heart surgery	1	24	282	8.51	8.51	8.51	8.51	8.51	5.53	12.4	0	-
SACRO	Sacrococcygeal teratoma	1	3	36	8.33	8.33	8.33	8.33	8.33	1.75	22.47	0	-
SKINCA	Dermatologic malignancy	1	6	154	3.90	3.90	3.90	3.90	3.9	1.44	8.29	0	-
STROKE	Neurosurgical intervention for stroke	1	4	20	20.00	20.00	20.00	20.00	20	5.73	43.66	0	-
THAL	Thal procedure	1	0	29	0.00	0	0	0	0	0	11.94	0	-
URETEROST	Ureterostomy	1	0	41	0.00	0	0	0	0	0	8.6	0	-
URETHRAL	Urethral stricture	1	0	91	0.00	0	0	0	0	0	3.97	0	-
UROL	Urological procedures, not otherwise specified	1	0	71	0.00	0	0	0	0	0	5.06	0	-
VALVEOBS	Reoperation for obstructed mechanical cardiac	1	23	129	17.83	17.83	17.83	17.83	17.83	11.65	25.54	0	-
VENOM	Surgery for snake evenomation	1	0	13	0.00	0	0	0	0	0	24.71	0	-
VULVCA	Vulvar carcinoma	1	0	11	0.00	0	0	0	0	0	28.49	0	-
XDRTB	Pulmonary resection for XDR-TB	1	0	5	0.00	0	0	0	0	0	52.18	0	-

*NB for procedure or diagnostic groups where a large discrepancy exists between gross POMR and inverse-variance aggregated POMR, there is likely to be a significant difference in reported POMR for large versus small studies and significant heterogeneity in underlying study populations.

Table s4. Inverse-variance aggregated POMR by procedure or diagnosis. Studies based in a high-risk population, such as those with a specific comorbidity, excluded.

Diagnostic or procedure code	Description	Number of studies	Total number of deaths	Total denominator	Gross POMR (%)	Median (%)	Range (min, max, %)		*Inverse variance* -aggregated POMR (%)	95% Confidence Interval		Degrees of Freedom	I-Squared (%)
CAES	Caesarean section	45	794	365685	0.22	0.19	0	15.61	0.29	0.18	0.42	44	94.46
CABG	Coronary artery bypass graft	37	5694	122069	4.66	3.56	0	52.81	4.1	3.17	5.14	36	97.83
EPH	Emergency peripartum hysterectomy	39	196	2245	8.73	10.30	0	31.03	7.81	5.81	10.04	38	64.27
VALVE	Cardiac valve procedures	34	1192	30265	3.94	4.07	0	15.07	4.15	3.01	5.44	33	93.79
CARD	Cardiac surgery, not otherwise specified	23	7663	120129	6.38	4.79	0	17.65	4.87	3.68	6.22	22	98.27
COLRES	Colon resection, excluding resection for volvul	26	288	10815	2.66	1.86	0	33.64	2.81	1.51	4.43	25	92.29
APPY	Appendicitis	20	28	5054	0.55	0	0	2.78	0.02	0	0.23	19	45.93
LUNGRES	Pulmonary resection, excluding resection for tu	23	96	5630	1.71	1.14	0	13.76	1.3	0.48	2.41	22	84.46
PERF	Perforated hollow viscus, excluding perforatio	22	264	2427	10.88	11.85	0	40.00	11.85	8.35	15.83	21	84.73
LIVRES	Hepatic resection	18	83	6932	1.20	1.38	0	13.16	0.64	0.13	1.4	17	67.06
MULTI	Multi-specialty patient population, usually insti	19	1645	177283	0.93	1.06	0.16	7.36	1.29	0.77	1.94	18	99.02
PCARD	Pediatric cardiac procedures, excluding comple	19	538	6618	8.13	7.14	0	24.24	6.76	4.99	8.75	18	81.36
RIM	Resection of intracranial mass	19	20	814	2.46	0	0	5.88	1.29	0.41	2.51	18	0.00
GASTCA	Gastric cancer	17	256	8192	3.13	2.08	0	16.13	3.26	1.59	5.43	16	94.42
INGHERN	Inguinal hernia	17	71	11196	0.63	0	0	9.73	0.38	0	1.22	16	93.48
LAPAR	Laparotomy, not meeting other abdominal surg	15	340	2934	11.59	11.11	4.94	38.30	12.4	9.27	15.89	14	83.18
PAED	Pediatric surgical procedures, not otherwise sp	15	343	54212	0.63	2.37	0	62.22	6.07	3.91	8.64	14	98.77
CHOLE	Cholecystectomy	12	4	5816	0.07	0	0	0.15	0	0	0	11	0.00
UTRUP	Uterine rupture	15	87	1169	7.44	8.22	0	17.50	7.36	4.42	10.88	14	71.74
ESOCA	Esophageal carcinoma	12	133	1793	7.42	5.41	0	24.00	5.47	2.37	9.63	11	90.00
CCHD	Complex congenital heart disease	12	93	596	15.60	14.65	0	61.54	14.94	7.03	24.75	11	83.32
BOBS	Bowel obstruction	9	113	1095	10.32	8.33	2.27	28.57	10.17	5.63	15.75	8	85.00
ICH	Intracranial hemorrhage	10	224	1011	22.16	25.48	3.77	62.22	24.47	15.88	34.16	9	89.05
LIVTRAUM	Hepatic trauma	10	133	909	14.63	17.01	6.80	61.11	15.84	10.31	22.16	9	76.86
WHIP	Whipple pancreaticoduodenectomy	9	90	2060	4.37	3.62	0	9.92	3.68	2.28	5.36	8	57.76
MIS	Minimally invasive surgery, not otherwise spec	8	0	1266	0.00	0	0	0	0	0	0.06	7	0.00
RECTAL	Rectal resection	9	6	1032	0.58	0	0	5.88	0.07	0	0.92	8	50.09
SPINE	Spine surgery, excluding trauma	9	11	518	2.12	0	0	8.96	0.77	0	3.8	8	67.18
TIP	Typhoid intestinal perforation	9	134	662	20.24	20.73	4.55	33.33	20.09	14.36	26.48	8	71.46
AABDO	Acute abdomen but not meeting other abdomin	8	228	2877	7.92	10.42	4.90	34.88	11.2	7.42	15.62	7	86.27
ACHI	All-comer head injury	7	364	1360	26.76	16.16	10	54.58	25.33	12.8	40.29	6	96.77
BILD	Bile duct procedures, excluding Whipple proce	8	51	714	7.14	2.30	0	21.54	4.08	0.1	11.63	7	91.49
INTUSS	Intussusception	8	43	355	12.11	3.66	0	33.70	4.8	0.03	14.28	7	88.09
TAD	Thoracic aortic disease	8	775	4203	18.44	8.66	0	20.30	9.5	3.96	16.74	7	91.49
AAA	Abdominal aortic aneurysm	6	164	1673	9.80	10.94	8.50	50.00	11.11	7.44	15.34	5	72.08
CARDAN	Cardiac ventricular aneurysm	7	41	925	4.43	5.00	2.01	14.29	5.57	2.28	9.98	6	74.97
CARDIACMYX	Cardiac myxoma	7	17	500	3.40	4.17	0	5.41	2.24	0.34	5.22	6	53.25
CTRAUM	Cardiac trauma	7	53	285	18.60	18.27	0	23.29	17	12.46	21.99	6	0.00
LIVHYDAT	Hepatic hydatidosis	7	29	2050	1.41	0	0	2.82	0.46	0	1.54	6	57.80
ANEUR	Intra-cranial aneurysm	6	93	881	10.56	7.28	2.05	16.77	7.99	3.57	13.84	5	85.62
ENDOCARD	Endocarditis	6	124	471	26.33	23.01	15.52	38.71	25.23	15.53	36.27	5	79.98
HYDRO	Hydrocephalus	6	18	1067	1.69	2.51	0	4.17	1.6	0.22	3.8	5	65.10
PANC_RES	Pancreatic resection, excluding Whipple proce	6	5	288	1.74	1.32	0	4.23	0.77	0	3.17	5	29.35
PVI	Peripheral vascular injury	6	23	458	5.02	2.89	0	11.29	3.52	0.63	8.09	5	74.72
THORHYDAT	Thoracic hydatidosis	6	4	1375	0.29	0.10	0	1.15	0	0	0.08	5	0.00
VASCU	Vascular procedures, not otherwise specified	4	85	918	9.26	5.95	1.85	9.89	5.94	2.06	11.2	3	43.09
BRES	Bowel resection, not otherwise specified	5	12	192	6.25	1	0	31.82	4.92	0	17.39	4	79.25
FOURN	Fournier's gangrene	5	34	162	20.99	20	0	30.77	14.22	3.31	29.65	4	73.65
HIPFRAC	Hip fracture	5	108	1380	7.83	8.94	4.31	10.76	7.73	5.15	10.77	4	72.87
HYST	Hysterectomy, excluding emergency peripartu	4	3	403	0.74	0	0	4.17	0.28	0	2.23	3	59.25
NEURO	Neurosurgical procedures, not otherwise speci	5	78	2430	3.21	5.68	2.05	16.67	5.78	1.87	11.3	4	88.22
PROST	Prostatectomy	5	7	1050	0.67	1.01	0	1.75	0.4	0.04	1	4	0.00
SPLEEN	Splenic procedures	5	36	278	12.95	10.00	0	21.90	8.86	2.01	18.84	4	76.20
THORTRAUM	Thoracic trauma, not otherwise specified	5	38	343	11.08	10.19	6.67	15.38	10.07	6.83	13.77	4	0.00
VOLV	Colonic volvulus	5	39	811	4.81	10.42	0.84	14.63	6.39	1.84	13.03	4	86.25
ABDOTB	Abdominal tuberculosis	4	73	440	16.59	14.65	3.05	38.78	15.66	2.66	35.53	3	94.48
AWH	Abdominal wall hernia	3	3	1246	0.24	0.50	0	4.88	0.38	0	2.98	2	-
BYPASS	Peripheral arterial bypass	4	16	424	3.77	2.85	2.30	18.18	4.24	0.98	9.22	3	70.13
CDC	Choledochal cyst	4	3	1147	0.26	0.10	0	0.88	0	0	0.18	3	0.00
CDH	Congenital diaphragmatic hernia	4	16	65	24.62	32.67	0	42.86	21.72	1.56	52.18	3	82.21
GASTROSCH	Gastroschisis	4	113	455	24.84	27.64	10.29	62.96	29.68	10.75	53.14	3	96.11
GOITRE	Goitre	4	0	223	0.00	0	0	0	0	0	0.88	3	0.00
HYDAT	Hydatid disease, not otherwise specified	4	3	207	1.45	1.64	0	5.88	0.66	0	4.92	3	50.97
MCHD	Mixed (pediatric and adult) congenital heart di	4	2	695	0.29	0.96	0	2.5	0.19	0	2.04	3	60.36
RADCYS	Radical cystectomy	4	8	482	1.66	9.07	1	20	0.02	0	2.67	3	55.26
STRAUM	Spinal trauma	4	17	135	12.59	10.35	4.76	20.41	11.25	4.38	20.32	3	46.81
TRACHEOST	Tracheostomy	4	43	540	7.96	15.47	0	20	9.56	0.24	27.7	3	95.95
AMPUT	Amputation	3	55	382	14.40	16.03	4.69	16.67	12.6	6.61	20.1	2	-
ANOMAL	Anorectal malformation	3	3	115	2.61	2.50	2.17	3.45	2.54	0.14	6.75	2	-
BABS	Intracranial abscess	3	23	136	16.91	16.67	5.88	40.00	18.09	4.29	37.78	2	-
BURN	Burn injuries	2	25	293	8.53	7.82	1.54	14.11	7.19	4.45	10.49	1	-
CGSW	Cranial gunshot wound	3	70	575	12.17	13.33	10.63	19.32	13.52	8.18	19.89	2	-
CHD	Congenital heart disease, adult population	3	14	479	2.92	2.96	1.31	4.19	2.77	1.29	4.73	2	-
CLEFT	Cleft lip or palate surgery	3	0	292	0.00	0	0	0	0	0	0.68	2	-
DCL	Damage control laparotomy	3	45	375	12.00	11.18	5.56	26.92	13.05	4.78	24.11	2	-
DIABINF	Diabetic limb infection	3	25	205	12.20	13.79	5.56	15.05	11.54	6.27	18.04	2	-
EATEF	Esophageal atresia or tracheo-esophageal fistul	3	42	153	27.45	23.40	8.70	45	24.41	6.76	48.04	2	-
ECTOP	Ectopic pregnancy	3	0	200	0.00	0	0	0	0	0	0.95	2	-
ESPERF	Esophageal perforation	3	7	76	9.21	9.68	6.67	10.00	9.02	3.06	17.11	2	-
HIRSCH	Hirschprung's disease	2	28	135	20.74	18.91	16	21.82	20.44	13.87	27.85	1	-
IATRES	Intestinal atresia	3	17	54	31.48	33.33	21.74	40.91	30.95	18.71	44.53	2	-
LIVABS	Hepatic abscess	3	17	144	11.81	9.45	0	62.50	15.86	0	50.47	2	-
OBGYNE	Obstetric and gynecologic procedures, not oth	3	157	9747	1.61	0.83	0	1.78	0.8	0.13	1.92	2	-
ONCOL	Oncologic diagnoses, not otherwise specified	3	8	297	2.69	2.56	0	7.14	1.97	0.05	5.58	2	-
PTRAUM	Pancreatic trauma	3	56	275	20.36	21.00	11.54	23.33	19.87	15.21	24.95	2	-
TDH	Traumatic diaphragmatic hernia	3	7	64	10.94	11.11	0	13.79	9.59	2.77	18.96	2	-
THYROID	Thyroid surgery, excluding goitre	3	0	345	0.00	0	0	0	0	0	0.19	2	-
TRAUM	Trauma, not otherwise specified	3	9	263	3.42	2.88	0	4.72	2.93	0.99	5.62	2	-
UGIB	Upper gastrointestinal bleed	3	31	97	31.96	18.75	0	70.37	27.24	0.03	71.41	2	-
VVF	Vesicovaginal fistula	3	1	773	0.13	0	0	0.20	0	0	0.33	2	-
APR	Abdomino-perineal resection	2	1	50	2.00	1.39	0	2.78	1.33	0	7.88	1	-
ASO	Arterial Switch Operation	2	12	154	7.79	7.90	6.98	8.82	7.76	3.91	12.66	1	-
CBT	Carotid body tumor	2	0	75	0.00	0	0	0	0	0	2.3	1	-
CEA	Carotid endarterectomy	2	1	177	0.56	0.42	0.00	0.84	0.39	0	2.26	1	-
CIRCUM	Male circumcision	2	0	3204	0.00	0	0	0	0	0	0	1	-
EPIL	Epilepsy	2	2	402	0.50	2.91	0.26	5.56	0	0	0.38	1	-
GSW	Gunshot wound	2	11	76	14.47	14.55	13.95	15.15	14.45	7.16	23.52	1	-
HYPOS	Hypospadias	2	0	179	0.00	0	0	0	0	0	0.96	1	-
ICBX	Intracranial biopsy	2	3	252	1.19	1.60	0.57	2.63	0.96	0.02	2.76	1	-
MALRO	Malrotation	2	4	40	10.00	14.34	6.45	22.22	8.41	0.89	20.3	1	-
MYOMEC	Myomectomy for uterine fibroids	2	0	329	0.00	0	0	0	0	0	0.54	1	-
NEC	Necrotising enterocolitis	2	47	137	34.31	39.70	31.58	47.83	34	26.13	42.31	1	-
NECFASC	Necrotising fasciitis	2	11	129	8.53	7.18	5.26	9.09	7.91	3.51	13.58	1	-
NEPHREC	Nephrectomy	2	1	574	0.17	0.09	0	0.18	0	0	0.01	1	-
PALL	Palliative procedures for intra-abdominal malig	2	15	70	21.43	23.75	7.5	40	19.19	10.55	29.51	1	-
PANCRE	Pancreatitis	2	11	94	11.70	22.08	5.26	38.89	9.11	3.7	16.22	1	-
PECTUS	Pectus excavatum	2	0	528	0.00	0	0	0	0	0	0.29	1	-
PERICAR	Pericardiectomy	2	18	139	12.95	9.80	5.56	14.05	12.23	6.99	18.52	1	-
PTCATH	Peritoneal dialysis catheter placement	2	5	398	1.26	4.46	0	8.93	0.09	0	0.9	1	-
RELAPCS	Relaparotomy after caesarean section	2	9	50	18.00	18.27	11.54	25.00	17.54	7.81	29.74	1	-
RSS	Renal stone surgery	2	2	542	0.37	0.19	0	0.38	0	0	0	1	-
SDH	Subdural hemorrhage	2	50	330	15.15	14.38	11.20	17.56	15.01	11.32	19.1	1	-
TRACHSTEN	Tracheal stenosis	2	3	57	5.26	15.29	2	28.57	2.02	0	9.14	1	-
TTHR	Tumor thrombus secondary to renal or adrenal	2	2	19	10.53	7.14	0	14.29	8.11	0	27.57	1	-
ABDOPR	Abdominal pregnancy	1	0	9	0.00	0	0	0	0	0	33.63	0	-
AFIB	Atrial fibrillation	1	0	10	0.00	0	0	0	0	0	30.85	0	-
ASCAR	Ascariasis	1	1	79	1.27	1.27	1.27	1.27	1.27	0.03	6.85	0	-
BIAL	Bilateral internal iliac artery ligation	1	1	8	12.50	12.50	12.50	12.50	12.5	0.32	52.65	0	-
BILAT	Biliary atresia	1	2	15	13.33	13.33	13.33	13.33	13.33	1.66	40.46	0	-
BTL	Bilateral tubal ligation	1	0	78	0.00	0	0	0	0	0	4.62	0	-
CHOLESTEA	Cholesteatoma	1	1	61	1.64	1.64	1.64	1.64	1.64	0.04	8.8	0	-
CHYST	Ceasarean hysterectomy	1	2	28	7.14	7.14	7.14	7.14	7.14	0.88	23.5	0	-
CLE	Congenital lobar emphysema	1	4	26	15.38	15.38	15.38	15.38	15.38	4.36	34.87	0	-
COLOST	Colostomy, not otherwise specified	0											
CORANOM	Coronary vessel anomalies	1	1	9	11.11	11.11	11.11	11.11	11.11	0.28	48.25	0	-
CRANHYDAT	Intracranial hydatitosis	1	2	97	2.06	2.06	2.06	2.06	2.06	0.25	7.25	0	-
CROHN	Bowel resection for Crohn's disease	1	3	46	6.52	6.52	6.52	6.52	6.52	1.37	17.9	0	-
CRUPT	Cardiac rupture	1	6	19	31.58	31.58	31.58	31.58	31.58	12.58	56.55	0	-
CWR	Chest wall resection	1	0	19	0.00	0	0	0	0	0	17.65	0	-
DECORT	Decortication for empyema	1	31	206	15.05	15.05	15.05	15.05	15.05	10.46	20.68	0	-
DISC	Discectomy	1	1	95	1.05	1.05	1.05	1.05	1.05	0.03	5.73	0	-
DPSS	Placement of diaphragmatic pacer	1	0	11	0.00	0	0	0	0	0	28.49	0	-
ECF	Enterocutaneous fistula	1	0	22	0.00	0	0	0	0	0	15.44	0	-
EDH	Epidural hematoma	1	2	18	11.11	11.11	11.11	11.11	11.11	1.38	34.71	0	-
EGYNE	Emergency gynecologic surgery, excluding ect	1	6	105	5.71	5.71	5.71	5.71	5.71	2.13	12.02	0	-
EVISC	Evisceration	1	14	31	45.16	45.16	45.16	45.16	45.16	27.32	63.97	0	-
EXENT	Pelvic exenteration	1	2	59	3.39	3.39	3.39	3.39	3.39	0.41	11.71	0	-
FASC	Fasciotomy	1	8	70	11.43	11.43	11.43	11.43	11.43	5.07	21.28	0	-

FEME	Frontoethmoidal meningoencephalocele	1	4	200	2.00	2	2	2	2	0.55	5.04	0	-
FEMFRAC	Femur fracture	1	0	96	0.00	0	0	0	0	0	3.77	0	-
FIA	Fistula-in-ano	1	0	8	0.00	0	0	0	0	0	36.94	0	-
FORBOD	Airway foreign body	1	0	7	0.00	0	0	0	0	0	40.96	0	-
FTI	Flexor tendon injury	1	0	31	0.00	0	0	0	0	0	11.22	0	-
GAST	Gastric procedure, not otherwise specified	1	0	5	0.00	0	0	0	0	0	52.18	0	-
GASTROJ	Gastrojejunostomy, not otherwise specified	1	0	32	0.00	0	0	0	0	0	10.89	0	-
GENS	General surgery, not otherwise specified	1	6	100	6.00	6	6	6	6	2.23	12.6	0	-
HELLER	Heller myotomy	1	0	60	0.00	0	0	0	0	0	5.96	0	-
HEMOR	Hemorrhoidectomy	1	0	43	0.00	0	0	0	0	0	8.22	0	-
HNC	Head and neck cancer	1	2	184	1.09	1.09	1.09	1.09	1.09	0.13	3.87	0	-
ILEALC	Ileal conduit	1	0	16	0.00	0	0	0	0	0	20.59	0	-
ILEOST	Ileostomy, not otherwise specified	1	0	68	0.00	0	0	0	0	0	5.28	0	-
IPAA	Ileal pouch-anal anastomosis	1	2	49	4.08	4.08	4.08	4.08	4.08	0.5	13.98	0	-
LAPORCH	Laparoscopic orchidecomy	1	0	48	0.00	0	0	0	0	0	7.4	0	-
LIVAMOEB	Hepatic amoebiasis	1	6	16	37.50	37.50	37.50	37.50	37.5	15.2	64.57	0	-
LLU	Lower extremity ulcer, not otherwise specified	1	10	100	10.00	10	10	10	10	4.9	17.62	0	-
MASTOID	Mastoidectomy	1	0	6	0.00	0	0	0	0	0	45.93	0	-
MEDIAST	Mediastinitis	1	1	16	6.25	6.25	6.25	6.25	6.25	0.16	30.23	0	-
MEDMASS	Mediastinal mass	1	17	105	16.19	16.19	16.19	16.19	16.19	9.72	24.65	0	-
MOYA	Moyamoya disease	1	2	25	8.00	8	8	8	8	0.98	26.03	0	-
NECKDIS	Neck dissection	1	1	14	7.14	7.14	7.14	7.14	7.14	0.18	33.87	0	-
NECKMA	Neck mass	1	12	148	8.11	8.11	8.11	8.11	8.11	4.26	13.73	0	-
NEOPNEUMO	Pneumothorax in neonates	1	6	10	60.00	60	60	60	60	26.24	87.84	0	-
NISSN	Nissen fundoplication	1	0	25	0.00	0	0	0	0	0	13.72	0	-
NPCR	Nasopharyngeal carcinoma resection	1	0	18	0.00	0	0	0	0	0	18.53	0	-
OMD	Omphalomesenteric duct remnant	1	2	29	6.90	6.90	6.90	6.90	6.9	0.85	22.77	0	-
OMPHAL	Omphalocele	1	20	82	24.39	24.39	24.39	24.39	24.39	15.58	35.12	0	-
ORBTRAUM	Orbital trauma	1	1	9	11.11	11.11	11.11	11.11	11.11	0.28	48.25	0	-
OTRAUM	Orthopedic trauma, not otherwise specified	1	6	248	2.42	2.42	2.42	2.42	2.42	0.89	5.19	0	-
OVACA	Ovarian malignancy	1	0	60	0.00	0	0	0	0	0	5.96	0	-
PARATHYROID	Parathyroidectomy	1	0	43	0.00	0	0	0	0	0	8.22	0	-
PEMB	Pulmonary embolectomy	1	2	16	12.50	12.50	12.50	12.50	12.5	1.55	38.35	0	-
PLACENTAACC	Placenta accreta	1	1	41	2.44	2.44	2.44	2.44	2.44	0.06	12.86	0	-
PNECK	Penetrating neck trauma	1	11	98	11.22	11.22	11.22	11.22	11.22	5.74	19.2	0	-
PTM	Post-traumatic meningitis	1	19	52	36.54	36.54	36.54	36.54	36.54	23.62	51.04	0	-
PULC	Pressure ulcer	1	2	55	3.64	3.64	3.64	3.64	3.64	0.44	12.53	0	-
PYLORSTEN	Pyloric stenosis	1	0	63	0.00	0	0	0	0	0	5.69	0	-
RCFUN	Rhinocerebral fungal infection	1	12	28	42.86	42.86	42.86	42.86	42.86	24.46	62.82	0	-
RECTO	Rectopexy	1	0	12	0.00	0	0	0	0	0	26.46	0	-
RHEPAT	Ruptured hepatoma	1	1	20	5.00	5	5	5	5	0.13	24.87	0	-
ROHS	Reoperation after open heart surgery	1	24	282	8.51	8.51	8.51	8.51	8.51	5.53	12.4	0	-
SACRO	Sacrococcygeal teratoma	1	3	36	8.33	8.33	8.33	8.33	8.33	1.75	22.47	0	-
SKINCA	Dermatologic malignancy	1	6	154	3.90	3.90	3.90	3.90	3.9	1.44	8.29	0	-
STROKE	Neurosurgical intervention for stroke	1	4	20	20.00	20	20	20	20	5.73	43.66	0	-
THAL	Thal procedure	1	0	29	0.00	0	0	0	0	0	11.94	0	-
URETEROST	Ureterostomy	1	0	41	0.00	0	0	0	0	0	8.6	0	-
URETHRAL	Urethral stricture	1	0	91	0.00	0	0	0	0	0	3.97	0	-
UROL	Urological procedures, not otherwise specified	1	0	71	0.00	0	0	0	0	0	5.06	0	-
VALVEOBS	Reoperation for obstructed mechanical cardiac	1	23	129	17.83	17.83	17.83	17.83	17.83	11.65	25.54	0	-
VENOM	Surgery for snake evenomation	1	0	13	0.00	0	0	0	0	0	24.71	0	-
VULVCA	Vulvar carcinoma	1	0	11	0.00	0	0	0	0	0	28.49	0	-
XDRTB	Pulmonary resection for XDR-TB	1	0	5	0.00	0	0	0	0	0	52.18	0	-

	Score name	Number of papers (%)
General clinical descriptors	ASA	74 (7.5)
	NYHA status	62 (6.3)
	Oncologic stage	49 (5.0)
	Child-Pugh score	12 (12.1)
	Body Mass Index	9 (9.1)
	POSSUM	6 (6.1)
	Surgical APGAR score	5 (5.1)
	Karnofsky Performance Index	5 (5.1)
	Charlson Index	5 (5.1)
	WHO HIV stage	3 (3.0)
	CCS	3 (3.0)
Cardiac Surgery	EuroSCORE	30 (3.0)
	Parsonnet score	5 (5.1)
	ABC	4 (4.1)
	STS-EACTS	3 (3.0)
	EuroScoreII	3 (3.0)
Trauma	GCS	26 (2.6)
	Organ Injury Scale	17 (1.7)
	ISS	13 (1.3)
	Revised Trauma Score	4 (4.1)
General Surgery	Perforation-Operation Time Interval	4 (4.1)
Neurosurgery	Hunt-Hess scale	4 (4.1)
	WFNS	3 (3.0)
	Frankel grade	3 (3.0)

Table s5. Clinical risk scores. Abbreviations: *ASA*- American Society of Anesthesiologists Physical Status Classification System. *NYHA*- New York Heart Association Functional Classification. *Oncologic stage*- any cancer staging system. *POSSUM*- Physiological and Operative Severity Score for the enUmeration of Morbidity and mortality. *CCS*- Canadian Cardiovascular Society grading of angina pectoris. *ABC- Aristotle Basic Complexity Score. STS-EACTS*- Society of Thoracic Surgeons-European Association of CardioThoracic Surgeons Mortality Levels. *GCS- Glasgow Coma Scale. Organ Injury Scale*- American Association for the Surgery of Trauma Organ Injury Scale or Abbreviated Injury Scale. *ISS*- Injury Severity Score. *RTS*- Revised Trauma Score. *WFNS*- World Federation of Neurosurgical Societies subarachnoid hemorrhage scale.
