# Supplementary material for: Perioperative mortality rates in low-income and middle-income countries: a systematic review and meta-analysis
Source: BMJ Glob Health. 2018 Jun 22;3(3):e000810. doi: 10.1136/bmjgh-2018-000810 (PMC6035511; doi:10.1136/bmjgh-2018-000810)
Supplement: Supplementary file 4 [file bmjgh-2018-000810supp004.pdf]

Reference	First Author	Article Title	Specialty	Procedure or Diagnosis	High-risk population?	Year Published	Data midpoint year	Retrospective or prospective	Study design	Country	Type of Facilities Included	Urban/ Rural/ Mix	Was POMR clearly defined?	Timeframe of POMR	POMR numerator	POMR denominator	Overall POMR
1	Abalos, E.	Caesarean section surgical techniques (CORONIS): a fractional, factorial, unmasked, randomised controlled trial	OBGYN	CAES	N	2013	2009	P	audit	Multiple	Mixed	Mixed	Y	6W	16	15729	0.001
2	Abantanga, F.A.	The range of abdominal surgical emergencies in children older than 1 year at the Komfo Anokye Teaching Hospital, Kumasi, Ghana	PAEDS	LAPAR	N	2009	2003	P	audit	Ghana	AH	U	N	IP	93	955	0.097
3	Abasiattai, A.M.	Emergency peripartum hysterectomy in a tertiary hospital in southern Nigeria	OBGYN	EPH	N	2013	2007	R	audit	Nigeria	AH	U	N	IP	4	28	0.143
4	Abbaszadeh M	The impact of carotid artery disease on outcomes of patients undergoing coronary artery bypass grafting	CARDI	CABG	N	2011	2004	R	nonrandomized cohort	Iran, Islamic Rep.	AH	U	N	IP	14	1978	0.007
5	Abd El Maksoud, W.	Comparative study between Lichtenstein procedure and modified darn repair in treating primary inguinal hernia: a prospective randomized controlled trial	GENSX	INGHERN	N	2014	2008	P	RCT	Egypt, Arab Rep.	AH	U	Y	30D	0	227	0.000
6	Abdel Wahab, M	Caudate lobe resection: an Egyptian center experience	HEPAT	LIVRES	N	2009	2003	R	audit	Egypt, Arab Rep.	AH	U	N	IP	3	54	0.056
7	Abdel-Aleem H	Effectiveness of tranexamic acid on blood loss in patients undergoing elective cesarean section: randomized clinical trial.	OBGYN	CAES	N	2013	2011	P	RCT	Egypt, Arab Rep.	AH	U	Y	IP	0	740	0.000
8	Abdelmolaa, A.	Assessment of Endoscopic Assisted Microsurgery in Treatment of Cerebellopontine Angle Lesions	NEURO	RIM	N	2012	2009	P	nonrandomized cohort	Egypt, Arab Rep.	AH	U	N	IP	0	25	0.000
9	Abdel-Wahab, M.	Prognostic factors affecting survival and recurrence after hepatic resection for hepatocellular carcinoma in cirrhotic liver	HEPAT	LIVRES	Y	2010	2001	R	audit	Egypt, Arab Rep.	AH	U	Y	IP	16	175	0.091
10	Abdulrahman, R.	Outcome of Coronary Artery Bypass: Comparison between on Pump and off Pump	CARDI	CABG	N	2009	2007	R	audit	Iran, Islamic Rep.	AH	U	Y	IP	14	994	0.014
11	Abdur-Rahman, L.O.	Bowel resection in Nigerian children	PAEDS	AABDO	N	2009	2002	R	audit	Nigeria	AH	U	N	IP	12	70	0.171
12	Abdur-Rahman, L.O.	Pediatric day case surgery: Experience from a tertiary health institution in Nigeria	PAEDS	PAED	N	2009	2006	P	audit	Nigeria	AH	U	N	IP	0	449	0.000
13	Abdur-Rahman, L. O.	Circumcision: perspective in a Nigerian teaching hospital	PAEDS	CIRCUM	N	2013	2004	R	audit	Nigeria	AH	U	N	IP	0	204	0.000
14	Abid, D.	Congenital heart disease in 37,294 births in Tunisia: birth prevalence and mortality rate	CARDI	PCARD	N	2014	2010	R	audit	Tanzania	Mixed	U	N	IP	8	33	0.242
15	Abid, O.	Complications After Surgical Tracheostomy	ENT	TRACHEOST	N	2013	2012	P	nonrandomized cohort	Pakistan	Other	U	N	IP	0	250	0.000
16	Abrao FC	Desmoid tumors of the chest wall: surgical challenges and possible risk factors	THORA	CWR	N	2011	2003	R	audit	Brazil	AH	U	Y	30D_IP	0	19	0.000
17	Abubakar, A. M.	Challenges in the management of early versus late presenting congenital diaphragmatic hernia in a poor resource setting	PAEDS	CDH	N	2011	2007	P	audit	Nigeria	AH	U	N	30D	2	6	0.333
18	Adademir T	Surgical treatment of aortic valve endocarditis: a 26-year experience	CARDI	ENDOCARD	N	2014	1998	R	nonrandomized cohort	Turkey	AH	U	Y	IP	27	174	0.155
19	Ademuyiwa, A.	Non-trauma related paediatric abdominal surgical emergencies in Lagos, Nigeria: Epidemiology and indicators of survival	PAEDS	AABDO	N	2012	2009	R	audit	Nigeria	AH	U	N	IP	13	129	0.101
20	Ademuyiwa, A.O.	Determinants of mortality in neonatal intestinal obstruction in Ile Ife, Nigeria	PAEDS	BOBS	N	2009	2000	R	audit	Nigeria	AH	U	N	IP	18	63	0.286
21	Adenekan AT	Perioperative adverse airway events in cleft lip and palate repair	PLAST	CLEFT	N	2011	2007	P	nonrandomized cohort	Nigeria	DH	Mixed	N	IP	0	116	0.000
22	Adeoye, P.	Peripheral vascular surgical procedures in Ilorin, Nigeria: indications and outcome	VASCU	VASCU	N	2011	2007	Mixed R/P	audit	Nigeria	AH	U	N	IP	1	14	0.071
23	Adhikari, S.	Etiology and Outcome of Acute Intestinal Obstruction: A Review of 367 Patients in Eastern India	GENSX	BOBS	N	2010	2006	R	audit	India	AH	U	N	IP	23	288	0.080
24	Adigun, T.A.	Factors influencing the immediate postoperative outcome in operated head injured patients in Ibadan, Nigeria	NEURO	ACHI	N	2012	2010	P	audit	Nigeria	AH	U	N	IP	16	99	0.162
25	Adisa, A. O.	Laparoscopic appendectomy in a Nigerian teaching hospital	GENSX	APPY	N	2012	2010	R	nonrandomized cohort	Nigeria	AH	U	N	IP	0	139	0.000
26	Adisa, A.	An Audit of Laparoscopic Surgeries in Ile-Ife, Nigeria	GENSX	MIS	N	2011	2009	P	audit	Nigeria	AH	U	N	IP	0	62	0.000
27	Adisa AO	Laparascopic cholecystectomy in Ile-Ife, Nigeria	GENSX	CHOLE	N	2011	2010	P	audit	Nigeria	AH	U	N	IP	0	24	0.000
28	Adisa A	Local adaptations aid establishment of laparoscopic surgery in a semiurban Nigerian hospital	GENSX	MIS	N	2013	2010	R	nonrandomized cohort	Nigeria	AH	Mixed	N	IP	0	175	0.000
29	Adoga, A.A.	Indications and outcome of pediatric tracheostomy: results from a Nigerian tertiary hospital	PAEDS	TRACHEOST	N	2010	2004	R	audit	Nigeria	AH	U	N	IP	8	46	0.174
30	Afolayan, J.M.	Evolving pattern of spinal anaesthesia in stable eclamptic patients undergoing caesarean section at University of Benin Teaching Hospital, Benin, Nigeria	OBGYN	CAES	Y	2014	2011	R	nonrandomized cohort	Nigeria	AH	U	N	IP	10	82	0.122
31	Afuwape, O.	Preliminary Experience with Laparoscopic Cholecystectomy in a Nigerian Teaching Hospital	GENSX	CHOLE	N	2012	2010	R	audit	Nigeria	AH	U	N	IP	0	13	0.000
32	Agrawal D	Can glasgow score at discharge represent final outcome in severe head injury?	NEURO	ACHI	N	2012	2009	R	nonrandomized cohort	India	AH	U	Y	IP	149	273	0.546
33	Agu, K.	Prevalence, Morbidity, and Mortality Patterns of Typhoid Ileal Perforation as Seen at the University of Nigeria Teaching Hospital Enugu Nigeria: An 8-year Review	GENSX	TIP	N	2014	2003	R	audit	Nigeria	AH	R	N	IP	15	50	0.300
34	Aguiar, P.	Brainstem cavernomas: a surgical challenge	NEURO	RIM	N	2012	2003	R	audit	Brazil	AH	U	Y	30D	0	13	0.000
35	Aguilar-Nascimento J	Multimodal approach in colorrectal surgery without mechanical bowel cleansing	GENSX	COLRES	N	2009	2006	P	nonrandomized cohort	Brazil	AH	U	N	IP	2	53	0.038
36	Ahmad, M.	Safety Of Single Layer Continuous Extra Mucosal Gut Anastomosis In Emergency	GENSX	BRES	N	2012	2008	P	nonrandomized cohort	Pakistan	DH	U	N	IP	4	50	0.080
37	Ahmad, Nazir	Management of Strangulated Inguinal Hernia in Adults	GENSX	INGHERN	N	2014	2012	P	nonrandomized cohort	Pakistan	AH	U	N	IP	0	50	0.000
38	Ahmad, N.	Outcome of Splenic Injuries in Adult Trauma Population	TRAUM	SPLEEN	N	NA	2012	P	nonrandomized cohort	Pakistan	AH	U	N	IP	4	40	0.100
39	Ahmad, T.	Role of Urgent Thoracotomy in Improving the Survival of Patients with Severe Chest Trauma	TRAUM	THORTRAUM	N	2009	2006	P	nonrandomized cohort	Pakistan	AH	U	N	IP	8	52	0.154
40	Ahmed, A.A.	Perioperative cardiac arrests in children at a university teaching hospital of a developing country over 15 years	PAEDS	PAED	N	2009	1999	R	audit	Pakistan	AH	U	Y	PO	7	20216	0.000
41	Ahmed, A.	Emergency abdominal surgery in Zaria, Nigeria	GENSX	AABDO	N	2010	2003	R	audit	Nigeria	AH	U	Y	IP	107	1788	0.060
42	Ahmed A.	Management and outcome of gastric carcinoma in Zaria, Nigeria	GENSX	GASTCA	N	2011	2002	Mixed R/P	audit	Nigeria	AH	U	N	IP	25	155	0.161

Reference	First Author	Article Title	Specialty	Procedure or Diagnosis	High-risk population?	Year Published	Data midpoint year	Retrospective or prospective	Study design	Country	Type of Facilities Included	Urban/ Rural/ Mix	Was POMR clearly defined?	Timeframe of POMR	POMR numerator	POMR denomiator	Overall POMR
43	Ahmed, A.	Trends in emergency surgical admissions in a tertiary health centre in Nigeria	MULTI	MULTI	N	2009	1986	R	audit	Nigeria	AH	U	N	IP	129	2162	0.060
44	Ahmed A	Endovascular coiling versus surgical clipping in the treatment of ruptured anterior communicating artery aneurysm in Cairo University Hospitals	NEURO	ICH	N	2013	2010	P	nonrandomized cohort	Egypt, Arab Rep.	AH	U	N	IP	4	15	0.267
45	Aikawa P	Impact of coronary artery bypass grafting in elderly patients	CARDI	CABG	N	2013	2011	P	nonrandomized cohort	Brazil	DH	U	Y	IP	9	253	0.036
46	Aisuodionoe-Shadrach O	Outcome of the TURP-TUVP sandwich procedure for minimally invasive surgical treatment of benign prostatic hyperplasia with volume larger than 40cc over a 4-year period in Nigeria	UROLO	PROST	N	2013	2009	R	nonrandomized cohort	Nigeria	AH	U	N	IP	1	57	0.018
47	Ajami, G.H.	Coronary Artery Origin Anomalies with Especial Emphasis on Delayed Diagnosis of Anomalous Origin of the Left Coronary Artery from Pulmonary Artery, A report from South of Iran	CARDI	CORANOM	N	2009	2001	R	audit	Iran, Islamic Rep.	AH	U	Y	IP	1	9	0.111
48	Akaba, G.O.	Uterine Rupture: Trends and Feto-Maternal Outcome in a Nigerian Teaching Hospital	OBGYN	UTRUP	N	2013	2008	R	audit	Nigeria	AH	U	N	IP	9	73	0.123
49	Akar, A.R.	Validation of the EuroSCORE risk models in Turkish adult cardiac surgical population.	CARDI	CARD	N	2011	2007	R	audit	Turkey	Mixed	Mixed	Y	IP	157	8018	0.020
50	Akhtar, R.	Outcome of Double Vs. Single Valve Replacement For rheumatic heart disease	CARDI	VALVE	N	2011	2001	P	nonrandomized cohort	Pakistan	AH	U	Y	30D_IP	19	493	0.039
51	Akhtar, R.P.	Clinical Outcome of Double Valve Repalcement With or Without Small Aortic Prosthesis	CARDI	VALVE	N	2012	2002	P	nonrandomized cohort	Pakistan	AH	U	Y	30D_IP	6	140	0.043
52	Akinci, M.	Risk factors related with unfavorable outcomes in groin hernia repairs	GENSX	INGHERN	N	2010	2007	R	audit	Turkey	AH	U	N	IP	0	639	0.000
53	Akinci, M.	Association of ventral incisional hernias with comorbid diseases	GENSX	AWH	N	2013	2008	R	audit	Turkey	AH	U	N	IP	0	1005	0.000
54	Alaei F	Balloon Angioplasty versus Surgical Repair of Coarctation of Aorta in Infants	CARDI	PCARD	N	2011	2007	R	nonrandomized cohort	Iran, Islamic Rep.	AH	U	N	IP	13	112	0.116
55	Alatise OI	Surgical Outcome of Abdominoperineal Resection for Low Rectal Cancer in a Nigerian Tertiary Institution	GENSX	APR	N	2009	1998	R	audit	Nigeria	AH	Mixed	Y	30D_IP	1	36	0.028
56	Albsoul, N.M.	Hurthle cell carcinoma: Expanded view	ENT	THYROID	N	2009	2009	R	nonrandomized cohort	Jordan	AH	U	N	30D	0	56	0.000
57	Alemayehu W	Primary repair of obstetric uterine rupture can be safely undertaken by non-specialist clinicians in rural Ethiopia: a case series of 386 women	OBGYN	UTRUP	N	2013	2004	R	audit	Ethiopia	CH	R	Y	IP	19	386	0.049
58	Alexander, P.A.	The Pattern of Hydatid Disease—A Retrospective Study from Himachal Pradesh, India	GENSX	HYDAT	N	2010	2001	R	audit	India	DH	Mixed	Y	30D	0	115	0.000
59	Ali A	Role of Surgery in Stages II and III Pediatric Abdominal Non-Hodgkin Lymphoma: A 5-Years Experience	PAEDS	ONCOL	N	2011	2007	R	nonrandomized cohort	Egypt, Arab Rep.	AH	U	N	IP	0	35	0.000
60	Ali M	Short-term complications of ventriculoperitoneal shunt in children suffering from hydrocephlaus	NEURO	HYDRO	N	2009	2006	R	nonrandomized cohort	Pakistan	CH	Mixed	N	IP	2	86	0.023
61	Ali, N.	Appendicitis And Its Surgical Management Experience At The University Of Maiduguri Teaching Hospital Nigeria	GENSX	APPY	N	2012	2005	Mixed R/P	audit	Nigeria	AH	U	N	IP	12	1257	0.010
62	Ali N	Causes and treatment outcome of perforation peritonitis in north eastern Nigeriaash_506 92..96	GENSX	PERF	N	2010	2006	R	nonrandomized cohort	Nigeria	CH	U	N	IP	40	153	0.261
63	Ali, N.	Outcome of surgery for toxic goitres in maiduguri: A single teaching hospital's perspective	ENT	GOITRE	N	2011	2007	P	audit	Nigeria	AH	U	N	30D	0	78	0.000
64	Ali, R.	Pneumothoraces in a Neonatal Tertiary Care Unit: Case Series	THORA	NEOPNEUMO	N	2013	2010	R	audit	Pakistan	AH	U	Y	IP	6	10	0.600
65	Aliev, A.	Strategic aspects of stomach cancer surgery	GENSX	GASTCA	N	2011	2005	R	nonrandomized cohort	Georgia	AH	U	N	IP	5	90	0.056
66	Allam, I.S.	Incidence of emergency peripartum hysterectomy in Ain-shams University Maternity Hospital, Egypt: A retrospective study	OBGYN	EPH	N	2014	2008	R	audit	Egypt, Arab Rep.	AH	U	N	IP	5	149	0.034
67	Almeida, R.M.S.	Surgical left ventricular reverse remodeling: 111 months of follow-up	CARDI	CARDAN	N	2009	2002	R	audit	Brazil	AH	U	Y	30D	4	28	0.143
68	Al-Mogheer B	Predictors of inhospital mortality in patients with infective endocarditis	CARDI	ENDOCARD	N	2013	2006	R	nonrandomized cohort	Egypt, Arab Rep.	AH	U	Y	IP	60	155	0.387
69	Al-Qubati F	Incidence and outcome of pulmonary complications after open cardiac surgery, Thowra Hospital, Cardiac center, Sana'a, Yemen	CARDI	CARD	N	2013	2006	P	nonrandomized cohort	Yemen, Rep.	AH	U	N	30D_IP	5	179	0.028
70	Alsmady, M.M.	Early and Mid-term Evaluation of Mechanical Heart Valve Replacement	CARDI	VALVE	N	2010	2004	R	audit	Jordan	AH	U	Y	30D_IP	3	118	0.025
71	Alves, A.C.	Implementation of a colorectal laparoscopic surgical program - Short term outcomes and conversion rates	GENSX	COLRES	N	2013	2008	P	audit	Brazil	AH	U	Y	30D	4	215	0.019
72	Alves, A.P.R.	Long-Term Results of the Modified Thal Procedure in Patients with Chagasic Megaesophagus	GENSX	THAL	N	2014	1998	Mixed R/P	audit	Brazil	AH	U	N	IP	0	29	0.000
73	Alvi A	Fulminant amoebic colitis: a rare fierce presentation of a common pathology	GENSX	LAPAR	Y	2013	2002	R	nonrandomized cohort	Pakistan	AH	U	N	IP	8	19	0.421
74	Alvi, A.R.	Delayed surgical therapy reduces mortality in patients with acute necrotizing pancreatitis	HEPAT	PANCRE	N	2011	2003	R	nonrandomized cohort	Pakistan	AH	U	Y	IP	7	18	0.389
75	Alvi, A.R.	Impact Of Rural Medical Care In Remote Mountainous Region Of Pakistan: Challenges And Opportunities	MULTI	MULTI	N	2009	1999	P	audit	Pakistan	Other	R	Y	IP	21	1990	0.011
76	AlWaqfi, N.	Coronary artery bypass: predictors of 30-day operative mortality in Jordanians	CARDI	CABG	N	2012	2007	R	audit	Jordan	AH	U	Y	30D	62	1046	0.059
77	Amaral F	Adult congenital heart disease: experience with the surgical approach	CARDI	CHD	N	2013	1990	R	nonrandomized cohort	Brazil	AH	U	Y	IP_30D	8	191	0.042
78	Ameh, E.	Challenges Of Managing Paediatric Abdominal Traumain A Nigerian Setting	PAEDS	LAPAR	N	2009	1996	R	audit	Nigeria	AH	U	N	IP	9	79	0.114
79	Aminmansour B.	Effect of decompressive hemicraniectomy on mortality of malignant middle cerebral artery infarction	NEURO	STROKE	N	2010	2008	P	audit	Iran, Islamic Rep.	AH	U	Y	30D	4	20	0.200
80	Amir, D.	Frequency of complications due to laparoscopic cholecystectomy in Hamedan Hospitals	GENSX	CHOLE	N	2012	2001	R	audit	Iran, Islamic Rep.	Mixed	U	N	IP	0	426	0.000
81	Amponsah, G.	Challenges of anaesthesia in the management of the surgical neonates in Africa	PAEDS	PAED	N	2010	2009	R	audit	Ghana	AH	U	Y	IP	54	190	0.284

Reference	First Author	Article Title	Specialty	Procedure or Diagnosis	High-risk population?	Year Published	Data midpoint year	Retrospective or prospective	Study design	Country	Type of Facilities Included	Urban/ Rural/ Mix	Was POMR clearly defined?	Timeframe of POMR	POMR numerator	POMR denominator	Overall POMR
82	Amudhan, A.	Management of Esophageal Perforation: Experience from a Tertiary Center in India	THORA	ESPERF	N	2009	2003	R	audit	India	AH	U	Y	30D	3	31	0.097
83	Andrade I	Use of EuroSCORE as a predictor of morbidity after cardiac surgery	CARDI	CARD	N	2014	2008	R	nonrandomized cohort	Brazil	CH	U	Y	IP	37	900	0.041
84	Andrade, I.N.G.	Assessment of the EuroSCORE as a predictor for mortality in valve cardiac surgery at the Heart Institute of Pernambuco	CARDI	VALVE	N	2010	2005	R	audit	Brazil	AH	U	Y	IP	66	840	0.079
85	Andreollo N	Roux-En-Y Gastroenteroanastomosis In The Treatment Of Stenosing And Advanced Gastric Adenocarcinoma	GENSX	GASTCA	N	2010	2003	R	nonrandomized cohort	Brazil	AH	U	N	IP	1	62	0.016
86	Andreollo N	Neoadjuvant Chemoradiotherapy And Surgery Compared With Surgery Alone In Squamous Cell Carcinoma Of The Esophagus	THORA	ESOCA	N	2013	1992	R	nonrandomized cohort	Brazil	AH	U	N	IP	14	123	0.114
87	Andrews NB	Neurosurgical procedures in Jehovah's Witnesses: The Tema experience	NEURO	NEURO	N	2009	2003	R	audit	Ghana	DH	U	N	IP	1	21	0.048
88	Ansar, A.	Hysterectomy as a management option for morbidly adherent placenta	OBGYN	CHYST	N	2014	2009	P	nonrandomized cohort	Pakistan	AH	U	N	IP	2	28	0.071
89	Anwar ul Haq, U	Factors Affecting Survival in Patients with Oesophageal Atresia and Traceho-oesophageal Fistula	PAEDS	EATEF	N	2009	2004	R	audit	Pakistan	AH	U	N	IP	27	60	0.450
90	Arantes A	A new expansive two-open-doors laminoplasty for multilevel cervical spondylotic myelopathy: for multilevel cervical spondylotic myelopathy:	NEURO	SPINE	N	2012	2003	P	nonrandomized cohort	Brazil	Mixed	U	N	IP	0	80	0.000
91	Aras, M.	Being a neighbor to Syria: A retrospective analysis of patients brought to our clinic for cranial gunshot wounds in the Syrian civil war	NEURO	CGSW	N	2014	2012	R	audit	Turkey	AH	U	N	IP	17	88	0.193
92	Araujo, S.	Surgical outcomes after preceptored laparoscopic colorectal surgery: Results of a Brazilian preceptorship program	GENSX	COLRES	N	2009	2007	P	case-control	Brazil	AH	U	N	IP	0	60	0.000
93	Archana, K.	A clinical review of emergency obstetric hysterectomy	OBGYN	EPH	N	2009	2003	R	audit	India	AH	U	N	IP	6	112	0.054
94	Ardeshiri, M.	Effect of Obesity on Mortality and Morbidity After Coronary Artery Bypass Grafting Surgery in Iranian Patients	CARDI	CABG	N	2014	2010	P	audit	Iran, Islamic Rep.	AH	U	Y	30D	3	235	0.013
95	Arinc, S.	Evaluation of pulmonary hydatid cyst cases	THORA	THORHYDAT	N	2009	2004	R	audit	Turkey	AH	U	N	IP	1	138	0.007
96	Arnold, M.	Long-term outcome of surgically managed necrotizing enterocolitis in a developing country	PAEDS	NEC	N	2010	1993	R	audit	South Africa	AH	U	Y	30D	36	114	0.316
97	Arveen, S.	Perforated Peptic Ulcer in South India: An Institutional Perspective	GENSX	PERF	N	2009	2007	P	audit	India	AH	U	Y	IP	28	328	0.085
98	Asadi-Pooya, A.A.	Corpus callosotomy is a valuable therapeutic option for patients with Lennox–Gastaut syndrome and medically refractory seizures	NEURO	EPIL	N	2013	2010	R	audit	Iran, Islamic Rep.	AH	U	Y	30D_IP	1	18	0.056
99	Asanin, B.	Traumatic epidural hematomas in posterior cranial fossa	NEURO	EDH	N	2009	1995	R	audit	Montenegro	AH	U	N	IP	2	18	0.111
100	Asefa, Z.	Perforated peptic ulcer disease in Zewditu Hospital	GENSX	PERF	N	2012	2007	R	audit	Ethiopia	AH	U	N	IP	12	76	0.158
101	Ashfaq, A.	Is early correction of congenital ventricular septal defect a better option in a developing country?	CARDI	PCARD	N	2010	2007	R	audit	Pakistan	DH	U	N	IP	2	39	0.051
102	Ashimi, A.	A prospective surveillance of ruptured uterus in a rural tertiary health facility in northwest Nigeria	OBGYN	UTRUP	N	2013	2011	P	nonrandomized cohort	Nigeria	DH	R	N	IP	0	41	0.000
103	Asicioglu, O.	Second-stage vs first-stage caesarean delivery: Comparison of maternal and perinatal outcomes	OBGYN	CAES	N	2014	2009	R	audit	Turkey	AH	U	N	IP	1	3817	0.000
104	Asicioglu, O.	Maternal and perinatal outcomes in women with placenta praevia and accreta in teaching hospitals in Western Turkey	OBGYN	CAES	Y	2014	2007	R	audit	Turkey	DH	U	N	IP	1	318	0.003
105	Aslam, V.	Transhiatal oesophagectomy	THORA	ESOCA	N	2009	2006	P	nonrandomized cohort	Pakistan	AH	U	Y	30D	1	50	0.020
106	Aslar, A. K.	Analysis of 230 Cases of Emergent Surgery for Obstructing Colon Cancer—Lessons Learned	GENSX	COLRES	N	2010	1999	P	audit	Turkey	AH	U	Y	30D_IP	29	230	0.126
107	Atik F	Results of the establishment of an organizational model in a cardiovascular surgery service	CARDI	CARD	N	2009	2006	R	nonrandomized cohort	Brazil	AH	U	Y	30D_IP	27	367	0.074
108	Avila, W.S.	Maternal-Fetal Outcome and Prognosis of Cardiac Surgery During Pregnancy	CARDI	CARD	Y	2009	1996	R	audit	Brazil	AH	U	N	IP	3	41	0.073
109	Awan, M.S.	Minicholecystectomy versus laproscopic cholecystectomy	GENSX	CHOLE	N	2011	2008	P	nonrandomized cohort	Pakistan	Mixed	U	N	30D	0	68	0.000
110	Aydin E	Comparison of superior septal approach with left atriotomy in mitral valve surgery	CARDI	VALVE	N	2014	2011	P	RCT	Turkey	AH	U	N	IP	6	91	0.066
111	Azarfarin, R.	Off-Pump Coronary Artery Bypass Surgery in Severe Left Ventricular Dysfunction	CARDI	CABG	N	2010	2007	R	nonrandomized cohort	Iran, Islamic Rep.	AH	U	Y	IP	24	689	0.035
112	Azeredo L	Late outcome analysis of the Braile Biomédica® pericardial valve in the aortic position	CARDI	VALVE	N	2014	2008	R	nonrandomized cohort	Brazil	AH	U	Y	IP	16	196	0.082
113	Badejoko O	Operative Gynecologic Laparoscopy in Ile-Ife, Nigeria: Preliminary Experience	OBGYN	MIS	N	2013	2012	P	nonrandomized cohort	Nigeria	AH	U	N	IP	0	51	0.000
114	Badejoko, O.	Obstetric hysterectomy: Trend and outcome in Ile-Ife, Nigeria	OBGYN	EPH	N	2013	2005	R	audit	Nigeria	AH	R	N	IP	10	55	0.182
115	Badrinath R	Outcomes and unmet need for neonatal surgery in a resource-limited environment: Estimates of global health disparities from Kampala, Uganda	PAEDS	PAED	N	2014	2012	P	nonrandomized cohort	Uganda	DH	U	Y	IP	11	88	0.125
116	Bagheri, J	Impacts of non-dialysis-dependent renal insufficiency on the early surgical outcomes after isolated coronary artery bypass graft surgery	CARDI	CABG	N	2013	2009	R	nonrandomized cohort	Iran, Islamic Rep.	AH	U	Y	30D	48	1359	0.035
117	Bagheri, J.	Effects of body mass index on the early surgical outcomes after coronary artery bypass grafting	CARDI	CABG	N	2014	2009	R	case-control	Iran, Islamic Rep.	AH	U	Y	30D	32	1673	0.019
118	Bagheri, J.	Effect of sex on early surgical outcomes of isolated coronary artery bypass grafting	CARDI	CABG	N	2014	2009	R	nonrandomized cohort	Iran, Islamic Rep.	AH	U	Y	30D	50	1390	0.036
119	Bagheri, R.	Outcome of repair of bronchial injury in 10 patients with blunt chest trauma	TRAUM	THORTRAUM	N	2014	2006	R	audit	Iran, Islamic Rep.	Mixed	U	Y	30D	1	10	0.100
120	Bagheri, R.	Pulmonary hydatid cyst: Analysis of 1024 cases	THORA	THORHYDAT	N	2011	1994	R	audit	Iran, Islamic Rep.	AH	U	N	IP	2	1024	0.002
121	Bagheri, R.	Surgical Management of Bronchiectasis: Analysis of 277 Patients	THORA	LUNGRES	N	2010	1996	R	audit	Iran, Islamic Rep.	AH	U	N	IP	2	277	0.007

Reference	First Author	Article Title	Specialty	Procedure or Diagnosis	High-risk population?	Year Published	Data midpoint year	Retrospective or prospective	Study design	Country	Type of Facilities Included	Urban/ Rural/ Mix	Was POMR clearly defined?	Timeframe of POMR	POMR numerator	POMR denomiator	Overall POMR
122	Bahar, I.	Open Heart Surgery in Patients with End-Stage Renal Failure: Fifteen-Year Experience	CARDI	CARD	N	2009	1998	R	audit	Turkey	AH	U	Y	30D_IP	787	16425	0.048
123	Bahebeck, J.	Limb-threatening and life-threatening diabetic extremities: clinical patterns and outcomes in 56 patients.	VASCU	DIABINF	N	2010	2007	P	audit	Cameroon	AH	U	N	IP	3	54	0.056
124	Baig, A.	Pyogenic Liver Abscess: A Five Year Retrospective Study in Slums of Karachi	HEPAT	LIVABS	N	2012	2006	R	audit	Pakistan	AH	U	N	IP	0	9	0.000
125	Balci, A.E.	Current Surgical Therapy for Bronchiectasis: SurgicalResultsandPredictive Factorsin86Patients	THORA	LUNGRES	N	2014	2006	R	audit	Turkey	AH	U	Y	30D_IP	1	86	0.012
126	Balderrabano-Saucedo, N	Pentalogy of Cantrell: Forty-two Years of Experience in the Hospital Infantil de Mexico Federico Go´mez	CARDI	CCHD	N	2011	1988	R	audit	Mexico	AH	U	N	IP	8	13	0.615
127	Bali, R.S.	Perforation peritonitis and the developing world	GENSX	PERF	N	2014	2011	R	audit	India	AH	U	N	IP	28	400	0.070
128	Bandre, E.	Hirschsprung's disease: Management problem in a developing country	PAEDS	HIRSCH	N	2010	2004	R	audit	Burkina Faso	AH	U	N	IP	4	25	0.160
129	Bani, H.M.N	Jejunal Disorders: Potentially Lethal Causes of Acute Abdomen Are Still Overlooked	GENSX	BRES	N	2009	2004	R	audit	Jordan	AH	U	N	IP	0	6	0.000
130	Banieghbal B	Minimally Invasive Surgery for Children with HIV/AIDS	GENSX	MIS	Y	2009	2004	R	nonrandomized cohort	South Africa	AH	U	N	IP	2	48	0.042
131	Banu T	Bringing Surgery to Rural Children: Chittagong, Bangladesh Experience	PAEDS	PAED	N	2013	2010	P	nonrandomized cohort	Bangladesh	Mixed	Mixed	N	IP	0	407	0.000
132	Barati, M.	Ten-year experience of rhinocerebral zygomycosis in a teaching hospital in Tehran	ENT	RCFUN	N	2010	2002	R	audit	Iran, Islamic Rep.	AH	U	Y	30D	12	28	0.429
133	Barnard, B.J.	Mitral valve replacement at Tygerberg Hospital: a 5 year follow-up	CARDI	VALVE	N	2010	2000	R	audit	South Africa	AH	U	Y	30D	9	160	0.056
134	Bartos, A.	Multi-organ resections for colorectal cancer: analysis of potential factors with role in the occurrence of postoperative complications and deaths	GENSX	COLRES	Y	2012	2008	R	nonrandomized cohort	Romania	AH	U	Y	30D	68	1821	0.037
135	Batajoo, H.	Laparoscopic versus open appendectomy in acute appendicitis	GENSX	APPY	N	2012	2010	R	nonrandomized cohort	Nepal	AH	U	N	IP	0	226	0.000
136	Beard, J.H.	Surgical Task-Shifting in a Low-Resource Setting: Outcomes After Major Surgery Performed by Nonphysician Clinicians in Tanzania	MULTI	MULTI	N	2014	2012	R	nonrandomized cohort	Tanzania	Mixed	Mixed	Y	IP	27	1667	0.016
137	Beerdawood, K.A.	Primary colorectal anastamosis, no preparation, no stoma needed	GENSX	COLRES	N	2014	2010	P	nonrandomized cohort	Jordan	AH	U	N	IP	0	130	0.000
138	Bekele, S.	Laparoscopic cholecystectomy at Myungsung Christian Medical Center, Ethiopia: A five-years experience	GENSX	CHOLE	N	2012	2007	R	audit	Ethiopia	Other	U	N	IP	1	681	0.001
139	Benjacholmas, V.	Short-term outcome of PDA ligation in the preterm indatns at king Chulalongkorn Memorial Hospital Thailand	CARDI	PCARD	N	2009	2002	R	audit	Thailand	AH	U	Y	IP	2	42	0.048
140	Benzagmout, M.	Pott's disease in children	NEURO	SPINE	N	2011	2003	R	audit	Morocco	AH	U	Y	30D	0	7	0.000
141	Beudeker N	The hidden mortality of imperforate anus	PAEDS	ANOMAL	N	2013	2007	R	audit	Malawi	AH	U	N	IP	1	46	0.022
142	Bezircioglu, I.	Do clinical and laboratory parameters effect maternal and fetal outcomes in pregnancies complicated with hemolysis, elevated liver enzymes, and low platelet count syndrome?	OBGYN	CAES	Y	2012	2006	R	audit	Turkey	AH	U	N	IP	0	40	0.000
143	Bhaijee, F.	Fibrolamellar hepatocellular carcinoma at a tertiary centre in South Africa	HEPAT	LIVRES	N	2009	1999	R	audit	South Africa	AH	U	Y	30D	0	7	0.000
144	Bhandarwar, A. H.	Surgical response to the 2008 Mumbai terror attack	TRAUM	TRAUM	N	2012	2008	P	audit	India	DH	U	N	IP	6	127	0.047
145	Bhatti, K., et al.	A clinic-pathologic correlation of elective abdominal hysterectomy at teaching hospital Khairpur, Pakistan	OBGYN	HYST	N	2013	2010	P	audit	Pakistan	AH	U	N	IP	0	150	0.000
146	Bi, Y	Safety and Survival Benefit of Surgical Management for Elderly Gastric Cancer Patients	GENSX	GASTCA	N	2014	2007	R	audit	China	AH	U	Y	30D_IP	1	48	0.021
147	Biccard, B.M.	Factors associated with mortality when chronic beta- blocker therapy is withdrawn in the peri-operative period in vascular surgical patients: a matched case–control study	VASCU	VASCU	N	2010	2005	R	case-control	South Africa	AH	U	Y	IP	82	829	0.099
148	Biluts, H.	In-patient surgical mortality In Tikur Anbessa hospital: a five-year review	MULTI	MULTI	N	2009	2004	R	audit	Ethiopia	AH	U	Y	IP	443	9860	0.045
149	Biro, G.	Cryopreserved homograft and autologous deep vein replacement for infrarenal aorto and iliaco-femoral graft infection: early and late results	VASCU	BYPASS	N	2011	2001	R	nonrandomized cohort	Hungary	AH	U	Y	IP_30D	6	33	0.182
150	Borracci R	Prospective validation of EuroSCORE II in patients undergoing cardiac surgery in Argentinean centres	CARDI	CARD	N	2014	2012	P	nonrandomized cohort	Argentina	Mixed	Mixed	Y	IP	21	503	0.042
151	Botianu, P.V.H.	Anatomo-Clinical Analysis of 14 Consecutive Cases of Primary Cystic Mesenterico-Epiploic Tumors	GENSX	BRES	N	2014	2004	R	audit	Romania	AH	U	N	IP	0	14	0.000
152	Bouassida, M.	Histopathologic characteristics and short-term outcomes of colorectal cancer in young Tunisian patients: one center's experience	GENSX	COLRES	N	2012	2005	R	audit	Tunisia	AH	U	N	IP	22	280	0.079
153	Breda, J.R.	Surgical biatrial ablation of atrial fibrillation: initial results	CARDI	VALVE	N	2010	2008	P	audit	Brazil	AH	U	Y	IP	0	15	0.000
154	Briand, V.	Maternal and Perinatal Outcomes by Mode of Delivery in Senegal and Mali: A Cross-Sectional Epidemiological Survey	OBGYN	CAES	N	2012	2008	P	nonrandomized cohort	Multiple	DH	Mixed	Y	IP	157	11255	0.014
155	Bukar M	Caesarean delivery at the Federal Medical Centre Gombe: A 3-year experience	OBGYN	CAES	N	2009	2002	R	audit	Nigeria	DH	U	N	IP	2	250	0.008
156	Bunchungmongkol, N	Anesthesia-related cardiac arrest in children: the thai anesthesia incidents study (THAI study)	PAEDS	PAED	N	2009	2003	P	audit	Thailand	Mixed	Mixed	Y	24H	40	25098	0.002
157	Cai, X.J.	Laparoscopic left hemihepatectomy: a safety and feasibility study of 19 cases	HEPAT	LIVRES	N	2009	2006	R	case-control	China	AH	U	N	IP	0	38	0.000
158	Caliskan, A.C.	Emergency peripartum hysterectomy: a 4-year review	OBGYN	EPH	N	2010	2002	R	audit	Turkey	AH	U	N	IP	6	62	0.097
159	Camille, A.	Advantages of early management of facial clefts in Africa	PLAST	CLEFT	N	2014	2005	R	audit	Cote d'Ivoire	Mixed	Mixed	N	IP	0	70	0.000
160	Campos F	Locally Advanced Colorectal Cancer:results of surgical treatment and prognostic factors	GENSX	COLRES	N	2011	2002	P	nonrandomized cohort	Brazil	AH	U	N	IP	3	90	0.033

Reference	First Author	Article Title	Specialty	Procedure or Diagnosis	High-risk population?	Year Published	Data midpoint year	Retrospective or prospective	Study design	Country	Type of Facilities Included	Urban/ Rural/ Mix	Was POMR clearly defined?	Timeframe of POMR	POMR numerator	POMR denominator	Overall POMR
161	Campos F	Surgical treatment of familial adenomatous polyposis: ileoretal anastomosis or restorative proctolectomy?	GENSX	COLRES	N	2009	1996	R	nonrandomized cohort	Brazil	AH	U	N	30D	0	88	0.000
162	Campos, FG	Evolution of Laparoscopic Colorectal Surgery in Brazil *Results of 4744 Patients From the National Registry*	GENSX	COLRES	N	2009	1999	R	audit	Brazil	Mixed	Mixed	N	IP	43	4744	0.009
163	Caputti GM	Off-pump coronary artery bypass surgery in selected patients is superior to the conventional approach for patients with severely depressed left ventricular function	CARDI	CABG	Y	2011	2003	R	nonrandomized cohort	Brazil	AH	U	Y	IP	18	217	0.083
164	Caronna, R.	Comparative analysis of primary repair vs resection and anastomosis, with laparostomy, in management of typhoid intestinal perforation: results of a rural hospital in northwestern Benin	GENSX	TIP	N	2013	2011	R	nonrandomized cohort	Benin	DH	R	N	IP	26	78	0.333
165	Carosella, V.C.	The first Latin-American risk stratification system for cardiac surgery: can be used as a graphic pocket-card score	CARDI	CARD	N	2009	2000	R	audit	Argentina	AH	U	Y	IP	281	3990	0.070
166	Casarim, A.L.M.	Carotid body tumor: retrospective analysis on 22 patients	ENT	CBT	N	2014	1996	R	audit	Brazil	AH	U	Y	30D	0	22	0.000
167	Cawich S	Is emergent laparoscopic cholecystectomy for acute cholecystitis safe in a low volume resource poor setting?	GENSX	CHOLE	N	2014	2010	R	nonrandomized cohort	Multiple	Mixed	Mixed	N	IP	0	74	0.000
168	Caylak, H.	Surgical Management of Bronchiectasis: A Collective review of 339 patients with long-term follow-up	THORA	LUNGRES	N	2011	2000	R	audit	Turkey	AH	U	Y	90D	2	339	0.006
169	Celiku, E.	Rectal cancer surgery.A ten years experience	GENSX	RECTAL	N	2010	2004	R	audit	Albania	AH	U	Y	IP	4	148	0.027
170	Cetin, G.	Single-Institutional 22 Years Experience on Cardiac Myxomas	CARDI	CARDIACMYX	N	2010	1996	R	audit	Turkey	AH	U	Y	IP	1	24	0.042
171	Chagas J	Multicentricity in the thyroid differentiated carcinoma	ENT	THYROID	N	2009	2002	R	nonrandomized cohort	Brazil	AH	U	N	IP	0	27	0.000
172	Chalya, P.L.	Dermatological malignancies at a Univeristy Teaching Hospital in North Western Tanzania: A retrospective review of 154 Cases	GENSX	SKINCA	N	2012	2008	R	audit	Tanzania	Mixed	U	Y	IP	6	154	0.039
173	Chalya, P.L.	Etiological spectrum and treatment outcome of Obstructive jaundice at a University teaching Hospital in northwestern Tanzania: A diagnostic and therapeutic challenges	HEPAT	BILD	N	2011	2008	P	audit	Tanzania	AH	U	Y	IP	18	110	0.164
174	Chalya, P.	Major limb amputations: A tertiary hospital experience in northwestern Tanzania	ORTHO	AMPUT	N	2012	2009	P	audit	Tanzania	AH	U	N	IP	27	162	0.167
175	Chalya, P.	Splenic injuries at Bugando Medical Centre in northwestern Tanzania: a tertiary hospital experience	TRAUM	SPLEEN	N	2012	2010	P	audit	Tanzania	AH	U	N	IP	23	105	0.219
176	Chalya, P.	Typhoid intestinal perforations at a University teaching hospital in Northwestern Tanzania: A surgical experience of 104 cases in a resource-limited setting	GENSX	TIP	N	2012	2009	Mixed R/P	audit	Tanzania	AH	U	N	IP	24	104	0.231
177	Chalya, P.L.	Clinical profile and outcome of surgical treatment of perforated peptic ulcers in Northwestern Tanzania: A tertiary hospital experience	GENSX	PERF	N	2011	2008	Mixed R/P	audit	Tanzania	AH	U	N	IP	9	84	0.107
178	Chalya, P.	Clinicopathological profile and surgical treatment of abdominal tuberculosis: a single centre experience in northwestern Tanzania	GENSX	ABDOTB	N	2013	2009	P	audit	Tanzania	AH	U	N	IP	48	212	0.226
179	Chalya, P.L.	Enterocutaneous fistula: a Tanzanian experience in a tertiary care hospital	GENSX	ECF	N	2010	2008	P	audit	Tanzania	AH	U	N	IP	0	22	0.000
180	Chamisa I	Pattern of civilian gunshot wounds in Durban, South Africa	TRAUM	GSW	N	2011	2006	P	nonrandomized cohort	South Africa	AH	U	N	IP	5	33	0.152
181	Chan, R.P.C.	Intensive Perioperative Glucose Control Does Not Improve Outcomes Of Patients Submitted To Open-Heart Surgery: A Randomized Controlled Trial	CARDI	CARD	N	2009	2008	P	RCT	Brazil	AH	U	Y	30D	5	109	0.046
182	Chander, J.	Laparoscopic Choledochoduodenostomy for Biliary Stone Disease: A Single-Center 10-Year Experience	HEPAT	BILD	N	2012	2006	P	audit	India	AH	U	N	IP	0	27	0.000
183	Chandra D	Surgical management of anomalous pulmonary venous connection to the superior vena cava - early results	CARDI	CARD	Y	2013	2012	P	nonrandomized cohort	India	AH	U	Y	IP	0	7	0.000
184	Chao, T.E.	Surgical Care in Liberia and Implications for Capacity Building	MULTI	MULTI	N	2014	2012	R	audit	Liberia	AH	U	Y	IP_30D	43	584	0.074
185	Chaudhary, R.	Influence of comorbidity in cancer surgery on treatment decisions, postoperative course and oncological outcome	GENSX	ONCOL	N	2013	2009	P	audit	India	AH	U	Y	30D	6	234	0.026
186	Chau-in W	Anesthesia-related complications of caesarean delivery in Thailand: 16,697 cases from the Thai anaesthesia incidents study	OBGYN	CAES	N	2010	2003	P	audit	Thailand	Mixed	Mixed	Y	24H	8	16697	0.000
187	Chen, B.	Modified McKeown Minimally Invasive Esophagectomy for Esophageal Cancer: A 5-Year Retrospective Study of 142 Patients in a Single Institution	THORA	ESOCA	N	2013	2009	R	audit	China	AH	U	Y	30D_IP	1	142	0.007
188	Chen, M.	Peripartum hysterectomy between 2009 and 2010 in Sichuan, China	OBGYN	EPH	N	2013	2009	P	audit	China	Mixed	Mixed	N	IP	1	64	0.016
189	Chen, Q.	Surgical treatment for pulmonary aspergilloma: a 35-year experience in the Chinese population	THORA	LUNGRES	N	2012	1992	R	audit	China	AH	U	Y	30D	3	256	0.012
190	Chen, X.	The stented elephant trunk procedure combined total arch replacement for Debakey I aortic dissection: operative result and follow-up	CARDI	TAD	N	2010	2006	R	audit	China	AH	U	Y	IP	4	28	0.143
191	Chen X	Comparison of short-term outcomes and perioperative systemic immunity of laparoscopy-assisted and open radical gastrectomy for gastric cancer	GENSX	GASTCA	N	2011	2011	R	nonrandomized cohort	China	AH	U	N	IP	1	30	0.033
192	Chen Z	Transcatheter Amplatzer Occlusion and Surgical Closure of Patent Ductus Arteriosus: Comparison of Effectiveness and Costs in a Low-Income Country	CARDI	PCARD	N	2009	2006	R	nonrandomized cohort	China	AH	U	N	IP	0	130	0.000
193	Chichom Mefire, A.	Diagnostic and therapeutic challenges of isolated small bowel perforations after blunt abdominal injury in low income settings: Analysis of twenty three new cases	GENSX	PERF	N	2013	2007	P	audit	Cameroon	AH	U	N	IP	2	23	0.087
194	Chigbu B	Lessons learned from the outcome of bloodless emergency laparotomies on Jehovah's Witness women presenting	OBGYN	UTRUP	N	2009	2003	R	audit	Nigeria	AH	U	N	IP	10	65	0.154

Reference	First Author	Article Title	Specialty	Procedure or Diagnosis	High-risk population?	Year Published	Data midpoint year	Retrospective or prospective	Study design	Country	Type of Facilities Included	Urban/ Rural/ Mix	Was POMR clearly defined?	Timeframe of POMR	POMR numerator	POMR denominator	Overall POMR
195	Chinnery GE	Surgical management and outcome of civilian gunshot injuries to the pancreas	TRAUM	PTRAUM	N	2012	1995	R	audit	South Africa	AH	U	Y	IP	46	219	0.210
196	Chirdan LB	Sacrococcygeal teratoma: Clinical characteristics and long-term outcome in Nigerian children	NEURO	SACRO	N	2009	1999	R	audit	Nigeria	AH	Mixed	N	IP	3	36	0.083
197	Chongsuvivatwong V	Maternal and fetal mortality and complications associated with cesarean section deliveries in teaching hospitals in Asia	OBGYN	CAES	N	2010	2003	P	nonrandomized cohort	Multiple	AH	U	N	IP	14	7390	0.002
198	Chowdhary, S.	Minimal Access Surgery in Children: A 5 Year Study	PAEDS	MIS	N	2012	2007	P	nonrandomized cohort	India	AH	U	N	30D	0	211	0.000
199	Chowdhury, F. H.	Intracranial epidermoid tumor; microneurosurgical management: An experience of 23 cases	NEURO	RIM	N	2013	2008	P	nonrandomized cohort	Bangladesh	Mixed	U	N	IP	0	23	0.000
200	Chu, K.	Cesarean section rates and indications in sub-Saharan Africa: a multi-country study from Medecins sans Frontieres	OBGYN	CAES	N	2012	2010	P	audit	Multiple	MSF	R	Y	IP	7	1276	0.005
201	Chu, K.	Surgical care for the direct and indirect victims of violence in the eastern Democratic Republic of Congo	MULTI	MULTI	N	2010	2008	P	audit	Congo, Dem. Rep.	DH	R	Y	OT	20	2441	0.008
202	Chu, K.M.	Operative Mortality in Resource-Limited Settings	MULTI	MULTI	N	2010	2004	R	audit	Multiple	MSF	Mixed	Y	OT	31	19643	0.002
203	Chu, K.M.	Providing surgical care in Somalia: A model of task shifting	MULTI	MULTI	N	2011	2008	P	audit	Somalia	MSF	U	Y	OT	8	1602	0.005
204	Claria, R.S.	Laparoscopic Resection for Liver Tumors	HEPAT	LIVRES	N	2009	2004	R	audit	Argentina	AH	U	Y	90D	0	28	0.000
205	Clarke, D.L.	Emergency operation for penetrating thoracic trauma in a metropolitan surgical service in South Africa.	TRAUM	THORTRAUM	N	2011	2007	R	audit	South Africa	AH	U	N	IP	11	108	0.102
206	Coelho, J. C. U.	Surgical Treatment of Cystic Neoplasms of the Pancreas	HEPAT	PANC_RES	N	2010	2003	R	audit	Brazil	Mixed	U	N	IP	1	27	0.037
207	Colafranceschi AS	Videothoracoscopy for Isolated Atrial Fibrillation Ablation through Bipolar Radiofrequency	CARDI	AFIB	N	2009	2007	R	nonrandomized cohort	Brazil	AH	U	N	IP	0	10	0.000
208	Colli B	Foramen magnum meningiomas: surgical treatment in a single public institution in a developing country	NEURO	RIM	N	2014	2004	R	nonrandomized cohort	Brazil	AH	U	N	IP	0	13	0.000
209	Conterno L	Impact of hospital infections on patients outcomes undergoing cardiac surgery at Santa Casa de Misericórdia de Marília	CARDI	CARD	N	2014	2008	R	nonrandomized cohort	Brazil	AH	U	Y	IP	132	2060	0.064
210	Corciova, F.C.	Echocardiographic predictors of adverse short-term outcomes after heart surgery in patients with mitral regurgitation and pulmonary hypertension	CARDI	VALVE	N	2012	2009	R	audit	Romania	AH	U	N	IP	4	171	0.023
211	Corrêa Neto, IJF.	Clinical outcomes of Fournier's gangrene from a tertiary hospital	UROLO	FOURN	N	2012	2009	R	audit	Brazil	Mixed	U	N	IP	4	13	0.308
212	Costa SR	En-bloc pancreatoduodenectomy and right hemicolectomy for treating locally advanced right colon cancer: a series of five patients	HEPAT	WHIP	Y	2009	2003	R	audit	Brazil	AH	U	N	IP	0	5	0.000
213	Cui, H.	Clinical Features and Treatment Outcomes of Moyamoya Disease in 125 Patients of Henan, China	NEURO	MOYA	N	2013	2008	R	audit	China	AH	U	Y	30D	2	25	0.080
214	Cunnigaiper, N. D.	Does Ochsner-Sherren regimen still hold true in the management of appendicular mass?	GENSX	APPY	N	2010	2006	R	audit	India	AH	U	N	IP	0	506	0.000
215	Curi-Curi, P.	Surgical repair of congenital mitral valve malformations	CARDI	PCARD	N	2010	2004	R	nonrandomized cohort	Mexico	AH	U	N	IP	1	14	0.071
216	Dabdoub	How I Do It: Myelomeningocele in Bolivia	NEURO	SPINE	N	2014	2009	R	audit	Bolivia	AH	U	Y	30D	4	60	0.067
217	Dakubo, J.	Gastro-duodenal peptic ulcer perforation	GENSX	PERF	N	2009	2002	Mixed R/P	audit	Ghana	AH	U	Y	30D	26	316	0.082
218	Dalcin R	Ten-Years Comparative Study Study After Surgical Treatment Of Perforated Peptic Ulcer According To Ulcer Relapse Between H. Pylori Positive, After Eradication, And Negative Patients	GENSX	PERF	N	2009	2002	R	nonrandomized cohort	Brazil	AH	U	N	IP	14	144	0.097
219	Dan, R.G.	Postoperative Morbidity and Mortality after Liver Resection. Retrospective Study on 133 Patients	HEPAT	LIVRES	N	2012	2005	R	audit	Romania	AH	U	Y	30D	3	133	0.023
220	Danisman,N.	Emergency peripartum hysterectomy: Experience of a major referral hospital in Ankara, Turkey	OBGYN	EPH	N	2014	2010	R	nonrandomized cohort	Turkey	AH	U	N	IP	0	48	0.000
221	Dar, M.I.	Coronary artery bypass surgery in old age group: Is age itself a barrier?	CARDI	CABG	N	2009	2005	R	audit	Pakistan	AH	U	Y	IP_30D	6	63	0.095
222	Davidov,M.	Early results of laparoscopic resection of the stomach	GENSX	GAST	N	2012	2007	P	nonrandomized cohort	Serbia	CH	U	N	30D	0	5	0.000
223	Davidovic,L.	Repair of abdominal aortic aneurysms in the presence of the horeshoe kidney	VASCU	AAA	Y	2011	1997	R	nonrandomized cohort	Serbia	Other	U	N	IP	2	19	0.105
224	Davoodi, S.	Short- and Mid-Term Results of Triple-Valve Surgery	CARDI	VALVE	Y	2009	2004	R	audit	Iran, Islamic Rep.	AH	U	Y	IP	5	100	0.050
225	Davoodi, S.	Outcomes and long-term quality of life of patients with severe left ventricular dysfunction who underwent coronary artery bypass surgery	CARDI	CABG	Y	2012	2005	P	audit	Iran, Islamic Rep.	AH	U	Y	IP	3	195	0.015
226	De Aguiar P	Posterior Communicating Artery Aneurysms	NEURO	ANEUR	N	2010	2004	R	audit	Brazil	CH	U	N	OT	3	39	0.077
227	de Almeida, A.G.	Incidence of SUDEP in a cohort of patients with refractory epilepsy	NEURO	EPIL	N	2010	1997	Mixed R/P	audit	Brazil	AH	U	Y	30D	1	384	0.003
228	De Araujo Silva, D.O	Chronic subdural hematomas and the elderly: Surgical results from a series of 125 cases: Old "horses" are not to be shot!	NEURO	SDH	N	2012	2007	R	audit	Brazil	AH	U	N	30D	14	125	0.112
229	de Silva, W.D.D.	Challenges in the management of extremity vascular injuries: A wartime experience from a tertiary centre in Sri Lanka	TRAUM	PVI	N	2011	2011	R	audit	Sri Lanka	AH	U	N	IP	0	70	0.000
230	Deboutte D	Cost-effectiveness of caesarean sections in a post-conflict environment: a case study of Bunia, Democratic Republic of the Congo	OBGYN	CAES	N	2013	2008	R	case-control	Congo, Rep.	Mixed	Mixed	N	IP	3	479	0.006
231	Dehaki, M.G.	Recurrence rate of different techniques for repair of coarctation of aorta: A 10 years experience	CARDI	PCARD	N	2010	1999	R	nonrandomized cohort	Iran, Islamic Rep.	AH	U	Y	IP	4	188	0.021
232	Demirci, O.	Emergency peripartum hysterectomy in a tertiary obstetric center: nine years evaluation	OBGYN	EPH	N	2011	2004	R	audit	Turkey	DH	U	N	IP	6	39	0.154
233	Dennison, M	Caesarean Sections at Juba Teaching Hospital 2008-2009	OBGYN	CAES	N	2010	2009	R	audit	South Sudan	AH	U	Y	IP	5	430	0.012
234	Diaconescu, M. R.	Surgical management of renal hyperparathyroidism: a preliminary series report	ENT	PARATHYROID	N	2011	2001	R	nonrandomized cohort	Romania	AH	U	N	IP	0	43	0.000
235	Dias, R.R.	Mortality and Embolic Potential of Cardiac Tumors	CARDI	CARDIACMYX	N	2014	1998	R	nonrandomized cohort	Brazil	AH	U	Y	IP	10	185	0.05405405
236	Dias, R.R.	Mortality Impact of Thoracic Aortic Disease in São Paulo State from 1998 to 2007	CARDI	TAD	N	2013	2002	R	nonrandomized cohort	Brazil	AH	U	Y	IP	725	3572	0.20296753

Reference	First Author	Article Title	Specialty	Procedure or Diagnosis	High-risk population?	Year Published	Data midpoint year	Retrospective or prospective	Study design	Country	Type of Facilities Included	Urban/ Rural/ Mix	Was POMR clearly defined?	Timeframe of POMR	POMR numerator	POMR denomiator	Overall POMR
237	Dias, R.R.	Aortic root reconstruction through valve-sparing operation: critical analysis of 11 years of follow-up	CARDI	TAD	N	2010	2002	R	nonrandomized cohort	Brazil	AH	U	Y	30D_IP	3	54	0.05555556
238	Dinc, B.	Comparing methods of ileostomy closure constructed in colorectal surgery in Turkey	GENSX	ILEOST	N	2014	2010	R	audit	Turkey	AH	U	Y	30D	0	68	0.000
239	Ding, Y.B.	Surgical outcomes for gastric cancer of a single institute in southeast China	GENSX	GASTCA	N	2012	1998	R	audit	China	AH	U	Y	30D_IP	5	1451	0.003
240	Dinkhuysen, J.J.	Clinical evaluation of the Spiral Pump® after improvements to the original project in patients submitted to cardiac surgeries with cardiopulmonary bypass	CARDI	CARD	N	2014	2012	P	audit	Brazil	AH	U	Y	IP	1	52	0.01923077
241	Diom, E.S.	Management of acquired cholesteatoma in children: A 15 year review in ENT service of CHNU de FANN Dakar	PAEDS	CHOLESTEA	N	2013	2002	R	nonrandomized cohort	Senegal	AH	U	N	IP	1	61	0.01639344
242	Dongo, A.	A Review of Posttraumatic Bowel Injuries in Ibadan	TRAUM	LAPAR	N	2011	2002	R	audit	Nigeria	AH	U	N	IP	3	27	0.111
243	Doumi, E.	Acute Abdomen at El Obeid Hospital, Western Sudan	GENSX	AABDO	N	2009	2004	P	audit	Sudan	AH	U	N	IP	36	421	0.086
244	Dracini, X.	Surgical Treatment of Gastric Cancer in Albania	GENSX	GASTCA	N	2012	2005	R	audit	Albania	Other	U	Y	IP	11	624	0.018
245	Du, Y.	Comparison of Two Tranexamic Acid Dose Regimens in Patients Undergoing Cardiac Valve Surgery	CARDI	VALVE	N	2014	2009	P	RCT	China	AH	U	Y	IP	0	175	0.000
246	Duan, S.	Classifications of clinical and bowel morphological changes and their relationship with characteristics of patients with incarcerated groin hernias	GENSX	INGHERN	N	2014	2001	R	nonrandomized cohort	China	AH	U	Y	30D	18	195	0.09230769
247	Duci, S.B.	Surgical Treatment of 55 Patients with Pressure Ulcers at the Department of Plastic and Reconstructive Surgery Kosovo during the Period 2000–2010: A Retrospective Study	PLAST	PULC	N	2013	2005	R	audit	Kosovo	AH	U	N	IP	2	55	0.036
248	Duishanbai S.	Clinical Experience in Treating Intracranial Hydatid Cysts: Report of 97 Cases	NEURO	CRANHYDAT	N	2011	1997	R	audit	China	AH	U	N	IP	2	97	0.021
249	Duttaroy, D.D.	Management Strategy for Dirty Abdominal Incisions: Primary or Delayed Primary Closure? A Randomized Trial	GENSX	LAPAR	N	2007	2006	P	RCT	India	AH	U	Y	IP	4	81	0.04938272
250	Edaigbini, S.A.	Vascular surgeries in West Africa: Challenges and prospects	VASCU	VASCU	N	2014	2010	R	audit	Nigeria	AH	U	N	IP	1	54	0.01851852
251	Edwin, F.	Outcome of left heart mechanical valve replacement in West African children - A 15-year retrospective study	CARDI	VALVE	N	2011	2000	R	audit	Ghana	AH	U	Y	30D	6	114	0.053
252	Edwin, F.	Experience from a single centre concerning the surgical spectrum and outcome of adolescents and adults with congenitally malformed hearts in West Africa	CARDI	CHD	N	2010	2000	R	audit	Ghana	AH	U	Y	30D	4	135	0.030
253	Efetie, E.	Audit Of Gynaecological Laparoscopies In National Hospital Abuja, Nigeria	OBGYN	MIS	N	2009	2002	R	audit	Nigeria	AH	U	N	IP	0	51	0.000
254	Eke, A.C.	Management options for vulvar carcinoma in a low resource setting	OBGYN	VULVCA	N	2010	2003	R	audit	Nigeria	AH	U	Y	IP	0	11	0.000
255	Ekenze SO	Profile of pediatric abdominal surgical emergencies in a developing country.	PAEDS	AABDO	N	2010	2008	P	audit	Nigeria	AH	U	N	IP	10	93	0.108
256	Ekenze, S. O.	Routine surgical intervention for childhood intussusception in a developing country	PAEDS	INTUSS	N	2010	2002	R	audit	Nigeria	AH	U	N	IP	6	71	0.085
257	Ekwunife, C.N.	First 100 Laparoscopic Surgeries in a Predominantly Rural Nigerian Population: A Template for Future Growth	GENSX	MIS	N	2014	2010	R	nonrandomized cohort	Nigeria	DH	R	N	IP	0	100	0
258	Ekwunife, O.	Jejuno-ileal atresia: A 2-year preliminary study on presentation and outcome	PAEDS	IATRES	N	2011	2009	P	audit	Nigeria	AH	U	N	IP	3	9	0.333
259	El-Baradie, M.	Adjuvant postoperative radiochemotherapy for patients with gastric carcinoma: a single institution experience	GENSX	GASTCA	N	2012	2004	R	audit	Egypt, Arab Rep.	AH	U	Y	30D	42	351	0.120
260	Elias, N	Myocardial Fibrosis and Ventricular Remodeling in Severe Chronic Aortic Regurgitation	CARDI	VALVE	N	2009	2007	P	nonrandomized cohort	Brazil	AH	U	N	30D	1	28	0.03571429
261	Eltayeb, A.A.	The role of surgery in management of necrotizing enterocolitis	PAEDS	NEC	N	2010	2007	P	audit	Egypt, Arab Rep.	AH	U	N	IP	11	23	0.478
262	Elusoji, S. O.	Thyroidectomy under ketamine anaesthesia in a semi urban hospital in Nigeria	ENT	GOITRE	N	2009	2003	P	nonrandomized cohort	Nigeria	Other	U	N	IP	0	55	0.000
263	Emmiler, M.	Gastrointestinal ischemia related mortality in patients undergoing off- or on-pump coronary artery bypass grafting	CARDI	CABG	N	2009	2005	R	nonrandomized cohort	Turkey	AH	U	Y	IP	44	2625	0.017
264	Engin, O.	Parasitic Appendicitis From Past to Present in Turkey	GENSX	APPY	N	2010	2005	R	audit	Turkey	AH	U	N	IP	0	9	0.000
265	Ercan, M	Surgical Outcome of Patients with Perforation After Endoscopic Retrograde Cholangiopancreatography	GENSX	PERF	N	2012	2008	P	nonrandomized cohort	Turkey	AH	U	N	IP	9	24	0.375
266	Erdogan, D.	Analysis of 3,776 pediatric inguinal hernia and hydrocele cases in a tertiary center	PAEDS	INGHERN	N	2013	2007	R	audit	Turkey	AH	U	N	IP	0	3776	0.000
267	Erek, E.	Analysis of results according to the Aristotle scoring system in congenital heart surgery	CARDI	PCARD	N	2014	2012	P	nonrandomized cohort	Turkey	AH	U	Y	IP	21	167	0.126
268	Eroglu, A.	Current management of esophageal perforation: 20 years experience_918 374.	THORA	ESPERF	N	2009	1998	R	audit	Turkey	AH	U	Y	IP	3	30	0.100
269	Escarain, M.C.	The Ross Procedure: A Fifteen-Year Experience	CARDI	VALVE	N	2011	2003	R	audit	Argentina	AH	U	Y	30D_IP	8	253	0.032
270	Esmat, M.	Application of Yang-Monti Principle in Ileal Ureter Substitution: is it a beneficial modification?	UROLO	ILEALC	N	2012	2007	P	audit	Egypt, Arab Rep.	AH	U	Y	IP	0	16	0.000
271	Etonyeaku A.C.	A review of the management of perforated duodenal ulcers at a tertiary hospital in south western Nigeria	GENSX	PERF	N	2013	2006	R	audit	Nigeria	AH	U	N	IP	6	45	0.133
272	Evans, C	Using direct clinical observation to assess the quality of cesarean delivery in Afghanistan: an exploratory study	OBGYN	CAES	N	2014	2010	Mixed R/P	audit	Afghanistan	Mixed	Mixed	Y	OT	0	63	0.000
273	Evsen, MS	Retrospective analysis of placenta accreta: management strategies – evaluation of 41 cases	OBGYN	PLACENTAACC	N	2012	2008	R	audit	Turkey	AH	Mixed	Y	6W	1	41	0.024
274	Eze, J.N.	Uterine rupture at a secondary hospital in Afikpo, Southeast Nigeria	OBGYN	UTRUP	N	2010	2004	R	audit	Nigeria	AH	U	N	IP	3	51	0.059
275	Ezomike, U. O.	Outcomes of surgical management of intestinal atresias	PAEDS	IATRES	N	2014	2009	R	audit	Nigeria	AH	U	N	IP	5	23	0.217
276	Ezomike, U. O.	Indication and outcome of childhood preventable bowel resections in a developing country	PAEDS	BRES	N	2014	2008	R	audit	Nigeria	AH	U	N	IP	7	22	0.318

Reference	First Author	Article Title	Specialty	Procedure or Diagnosis	High-risk population?	Year Published	Data midpoint year	Retrospective or prospective	Study design	Country	Type of Facilities Included	Urban/ Rural/ Mix	Was POMR clearly defined?	Timeframe of POMR	POMR numerator	POMR denomiator	Overall POMR
277	Falase, B.	Open heart surgery in Nigeria; a work in progress	CARDI	CARD	N	2013	2008	R	nonrandomized cohort	Nigeria	AH	U	Y	30D	9	51	0.17647059
278	Fan, H.G.	Repair of left ventricular aneurysm: ten-year experience in Chinese patients	CARDI	CARDAN	N	2009	2000	R	audit	China	AH	U	Y	30D	10	497	0.020
279	Fantini, F.A.	Fontan operation: a technique in evolution	CARDI	CCHD	N	2009	2006	P	RCT	Brazil	DH	U	Y	IP	2	40	0.05
280	Faouzi, N.	Intestinal volvulus: aetiology, morbidity and mortality in Tunisian children	PAEDS	MALRO	N	2011	2004	R	audit	Tunisia	AH	U	N	IP	2	31	0.065
281	Faria, M.S.M	Colorectal resection without mechanical colon cleansing: experience with 54 patients	GENSX	COLRES	N	2012	2004	R	nonrandomized cohort	Brazil	DH	U	N	IP	1	54	0.01851852
282	Fatimi, S.H.	Outcomes of surgical management of tracheobronchial injuries: a case series from a developing country	TRAUM	THORTRAUM	N	2011	2006	R	audit	Pakistan	AH	U	N	IP	1	15	0.067
283	Fatimi S.H.	Major thoracic vessels and cardiac trauma: case series from a center in a developing country	TRAUM	CTRAUM	N	2012	2003	R	audit	Pakistan	AH	U	N	IP	2	13	0.154
284	Fattahi, E.	Independent predictors of in-hospital re-bleeding, need of operation and mortality in acute upper gastrointestinal bleeding.	GENSX	UGIB	N	2011	2011	P	audit	Iran, Islamic Rep.	AH	U	N	IP	19	27	0.704
285	Fawole, A.	Predictors of maternal mortality in institutional deliveries in Nigeria	OBGYN	CAES	N	2012	2004	P	audit	NIgeria	Mixed	Mixed	N	IP	31	1344	0.023
286	Fedakar A	Results of mitral vale repair in rheumatic mitral lesions	CARDI	VALVE	N	2009	2003	R	audit	Turkey	AH	U	Y	IP	4	173	0.023
287	Feng, B.	Arterial switch for transposition of the great vessels and Taussig-Bing anomaly after six months of age	CARDI	ASO	N	2009	2004	R	nonrandomized cohort	China	AH	U	N	IP	6	68	0.088
288	Feng, J.F.	Prognostic analysis of carcinoma of the ampulla of Vater: pancreaticoduodenectomy versus local resection	HEPAT	PANC_RES	N	2012	2000	R	nonrandomized cohort	China	AH	U	Y	30D	3	71	0.04225352
289	Fente, B. G.	Experience working with Nurse Anesthetists' as Non-Physician Anesthesia Providers in a temporary Semi-Urban Niger Delta University Teaching Hospital, Okolobiri and review of the literature	MULTI	MULTI	N	2013	2008	R	audit	Nigeria	AH	R	Y	24H	6	1389	0.004
290	Fernandes, E.O.	Thoracic surgery: risk factors for postoperative complications of lung resection	THORA	LUNGRES	N	2011	2010	P	nonrandomized cohort	Brazil	AH	U	Y	30D	26	189	0.13756614
291	Fernandes, R.H.O	Transanal rectopexy – twelve case studies	GENSX	RECTO	N	2012	2004	R	nonrandomized cohort	Brazil	AH	U	N	30D	0	12	0
292	Fernandes, R.L.	Complete removal of the spinal nerve sheath tumors. Surgical technics and results from a series of 30 patients.	NEURO	SPINE	N	2014	1999	R	nonrandomized cohort	Brazil	AH	U	N	IP	1	30	0.03333333
293	Fesseha, N.	A national review of cesarean delivery in Ethiopia	OBGYN	CAES	N	2011	2007	R	audit	Ethiopia	Mixed	Mixed	N	IP	2	267	0.007
294	Fofie, C.O.	A Two-Year Review Of Uterine Rupture In A Regional Hospital	OBGYN	UTRUP	N	2010	2008	R	audit	Ghana	AH	U	N	IP	4	41	0.098
295	Fontana, R	Surgical Outcomes and Prognostic Factors in Patients with Synchronous Colorectal Liver Metastases	GENSX	LIVRES	N	2014	2002	R	nonrandomized cohort	Brazil	AH	U	Y	90D	2	59	0.03389831
296	Fontes, P.R.O	Low mortality rate in 97 consecutive pancreaticoduodenectomies: the experience of a group	HEPAT	WHIP	N	2014	2006	R	nonrandomized cohort	Brazil	AH	U	Y	30D	2	97	0.02061856
297	Forny, D.N.	Choledochal cyst in childhood: review of 30 cases	HEPAT	CDC	N	2014	2002	R	nonrandomized cohort	Brazil	AH	U	N	30D	0	30	0
298	Furlanetto, G.	New technique: Norwood operation with regional cerebral and coronary perfusion	CARDI	CCHD	N	2009	2007	Mixed R/P	nonrandomized cohort	Brazil	DH	U	Y	IP	2	8	0.25
299	Gadelhak, N.	Diagnosis and management of choledochal cyst: 20 years of single center experience	HEPAT	CDC	N	2014	2001	R	nonrandomized cohort	Egypt, Arab Rep.	AH	U	N	IP	0	47	0
300	Gajin, P	Urgent carotid endarterectomy in patients with acute neurological ischemic events within six hours after symptoms onset	VASCU	CEA	N	2013	2003	P	nonrandomized cohort	Serbia	AH	U	Y	30D	0	58	0
301	Gala T	Experience of managing complicated diverticulitis of colon: a retrospective case series from south asian country	GENSX	COLRES	N	2014	2000	R	audit	Pakistan	AH	U	Y	30D	7	45	0.156
302	Galukande, Moses	Mass safe male circumcision: early lessons from a Ugandan urban site - a case study	UROLO	CIRCUM	N	2012	2011	P	nonrandomized cohort	Uganda	AH	U	N	IP	0	3000	0
303	Ganie, F.A.	The Increasing Rate of Secondary Amputation in Popliteal Arterial Injury Associated with Multi-Organ Injuries and Hypotension	TRAUM	PVI	N	2012	2009	R	nonrandomized cohort	India	DH	U	N	IP	10	95	0.10526316
304	Ganigara, M.	Extracardiac Fontan Operation after Late Bidirectional Glenn Shunt	CARDI	CCHD	N	2010	2003	R	audit	India	AH	U	Y	IP_30D	0	33	0.000
305	Gao, J. S.	Inguinal hernia repair with tension-free hernioplasty under local anesthesia	GENSX	INGHERN	N	2009	2007	R	audit	China	AH	U	Y	30D	0	110	0.000
306	Gao, X.	Childhood and juvenile meningiomas	NEURO	RIM	N	2009	2000	R	audit	China	AH	U	N	IP	2	54	0.037
307	Garofallo, S.B.	Applicability of Two International Risk Scores in Cardiac Surgery in a Reference Center in Brazil	CARDI	CARD	N	2014	2007	R	nonrandomized cohort	Brazil	AH	U	Y	IP	83	1065	0.07793427
308	Gathura, E.	Outcomes of ventriculoperitoneal shunt insertion in Sub-Saharan Africa	NEURO	HYDRO	N	2010	2005	R	audit	Kenya	AH	U	N	IP	3	574	0.005
309	Gerges, S.S.	Laparoscopic cholecystectomy in liver cirrhosis patients: An Egyptian experienceash_574 12..16	GENSX	CHOLE	Y	2012	2010	Mixed R/P	nonrandomized cohort	Egypt, Arab Rep.	AH	U	N	IP	0	177	0
310	Gessessew, A.	Task shifting and sharing in Tigray, Ethiopia, to achieve comprehensive emergency obstetric care	OBGYN	CAES	N	2010	2007	R	audit	Ethiopia	Mixed	U	N	IP	17	2835	0.006
311	Ghandehari, K.	Results of surgical and nonsurgical treatment of aneurysms in a developing country.	NEURO	ICH	N	2011	2007	P	nonrandomized cohort	Iran, Islamic Rep.	AH	U	N	IP	28	45	0.622
312	Gharsallah, H.	Cardiac Surgery in Patients on Hemodialysis: Eight Years Experience of the Tunisian Military Hospital	CARDI	CARD	Y	2010	2003	R	audit	Tunisia	AH	U	N	IP	6	26	0.231
313	Ghezzi, T.L.	Analysis of the videolaparaoscopy potentiality in the surgical treatment of the bowel obstruction	GENSX	BOBS	N	2010	2006	R	audit	Brazil	AH	U	Y	30D	26	135	0.193
314	Ghodsi, M	Exophytic Glioma of the Medulla: Presentation, Management and Outcome	NEURO	RIM	N	2013	2007	R	audit	Iran, Islamic Rep.	AH	U	Y	30D	0	11	0.000
315	Ghosh, S.	Clinical, Laboratory and Management Profile in Patients of Liver Abscess from Northern India	HEPAT	LIVABS	N	2014	2012	P	audit	India	AH	U	N	IP	5	8	0.625
316	Ghritlaharey, R.K.	Exploratory laparotomy for acute intestinal conditions in children: a review of 10 years of experience with 334 cases.	PAEDS	LAPAR	N	2011	2004	R	audit	India	AH	U	N	IP	34	334	0.102

Reference	First Author	Article Title	Specialty	Procedure or Diagnosis	High-risk population?	Year Published	Data midpoint year	Retrospective or prospective	Study design	Country	Type of Facilities Included	Urban/ Rural/ Mix	Was POMR clearly defined?	Timeframe of POMR	POMR numerator	POMR denomiator	Overall POMR
317	Gil G	Surgical Treatment of Endemic Goiter in a Nonhospital Setting without General Anesthesia in Africa	ENT	GOITRE	N	2014	2008	R	audit	Multiple	CH	R	N	IP	0	31	0.000
318	Gilyoma, J.	Ten-year experiences with Tracheostomy at a University teaching hospital in Northwestern Tanzania: A retrospective review of 214 cases	ENT	TRACHEOST	N	2011	2005	R	audit	Tanzania	AH	U	N	IP	29	214	0.136
319	Gilyoma, J.M.	Cut throat injuries at a university teaching hospital in northwestern Tanzania: a review of 98 cases.	TRAUM	PNECK	N	2014	2011	Mixed R/P	audit	Tanzania	AH	U	N	IP	11	98	0.112
320	Gnanappa, G.	Outcome of Complex Adult Congenital Heart Surgery in the developing world	CARDI	CHD	N	2011	2005	R	nonrandomized cohort	India	AH	U	N	IP	2	153	0.013
321	Gomes, W.J.	The renewed concept of the Batista operation for ischemic cardiomyopathy: maximum ventricular reduction	CARDI	CARDAN	N	2011	2005	Mixed R/P	nonrandomized cohort	Brazil	AH	U	Y	30D	3	76	0.03947368
322	Gong J	Short-term outcomes of laparoscopic total mesorectal excision compared to open surgery	GENSX	RECTAL	N	2012	2010	R	nonrandomized cohort	China	AH	U	N	IP	0	138	0.000
323	Gonullu, D.	Treatment of Penetrating Hepatic Injuries: A Retrospective Analysis of 50 Patients	TRAUM	LIVTRAUM	N	2009	2004	R	nonrandomized cohort	Turkey	AH	U	N	IP	5	43	0.116
324	Gonzales, G.F.	Pregnancy outcomes associated with Cesarean deliveries in Peruvian public health facilities	OBGYN	CAES	N	2013	2005	R	audit	Peru	Mixed	Mixed	Y	6W	161	152110	0.001
325	Gonzalez, Q.H.	Laparoscopic versus open total mesorectal excision: a nonrandomized comparative prospective trial in a tertiary center in Mexico City	GENSX	RECTAL	N	2009	2006	P	audit	Mexico	DH	U	Y	30D	0	56	0.000
326	Govender, M.	Current management of large bowel injuries and factors influencing outcome.	TRAUM	LAPAR	N	2009	2001	P	audit	South Africa	AH	U	Y	30D	29	177	0.164
327	Grinberg, M	Validation of a New Surgical Risk Score for Heart Valve Surgery: VMCP	CARDI	VALVE	N	2009	2007	R	audit	Brazil	AH	U	Y	IP	44	764	0.058
328	Guedes, M.A.V.	Mitral valve surgery using right anterolateral thoracotomy: is the aortic cannulation a safety procedure?	CARDI	VALVE	N	2010	1995	R	nonrandomized cohort	Brazil	AH	U	Y	IP	0	100	0
329	Guler, A.	Can cardiac surgery be performed safely on patients with haematological malignancies	CARDI	CARD	Y	2012	2006	R	audit	Turkey	AH	U	Y	IP	0	15	0.000
330	Gunawansa, N.	Open repair of infra renal abdominal aortic aneurysms: a single center experience from the developing world	VASCU	AAA	N	2011	2007	R	audit	Sri Lanka	AH	U	Y	IP	14	79	0.177
331	Gungorduk, K.	Peripartum hysterectomy in Turkey: a case-control study	OBGYN	EPH	N	2009	2004	R	audit	Turkey	AH	U	N	IP	2	91	0.022
332	Guo, F.	Surgical strategy for gastric cancer patients with liver cirrhosis: A retrospective cohort study	GENSX	GASTCA	Y	2014	2006	R	nonrandomized cohort	China	AH	U	Y	IP	11	58	0.18965517
333	Guo, Q.	Surgical treatment of pancreatic islet cell tumor: report of 44 cases	HEPAT	PANC_RES	N	2013	2005	R	audit	China	AH	U	Y	IP	0	44	0.000
334	Gupta, A.R.	Minimal access surgery in children: An initial experience of 28 months	PAEDS	MIS	N	2009	2006	R	audit	India	AH	U	N	IP	0	193	0.000
335	Gursoy, S.	Seven years experience of bronchogenic cysts	THORA	LUNGRES	N	2009	2003	R	audit	Turkey	AH	U	N	30D	0	28	0.000
336	Gursoy, S.	Primary Intrathoracic Extrapulmonary Hydatid Cysts	THORA	THORHYDAT	N	2009	2005	R	nonrandomized cohort	Turkey	AH	U	N	30D	0	14	0
337	Gurtani, F.M.	Emergency peripartum hysterectomy in Isfahan; maternal mortality and morbidity rates among the women who underwent peripartum hysterectomy	OBGYN	EPH	N	2012	2006	R	audit	Iran, Islamic Rep.	AH	U	N	IP	7	41	0.171
338	Gwely, N.N.	Management of Stab Wounds of the Heart: Analysis of 73 Cases in 10 Years	TRAUM	CTRAUM	N	2010	2003	R	audit	Egypt, Arab Rep.	AH	U	N	IP	17	73	0.233
339	Haddad, R	Reduction aortoplasty with external wrapping associated with aortic valve replacement in high-risk patients	CARDI	TAD	N	2009	2006	Mixed R/P	nonrandomized cohort	Brazil	DH	U	Y	IP	0	6	0
340	Hadi, A.	Surgical management of bile duct injuries following open or laparoscopic cholecystectomy	HEPAT	BILD	N	2013	2005	P	audit	Pakistan	AH	U	Y	IP	1	32	0.031
341	Hadzi-Djokic, J.	Vesico-vaginal fistula: report of 220 cases	OBGYN	VVF	N	2009	1991	R	audit	Serbia	AH	U	N	IP	0	220	0.000
342	Hagander, L.	Major Neonatal Surgery Under Local Anesthesia: A Cohort Study from Bangladesh	PAEDS	PAED	N	2014	2010	P	audit	Bangladesh	Mixed	Mixed	Y	IP	63	568	0.111
343	Haider, S	To Find out Maternal and Fetal Outcome of Cardiac Disease in Pregnancy	OBGYN	CAES	Y	2014	2010	R	audit	Pakistan	AH	U	N	IP	0	5	0.000
344	Hajjar, L.A.	Transfusion Requirements After Cardiac Surgery The TRACS Randomized Controlled Trial	CARDI	CARD	N	2010	2009	P	RCT	Brazil	AH	U	Y	30D	28	512	0.055
345	Halesha, B	Emergency Peripartum Hysterectomy: A Retrospective Study at a Tertiary Care Hospital in Karnataka	OBGYN	EPH	N	2013	2010	R	audit	India	AH	U	N	IP	4	25	0.160
346	Hamid R	Late -presenting congenital diaphragmatic hernia	PAEDS	CDH	N	2014	2010	R	audit	India	AH	U	N	30D	0	20	0.000
347	Hannan, M	Intestinal obstruction in children due to segmental enteritis: experience in Chittagong, Bangladesh	PAEDS	BOBS	N	2012	2006	R	audit	Bangladesh	AH	U	Y	30D	2	24	0.083
348	Hasbahceci, M.	Laparoscopic Cholecystectomy in a Single, Non-teaching Hospital: An Analysis of 1557 Patients	GENSX	CHOLE	N	2012	2005	R	audit	Turkey	CH	U	N	IP	2	1557	0.001
349	Hashemi, S	Timing of Surgery for Aneurysmal Subarachnoid Hemorrhage	NEURO	ICH	N	2010	2006	R	nonrandomized cohort	Iran, Islamic Rep.	AH	U	N	IP	17	70	0.243
350	Hashemzadeh, S	Surgical treatment of postintubation tracheal stenosis: Iranian experience of effect of previous tracheostomy	THORA	TRACHSTEN	N	2012	2007	R	audit	Iran, Islamic Rep.	AH	U	Y	IP	1	50	0.020
351	Hassan, N.	Uterine Rupture at LUMHS: A Review of 85 Cases	OBGYN	UTRUP	N	2009	2005	P	audit	Pakistan	AH	U	N	IP	5	84	0.060
352	Haynes, A.B.	Surgical outcome measurement for a global patient population: validation of the Surgical Apgar Score in 8 countries.	MULTI	MULTI	N	2010	2008	P	audit	Multiple	Mixed	U	Y	30D	83	5909	0.014
353	Hellar, A.M.	The Pattern and Surgical Management of Diabetic Foot at Muhimbili National Hospital, Dar-es-salaam, Tanzania	VASCU	DIABINF	N	2011	2008	P	audit	Tanzania	AH	U	Y	IP	8	58	0.138
354	Hiep, P.N.	Laparoscopic surgery in rectal cancer: a retrospective analysis	GENSX	RECTAL	N	2009	2004	R	audit	Vietnam	AH	U	Y	30D	0	60	0.000
355	Hovnanian, A.L.	Surgical Myocardial Revascularization Of Patients With Ischemic Cardiomyopathy And Severe Left Ventricular Disfunction	CARDI	CABG	Y	2010	2001	R	audit	Brazil	AH	U	Y	IP	9	244	0.037
356	Hu, G.	Analysis of 287 Patients with Aortic Dissection: General Characteristics, Outcomes and Risk Factors in a Single Center	CARDI	TAD	N	2011	2005	R	audit	China	AH	U	Y	30D	4	36	0.111

Reference	First Author	Article Title	Specialty	Procedure or Diagnosis	High-risk population?	Year Published	Data midpoint year	Retrospective or prospective	Study design	Country	Type of Facilities Included	Urban/ Rural/ Mix	Was POMR clearly defined?	Timeframe of POMR	POMR numerator	POMR denominator	Overall POMR
357	Huang, L,	Prealbumin is predictive for postoperative liver insufficiency in patients undergoing liver resection	HEPAT	LIVRES	N	2012	2009	R	audit	China	AH	U	Y	IP	1	427	0.002
358	Huang, Z	Hepatic resection: an analysis of the impact of operative and perioperative factors on morbidity and mortality rates in 2008 consecutive hepatectomy cases	HEPAT	LIVRES	N	2009	1995	R	audit	China	AH	U	Y	IP	11	2008	0.005
359	Hussain, D.	Outcome of extra-mucosal small gut anastomosis in a peripheral hospital	GENSX	BRES	N	2009	2006	P	nonrandomized cohort	Pakistan	AH	U	N	IP	1	100	0.010
360	Hyginus, E.	Morbidity and mortality following high order caesarean section in a developing country	OBGYN	CAES	Y	2012	2003	R	case-control	Nigeria	AH	U	N	IP	0	272	0.000
361	Ibrahim, A.G.	One-Stage Urethroplasty for Strictures in Maiduguri, North Eastern Nigeria	UROLO	URETHRAL	N	2012	2005	R	audit	Nigeria	AH	U	N	IP	0	91	0.000
362	Iddriss, A.	Pulmonary Resection for Extensively Drug Resistant Tuberculosis in Kwazulu-Natal, South Africa	THORA	XDRTB	N	2012	2008	R	audit	South Africa	AH	U	N	30D	0	5	0.000
363	Idowu, O.	Outcome of endoscopic third ventriculostomy and Chhabra shunt system in noncommunicating non-tumor childhood hydrocephalus	NEURO	HYDRO	N	2009	2007	Mixed R/P	audit	Nigeria	AH	U	N	IP	0	36	0.000
364	Igberase,G.O.	High caesarean section rate: A ten year experience in a tertiary hospital in the Niger Delta, Nigeria	OBGYN	CAES	N	2009	1999	R	nonrandomized cohort	Nigeria	CH	R	N	IP	25	1777	0.014
365	Iglezias, JCR	Degree of risk related to procedures performed IN conjunction with surgical myocardial revascularization IN octogenarians	CARDI	CABG	N	2009	2002	R	nonrandomized cohort	Brazil	AH	U	Y	IP	31	147	0.211
366	Igwegbe, A.O.	Risk factors and perinatal outcome of uterine rupture in a low-resource setting	OBGYN	UTRUP	N	2013	2005	R	audit	Nigeria	AH	U	N	IP	7	43	0.163
367	Ikeoka, DT	Evaluation of the Society of Thoracic Surgeons score system for isolated coronary bypass graft surgery in a Brazilian population	CARDI	CABG	N	2014	2010	R	audit	Brazil	AH	U	Y	30D_IP	47	1083	0.043
368	Ilori, I.U.	Factors associated with mortality in neonatal surgical emergencies in a developing tertiary hospital in Nigeria	PAEDS	PAED	N	2013	2009	R	audit	Nigeria	AH	U	N	IP	28	45	0.622
369	Islam, J.	Lessons from emergency laparotomy for abdominal tuberculosis in the HIV/AIDS era	GENSX	ABDOTB	N	2014	2009	P	audit	South Africa	AH	Mixed	N	IP	19	49	0.388
370	Islam, M	Rate of cesarean delivery at hospitals providing emergency obstetric care in Bangladesh	OBGYN	CAES	N	2014	2008	R	audit	Bangladesh	Mixed	Mixed	Y	IP	0	1075	0.000
371	Iyem, H.	Evaluation of the reliability of the EurosCorE risk- analysis prediction in high-risk older patients undergoing CaBg	CARDI	CARD	Y	2009	2008	P	audit	Turkey	AH	U	Y	30D	8	128	0.063
372	Jaha, L.	A decade of civilian vascular trauma in Kosovo	TRAUM	PVI	N	2012	2005	Mixed R/P	audit	Kosovo	AH	U	Y	OT	4	120	0.033
373	Jain, B.K.	Colonic perforation with peritonitis in amoebiasis: A tropical disease with high mortalit	GENSX	PERF	N	2013	2011	R	audit	India	AH	U	N	IP	6	15	0.400
374	Jaipuria, J	Paediatric extremity vascular injuries - Experience from a large urban trauma centre in India	TRAUM	PVI	N	2014	2010	R	audit	India	AH	U	N	IP	2	82	0.024
375	Jakab, F.	Transverse hepatectomy: a 14-years experience	HEPAT	LIVRES	N	2012	2001	P	nonrandomized cohort	Hungary	AH	U	N	IP	2	22	0.091
376	Jan, W. A.	Outcome of open versus laproscopic appendicectomy in department of surgery, lady reading hospital, Peshawar	GENSX	APPY	N	2011	2008	P	RCT	Pakistan	AH	U	N	IP	0	120	0.000
377	Janati, M.	Outcome of penetrating cardiac injuries in southern Iran, Shiraz	TRAUM	CTRAUM	N	2013	2004	R	audit	Iran, Islamic Rep.	AH	U	N	IP	8	36	0.222
378	Janjua, A. M.	Double flap patch closure of VSD with elevated pulmonary vascular resistance: An experience at AFIC/NIHD	CARDI	MCHD	N	2011	2007	P	nonrandomized cohort	Pakistan	AH	U	Y	30D	1	40	0.025
379	Jat,N.	Laparoscopic cholecystectomy - 5 years experience at SOM Fauji foundation hospital Karachi	GENSX	CHOLE	N	2011	2007	R	nonrandomized cohort	Pakistan	CH	U	N	IP	0	290	0.000
380	Javaid, S.	Postpartum and emergency caesarean hysterectomy	OBGYN	EPH	N	2011	2009	R	audit	Pakistan	AH	U	N	IP	0	13	0.000
381	Jehangir, S	Intussusception in southern India: Comparison of retrospective analysis and active surveillance	PAEDS	INTUSS	N	2014	2011	Mixed R/P	audit	India	AH	U	Y	IP	0	31	0.000
382	Ji, Q	Risk Factors for Pulmonary Complications Following Cardiac Surgery with Cardiopulmonary Bypass	CARDI	CARD	Y	2013	2008	R	audit	China	AH	U	Y	30D_IP	72	2056	0.035
383	Jiang, T.	The diagnosis and treatment of subependymal giant cell astrocytoma combined with tuberous sclerosis	NEURO	RIM	N	2011	2001	R	audit	China	AH	U	N	IP	0	16	0.000
384	Joaquim, AF	Axis instrumentation: surgical results	NEURO	SPINE	N	2012	2010	R	audit	Brazil	AH	U	Y	IP	0	17	0.000
385	Kacila, M.	Assessment Of The Initial And Modified Parsonnet Score In Mortality Prediction Of The Patients Operated In The Sarajevo Heart Cente	CARDI	CARD	N	2010	2007	R	audit	Bosnia and Herzeg	AH	U	Y	IP	6	145	0.041
386	Kadowa, I.	Ruptured uterus in rural Uganda: prevalence, predisposing factors and outcomes	OBGYN	UTRUP	N	2010	2005	R	audit	Uganda	DH	U	N	IP	6	73	0.082
387	Kakar, B.K.	Surgical Experience of Chronic Constrictive Pericarditis at Quetta, Pakistan	CARDI	PERICAR	N	2012	2003	R	audit	Pakistan	AH	U	Y	30D	1	18	0.056
388	Kamani, F.	Perforated peptic ulcer disease: mid-term outcome among Iranian population	GENSX	PERF	N	2010	2000	R	audit	Iran, Islamic Rep.	AH	U	Y	30D	4	56	0.071
389	Kamilya, G.	Maternal mortality and cesarean delivery: an analytical observational study	OBGYN	CAES	N	2010	2004	R	audit	India	AH	U	N	IP	51	16224	0.003
390	Kandakure, P.R.	Sternotomy Approach for Modified Blalock-Taussig Shunt: Is It a Safe Option?	CARDI	CCHD	N	2010	2008	R	audit	India	AH	U	Y	IP	1	20	0.050
391	Kandasamy, T.	Cesarean delivery surveillance system at a maternity hospital in Kabul, Afghanistan.	OBGYN	CAES	N	2009	2006	R	audit	Afghanistan	DH	U	Y	IP	2	392	0.005
392	Kara,M.	Analysis of uterine rupture cases in Agri: A five-year experience	OBGYN	UTRUP	N	2010	2006	R	nonrandomized cohort	Turkey	CH	U	N	IP	2	44	0.045
393	Kara, M	Emergency peripartum hysterectomy cases in Agri: a 6-year review	OBGYN	EPH	N	2011	2006	R	audit	Turkey	AH	U	N	IP	3	54	0.056
394	Karapandzic, V.M.	Risk Assessment in Coronary Patients Undergoing Abdominal Nonvascular Surgery	GENSX	LAPAR	Y	2009	2007	P	audit	Serbia	AH	U	Y	30D	6	111	0.054
395	Karayalcin, R.	Emergency peripartum hysterectomy	OBGYN	EPH	N	2011	2005	R	audit	Turkey	AH	U	N	IP	2	73	0.027
396	Kargar, S.	Laparoscopic versus open appendectomy; which method to choose? A prospective randomized comparison	GENSX	APPY	N	2011	2008	P	RCT	Iran, Islamic Rep.	AH	U	Y	30D	0	100	0.000
397	Karkee, R	Obstetric complications and cesarean delivery in Nepal	OBGYN	CAES	N	2014	2012	P	audit	Nepal	Mixed	Mixed	Y	6W	0	85	0.000

Reference	First Author	Article Title	Specialty	Procedure or Diagnosis	High-risk population?	Year Published	Data midpoint year	Retrospective or prospective	Study design	Country	Type of Facilities Included	Urban/ Rural/ Mix	Was POMR clearly defined?	Timeframe of POMR	POMR numerator	POMR denomiator	Overall POMR
398	Karpelowsky, J.S.	Outcomes of human immunodeficiency virus-infected and -exposed children undergoing surgery--a prospective study.	PAEDS	PAED	Y	2009	2005	P	nonrandomized cohort	South Africa	AH	U	Y	IP	6	95	0.063
399	Karpelowsky, J.S.	Comparison of in-hospital morbidity and mortality in HIV-infected and uninfected children after surgery	PAEDS	PAED	N	2012	2006	P	nonrandomized cohort	South Africa	AH	U	Y	IP	6	327	0.018
400	Karpelowsky, J.S.	Predictors of postoperative complications in HIV-infected children undergoing surgery.	PAEDS	PAED	Y	2011	2006	P	audit	South Africa	AH	U	Y	IP	6	82	0.073
401	Kassi, A.	Morbidity and Mortality of Hartmann's Procedure for Sigmoid Volvulus at the University Hospital of Cocody, Abidjan	GENSX	VOLV	N	2011	2003	R	audit	Cote d'Ivoire	AH	U	N	IP	3	25	0.120
402	Kaushish L.C.R.	Beating Heart versus Conventional Coronary Bypass Surgery : Our Experience	CARDI	CABG	N	2010	2006	R	nonrandomized cohort	India	Mixed	Mixed	N	IP	7	400	0.018
403	Kaya, B.	Immediate appendectomy for appendiceal mass	GENSX	APPY	N	2012	2007	R	audit	Turkey	AH	U	N	IP	0	47	0.000
404	Kazim, S.F.	Appendicitis in pregnancy: experience of thirty-eight patients diagnosed and managed at a tertiary care hospital in Karachi.	GENSX	APPY	Y	2009	1998	R	audit	Pakistan	AH	U	N	IP	0	37	0.000
405	Kendig, C. E.	Pediatric surgical care in Lilongwe, Malawi: outcomes and opportunities for improvement	PAEDS	PAED	N	2014	2012	R	audit	Malawi	DH	U	Y	IP	14	392	0.036
406	Kertai, M.D.	Intraoperative use of packed red blood cell transfusion and mortality in patients underoign abdominal or thoracoabdominal aortic aneurysm surgery	VASCU	AAA	N	2009	2003	R	audit	Hungary	AH	U	Y	30D	85	1000	0.085
407	Khalaf, I.	The outcome of open renal stone surgery calls for limitation of its use: A single institution experience	UROLO	RSS	N	2013	2005	R	audit	Egypt, Arab Rep.	AH	U	Y	30D	2	533	0.004
408	Khaleghnejad Tabari, A	Acute Mediastinitis in Children: A Nine-Year Experience	PAEDS	MEDIAST	N	2013	2005	R	audit	Iran, Islamic Rep.	AH	U	N	IP	1	16	0.063
409	Khalil, J.	Laparoscopic versus open appendectomy: a comparison of primary outcome measures	GENSX	APPY	N	2011	2008	P	RCT	Pakistan	AH	U	Y	30D	0	147	0.000
410	Khamechian, T	Pattern of splenectomy indications in Kashan Shahid-Beheshti Hospital: A 5 year study	TRAUM	SPLEEN	N	2013	2009	Mixed R/P	audit	Iran, Islamic Rep.	DH	U	N	IP	8	99	0.081
411	Khan, A.	Changing trends in incidence and indications of caesarean section	OBGYN	CAES	N	2014	2006	P	audit	Bangladesh	AH	U	Y	IP	0	130	0.000
412	Khan, B.	A ten year review of emergency peripartum hysterectomy in a tertiary care hospital	OBGYN	EPH	N	2012	2005	R	audit	Pakistan	AH	R	N	IP	23	218	0.106
413	Khan,I.M.	Palliative surgery for pancreatic carcinoma	HEPAT	PALL	N	2010	2007	P	nonrandomized cohort	Pakistan	AH	U	N	IP	3	40	0.075
414	Khan, K.	Modified posterior sagittal ano-rectoplasty: a new approach for the management of ano-rectal malformations in children	PAEDS	ANOMAL	N	2012	2005	P	audit	Pakistan	AH	U	N	IP	1	40	0.025
415	Khan, K. I.	Comparison of rate of surgical wound infection, length of hospital stay and patient convenience in complicated appendicitis between primary closure and delayed primary closure	GENSX	APPY	Y	2012	2008	P	RCT	Pakistan	AH	U	N	IP	0	100	0.000
416	Khan, M.	Clinical profile and surgical outcome for pulmonary aspergilloma: Nine year retrospective observational study in a tertiary care hospital	THORA	LUNGRES	N	2011	2004	R	audit	India	AH	U	Y	30D	1	52	0.019
417	Khan, M.A.	Clinical review of pediatric pilocytic astrocytomas treated at a tertiary care hospital in Pakistan	NEURO	RIM	N	2012	2001	R	audit	Pakistan	AH	U	Y	IP	1	22	0.045
418	Khan, M.B.	Civilian Craniocerebral Gunshot Injuries in a Developing Country: Presentation, Injury Characteristics, Prognostic Indicators, and Complications.	NEURO	CGSW	N	2014	2004	R	audit	Pakistan	AH	U	N	IP	6	45	0.133
419	Khan, M.R.	Early postoperative outcome after curative colorectal cancer surgery	GENSX	COLRES	N	2011	2003	R	audit	Pakistan	AH	U	Y	30D	4	250	0.016
420	Khan, M.	Impact of age on outcome after colorectal cancer surgery in the elderly - a developing country perspective	GENSX	COLRES	N	2011	2003	R	audit	Pakistan	AH	U	Y	30D	6	271	0.022
421	Khan, M.R.	Abdominal wall hernia repair in cirrhotic patients: outcomes seen at a tertiary care hospital in a developing country	GENSX	AWH	Y	2010	2004	R	audit	Pakistan	AH	U	Y	30D	2	61	0.033
422	Khan MS	Cardiac myxoma: A surgical experience of 38 patients over 9 years, at SSKM hospital Kolkata, India	CARDI	CARDIACMYX	N	2013	2007	R	audit	India	AH	U	Y	30D	2	38	0.053
423	Khan, M.Z.	Outcome and factors associated with hospital mortality in patients with impaired left ventricular function undergoing coronary artery bypass grafting: where do we stand?	CARDI	CABG	Y	2009	2007	R	audit	Pakistan	AH	U	Y	30D	12	190	0.063
424	Khan, T.R.	Traumatic diaphragmatic injuries in children: do they really mark the severity of injury? Our experience	TRAUM	TDH	N	2009	2005	R	audit	India	AH	U	N	IP	0	8	0.000
425	Khan, Z.	Early complications of simple anterior cervical discetomy	NEURO	DISC	N	2012	2009	P	audit	Pakistan	AH	U	N	IP	1	95	0.011
426	Khanna, A.K.	A Case Series Describing 118 Patients With Lower Limb Necrotizing Fasciitis	GENSX	NECFASC	N	2009	2001	R	audit	India	AH	U	N	IP	10	110	0.091
427	Khanum, F.	Emergency peripartum hysterectomy in a tertiary care hospital	OBGYN	EPH	N	2013	2008	R	audit	Pakistan	Mixed	Mixed	N	IP	9	51	0.176
428	Khemakhem, R.	Congenital diaphragmatic hernia in neonate: A retrospective study about 28 observations	PAEDS	CDH	N	2012	2004	R	audit	Tunisia	AH	U	N	IP	8	25	0.320
429	Khorram-Manesh, A.	Management of Traumatic Liver Injuries without a Valid Trauma System	TRAUM	LIVTRAUM	N	2009	2003	R	audit	Iran, Islamic Rep.	AH	U	Y	IP	39	216	0.181
430	Khumjui, C.	Incidence of intussusception among children 0–5 years of age in Thailand, 2001–2006	PAEDS	INTUSS	N	2009	2003	R	audit	Thailand	DH	Mixed	N	IP	0	35	0.000
431	Kiboi, J.G.	Outcome after acute traumatic subdural haematoma in Kenya: a single-centre experience	NEURO	SDH	N	2010	2008	R	audit	Kenya	AH	Mixed	Y	IP	36	205	0.176
432	Kim, Y.	Quality of caesarean delivery services and documentation in first-line referral facilities in Afghanistan: a chart review	OBGYN	CAES	N	2012	2009	R	audit	Afghanistan	Mixed	Mixed	N	IP	27	173	0.156
433	Kishore, G.S.B.	Traumatic diaphragmatic hernia: tertiary centre experience	TRAUM	TDH	N	2010	2005	R	audit	India	AH	U	N	IP	3	27	0.111
434	Kitara, D.L.	The Postoperative Complications Prediction in Mulago Hospital using POSSUM Scoring System.	GENSX	LAPAR	N	2010	2003	P	audit	Uganda	AH	U	Y	30D	11	76	0.145

Reference	First Author	Article Title	Specialty	Procedure or Diagnosis	High-risk population?	Year Published	Data midpoint year	Retrospective or prospective	Study design	Country	Type of Facilities Included	Urban/ Rural/ Mix	Was POMR clearly defined?	Timeframe of POMR	POMR numerator	POMR denomiator	Overall POMR
435	Kithikii, K.P.	Risk Factors Related to Hospital Mortality in Kenyan Patients with Traumatic Intracranial Haematomas	NEURO	ACHI	N	2011	2004	R	audit	Kenya	AH	U	Y	IP	70	447	0.157
436	Koh, K.H.	Outcome of Coronary Artery Bypass Grafting in End Stage Renal Disease Patients	CARDI	CABG	Y	2012	2006	Mixed R/P	case-control	Malaysia	AH	U	N	IP	0	11	0.000
437	Kong, V.	Acute Appendicitis in a Developing Country	GENSX	APPY	N	2012	2011	P	audit	South Africa	AH	U	N	IP	4	200	0.020
438	Kong, V	Quantifying the disparity in outcome between urban and rural patients with acute appendicitis in South Africa	GENSX	APPY	N	2013	2011	P	nonrandomized cohort	South Africa	DH	U	Y	IP	8	500	0.016
439	Korejo, R.	Emergency obstetric hysterectomy	OBGYN	EPH	N	2012	2006	R	audit	Pakistan	AH	U	N	IP	11	121	0.091
440	Koszta, G.	Lower whole blood selenium level is associated with higher operative risk and mortality following cardiac surgery	CARDI	CARD	N	2012	2011	P	audit	Hungary	AH	U	Y	30D	11	197	0.056
441	Koszta, G.	Performance of EuroSCORE II in Hungary: A Single-centre Validation Study	CARDI	CARD	N	2014	2011	P	audit	Hungary	AH	U	Y	IP	123	2287	0.054
442	Kotb, S.	Renal recoverability in infants with obstructive calcular anuria: Is it better than in older children?	PAEDS	RSS	N	2013	2010	P	audit	Egypt, Arab Rep.	AH	U	Y	30D	0	9	0.000
443	Kothari, K.	Comparison of esophagectomy with and without thoracotomy in a low-resource tertiary care center in a developing country	THORA	ESOCA	N	2011	2004	P	nonrandomized cohort	India	AH	U	N	IP	2	62	0.032
444	Kotze, P.G.	Complications after intestinal resection in Crohn's disease: laparoscopic versus conventional approach laparoscopic versus conventional approach	GENSX	CROHN	N	2013	2010	R	audit	Brazil	AH	U	Y	30D	3	46	0.065
445	Kritayakirana, K.	Cardiac trauma: has survival improved? A university hospital experience in Bangkok, Thailand	CARDI	CTRAUM	N	2013	2002	R	audit	Thailand	AH	U	N	IP	6	44	0.136
446	Kucukarslan N	Coronary artery bypass surgery in patients with malignancy: a single-center study with comparison to patients without malignancy.	CARDI	CABG	Y	2009	2001	R	nonrandomized cohort	Turkey	AH	U	N	IP	4	98	0.041
447	Kwari, D.Y.	Cleft lip and palate surgery in children: Anaesthetic considerations	PLAST	CLEFT	N	2010	2009	P	audit	Nigeria	Mixed	U	Y	IP	0	106	0.000
448	Kwari, Y.D.	Pattern of perioperative cardiac arrests at University of Maiduguri Teaching Hospital	MULTI	MULTI	N	2010	2008	R	audit	Nigeria	AH	U	Y	OT	7	4051	0.002
449	Lacerda, CF	Totally laparoscopic liver resection: new Brazilian experience	HEPAT	LIVRES	N	2014	2011	R	audit	Brazil	AH	U	N	IP	1	61	0.016
450	Laiq, N.	Intravenous Magnesium Prevents Atrial Fibrillation After Valvular Heart Surgery	CARDI	VALVE	N	2013	2008	P	RCT	Pakistan	AH	U	N	IP	0	100	0.000
451	Lakhey, P.J.	Perioperative Outcomes of Pancreaticoduodenectomy: Nepalese Experience	HEPAT	WHIP	N	2010	2007	R	audit	Nepal	AH	U	Y	30D_IP	0	24	0.000
452	Lal, P.	Laparoscopic Nissen Fundoplication Is an Excellent Modality for GERD: Early Experience from a Tertiary Care Hospital in India	GENSX	NISSN	N	2010	2008	P	audit	India	AH	U	N	30D	0	25	0.000
453	Landry, E.	Assessing the quality of record keeping for cesarean deliveries: results from a multicenter retrospective record review in five low-income countries	OBGYN	CAES	N	2014	2008	R	audit	STRAT	Mixed	Mixed	N	IP	46	2941	0.016
454	Laohawiriyakamol, S.	Surgery in management of snake envenomation in children	PAEDS	VENOM	N	2011	2003	R	audit	Thailand	AH	U	N	IP	0	13	0.000
455	Lashkarizadeh, M.R.	Surgical management of femoral artery pseudoaneurysms secondary to drug abuse	VASCU	VASCU	N	2011	2005	R	nonrandomized cohort	Iran, Islamic Rep.	AH	U	N	IP	1	21	0.048
456	Latipov, R	Childhood intussusception in Uzbekistan: Analysis of retrospective surveillance data	PAEDS	INTUSS	N	2011	2006	R	audit	Uzbekistan	Mixed	Mixed	N	IP	3	34	0.088
457	Leake,P.A.	Open abdominal aortic aneurysm repair in the era of endovascular repair	VASCU	AAA	N	2011	1999	R	nonrandomized cohort	Jamaica	AH	U	Y	30D_IP	5	54	0.093
458	Leake, P.A.	Colorectal Anastomotic Leakage at the University Hospital of theWest Indies An Analysis of Risk Factors	GENSX	COLRES	N	2013	2007	R	audit	Jamaica	AH	U	Y	30D	0	133	0.000
459	Lei, Z	The diagnosis and treatment for hilar cholangiocarcinoma: a report of 136 cases	HEPAT	LIVRES	Y	2012	2000	R	audit	China	AH	U	Y	IP	0	136	0.000
460	Lenzi, A	Hospital mortality in surgery for right ventricular outflow tract reconstruction using pulmonary homograft	CARDI	CCHD	N	2010	2001	R	audit	Brazil	AH	U	Y	IP	17	92	0.185
461	Leon-Wyss, J.	Persistent institutional difficulties in surgery for transposition of the great arteries in Guatemala: analysis with the Aristotle basice and comprehensive scores	CARDI	CCHD	N	2011	2003	R	audit	Guatemala	Mixed	Mixed	Y	IP	31	79	0.392
462	Leon-Wyss, J.R.	Pediatric cardiac surgery: a challenge and outcome analysis of the Guatemala effort.	CARDI	PCARD	N	2009	2002	R	audit	Guatemala	AH	U	Y	IP	218	2630	0.083
463	Letchumanan, VP	Diagnosis and management of ruptured hepatoma: single center experience over 10 years	HEPAT	RHEPAT	N	2013	2005	R	audit	Malaysia	DH	Mixed	Y	30D	1	20	0.050
464	Lewis, A	Genitourinary Fistula Experience in Sierra Leone: Review of 505 Cases	OBGYN	VVF	N	2009	2005	R	audit	Sierra Leone	DH	U	Y	IP	1	505	0.002
465	Li, B.	Left Thoracoabdominal Approach in Surgical Treatment of Adenocarcinoma of the Esophagogastric Junction in the Northern Henan Province of China	THORA	ESOCA	N	2011	2005	R	audit	China	AH	U	Y	IP	0	135	0.000
466	Li C	Risk factors for predicting postoperative complications after open infrarenal abdominal aortic aneurysm repair: results from a single vascular center in China	VASCU	AAA	N	2013	2007	R	audit	China	AH	U	Y	30D	28	316	0.089
467	Li, J.	Microneurosurgical management of anterior choroidal artery aneurysms: a 16-year institutional experience of 102 patients	NEURO	ANEUR	N	2012	2002	R	audit	China	AH	U	N	IP	7	102	0.069
468	Li, J.	Comparison of open and laparoscopic preperitoneal repair of groin hernia	GENSX	INGHERN	N	2013	2009	R	nonrandomized cohort	China	AH	U	Y	30D	1	1760	0.001
469	Li, M.	Single-staged anterior and posterior spinal fusion: A safe and effective alternative for severe and rigid adolescent idiopathic scoliosis in China	NEURO	SPINE	N	2009	2002	R	audit	China	AH	U	Y	30D	0	31	0.000

Reference	First Author	Article Title	Specialty	Procedure or Diagnosis	High-risk population?	Year Published	Data midpoint year	Retrospective or prospective	Study design	Country	Type of Facilities Included	Urban/ Rural/ Mix	Was POMR clearly defined?	Timeframe of POMR	POMR numerator	POMR denomiator	Overall POMR
470	Li, Q	Cross-Sectional Study of Craniocerebral Trauma in a Tertiary Hospital After 2008 Sichuan Earthquake: A Brief Report of 242 Cases and Experiences From West China Hospital	NEURO	ACHI	N	2011	2008	R	audit	China	AH	U	Y	IP	5	50	0.100
471	Li, Y.	The Chinese coronary artery bypass grafting registry study: analysis of the national multicentre database of 9248 patients	CARDI	CABG	N	2009	2004	R	audit	China	Mixed	Mixed	Y	IP	302	9248	0.033
472	Li, Z	Surgical treatment of interrupted aortic arch associated with ventricular septal defect and patent ductus arteriosus in patients over one year of age	CARDI	CCHD	N	2014	2010	R	audit	China	AH	U	Y	IP	2	19	0.105
473	Liang, C	Severe intraoperative complications during VATS Lobectomy compared with thoracotomy lobectomy for early stage non-small cell lung cancer	THORA	LUNGRES	N	2013	2010	R	nonrandomized cohort	China	AH	U	Y	OT	0	659	0.000
474	Liang, X	Effectiveness and Safety of Laparoscopic Resection Versus Open Surgery in Patients with Rectal Cancer: A Randomized, Controlled Trial from China	GENSX	RECTAL	N	2011	2006	R	RCT	China	AH	U	Y	30D	0	343	0.000
475	Limpastan, K.	Factors influencing the outcome of decompressive craniectomy used in the treatment of severe traumatic brain injury	NEURO	ACHI	N	2013	2007	R	audit	Thailand	AH	U	Y	IP	71	159	0.447
476	Lin J-X	Laparoscopy-assisted gastrectomy with D2 lymph node dissection for advanced gastric cancer without serosa invasion: a matched cohort study from South China	GENSX	GASTCA	N	2013	2009	R	nonrandomized cohort	China	AH	U	Y	IP	3	166	0.018
477	Lisboa, L.A.F.	Evolution of cardiovascular surgery at the Instituto do Coracao; analysis of 71,305 surgeries	CARDI	CARD	N	2010	1995	R	audit	Brazil	AH	U	Y	IP	5348	71305	0.075
478	Litorp, H	Maternal near-miss and death and their association with caesarean section complications: a cross-sectional study at a university hospital and a regional hospital in Tanzania	OBGYN	CAES	N	2014	2012	P	audit	Tanzania	Mixed	U	Y	6W	10	2648	0.004
479	Liu, H.	Optimal treatment determination on the basis of haematoma volume and intra-cerebral haemorrhage score in patients with hypertensive putaminal haemorrhages: a retrospective analysis of 310 patients	NEURO	ICH	N	2014	2011	R	audit	China	AH	U	Y	30D	18	129	0.140
480	Liu, S	Comparison of immediate results and mid-term follow-up of surgical and percutaneous closure of ruptured sinus of Valsalva aneurysm	VASCU	CARDAN	N	2014	2003	R	nonrandomized cohort	China	AH	U	Y	IP	1	20	0.050
481	Liu, Y.L.	Safety and Efficacy of Arterial Switch Operation in Previously Inoperable Patients	CARDI	ASO	N	2010	2004	R	audit	China	AH	U	Y	IP	6	86	0.070
482	Lobão, C.A.F.	Comparison between frame-based stereotaxy and neuronavigation in an oncology center	NEURO	ICBX	N	2009	2005	R	audit	Brazil	AH	U	N	IP	2	76	0.026
483	Locali, R.F.	Renal and Adrenal Tumors with Cardiac Invasion: Immediate Surgical Results in 14 Patients	UROLO	TTHR	N	2009	2002	R	audit	Brazil	AH	U	Y	IP	2	14	0.143
484	Lofvenmark I.	Traumatic spinal cord injury in Botswana: characteristics, aetiology and mortality	TRAUM	STRAUM	N	2015	2012	R	audit	Botswana	Mixed	Mixed	Y	IP	10	49	0.204
485	Lohsiriwat V	Outcomes of Local Excision for Early Rectal Cancer: a 6-year Experience from the Largest University Hospital in Thailand	GENSX	RECTAL	N	2013	2007	R	audit	Thailand	AH	U	Y	30D	0	22	0.000
486	Lohsiriwat, V.	Impact of Metabolic Syndrome on the Short-Term Outcomes of Colorectal Cancer Surgery	GENSX	COLRES	N	2010	2007	P	audit	Thailand	AH	U	Y	30D	0	114	0.000
487	Lohsiriwat, V	Enhanced recovery after surgery vs conventional care in emergency colorectal surgery	GENSX	COLRES	N	2014	2012	R	nonrandomized cohort	Thailand	AH	U	Y	30D	0	60	0.000
488	Lone, Y	Outcome of the Surgical Treatment of Bullous Lung Disease: A Prospective Study	THORA	LUNGRES	N	2012	2010	P	audit	India	AH	U	Y	30D	3	54	0.056
489	Long, TC	Laparoscopic liver resection: 5-year experience at a single center	HEPAT	LIVRES	N	2014	2010	P	audit	Vietnam	AH	U	N	IP	0	173	0.000
490	Lopes-Junior, AG	Hepatectomy: A critical analysis on expansion of the indicators	HEPAT	LIVRES	N	2014	2011	P	audit	Brazil	AH	U	Y	30D	5	38	0.132
491	Lopez-Basave, H.	Pelvic exenteration for colorectal cancer: oncologic outcome in 59 patients at a single institution	GENSX	EXENT	N	2012	2002	R	audit	Mexico	AH	U	N	IP	2	59	0.034
492	Lotfy, M.	Decompressive craniotomy after traumatic brain injury: post operative clinical outcome	NEURO	ACHI	N	2010	2006	R	audit	Egypt, Arab Rep.	AH	U	N	IP	6	20	0.300
493	Lourenço, I.L	Pressure support-ventilation versus spontaneous breathing with "T-Tube" for interrupting the ventilation after cardiac operations	CARDI	CARD	N	2013	2011	P	RCT	Brazil	AH	U	Y	IP	0	28	0.000
494	Loveland, J.	A review of paediatric liver resections in Johannesburg: Experiences and preferred technique	HEPAT	LIVRES	N	2012	2008	R	audit	South Africa	AH	U	N	IP	1	21	0.048
495	Lucumay, E	Paediatric neck masses at a University teaching hospital in northwestern Tanzania: a prospective analysis of 148 cases	PAEDS	NECKMA	N	2014	2013	P	audit	Tanzania	AH	U	Y	IP	12	148	0.081
496	Lukong, C. S.	Colostomy in neonates under local anaesthesia: indications, technique and outcome	PAEDS	COLOST	Y	2012	2010	P	audit	Nigeria	AH	U	N	IP	2	38	0.053
497	Lumbiganon, P.	Method of delivery and pregnancy outcomes in Asia: the WHO global survey on maternal and perinatal health 2007–08	OBGYN	CAES	N	2010	2008	R	nonrandomized cohort	Multiple	Mixed	Mixed	Y	IP	35	29428	0.001
498	Luo, G.	Surgical Treatment for Primary Mitral Valve Tumor: A 25-Year Single-Center experience	CARDI	VALVE	N	2011	1996	R	audit	China	AH	U	Y	IP	0	11	0.000
499	Luqman, Z.	Is urgent coronary artery bypass grafting a safe option in octogenarians? A developing country perspective	CARDI	CABG	N	2009	2006	P	audit	Pakistan	AH	U	Y	30D	3	31	0.097
500	Lynch, J.C.	Diagnosis of, surgical technique for and treatment results from medullary lipomas associated with spinal dysraphism	NEURO	RIM	N	2011	1996	R	audit	Brazil	AH	U	N	IP	0	38	0.000
501	Lynch, J.C.	Microsurgical ressection for parasagittal meningiomas with preservation of the parasagittal sinus and excelent neurovascular control	NEURO	RIM	N	2012	1995	R	audit	Brazil	Other	U	N	IP	1	58	0.017
502	Ma, D.	A retrospective study in management of carotid body tumour	ENT	CBT	N	2009	2000	R	audit	China	Mixed	U	Y	IP	0	53	0.000
503	Ma, L. K.	Pregnancy outcomes of repeat cesarean section in Peking Union Medical College Hospital	OBGYN	CAES	N	2009	2002	R	case-control	China	AH	U	N	IP	0	412	0.000

Reference	First Author	Article Title	Specialty	Procedure or Diagnosis	High-risk population?	Year Published	Data midpoint year	Retrospective or prospective	Study design	Country	Type of Facilities Included	Urban/ Rural/ Mix	Was POMR clearly defined?	Timeframe of POMR	POMR numerator	POMR denominator	Overall POMR
504	Ma, LL	Laparoendoscopic Single-Site Radical Cystectomy and Urinary Diversion: Initial Experience in China Using a Homemade Single-Port Device	UROLO	RADCYS	N	2012	2011	R	audit	China	AH	U	N	IP	1	5	0.200
505	Maaloe, N.	Disclosing doubtful indications for emergency cesarean sections in rural hospitals in Tanzania: a retrospective criterion-based audit	OBGYN	CAES	N	2012	2009	R	audit	Tanzania	DH	R	N	IP	4	303	0.013
506	Mabula, J.	Bowel perforation secondary to illegally induced abortion: a tertiary hospital experience in Tanzania	OBGYN	PERF	N	2012	2006	R	audit	Tanzania	AH	U	N	IP	7	68	0.103
507	Mabula, J.	Surgical management of inguinal hernias at Bugando Medical Centre in northwestern Tanzania: our experiences in a resource-limited setting	GENSX	INGHERN	N	2012	2011	P	audit	Tanzania	AH	U	N	IP	44	452	0.097
508	Mabula, J.B.	Predictors of Outcome among patients with Obstructive jaundice at Bugando Medical Centre in north-western Tanzania	HEPAT	BILD	N	2013	2009	P	audit	Tanzania	AH	U	N	IP	28	130	0.215
509	Mabula, J.B.	Hirschsprung's disease in children: a five year experience at a University teaching hospital in northwestern Tanzania	PAEDS	HIRSCH	N	2014	2010	P	audit	Tanzania	AH	U	N	IP	24	110	0.218
510	Mabula, J.	Gastric cancer at a university teaching hospital in northwestern Tanzania: a retrospective review of 232 cases	GENSX	GASTCA	N	2012	2009	R	audit	Tanzania	AH	U	Y	IP	33	223	0.148
511	Machado, M.A.C.	Laparoscopic Pancreatic Resection. From Enucleation To Pancreatoduodenectomy. 11-Year Experience	HEPAT	PANC_RES	N	2013	2006	R	audit	Brazil	AH	U	N	IP	0	96	0.000
512	Machado, M.d.N.	Acute Kidney Injury after On-pump Coronary Artery Bypass Graft Surgery	CARDI	CABG	N	2009	2005	R	nonrandomized cohort	Brazil	DH	Mixed	Y	30D	56	817	0.069
513	Machado, M.N.	Surgical treatment for infective endocarditis and hospital mortality in a Brazilian single-center	CARDI	VALVE	N	2013	2006	R	nonrandomized cohort	Brazil	AH	U	Y	IP_30D	82	837	0.098
514	Madureira, F.A.V.	Quality of life after Heller-Dor's cardiomiotomy	GENSX	HELLER	N	2009	2004	R	audit	Brazil	AH	U	N	IP	0	60	0.000
515	Mahmoudi,S.	Pediatric Hydatidosis in Iranian Referral Pediatrics Center	GENSX	HYDAT	N	2013	2007	R	audit	Iran, Islamic Rep.	AH	U	N	IP	1	17	0.059
516	Malagon Reyes, R.M.	Experience of the MALA Bag in the Open Abdomen Management in an Obstetrical Intensive Care Unit	GENSX	DCL	N	2013	2010	Mixed R/P	audit	Mexico	AH	U	Y	IP	1	18	0.056
517	Malekpour-Afshar, R.	Post Traumatic Meningitis in Neurosurgery Department	NEURO	PTM	N	2009	2005	R	audit	Iran, Islamic Rep.	AH	U	N	IP	19	52	0.365
518	Malhotra, A	Clinical and hemodynamic study of tilting disc heart valve: Single-center study	CARDI	VALVE	N	2014	2008	P	audit	India	AH	U	N	IP	3	200	0.015
519	Malik, A.	Atrial myxoma: An experience in a tertiary care center	CARDI	CARDIACMYX	N	2011	2001	R	nonrandomized cohort	Pakistan	AH	U	N	IP	1	57	0.018
520	Malik, A.A.	Surgical management of complicated hydatid cysts of the liver	HEPAT	LIVHYDAT	N	2010	2004	P	audit	India	AH	U	Y	30D	0	69	0.000
521	Malik, A.A.	Pyogenic liver abscess: Changing patterns in approach	HEPAT	LIVABS	N	2010	2004	P	audit	India	AH	U	Y	30D_IP	12	127	0.094
522	Malik, A.M.	Factors influencing morbidity and mortality in elderly population undergoing inguinal hernia surgery	GENSX	INGHERN	N	2010	2006	R	audit	Pakistan	Mixed	U	N	IP	7	212	0.033
523	Malik, A. M.	Video-assisted laparoscopic extracorporeal appendectomy versus open appendectomy	GENSX	APPY	N	2009	2005	P	nonrandomized cohort	Pakistan	AH	U	N	IP	0	283	0.000
524	Malik, M.	Is EuroSCORE applicable to Indian patients undergoing cardiac surgery?	CARDI	CARD	N	2010	2009	P	audit	India	AH	U	Y	IP	33	1000	0.033
525	Maluf, M.A.	One and a half ventricular repair as an alternative for hypoplastic right ventricle	CARDI	CCHD	N	2010	2005	R	audit	Brazil	AH	U	Y	IP	1	9	0.111
526	Manning, R.G.	Should Laparoscopic Cholecystectomy be Practiced in the Developing World? The Experience of the First Training Program in Afghanistan	GENSX	CHOLE	N	2009	2006	R	audit	Afghanistan	AH	U	N	IP	0	137	0.000
527	Mansour-Ghanaei, F.	Clinical features of hydatid disease in Guilan (the North Province of Iran): A ten-year study	GENSX	HYDAT	N	2012	2005	R	nonrandomized cohort	Iran, Islamic Rep.	CH	U	N	IP	2	61	0.033
528	Marin, F.	GRADE OF ESOPHAGEAL CANCER AND NUTRITIONAL STATUS IMPACT ON POSTSURGERY OUTCOMES	THORA	ESOCA	N	2010	2004	R	nonrandomized cohort	Brazil	AH	U	Y	30D	24	100	0.240
529	Maroof, S. A.	Surgical management of omphalomesenteric duct remnants in children	PAEDS	OMD	N	2009	2006	P	nonrandomized cohort	Pakistan	AH	U	N	IP	2	29	0.069
530	Mataraci, I.	Postoperative Revision Surgery for Bleeding in a Tertiary Heart Center	CARDI	ROHS	N	2010	2002	R	audit	Turkey	AH	U	Y	IP	24	282	0.085
531	Mbamara, S.	An analysis of uterine rupture at the Nnamdi Azikiwe University Teaching Hospital Nnewi, Southeast Nigeria	OBGYN	UTRUP	N	2012	2006	R	audit	Nigeria	AH	U	N	IP	3	25	0.120
532	Medeiros, B.A.	Perineal rectosigmoidectomy on treatment of rectal procidentia: analysis of 48 cases	GENSX	COLRES	N	2012	2005	R	audit	Brazil	AH	U	N	IP	0	48	0.000
533	Medeiros, B. A.	Proctocolectomy and ileal J-pouch anal anastomosis on the surgical treatment of familial adenomatous polyposis and ulcerative colitis: analysis of 49 cases	GENSX	IPAA	N	2012	2010	R	audit	Brazil	AH	U	N	IP	2	49	0.041
534	Mehrabi Bahar, M.	Wound infection incidence in patients with simple and gangrenous or perforated appendicitis	GENSX	APPY	N	2010	2008	R	nonrandomized cohort	Iran, Islamic Rep.	AH	U	N	IP	0	400	0.000
535	Memon, A.A.	An audit of secondary peritonitis at a tertiary care university hospital of Sindh, Pakistan	GENSX	LAPAR	N	2012	2009	R	audit	Pakistan	AH	U	N	IP	52	311	0.167
536	Memon, A. A.	Management of recurrent inguinal hernia at a tertiary care hospital of southern Sindh, Pakistan	GENSX	INGHERN	N	2013	2008	R	audit	Pakistan	AH	U	N	IP	0	62	0.000
537	Memon, A.A.	Traumatic pancreatic injury - an elusive diagnosis: experience from a developing country urban trauma referral centre	TRAUM	PTRAUM	N	2013	1999	R	audit	Pakistan	AH	U	Y	IP	7	30	0.233
538	Memon, A.S.	Management Of Ruptured Amoebic Liver Abscess: 22-Years Experience	HEPAT	LIVAMOEB	N	2010	1996	R	nonrandomized cohort	Pakistan	AH	U	N	IP	6	16	0.375
539	Memon, M.R.	Role of laparoscopy in blunt abdominal trauma	TRAUM	TRAUM	N	2013	2011	P	audit	Pakistan	AH	Mixed	N	IP	0	32	0.000
540	Memon, M.R.	Results of stoppa's sublay mesh repair in incisional and ventral hernias	GENSX	AWH	N	2010	2006	R	audit	Pakistan	Mixed	Mixed	N	IP	1	200	0.005
541	Memon, S.	Pattern of obstructed labour at a public sector university hospital of Sindh, Pakistan	OBGYN	CAES	N	2009	2005	R	audit	Pakistan	AH	Mixed	N	IP	0	36	0.000

Reference	First Author	Article Title	Specialty	Procedure or Diagnosis	High-risk population?	Year Published	Data midpoint year	Retrospective or prospective	Study design	Country	Type of Facilities Included	Urban/ Rural/ Mix	Was POMR clearly defined?	Timeframe of POMR	POMR numerator	POMR denomiator	Overall POMR
542	Menezes, F.H.	Physiologic component of the estimation of physiologic ability and surgical stress scoring system as a predictor of immediate outcome after elective open abdominal aortic aneurysm repair.	VASCU	AAA	N	2011	2004	R	audit	Brazil	AH	U	Y	30D	27	214	0.126
543	Meng, G.	Internal compared with external drainage of pancreatic duct during pancreaticoduodenectomy: a retrospective study	HEPAT	WHIP	N	2014	2005	R	nonrandomized cohort	China	AH	U	Y	30D_IP	13	316	0.041
544	Mesbah, Y.	Emergency peripartum hysterectomy: The experience of a tertiary referral hospital	OBGYN	EPH	N	2012	2010	R	audit	Egypt, Arab Rep.	AH	U	N	IP	4	29	0.138
545	Mezue, W.	Chest injuries associated with head injury	NEURO	ACHI	Y	2012	2006	R	audit	Nigeria	Mixed	Mixed	N	IP	13	30	0.433
546	Mezue, W.C.	Management of Giant Pituitary Tumors Affecting Vision in Nigeria	NEURO	RIM	N	2012	2005	R	audit	Nigeria	AH	U	N	IP	0	20	0.000
547	Mezue, W.	Management of intracranial meningiomas in Enugu, Nigeria	NEURO	RIM	N	2012	2008	Mixed R/P	audit	Nigeria	AH	U	N	IP	2	52	0.038
548	Miah, Z.I.	Outcome of 500 cases of transurethral resection of prostate (TURP) in district level teaching hospital	UROLO	PROST	N	2013	2006	P	audit	Bangladesh	AH	U	N	IP	2	500	0.004
549	Min CM	Why the postoperative mortality rate of gastric cancer is lower in our centre	GENSX	GASTCA	N	2010	2007	R	audit	China	AH	U	Y	IP	2	697	0.003
550	Mir, I.S.	Is laparoscospic orchidectomy the treatment of choice in adults with impalpable testis in rural hospitals in the developingworld?	UROLO	LAPORCH	N	2009	2004	P	nonrandomized cohort	India	CH	R	Y	30D	0	48	0.000
551	Mir MR	Short- and Long-Term Survival of Esophageal Cancer Patients Treated at the Cancer Institute of Iran	THORA	ESOCA	N	2013	2001	R	audit	Iran, Islamic Rep.	AH	U	Y	IP	47	434	0.108
552	Mirhosseini, S.J.	Preoperative C–reactive protein can predict early clinical outcomes following elective off-pump CABG surgery in patients with severe left ventricle dysfunction	CARDI	CABG	Y	2012	2011	P	nonrandomized cohort	Iran, Islamic Rep.	Other	U	Y	IP	2	104	0.019
553	Mirhosseini, S.J.	Effect of Preoperative Anemia on Short Term Clinical Outcomes in Diabetic Patients after Elective Off–Pump CABG Surgery	CARDI	CABG	Y	2012	2011	P	nonrandomized cohort	Iran, Islamic Rep.	AH	U	Y	IP	2	86	0.023
554	Mirshemirani, A	Liver Hydatid Cysts in Children (A 14-year review)	HEPAT	LIVHYDAT	N	2011	2003	R	audit	Iran, Islamic Rep.	AH	U	N	IP	1	100	0.010
555	Mirshemirani, A. R.	Surgical treatment of pulmonary hydatid cyst in 72 children	THORA	THORHYDAT	N	2009	1999	R	nonrandomized cohort	Iran, Islamic Rep.	AH	U	N	30D	0	72	0.000
556	Mirza, B.	Surgical aspects of intestinal tuberculosis in children: our experience	PAEDS	ABDOTB	N	2011	2008	R	audit	Pakistan	AH	U	N	IP	1	15	0.067
557	Mocumbi, A.O.	Challenges on the management of congenital heart disease in developing countries.	CARDI	PCARD	N	2011	2004	R	audit	Mozambique	AH	U	Y	30D	8	196	0.041
558	Mohamed, A.A.	Blunt abdominal trauma requiring laparotomy in polytraumatized patients	TRAUM	LAPAR	N	2010	2007	R	audit	Egypt, Arab Rep.	AH	U	Y	IP	36	94	0.383
559	Mohamed, A.A.	Laparoscopic appendectomy in complicated appendicitis: Is it safe?	GENSX	APPY	N	2013	2008	R	nonrandomized cohort	Egypt, Arab Rep.	AH	U	Y	30D	0	214	0.000
560	Mohanty, N.K.	Analysis of the perioperative and five-year oncological outcome of two hundred cases of open radical cystectomy: A single center experience	UROLO	RADCYS	N	2012	2006	P	audit	India	AH	U	Y	30D_IP	2	200	0.010
561	Moiyadi, A.V.	Perioperative outcomes following surgery for brain tumors: Objective assessment and risk factor evaluation	NEURO	RIM	N	2012	2008	R	audit	India	AH	U	Y	IP	7	196	0.036
562	Molina, G.	Evaluation of Surgical Care in El Salvador Using the WHO Surgical Vital Statistics	MULTI	MULTI	N	2013	2009	R	audit	El Salvador	Mixed	Mixed	Y	IP_30D	483	30616	0.016
563	Monsalve, G.A.	Pregnant patient with cardiac diseases* Risk based peripartum management. Case series 2005-2009	OBGYN	CAES	Y	2010	2007	R	audit	Colombia	AH	U	Y	30D	0	13	0.000
564	Montufar-Rueda, C.	Severe Postpartum Hemorrhage from Uterine Atony: A Multicentric Study	OBGYN	EPH	N	2013	2011	P	audit	Multiple	Mixed	Mixed	N	IP	7	52	0.135
565	Moodley, J.	Breech presentation at a district level hospital in South Africa	OBGYN	CAES	N	2010	2006	R	audit	South Africa	AH	U	N	IP	0	382	0.000
566	Morales, C.	Efficacy and safety of non-operative management of blunt liver trauma	TRAUM	LIVTRAUM	N	2011	2006	Mixed R/P	audit	Colombia	AH	U	Y	IP	3	14	0.214
567	Morrison, B.	Radical Prostatectomy Outcomes at the University Hospital of the West Indies: 2000–2007	UROLO	PROST	N	2011	2003	R	audit	Jamaica	AH	U	N	IP	0	116	0.000
568	Mosaddeghi, K.S.	Capsulorrhaphy in the management of liver hydatid cyst	HEPAT	LIVHYDAT	N	2014	2000	R	nonrandomized cohort	Iran, Islamic Rep.	Other	U	Y	30D	0	250	0.000
569	Mozafar, M.	Trans-thoracic versus trans-hiatal esophagectomy complications and outcomes in patients with esophageal cancer in Shohada-E-Tajrish Hospital, Tehran, Iran	THORA	ESOCA	N	2010	2004	R	nonrandomized cohort	Iran, Islamic Rep.	AH	U	Y	30D	5	100	0.050
570	Mpabalwani, E.M.	Intussusception incidence rates in 9 Zambian hospitals, 2007-2011: Prerotavirus vaccine introduction	PAEDS	INTUSS	N	2014	2009	Mixed R/P	nonrandomized cohort	Zambia	Mixed	Mixed	N	IP	31	92	0.337
571	Mukasa, P.K.	Uterine rupture in a teaching hospital in Mbarara, western Uganda, unmatched case-control study	OBGYN	UTRUP	N	2013	2005	R	case-control	Uganda	AH	U	N	IP	10	77	0.130
572	Mulaudzi, TV	The influence of diabetes on short-term outcome following a prosthetic above-the-knee femoro-popliteal bypass	VASCU	BYPASS	N	2009	2003	R	nonrandomized cohort	South Africa	AH	U	Y	30D	5	217	0.023
573	Mumtaz, K.	Hepaticcystic echinococcosis:clinical characteristics and outcomesinPakistan	HEPAT	LIVHYDAT	N	2009	2000	R	audit	Pakistan	AH	U	N	IP	2	71	0.028
574	Murtaza, B.	Postoperative Complications In Emergency Versus Elective Laparotomies At A Peripheral Hospital	GENSX	LAPAR	N	2010	2007	P	audit	Pakistan	AH	U	Y	30D	7	104	0.067
575	Mushtaque, M.	Pellet gunfire injuries among agitated mobs in Kashmir	TRAUM	GSW	N	2012	2010	R	audit	India	AH	U	N	IP	6	43	0.140
576	Mutyaba, A.K	Constrictive pericarditis requiring pericardiectomy at Groote Schuur Hospital, Cape Town, South Africa: Causes and perioperative outcomes in the HIV era (1990-2012)	CARDI	PERICAR	N	2014	2001	R	audit	South Africa	AH	U	Y	IP_30D	17	121	0.140
577	Muyembe-Tamfum, J.J.	An outbreak of peritonitis caused by multidrug- resistant Salmonella Typhi in Kinshasa, Democratic Republic of Congo	GENSX	TIP	N	2008	2004	R	audit	Congo, Dem. Rep.	Mixed	Mixed	N	IP	6	41	0.146
578	Najam, R.	Emergency obstetric hysterectomy: a retrospective study at a tertiary care hospital.	OBGYN	EPH	N	2010	2008	R	audit	India	AH	U	N	IP	3	24	0.125
579	Narasimman S	Review of Oesophageal Atresia and Tracheoesophageal Fistula in Hospital Sultanah Bahiyah, Alor Star. Malaysia	PAEDS	EATEF	N	2013	2004	R	audit	Malaysia	DH	U	N	IP	11	47	0.234
580	Nasio, N.A.	Perforated Peptic Ulcer Disease at Kenyatta National Hospital, Nairobi	GENSX	PERF	N	2009	2005	R	audit	Kenya	AH	U	N	IP	4	28	0.143

Reference	First Author	Article Title	Specialty	Procedure or Diagnosis	High-risk population?	Year Published	Data midpoint year	Retrospective or prospective	Study design	Country	Type of Facilities Included	Urban/ Rural/ Mix	Was POMR clearly defined?	Timeframe of POMR	POMR numerator	POMR denominator	Overall POMR
581	Nasir, A.A.	Outcomes of surgical treatment of malrotation in children	PAEDS	MALRO	N	2011	2004	R	audit	Nigeria	AH	U	Y	30D	2	9	0.222
582	Nasir, A.A.	Predictor of mortality in children with typhoid intestinal perforation in a Tertiary Hospital in Nigeria	PAEDS	TIP	N	2011	2005	R	audit	Nigeria	AH	U	N	IP	16	153	0.105
583	Nassar, O.A.H.	Modified Pseudocontinent Perineal Colostomy: A Special Technique	GENSX	APR	N	2011	2005	P	nonrandomized cohort	Egypt, Arab Rep.	AH	U	Y	30D	0	14	0.000
584	Nazeem, W.M.	Intracranial Meningiomas in Children: Surgical Experience	NEURO	RIM	N	2012	2011	R	audit	Egypt, Arab Rep.	AH	U	Y	30D	0	9	0.000
585	Nazem, M.	Evaluation of early and late complications in patients with congenital lobar emphysema: A 12 year experience	PAEDS	CLE	N	2010	2002	P	audit	Iran, Islamic Rep.	AH	U	N	IP	4	26	0.154
586	Nega, B.	Pattern of acute abdomen and variables associated with adverse out come in rural primary hospital setting	GENSX	AABDO	N	2009	2005	P	audit	Ethiopia	CH	R	N	IP	7	143	0.049
587	Nega, B.	Truncal Vagotomy for Peptic Pyloric Stenosis and Assessment of Completeness by Acid Tests	GENSX	GASTROJ	N	2010	2005	P	audit	Ethiopia	AH	U	N	IP	0	32	0.000
588	Neumann, CR	Risk Factors for Mortality in Traumatic Cervical Spinal Cord Injury: Brazilian Data	TRAUM	STRAUM	N	2009	2003	R	audit	Brazil	AH	U	Y	IP	2	42	0.048
589	Nguyen, H.S.	A Modified Carpentier's Technique for Ebstein's Anomaly Repair	CARDI	MCHD	N	2014	2008	R	audit	Vietnam	Other	U	Y	30D	1	52	0.019
590	Nikibakhsh,A.	Outcome of Immediate Use of the Permanent Peritoneal Dialysis Catheter in Childrenwith Acute and Chronic Renal Failure	GENSX	PTCATH	N	2013	2008	P	audit	Iran, Islamic Rep.	AH	U	N	30D	5	56	0.089
591	Niramis, R.	Influence of Down's syndrome on management and outcome of patients with congenital intrinsic duodenal obstruction.	PAEDS	BOBS	N	2010	2001	R	audit	Thailand	AH	U	Y	30D	21	227	0.093
592	Nisar, N.	Emergency Peripartum Hysterectomy: Frequency, Indications And Maternal Outcome	OBGYN	EPH	N	2009	2006	R	audit	Pakistan	AH	U	N	IP	4	21	0.190
593	Nizam, K.	Cesarean section rate: Much room for reduction	OBGYN	CAES	N	2010	2008	P	nonrandomized cohort	Pakistan	AH	U	N	IP	0	656	0.000
594	Nogueira, L.	Cutaneous ureterostomy with definitive ureteral stent as urinary diversion option in unfit patients after radical cystectomy	UROLO	URETEROST	N	2013	2007	R	audit	Brazil	AH	U	N	IP	0	41	0.000
595	Nooraei, N.	Preoperative assessment of mechanical ventilation requirement after surgical treatment of esophageal cancer	THORA	ESOCA	N	2010	2004	R	nonrandomized cohort	Iran, Islamic Rep.	AH	U	Y	IP	17	119	0.143
596	Nooren, M.	Obstetric hysterectomy: a life saving emergency	OBGYN	EPH	N	2013	2008	R	audit	India	AH	U	N	IP	3	20	0.150
597	Novaes, F.R.	Results of Castro Bernardes intraluminal ring in surgery for ascending aortic aneurysms and dissections	CARDI	TAD	N	2013	2010	R	audit	Brazil	AH	U	N	IP	15	95	0.158
598	Nuhu, A.	Operative management of typhoid ileal perforation in children	PAEDS	TIP	N	2010	2006	R	audit	Nigeria	DH	U	N	IP	13	46	0.283
599	Nuhu, I.	Acute sigmoid volvulus in a West African population	GENSX	VOLV	N	2010	2002	R	audit	Gambia, The	AH	U	N	IP	5	48	0.104
600	Nuhu, A.	Acute perforated duodenal ulcer in Maiduguri: experience with simple closure and Helicobacter pylori eradication	GENSX	PERF	N	2009	2003	R	audit	Nigeria	AH	U	N	IP	9	55	0.164
601	Nuhu, A.	Day case haemorrhoidectomy in a developing country	GENSX	HEMOR	N	2009	2004	R	nonrandomized cohort	Nigeria	AH	U	N	IP	0	43	0.000
602	Nunes, J. C.	Intraoperative and Anesthesia-Related Cardiac Arrest and Its Mortality in Older Patients: A 15-Year Survey in a Tertiary Teaching Hospital	MULTI	MULTI	N	2014	2003	R	audit	Brazil	AH	U	Y	OT	68	18367	0.004
603	Nwafor, M. I.	Perinatal outcome of preterm cesarean section in a resource-limited centre: a comparison between general anaesthesia and subarachnoid block	OBGYN	CAES	N	2014	2003	R	nonrandomized cohort	Nigeria	AH	U	N	IP	0	236	0.000
604	Nwankwo, O.E.	Epidemiological and treatment profiles of spinal cord injury in southeast Nigeria	NEURO	STRAUM	N	2013	2011	R	audit	Nigeria	AH	U	N	IP	1	17	0.059
605	Nwobodo, E.I.	Emergency Obstetric Hysterectomy in a Tertiary Hospital in Sokoto, Nigeria	OBGYN	EPH	N	2012	2007	R	audit	Nigeria	AH	U	N	IP	9	74	0.122
606	Nwobodo, E.	Elective caesarean section in a tertiary hospital in Sokoto, north western Nigeria	OBGYN	CAES	N	2011	2006	R	audit	Nigeria	AH	U	N	IP	18	2284	0.008
607	Nyamtema, A.S.	Tanzanian lessons in using non-physician clinicians to scale up comprehensive emergency obstetric care in remote and rural areas	OBGYN	OBGYNE	N	2011	2009	P	audit	Tanzania	DH	Mixed	N	IP	0	278	0.000
608	Nyengidiki, T.K.	Rupture of the gravid uterus in a tertiary health facility in the Niger delta region of Nigeria: a 5-year review	OBGYN	UTRUP	N	2011	2005	R	audit	Nigeria	AH	U	N	IP	7	40	0.175
609	Obalum, D.	Lower limb amputations at a Nigerian Private Tertiary hospital	ORTHO	AMPUT	N	2009	2001	R	audit	Nigeria	AH	U	N	IP	3	64	0.047
610	Obeidat, B.	Tubal ectopic pregnancy in the north of Jordan: Presentation and management	OBGYN	ECTOP	N	2010	2005	R	nonrandomized cohort	Jordan	AH	U	N	IP	0	50	0.000
611	Obiechina, N.J.A.	Emergency peripartum hysterectomy in Nnewi, Nigeria: A 10-year review	OBGYN	EPH	N	2012	2004	R	audit	Nigeria	AH	U	N	IP	9	29	0.310
612	Obiechina, N.J.A.	Vaginal Hysterectomy in a Nigerian Tertiary Health Facility	OBGYN	HYST	N	2010	2002	R	audit	Nigeria	AH	U	N	IP	0	47	0.000
613	Ogelle, O.	Current Trends in Hysterectomies at a Nigerian Tertiary Center	OBGYN	HYST	N	2010	2004	R	audit	Nigeria	AH	U	N	IP	3	72	0.042
614	Ogundoyin, O.O.	Pattern and outcome of childhood intestinal obstruction at a Tertiary Hospital in Nigeria	PAEDS	BOBS	N	2009	2000	R	audit	Nigeria	AH	U	N	IP	4	130	0.031
615	Ohene-Yeboah, M.	Penetrating Abdominal Injuries In Adults Seen At Two Teaching Hospitals In Ghana	TRAUM	LAPAR	N	2010	2003	Mixed R/P	audit	Ghana	AH	U	N	IP	18	331	0.054
616	Okafor, U.	A review of anaesthesia for emergency laparotomy in paediatric intestinal obstruction	PAEDS	BOBS	N	2009	2008	R	audit	Nigeria	AH	U	N	IP	1	44	0.023
617	Okafor, U.V.	Anaesthetic and Obstetric challenges of morbid obesity in caesarean deliveries-a study in South-eastern Nigeria	OBGYN	CAES	Y	2012	2009	R	audit	Nigeria	AH	U	N	IP	0	31	0.000
618	Okafor, U	Trends of different forms of anaesthesia for caesarean section in South-eastern Nigeria	OBGYN	CAES	N	2009	2004	R	audit	Nigeria	AH	U	N	IP	1	729	0.001
619	Okafor, U.V.	Anaesthetic challenges in emergency peripartum hysterectomy in West Africa: a Nigerian perspective	OBGYN	EPH	N	2010	2002	R	audit	Nigeria	AH	U	N	IP	2	16	0.125
620	Okonta, K.E.	Intra-aortic balloon pump in coronary artery bypass graft - factors affecting outcome	CARDI	CABG	Y	2011	2009	R	audit	India	Other	U	Y	IP_30D	47	107	0.439
621	Okorie, C.	Modifying and increasing day-case procedures to solve local problems: experience of a urology unit	UROLO	UROL	N	2012	2010	P	audit	Nigeria	Mixed	Mixed	N	IP	0	71	0.000

Reference	First Author	Article Title	Specialty	Procedure or Diagnosis	High-risk population?	Year Published	Data midpoint year	Retrospective or prospective	Study design	Country	Type of Facilities Included	Urban/ Rural/ Mix	Was POMR clearly defined?	Timeframe of POMR	POMR numerator	POMR denomiator	Overall POMR
622	Okoro, P. E., Igwe, P., Op	Pattern and survival of biliary atresia patients; experience in southern Nigeria	HEPAT	BILAT	N	2013	2009	P	audit	Nigeria	AH	U	N	IP	2	15	0.133
623	Oladapo, O.T.	Relative morbidity of abdominal myomectomy for very large uterine fibroids in a developing country hospital	OBGYN	MYOMEC	N	2011	2004	R	nonrandomized cohort	Nigeria	AH	U	N	IP	0	224	0.000
624	Olamijulo, J.	Emergency Obstetric Hysterectomy in a Nigerian teaching hospital: a ten-year review	OBGYN	EPH	N	2013	2001	R	case-control	Nigeria	AH	U	N	IP	4	34	0.118
625	Olavarrieta, JRL	Expectations and patient satisfaction related to the use of thoracotomy and video-assisted thoracoscopic surgery for treating recurrence of spontaneous primary pneumothorax*	THORA	LUNGRES	N	2009	1996	R	nonrandomized cohort	Venezuela, RB	AH	U	N	IP	0	100	0.000
626	Olayiwola, B.	Day-case herniotomy surgery for children with inhalational anaesthesia in Lagos, Nigeria	PAEDS	INGHERN	N	2011	2004	R	audit	Nigeria	AH	U	N	IP	0	181	0.000
627	Oliveira Junior, J.L.	Does the coronary disease increase the hospital mortality in patients with aortic stenosis undergoing valve replacement?	CARDI	CARD	Y	2009	2003	P	nonrandomized cohort	Brazil	AH	U	Y	IP	93	615	0.151
628	Oliveira M	Programmable valve represents an efficient and safe tool in the treatment of idiopathic normal-pressure hydrocephalus patients	NEURO	HYDRO	N	2012	2012	P	nonrandomized cohort	Brazil	AH	U	N	IP	1	24	0.042
629	Oliveira, T.M.L.	Mortality and Complications of Coronary Artery Bypass Grafting in Rio de Janeiro, from 1999 to 2003	CARDI	CABG	N	2010	2001	R	nonrandomized cohort	Brazil	Mixed	Mixed	Y	30D	67	546	0.123
630	Omisanjo, O.A.	Clinical characteristics and outcome of management of Fournier's gangrene at the Lagos State University Teaching Hospital, Ikeja, Lagos, Nigeria	UROLO	FOURN	N	2014	2010	R	audit	Nigeria	AH	U	N	IP	0	11	0.000
631	Omole-Ohonsi, A.	Non-emergency hysterectomy: why the aversion?	OBGYN	HYST	N	2009	2002	P	audit	Nigeria	AH	U	N	IP	0	134	0.000
632	Omole-Ohonsi, A	Surgical Management of Uterine Fibroids at Aminu Kano Teaching Hospital	OBGYN	MYOMEC	N	2011	2005	R	audit	Nigeria	AH	U	N	IP	0	105	0.000
633	Omole-Ohonsi, A.	Emergency Peripartum Hysterectomy in a Developing Country	OBGYN	EPH	N	2012	2005	R	case-control	Nigeria	AH	U	N	IP	4	30	0.133
634	Onan, B.	Cardiac and great vessel injuries after chest trauma: our 10-year experience	TRAUM	CTRAUM	N	2011	2004	R	audit	Turkey	AH	U	Y	IP	19	104	0.183
635	Onan SI	Clinical Outcome of Patients in a Start-Up Congenital Heart Surgery Program in Turkey	CARDI	PCARD	N	2013	2011	R	audit	Turkey	DH	U	Y	IP	66	616	0.107
636	Onat, S.	Urgent thoracotomy for penetrating chest trauma: Analysis of 158 patients of a single center	TRAUM	THORTRAUM	N	2010	2004	R	audit	Turkey	AH	U	N	IP	17	158	0.108
637	Onder, A.	Associated organ injuries in pancreatic injuries, morbidity, and mortality	TRAUM	PTRAUM	N	2012	2007	R	audit	Turkey	AH	U	N	IP	3	26	0.115
638	Oneko, O.	Management of the Placenta in Advanced Abdominal Pregnancies at an East African Tertiary Referral Center	OBGYN	ABDOPR	N	2010	2002	R	audit	Tanzania	AH	U	N	IP	0	9	0.000
639	Onyebuchi, A.	Determinants of decision-to-intervention time in the management and therapeutic outcome of emergency gynecological surgeries in south east Nigeria	OBGYN	EGYNE	N	2014	2012	P	audit	Nigeria	AH	U	Y	IP	6	105	0.057
640	Opara, P.I	Surgical admissions in a newborn unit in a low resource setting, challenges in management and outcomes	PAEDS	PAED	N	2014	2011	R	audit	Nigeria	AH	U	N	IP	19	60	0.317
641	Oral, A.	Diagnosis and management of hydatid liver disease in children: a report of 156 patients with hydatid disease	HEPAT	LIVHYDAT	N	2012	2002	R	audit	Turkey	AH	U	N	IP	0	105	0.000
642	Ordonez, C.A.	Damage control resuscitation: Early decision strategies in abdominal gunshot wounds using an easy ''ABCD'' mnemonic	TRAUM	DCL	N	2012	2006	P	audit	Colombia	AH	U	N	IP	37	331	0.112
643	Oribabor FO	Perforated Duodenal Ulcer; Management in a Resource Poor, Semi-Urban Nigerian Hospital	GENSX	PERF	N	2013	2007	R	audit	Nigeria	DH	U	N	IP	4	30	0.133
644	OSAIKHUWUOMWAN, J.A	Reappraisal of ruptured uterus in an urban tertiary center in the Niger-delta region of Nigeria	OBGYN	UTRUP	N	2011	2005	R	audit	Nigeria	AH	U	N	IP	2	33	0.061
645	Osifo, O.D.	Challenges of giant ventral hernia repair in children in an African tertiary care center with limited resources	PAEDS	AWH	N	2008	2002	R	audit	Nigeria	AH	U	N	IP	2	41	0.049
646	Osifo, O.D.	Primary perineal surgeries for the low and intermediate anorectal anomalies: 5-year results in a developing country	PAEDS	ANOMAL	N	2008	2005	R	nonrandomized cohort	Nigeria	AH	U	N	IP	1	29	0.034
647	Osifo, O.D.	Hypospadias repair in a resource-poor region: Coping with the challenges in 5 years	UROLO	HYPOS	N	2010	2005	P	audit	Nigeria	AH	U	N	IP	0	127	0.000
648	Osifo, O. D.	Appendicitis in children: An increasing health scourge in a developing country	GENSX	APPY	N	2009	2002	R	audit	Nigeria	AH	U	N	IP	1	481	0.002
649	Osifo, O.D.	Peritonitis in Children: Our Experience in Benin City, Nigeria	PAEDS	LAPAR	N	2011	2006	P	audit	Nigeria	AH	U	N	IP	18	182	0.099
650	Osifo, O. D.	Neonatal intestinal obstruction in Benin, Nigeria	PAEDS	BOBS	N	2009	2007	P	audit	Nigeria	AH	U	N	IP	13	58	0.224
651	Osifo, O. D.	Omphalocele management using goal-oriented classification in African centre with limited resources	PAEDS	OMPHAL	N	2011	2004	P	audit	Nigeria	AH	U	N	IP	20	82	0.244
652	Osifo, O.D.	The Predictors, Prevalence and Outcome of Burst Abdomen in Emergency Paediatric Surgical Centre.	GENSX	EVISC	N	2010	2005	R	audit	Nigeria	Mixed	U	N	IP	14	31	0.452
653	Osifo, O.D.	Duodenal obstruction: etiology, morbidity and mortality among edo state children, Nigeria	PAEDS	BOBS	Y	2009	2002	R	audit	Nigeria	AH	U	N	IP	24	63	0.381
654	Oucheng, N.	Frontoethmoidal meningoencephalocele: appraisal of 200 operated cases	NEURO	FEME	N	2010	2007	P	audit	Cambodia	AH	U	N	IP	4	200	0.020
655	Ozdemir, A.C.	Bileaflet versus Posterior-Leaflet-Only Preservation in Mitral Valve Replacement	CARDI	VALVE	N	2014	2010	R	nonrandomized cohort	Turkey	AH	U	Y	IP	1	70	0.014
656	Ozer, I.	Surgical treatment for gastric cancer in Turkish patients over age 70: early postoperative results and risk factors for mortality	GENSX	GASTCA	N	2010	2004	P	audit	Turkey	AH	U	Y	30D	26	549	0.047
657	Ozogul, Y.B.	Short-term outcomes after surgery for colorectal cancer in Turkish patients aged 70 and above	GENSX	COLRES	N	2010	2004	P	audit	Turkey	AH	U	Y	30D_IP	18	660	0.027
658	Ozyurtkan, M.O.	Surgical Treatment of Intrathoracic Hydatid Disease: A 5-Year Experience in an Endemic Region	THORA	THORHYDAT	N	2009	2006	R	audit	Turkey	AH	U	N	30D	0	40	0.000
659	Pais-Costa, S.R.	Laparoscopic hepatectomy: indications and results from 18 resectable cases	HEPAT	LIVRES	N	2011	2009	R	audit	Brazil	Mixed	Mixed	N	IP	0	18	0.000

Reference	First Author	Article Title	Specialty	Procedure or Diagnosis	High-risk population?	Year Published	Data midpoint year	Retrospective or prospective	Study design	Country	Type of Facilities Included	Urban/ Rural/ Mix	Was POMR clearly defined?	Timeframe of POMR	POMR numerator	POMR denomiator	Overall POMR
660	Paiva-Neto, M.A.	Supra-orbital keyhole removal of anterior fossa and parasellar meningiomas	NEURO	RIM	N	2010	2004	R	audit	Brazil	Mixed	U	N	30D	1	24	0.042
661	Pal, K.	Pancreaticoduodenectomy: A developing country perspective	HEPAT	WHIP	N	2011	1997	R	audit	Pakistan	AH	U	Y	30D	12	121	0.099
662	Pan, X.	Comparison of short and long-term results between sleeve resection and pneumonectomy in lung cancer patients over 70 years old: 10 years experience from a single institution in China	THORA	LUNGRES	N	2014	2007	R	nonrandomized cohort	China	AH	U	Y	IP	3	105	0.029
663	Pande, S.	Early and mid-term results of minimally invasive coronary artery bypass grafting	CARDI	CABG	N	2014	2010	R	audit	India	AH	U	Y	30D	0	33	0.000
664	Pardhan, A.	Outcomes of Splenectomy for Idiopathic Thrombocytopenic Purpura in adults: A Developing Country Perspective	GENSX	SPLEEN	N	2014	2005	R	audit	Pakistan	AH	U	N	IP	0	27	0.000
665	Parsak, C.K.	Reconstruction of Cervical Esophagus Defects by Free Jejunal Flap After Proximal Esophageal Carcinoma Resections	THORA	ESOCA	Y	2011	2008	R	audit	Turkey	AH	U	N	IP	1	9	0.111
666	Parveen, Z.	Palliative Surgery For Intestinal Obstruction Due To Recurrent Ovarian Cancer	OBGYN	PALL	N	2009	2003	R	audit	Pakistan	AH	U	Y	30D_IP	12	30	0.400
667	Paulista, M.D.	Surgical treatment of partial anomalous pulmonary venous connection to the superior vena cava	CARDI	MCHD	N	2009	1997	R	audit	Brazil	AH	U	Y	IP	0	95	0.000
668	Peer, S.M.	Traumatic diaphragmatic hernia-our experience	TRAUM	TDH	N	2009	2003	R	audit	India	AH	U	N	IP	4	29	0.138
669	Pei, G.	Risk factors for postoperative complications after lung resection for non-small cell lung cancer in elderly patients at a single institution in China	THORA	LUNGRES	N	2014	2008	P	case-control	China	AH	U	Y	30D_IP	11	476	0.023
670	Pembe, A.B.	Pregnancy outcome after one previous caesarean section at a tertiary university teaching hospital in Tanzania	OBGYN	CAES	N	2010	2006	P	audit	Tanzania	AH	U	N	IP	1	306	0.003
671	Pembe, A.B.	Emergency peripartum hysterectomies at Muhimbili National Hospital, Tanzania: a review of cases from 2003 to 2007	OBGYN	EPH	N	2012	2005	R	audit	Tanzania	AH	U	N	IP	17	165	0.103
672	Pereira, C.	Emergency obstetric surgery by non-physician clinicians in Tanzania	OBGYN	OBGYNE	N	2011	2003	R	audit	Tanzania	Mixed	Mixed	Y	IP	147	8257	0.018
673	Pereira, L.C.M	Outcome of fully awake craniotomy for lesions near the eloquent cortex: analysis of a prospective surgical series of 79 supratentorial primary brain tumors with long follow-up	NEURO	RIM	N	2009	2002	P	audit	Brazil	AH	U	Y	30D	1	79	0.013
674	Pereira, S.R.M.	The Impact of Prefracture and Hip Fracture Characteristics on Mortality in Older Persons in Brazil	ORTHO	HIPFRAC	N	2010	2001	P	audit	Brazil	CH	U	Y	IP_30D	22	246	0.089
675	Perveen, F.	Use of bilateral internal iliac artery ligation for controlling severe obstetric haemorrhage	OBGYN	BIAL	N	2011	2009	R	audit	Pakistan	AH	U	N	IP	1	8	0.125
676	Phadungkiatwattana, P	Analyzing the impact of private service on the cesarean section rate in public hospital Thailand.	OBGYN	CAES	N		2007	R	nonrandomized cohort	Thailand	AH	U	N	IP	0	2841	0.000
677	Piegas, Leopoldo S.	Myocardial revascularization surgery (MRS): results from National Health System (SUS)	CARDI	CABG	N	2009	2006	R	audit	Brazil	Mixed	Mixed	Y	IP	3934	63272	0.062
678	Pires de Aguiar, P.	Interventricular meningiomas in adults- clinical series and review of the literature	NEURO	RIM	N	2011	2003	R	audit	Brazil	AH	U	N	IP	0	13	0.000
679	Pires, LV	Role of Hysterectomy in the Management of Patients with Gestational Trophoblastic Neoplasia Importance of Receiving Treatment in Reference Centers	OBGYN	HYST	Y	2012	1995	R	nonrandomized cohort	Brazil	Mixed	Mixed	Y	IP	4	57	0.070
680	Pitcher, G.J.	Fetal extraperitoneal rectal perforation: a rare neonatal emergency	PAEDS	PERF	N	2008	1993	R	audit	South Africa	AH	U	N	30D	0	9	0.000
681	Pivatto Junior, F.	Morbimortality in Octogenarian Patients Submitted to Myocardial Revascularization Surgery	CARDI	CABG	N	2010	2004	R	audit	Brazil	AH	U	Y	IP	20	140	0.143
682	Pokharel, N.	Acute appendicitis in elderly patients: a challenge for surgeons	GENSX	APPY	N	2011	2011	R	audit	Nepal	AH	U	N	IP	2	200	0.010
683	Poletti, G.B.	Descriptive analysis of and overall survival after surgical treatment of lung metastases*	THORA	LUNGRES	N	2013	2004	R	audit	Brazil	AH	U	Y	30D	3	154	0.019
684	Pomerantzeff, P.M.A.	Mitral valve repair in patients with rheumatic mitral insufficiency. Twenty years of techniques and results	CARDI	VALVE	N	2009	1995	R	audit	Brazil	AH	U	Y	IP	3	330	0.009
685	Pourzand, A.	Management of high-risk popliteal vascular blunt trauma: clinical experience with 62 cases	TRAUM	PVI	N	2010	2006	P	nonrandomized cohort	Iran, Islamic Rep.	AH	U	Y	IP	7	62	0.113
686	Pradhan, G. B.	Inguinal herniotomy in children: a one year survey at Nepal Medical College Teaching Hospital	GENSX	INGHERN	N	2011	2008	P	audit	Nepal	AH	U	Y	7D	0	61	0.000
687	Pradhan, M.	Emergency Peripartum Hysterectomy as Postpartum Hemorrhage Treatment: Incidence, Risk factors, and Complications	OBGYN	EPH	N	2014	2009	R	audit	China	AH	U	Y	IP	0	61	0.000
688	Qadir, I.	Risk stratification analysis of operative mortality in isolated coronary artery bypass graft patients in Pakistan: comparison between additive and logistic EuroSCORE models	CARDI	CABG	N	2011	2008	R	audit	Pakistan	AH	U	Y	30D	76	2004	0.038
689	Qadir, I.	Mortality from isolated coronary bypass surgery: a comparison of the Society of Thoracic Surgeons and the EuroSCORE risk prediction algorithms	CARDI	CABG	N	2012	2010	R	audit	Pakistan	AH	U	Y	30D	11	380	0.029
690	Qamarunisa, H	Frequency, Maternal And Fetal Outcome Of Abruptio Placenta In A Rural Medical College Hospital, Mirpurkhas Sindh	OBGYN	CAES	Y	2010	2007	P	audit	Pakistan	AH	R	N	IP	0	21	0.000
691	Qazi, Q.	Clinical presentations and complications associated with tubal rupture in patients with tubal ectopic pregnancy	OBGYN	ECTOP	N	2010	2003	R	nonrandomized cohort	Pakistan	AH	U	N	IP	0	50	0.000
692	Qiao, G.	Laparoscopic cyst excision and Roux-Y hepaticojejunostomy for children with choledochal cysts in China: a multicenter study	HEPAT	CDC	N	2015	2006	R	audit	China	AH	U	Y	30D	2	956	0.002
693	Qiao, Q.	Prognostic Factors after Pancreatoduodenectomy for Distal Bile Duct Cancer	HEPAT	WHIP	N	2011	2003	R	audit	China	AH	U	Y	IP	8	122	0.066
694	Han, Q.	Reinforced aortic root reconstruction for acute type A aortic dissection involving the aortic root	CARDI	TAD	N	2013	2003	R	audit	China	Mixed	Mixed	Y	IP	10	161	0.062

Reference	First Author	Article Title	Specialty	Procedure or Diagnosis	High-risk population?	Year Published	Data midpoint year	Retrospective or prospective	Study design	Country	Type of Facilities Included	Urban/ Rural/ Mix	Was POMR clearly defined?	Timeframe of POMR	POMR numerator	POMR denomiator	Overall POMR
695	Queiroz, A.B.	Repair of ruptured abdominal aortic aneurysms with bifurcated endografts: a single-center study	VASCU	AAA	N	2014	2012	P	audit	Brazil	AH	U	Y	30D	5	10	0.500
696	Raagab, A.	Re-laparotomy after Cesarean Section: Risk, Indications and Management options	OBGYN	RELAPCS	N	2014	2010	P	case-control	Egypt, Arab Rep.	AH	U	N	IP	3	26	0.115
697	Rabiu KA	Comparison of maternal and neonatal outcomes following cesarean section in second versus first stage of labour in a tertiary hospital in Nigeria	OBGYN	CAES	N	2011	2008	R	audit	Nigeria	AH	U	N	IP	3	347	0.009
698	Raboi, A.	Mechanical Valve Dysfunction in Yemen	CARDI	VALVEOBS	N	2010	2005	R	audit	Yemen, Rep.	AH	U	Y	IP	23	129	0.178
699	Rachid, S.	Laparoscopic cholecystectomy in sickle cell patients in Niger	GENSX	CHOLE	Y	2009	2006	P	audit	Niger	DH	U	N	IP	0	47	0.000
700	Radak, D.	Renal tumor with tumor thrombus in inferior vena cava and right atrium: the report of five cases with long-term follow-up	UROLO	TTHR	N	2011	2006	R	audit	Serbia	AH	U	Y	IP	0	5	0.000
701	Radinovic, K.S.	Effect of the Overlap Syndrome of Depressive Symptoms and Delirium on Outcomes in Elderly Adults with Hip Fracture: A Prospective Cohort Study	ORTHO	HIPFRAC	N	2014	2010	P	nonrandomized cohort	Serbia	AH	U	Y	30D	27	277	0.097
702	Rahman, G.A.	Possible risk factors for respiratory complications after thyroidectomy: An observational study	ENT	THYROID	N	2009	1996	P	audit	Nigeria	AH	U	N	IP	0	262	0.000
703	Rahman, G.A.	Rectal cancer: Pattern and outcome of management in University of Ilorin teaching hospital, Ilorin, Nigeria	GENSX	RECTAL	N	2010	2000	P	audit	Nigeria	AH	U	Y	30D	2	34	0.059
704	Rahmanian, A	Benefits of early aneursym surgery: Southern Iran Experience	NEURO	ANEUR	N	2012	2009	Mixed R/P	nonrandomized cohort	Iran, Islamic Rep.	AH	U	N	IP	56	334	0.168
705	Ramku EMD	Considerations for Children's tracheostomy report of 30 patients seen in a 4 year period	PAEDS	TRACHEOST	N	2009	2004	R	audit	Kosovo	AH	U	N	IP	6	30	0.200
706	Ramli, R. R.	Outcome of neck dissections in a rural tertiary University Hospital in Malaysia	ENT	NECKDIS	N	2011	2006	R	audit	Malaysia	AH	R	N	IP	1	14	0.071
707	Ramos Junior, F	Surgical management of intracranial aneurysms in the “coiling age”	NEURO	ANEUR	N	2009	2004	R	audit	Brazil	AH	U	Y	IP	18	149	0.121
708	Raso, J.L.	Bridge-therapy with enoxaparin in the preoperative period of endarterectomy	VASCU	CEA	N	2010	2005	P	audit	Brazil	DH	Mixed	N	IP	1	119	0.008
709	Rathi, P. K.	Laparoscopic cholecystectomy without the use of drain in selected cases	GENSX	CHOLE	N	2011	2009	P	nonrandomized cohort	Pakistan	AH	U	N	IP	0	100	0.000
710	Rathod, J.B.	Upper gastrointestinal bleeding: audit of a single center experience in Western India	GENSX	UGIB	N	2011	2002	P	audit	India	AH	U	Y	30D	0	6	0.000
711	Raza, M. S.	Flexor zone 5 cut injuries: Emergency management and outcome	PLAST	FTI	N	2014	2010	P	nonrandomized cohort	Pakistan	AH	U	N	IP	0	31	0.000
712	Reddy, D.	Holes in the heart: an atlas of intracardiac injuries following penetrating trauma	CARDI	CTRAUM	N	2014	2008	R	audit	South Africa	DH	U	Y	IP	0	10	0.000
713	Redman, L.A.	HIV, vascular surgery and cardiovascular outcomes: a South African cohort study	VASCU	VASCU	Y	2014	2009	P	nonrandomized cohort	South Africa	DH	U	Y	30D	11	225	0.049
714	Redwan, A.A.	Complex Post-cholecystectomy Biliary Injuries: Management with 10 Years’ Experience in a Major Referral Center	HEPAT	BILD	N	2012	2005	Mixed R/P	nonrandomized cohort	Egypt, Arab Rep.	AH	U	N	IP	2	135	0.015
715	Rehman, L.	"Outcome of Cloward Technique in Cervical Disc Prolapse"	NEURO	SPINE	N	2010	2008	P	audit	Pakistan	AH	U	N	IP	0	30	0.000
716	Rehman, Z.U.	Intussusceptions in Adults: Clinical Features and Operative Procedures	GENSX	INTUSS	N	2010	2003	R	audit	Pakistan	AH	U	Y	30D	0	18	0.000
717	Rekik, S.	Short- and long-term outcomes of surgery for active infective endocarditis: a Tunisian experience	CARDI	ENDOCARD	N	2009	2001	R	audit	Tunisia	AH	U	Y	IP	24	88	0.273
718	Rekik, S.	Prosthetic valve endocarditis: management strategies and prognosis	CARDI	ENDOCARD	N	2009	2001	R	nonrandomized cohort	Tunisia	AH	U	Y	IP	7	20	0.350
719	Ren, L.	Mortality rate prediction by Physiological and Operative Severity Score for the Enumeration of Mortality and Morbidity (POSSUM), Portsmouth POSSUM and Colorectal POSSUM and the development of new scoring systems in Chinese colorectal cancer patients	GENSX	COLRES	N	2009	1998	R	audit	China	AH	U	Y	IP	9	903	0.010
720	Ren, M	Comparison of short-term effect of thoracoscopic segmentectomy and thoracoscopic lobectomy for the solitary pulmonary nodule and early-stage lung cancer	THORA	LUNGRES	N	2014	2011	R	nonrandomized cohort	China	AH	U	N	IP	0	82	0.000
721	Restrepo, C.A.	Implantation of peritoneal catheters by laparotomy: nephrologists obtained siilar results to general surgeons	GENSX	PTCATH	N	2014	2004	Mixed R/P	nonrandomized cohort	Colombia	AH	U	Y	30D	0	342	0.000
722	Rezende, K.F.	Direct costs and outcomes for inpatients with diabetes mellitus and foot ulcers in a developing country: The experience of the public health system of Brazil	VASCU	DIABINF	N	2009	2003	P	nonrandomized cohort	Brazil	Mixed	U	Y	IP	14	93	0.151
723	Ribeiro, G.	Surgery for Valvular Heart Disease: A Population-Based Study in a Brazilian Urban Center	CARDI	VALVE	N	2012	2003	R	audit	Brazil	Mixed	U	Y	IP	58	491	0.118
724	Ribeiro TA	Predictors of hip fracture mortality at a general hospital in South Brazil: an unacceptable surgical delay	ORTHO	HIPFRAC	N	2014	2008	P	audit	Brazil	AH	U	Y	IP	18	418	0.043
725	Rivetti, L	Surgical Treatment of Ventricular Tachyarrhythmia. Which one is the Ideal Patient?	CARDI	CARDAN	N	2011	2009	R	audit	Brazil	AH	U	N	IP	2	63	0.032
726	Robbs, J.V.	Management of HIV Vasculopathy - A South African Experience	VASCU	VASCU	Y	2010	2007	R	audit	South Africa	DH	U	Y	30D	14	141	0.099
727	Rodrigues, A.J.	Risk factors for acute kidney injury after cardiac surgery	CARDI	CARD	N	2009	2006	R	nonrandomized cohort	Brazil	AH	U	Y	IP	81	769	0.105
728	Rodrigues, A.J.	Isolated Mitral and Aortic Valve Replacement with the St. Jude Medical Valve: A Midterm Follow-up	CARDI	VALVE	N	2009	1999	R	nonrandomized cohort	Brazil	AH	U	N	IP	20	270	0.074
729	Rodrigues, L.C.L.	Effectiveness Of The Surgical Intervention In The Quality Of Life And Survival Of Patients With Metastatic Lesions In The Spine	NEURO	SPINE	N	2014	2008	P	audit	Brazil	AH	U	Y	30D	6	67	0.090
730	Roodpeyma, S.	A prospective observational study of paediatric cardiac surgery outcomes in a postoperative intensive care unit in Iran	CARDI	PCARD	N	2013	2010	P	audit	Iran, Islamic Rep.	AH	U	Y	IP	25	202	0.124
731	Rooh ul, M	Laparoscopic treatment of hepatic hydatid cyst	HEPAT	LIVHYDAT	N	2011	2008	P	audit	Pakistan	AH	U	N	IP	0	43	0.000

Reference	First Author	Article Title	Specialty	Procedure or Diagnosis	High-risk population?	Year Published	Data midpoint year	Retrospective or prospective	Study design	Country	Type of Facilities Included	Urban/ Rural/ Mix	Was POMR clearly defined?	Timeframe of POMR	POMR numerator	POMR denomiator	Overall POMR
732	Rouf, S.	Relaparotomy after cesarean section: Experience from a tertiary referral and teaching hospital of Bangladesh	OBGYN	RELAPCS	N	2009	2007	R	audit	Bangladesh	AH	U	N	IP	6	24	0.250
733	Rukewe, A.	Anaesthesia for caesarean deliveries and maternal complications in a Nigerian teaching hospital	OBGYN	CAES	N	2014	2009	R	audit	Nigeria	AH	U	Y	OT	10	3389	0.003
734	Sa, M.P.B.	GuaragnaSCORE satisfactorily predicts outcomes in heart valve surgery in a Brazilian hospital	CARDI	VALVE	N	2012	2009	R	audit	Brazil	AH	U	Y	IP	74	491	0.151
735	SA, M.P.B.O.	Risk factors for low cardiac output syndrome after coronary artery bypass grafting surgery	CARDI	CABG	N	2012	2009	P	case-control	Brazil	AH	U	N	IP	47	89	0.528
736	Saaiq, M.	Presentation and outcome of surgically managed liver trauma: experience at a tertiary care teaching hospital	TRAUM	LIVTRAUM	N	2013	2006	R	audit	Pakistan	AH	U	Y	IP	11	113	0.097
737	Saaiq, M.	Abdominal tuberculosis: epidemiologic profile and management experience of 233 cases	GENSX	ABDOTB	N	2012	2005	P	audit	Pakistan	AH	U	Y	IP	5	164	0.030
738	Sabzi, F.	Low dose aprotinin increases mortality and morbidity in coronary artery bypass surgery	CARDI	CABG	N	2012	2008	P	RCT	Iran, Islamic Rep.	AH	U	Y	IP	24	653	0.037
739	Sadaf, N.	Maternal and foetal outcome in HELLP syndrome at tertiary care hospital	OBGYN	CAES	Y	2013	2007	P	audit	Pakistan	AH	U	N	IP	0	24	0.000
740	Sadaka, M	Study of infective endocarditis in Alexandria main university hospitals	CARDI	ENDOCARD	N	2013	2010	P	nonrandomized cohort	Egypt, Arab Rep.	AH	U	Y	IP	3	16	0.188
741	Sadeck, L.S.R.	Effects of therapeutic approach on the neonatal evolution of very low birth weight infants with patent ductus arteriosus	CARDI	PCARD	N	2014	2010	R	nonrandomized cohort	Brazil	Mixed	Mixed	N	IP	15	102	0.147
742	Sadeghi, M. M., Arasteh,	Evaluation of accuracy of Euroscore risk model in prediction of perioperative mortality after Coronary Bypass Graft Surgery in Isfahan, Iran	CARDI	CABG	N	2011	2008	P	audit	Iran, Islamic Rep.	AH	U	Y	30D	49	1362	0.036
743	Sadrizadeh, A.	Evaluation of the effect of pulmonary hydatid cyst location on the surgical technique approaches	THORA	THORHYDAT	N	2014	2011	P	nonrandomized cohort	Iran, Islamic Rep.	AH	U	N	IP	1	87	0.011
744	Saeed, F.	Peripartum hysterectomy: a ten-year experience at a tertiary care hospital in a developing country	OBGYN	EPH	N	2010	2000	R	audit	Pakistan	AH	U	N	IP	2	39	0.051
745	Safari, S.	Outcomes of Fasciotomy in Patients With Crush-induced Acute Kidney Injury After Bam Earthquake	TRAUM	FASC	N	2011	2003	R	nonrandomized cohort	Iran, Islamic Rep.	Mixed	Mixed	N	IP	8	70	0.114
746	Sah, B.K.	Reoperation for early postoperative complications after gastric cancer surgery in a Chinese hospital	GENSX	GASTCA	N	2010	2007	R	nonrandomized cohort	China	AH	U	Y	IP	17	1639	0.010
747	Saha L	Study on primary cesarean section	OBGYN	CAES	N	2011	2004	P	audit	Bangladesh	AH	U	Y	IP	0	100	0.000
748	Saha, N.	Comparison of post operative morbidity between laparoscopic and open appendectomy in children	GENSX	APPY	N	2010	2008	P	nonrandomized cohort	Bangladesh	AH	U	Y	7D	0	60	0.000
749	Sahin, S.	Emergency peripartum hysterectomy: our 12-year experience	OBGYN	EPH	N	2014	2007	R	audit	Turkey	AH	U	Y	IP	2	44	0.045
750	Sai, Ke	Neurosurgical interventions for patients with nasopharyngeal carcinoma: a single institution experience	NEURO	NPCR	N	2013	2005	R	audit	China	AH	U	N	30D	0	18	0.000
751	Saidi, H	Motorcycle injuries at a tertiary referral hospital in Kenya: injury patterns and outcome	TRAUM	TRAUM	N	2013	2010	P	audit	Kenya	AH	U	N	IP	3	104	0.029
752	Salahuddin, O.	Acute appendicitis in the elderly; Pakistan Ordnance Factories hospital, Wah Cantt. experience	GENSX	APPY	N	2012	2007	P	nonrandomized cohort	Pakistan	AH	U	N	IP	1	36	0.028
753	Salama, I.A.	Iatrogenic Biliary Injuries: Multidisciplinary Management in a Major Tertiary Referral Center	HEPAT	BILD	N	2014	2007	R	audit	Egypt, Arab Rep.	AH	U	N	IP	1	198	0.005
754	Salehi, R.	Surgical embolectomy in treating acute massive pulmonary embolism	THORA	PEMB	N	2013	2007	R	audit	Iran, Islamic Rep.	AH	U	Y	IP	2	16	0.125
755	Salehi, S.H.	Electrical injury in construction workers: A special focus on injury with electrical power	TRAUM	BURN	Y	2014	2011	R	audit	Iran, Islamic Rep.	AH	U	N	IP	9	202	0.045
756	Saluja, S.S.	Management of Choledochal Cysts and Their Complications	HEPAT	CDC	N	2012	2006	P	audit	India	AH	U	N	IP	1	114	0.009
757	Samuel, J.C.	Re-examining treatment strategies for sigmoid volvulus: An analysis of treatment and outcomes in Lilongwe, Malawi	GENSX	VOLV	N	2010	2006	R	audit	Malawi	AH	U	N	IP	2	239	0.008
758	Samuel, J.C.	An Observational Study of the Etiology, clinical presentation and outcomes associated with peritonitis in Lilongwe, Malawi	GENSX	AABDO	N	2011	2008	R	audit	Malawi	AH	U	Y	IP	28	190	0.147
759	Sangkomkamhang U	Mode of delivery and outcomes in preterm births	OBGYN	CAES	N	2011	2008	P	audit	Multiple	Mixed	Mixed	N	IP	2	294	0.007
760	Sani, R.	The Impact of Launching Surgery at the District Level in Niger	MULTI	MULTI	N	2009	2007	R	audit	Niger	DH	R	N	IP	15	544	0.028
761	Santo, M.A.	COMMON BILE DUCT STONES. Analysis of the videolaparoscopic surgical treatment	HEPAT	BILD	N	2012	2008	P	audit	Brazil	AH	U	N	IP	0	70	0.000
762	Santos, A.A.	Impact on early and late mortality after blood transfusion in coronary artery bypass graft surgery	CARDI	CABG	N	2012	2009	R	nonrandomized cohort	Brazil	AH	U	Y	30D	129	3004	0.043
763	Santos, A.C.	Emergency surgery for complicated colorectal cancer in central Brazil	GENSX	COLRES	N	2014	2008	R	audit	Brazil	AH	U	Y	IP	36	107	0.336
764	Santos, H.N.	Predictors of stroke in patients undergoing cardiac surgery	CARDI	CARD	N	2013	2003	R	nonrandomized cohort	Brazil	AH	U	Y	IP	425	4626	0.092
765	Santos, T.S.	Impacted foreign bodies in orbital region: review of nine cases	TRAUM	ORBTRAUM	N	2010	2008	R	audit	Brazil	CH	U	N	OT	1	9	0.111
766	Hayashi, L.Y.	Incidence, indication and complications of postoperative reintubation after elective intracranial surgery	NEURO	NEURO	N	2013	2004	P	nonrandomized cohort	Brazil	AH	U	Y	IP	16	169	0.095
767	Saramma, P.P.	Hyponatremia after aneurysmal subarachnoid hemorrhage: Implications and outcomes	NEURO	ICH	N	2013	2010	R	audit	India	AH	U	Y	IP	2	53	0.038
768	Saramma, P.P.	Alcohol-based hand rub and surgical site infection after elective neurosurgery: An intervention	NEURO	NEURO	N	2011	2006	R	audit	India	AH	U	N	30D	39	1899	0.021
769	Sardenberg, RAdS	Pulmonary metastasectomy from soft tissue sarcomas	THORA	LUNGRES	N	2010	1998	R	audit	Brazil	AH	U	Y	30D	0	77	0.000
770	Sarioglu, T.	Challenges in the management of patients with functionally univentricular heart in Turkey	CARDI	CCHD	N	2012	1999	R	audit	Turkey	AH	U	N	IP	24	256	0.094
771	Satomi, E.	Identification And Treatment Of Osteoporosis Among Elderly Patients With Hip Fractures	ORTHO	HIPFRAC	N	2009	2005	R	audit	Brazil	AH	U	Y	IP	7	123	0.057
772	Saula, P.W.	Hypertrophic pyloric stenosis in the Third World	PAEDS	PYLORSTEN	N	2011	2006	R	audit	South Africa	AH	U	Y	30D	0	63	0.000
773	Savlovschi, C.	Post-surgery morbidity and mortality in colorectal cancer in elderly subjects.	GENSX	COLRES	N	2013	2005	R	audit	Romania	AH	U	N	IP	6	36	0.167

Reference	First Author	Article Title	Specialty	Procedure or Diagnosis	High-risk population?	Year Published	Data midpoint year	Retrospective or prospective	Study design	Country	Type of Facilities Included	Urban/ Rural/ Mix	Was POMR clearly defined?	Timeframe of POMR	POMR numerator	POMR denominator	Overall POMR
774	Sayed, S.	Effect of Different Preoperative Patient Characteristics on Coronary Surgery Outcome: A Comparative Study Between a Developing and a Developed Country	CARDI	CABG	N	2009	1999	P	audit	Egypt, Arab Rep.	AH	U	Y	30D	4	60	0.067
775	Sayyah-Melli, M	Comparison of Platinum-based Neoadjuvant Chemotherapy and Primary Debulking Surgery in Patients with Advanced Ovarian Cancer	OBGYN	OVACA	N	2013	2012	P	nonrandomized cohort	Iran, Islamic Rep.	AH	U	N	IP	0	60	0.000
776	Scott, C.	The provision of surgical care for children with cardiac disease: The Jamaican experience - an 18-year review	CARDI	PCARD	N	2012	2002	R	audit	Jamaica	AH	U	Y	30D	15	294	0.051
777	Seal, S.	Outcome in Second- versus First-Stage Cesarean Delivery in a Teaching Institution in Eastern India	OBGYN	CAES	N	2010	2005	R	nonrandomized cohort	India	AH	U	N	IP	2	1826	0.001
778	Secchi, M.A.	Surgical management of liver hydatidosis: a multi centre series of 1412 patients	HEPAT	LIVHYDAT	N	2009	1991	Mixed R/P	nonrandomized cohort	Argentina	Mixed	Mixed	Y	60D_IP	26	1412	0.018
779	Sehitogullari, A.	A long-term study assessing the factors influencing survival and morbidity in the surgical management of bronchiectasis	THORA	LUNGRES	N	2011	2006	R	audit	Turkey	AH	U	Y	30D	0	129	0.000
780	Sekabira J	Gastroschisis: a third world perspective.	PAEDS	GASTROSCH	N	2009	2004	R	audit	South Africa	AH	U	N	IP	42	102	0.412
781	Sekirime, W.K.	Outcome of cesarean section in asymptomatic HIV-1 infection in Kampala, Ugandajog_1002 679..688	OBGYN	CAES	N	2009	2008	P	nonrandomized cohort	Uganda	AH	U	N	IP	7	500	0.014
782	Sen Gupta TKS	Emergency managemnt of sigmoid volvulus - Institutional experience over four years	GENSX	VOLV	N	2011	2008	R	audit	India	AH	U	N	IP	12	82	0.146
783	Sen I	Clinical profile of aortoiliac occlusive disease and outcomes of aortobifemoral bypass in India	VASCU	BYPASS	N	2013	2007	R	audit	India	AH	U	Y	IP_30D	3	99	0.030
784	Sengun, I.S.	Results Of Diaphragm Pacing Application in Amyotrophic Lateral Sclerosis Patients. First Turkish Experience	THORA	DPSS	N	2013	2012	P	audit	Turkey	AH	U	N	IP	0	11	0.000
785	Serrano Júnior, C. V.	Efficacy of aneurysmectomy in patients with severe left ventricular dysfunction: favorable short-and long-term results in ischemic cardiomyopathy	CARDI	CARDAN	N	2010	1994	R	audit	Brazil	AH	U	Y	IP	11	169	0.065
786	Shahabuddin, S.	Redo coronary artery surgery; early and intermediate outcomes from a tertiary care hospital in a developing country	CARDI	CABG	Y	2011	2002	R	audit	Pakistan	AH	U	Y	30D	6	82	0.073
787	Shaikh, A. R.	Changing practice of rectal cancer surgery in Pakistan	GENSX	RECTAL	N	2010	2002	R	audit	Pakistan	AH	U	N	IP	0	200	0.000
788	Shaikh, A.R.	Laparoscopic surgery for rectal cancer - An early experience from Pakistan	GENSX	RECTAL	N	2014	2010	P	audit	Pakistan	AH	U	Y	30D	0	31	0.000
789	Shaikh, A. R.	Inguinal mesh hernioplasty under local anaesthesia	GENSX	INGHERN	N	2012	2008	P	audit	Pakistan	AH	U	Y	IP	0	108	0.000
790	Shaikh, A. R.	Clinical outcomes of laparoscopic versus open appendectomy	GENSX	APPY	N	2009	2004	P	nonrandomized cohort	Pakistan	AH	U	Y	30D	0	100	0.000
791	Shaikh, G.S.	Traumatic duodenal injuries and surgical management at tertiary care hospital Chandka Medical College Hospital Larkana	TRAUM	LAPAR	N	2011	2008	R	audit	Pakistan	AH	U	N	IP	9	32	0.281
792	Shaikh, N. B.	Morbidity and mortality associated with obstetric hysterectomy	OBGYN	EPH	N	2010	2005	R	audit	Pakistan	AH	U	N	IP	5	41	0.122
793	Shamim, M. S.	Non-operative management is superior to surgical stabilization in spine injury patients with complete neurological deficits: A perspective study from a developing world country, Pakistan	NEURO	STRAUM	N	2011	2006	R	case-control	Pakistan	AH	U	Y	30D	4	27	0.148
794	Sheikh, M.	Outcome of coronary artery bypass grafting in a tertiary-care center in Pakistan	CARDI	CABG	N	2014	2009	R	audit	Pakistan	AH	U	Y	30D	98	2851	0.034
795	Sheng, Q. F.	Re-operation for Hirschsprung's disease: experience in 24 patients from China	PAEDS	HIRSCH	Y	2012	2004	R	audit	China	AH	U	N	30D	0	24	0.000
796	Shetty, G.	Complications as indicators of quality assurance after 401 consecutive colorectal cancer resections: the importance of surgeon volume in developing colorectal cancer units in India	GENSX	COLRES	N	2012	2005	Mixed R/P	audit	India	AH	U	N	IP	5	401	0.012
797	Shetty, V.H.	Role of Ultrasonography in Diagnosis of Ectopic Pregnancy with Clinical Analysis and Management in Tertiary Care Hospital	OBGYN	ECTOP	N	2014	2007	P	audit	India	AH	U	N	IP	0	100	0.000
798	Shi J-H	A mortality risk assessment model for cardiac valve replacement surgery and its application in the use of prophylactic extracorporeal membrane oxygenation	CARDI	VALVE	N	2010	1999	R	audit	China	AH	U	Y	IP	266	4640	0.057
799	Shi, Y.	Comparing the effects of Bassini versus tension-free hernioplasty: 3 years' follow-up	GENSX	INGHERN	N	2010	2003	P	RCT	China	AH	U	N	IP	0	552	0.000
800	Shiekh, K.A.	Mechanical small bowel obstruction in children at a tertiary care centre in Kashmir	PAEDS	BOBS	N	2010	2006	P	audit	India	AH	U	N	IP	5	126	0.040
801	Shrestha, A.	Outcome of Head Injury Patients Undergoing Surgical Management: A Tertiary Level Experience	NEURO	ACHI	N	2011	2010	P	nonrandomized cohort	Nepal	AH	U	N	IP	47	312	0.151
802	Shrikhande SV	Evolution of pancreatoduodenectomy in a tertiary cancer center in India: Improved results from service reconfiguration	HEPAT	WHIP	N	2013	2001	Mixed R/P	nonrandomized cohort	India	AH	U	Y	30D	27	500	0.054
803	Shu, Q	Experience in minimally invasive Nuss operation for 406 children with pectus excavatum	PAEDS	PECTUS	N	2011	2007	R	audit	China	AH	U	N	30D	0	406	0.000
804	Shu'aibu, S.I.	Preliminary Experience With Radical Cystectomy And W–Ileal Pouch For Muscle Invasive Transitional Cell Bladder Carcinoma	UROLO	RADCYS	N	2012	2009	R	audit	Nigeria	AH	U	N	IP	1	6	0.167
805	Shukla, P.	Peri-operative outcomes for pancreatoduodenectomy in India: a multi-centric study	HEPAT	WHIP	N	2009	2006	R	audit	India	AH	U	Y	30D	26	718	0.036
806	Shyam, D. C.	Inguinal hernias in patients of 50 years and above. Pattern and outcome	GENSX	INGHERN	N	2013	2012	P	audit	India	AH	U	N	30D	0	57	0.000
807	Sibanda, M.	A prospective evaluation of lower extremity ulcers in a Zimbabwean population	PLAST	LLU	N	2009	2008	P	audit	Zimbabwe	AH	U	Y	IP	10	100	0.100
808	Siddappa, S.	Xanthogranulomatous Pyelonephritis: A Retrospective Review of 16 Cases	UROLO	NEPHREC	N	2011	2008	R	audit	India	AH	U	N	IP	0	16	0.000
809	Siddiqui, M.A.	Risk factors of prolonged mechanical ventilation following open heart surgery: what has changed over the last decade?	CARDI	CARD	N	2012	2010	R	audit	Pakistan	AH	U	N	IP	31	1617	0.019

Reference	First Author	Article Title	Specialty	Procedure or Diagnosis	High-risk population?	Year Published	Data midpoint year	Retrospective or prospective	Study design	Country	Type of Facilities Included	Urban/ Rural/ Mix	Was POMR clearly defined?	Timeframe of POMR	POMR numerator	POMR denominator	Overall POMR
810	Siddiqui, M. T.	Contegra valved conduit in the paediatric population: An exciting prospect for right ventricle to pulmonary artery reconstruction; experience and outcomes at Aga Khan University	CARDI	CCHD	N	2012	2006	R	audit	Pakistan	AH	U	Y	IP	3	16	0.188
811	Sie Essoh, J.	Limb amputations in adults in an ivorian teaching hospital	ORTHO	AMPUT	N	2009	1997	R	audit	Cote d'Ivoire	AH	U	Y	30D	25	156	0.160
812	Silva, A.R.	Surgical Mitral Valve Repair in Children with Rheumatic Fever	CARDI	VALVE	N	2008	2000	R	audit	Brazil	AH	U	Y	IP	3	40	0.075
813	Silva, K.P.	Newborns with congenital heart diseases: epidemiological data from a single reference center in Brazil epidemiological data from a single reference center in Brazil	CARDI	PCARD	N	2014	2010	R	nonrandomized cohort	Brazil	AH	U	N	IP	5	27	0.185
814	Silva, L.	Horizontal right axillary minithoracotomy: aesthetic and effective option for atrial and ventricular septal defect repair in infants and toddlers	CARDI	PCARD	N	2013	2012	R	audit	Brazil	AH	U	N	IP	0	50	0.000
815	Silveira Filho LM	A bovine pericardium rigid prosthesis for left ventricle restoration: 12 years of follow-up	CARDI	CARDAN	N	2011	2003	R	case-control	Brazil	AH	U	Y	30D	10	72	0.139
816	Sincos, I.R.	Endovascular and open repair for blunt aortic injury, treated in one clinical institution in Brazil. A case series	TRAUM	CTRAUM	N	2011	2005	R	case-control	Brazil	AH	U	Y	30D	1	5	0.200
817	Singh, J.B.	Management of otogenic lateral sinus thrombosis	ENT	MASTOID	N	2014	2008	R	audit	India	AH	U	Y	30D	0	6	0.000
818	Singh, H.	A simple prognostic scoring system for typhoid ileal perforation peritonitis	GENSX	TIP	N	2010	2006	P	audit	India	AH	U	Y	IP	17	82	0.207
819	Singh, R.	Preoperative predictors of mortality in adult patients with perforation peritonitis	GENSX	PERF	N	2011	2008	P	audit	India	AH	U	Y	IP	15	84	0.179
820	Singh, S. P.	Laparoscopic Cholecystectomy v/s Open Cholecystectomy: A comparative study at LLRM Medical College & Hospital, Meerut	GENSX	CHOLE	N	2012	2008	P	audit	India	AH	U	N	IP	0	46	0.000
821	Singh, G.	Primary Supratentorial Haemorrhage – Surgery or no Surgery in an Indian Setup	NEURO	ICH	N	2014	2012	P	audit	India	AH	U	Y	30D	10	28	0.357
822	Singh, V.	Repair of vesicovaginal fistula by the transabdominal route: outcome at a north Indian tertiary hospital	OBGYN	VVF	N	2011	2005	R	audit	India	AH	U	N	IP	0	48	0.000
823	Siqueira Júnior, T. M.	Transperitoneal versus extraperitoneal laparoscopic radical prostatectomy during the learning curve: does the surgical approach affect the complication rate?	UROLO	PROST	N	2010	2005	R	nonrandomized cohort	Brazil	AH	U	N	30D	1	80	0.013
824	Smaniotto, B.	Hepatic trauma: analysis of the treatment with intrahepatic balloon in a university hospital of curitiba	TRAUM	LIVTRAUM	N	2009	2003	R	audit	Brazil	AH	U	N	IP	11	18	0.611
825	Soares, R.R.	Intraoperative Volume replacement: crystalloids versus colloids in Surgical Myocardial Revascularization Without Cardiopulmonary Bypass (CPB)	CARDI	CABG	N	2009	2007	P	RCT	Brazil	AH	U	N	IP	1	40	0.025
826	Sodhi, HBS	The clinical profile, management, and overall outcome of aneurysmal subarachnoid hemorrhage at the neurosurgical unit of a tertiary care center in India	NEURO	ICH	N	2014	2013	P	audit	India	AH	U	Y	90D	85	307	0.277
827	Sohrab, N.	Prevalence And Risk Factors Of Mortality After Surgery For Congenital Heart Disease In Tabriz, Iran	CARDI	PCARD	N	2010	2007	R	audit	Iran, Islamic Rep.	AH	U	N	IP	120	1516	0.079
828	Sohrabi, B.	The impact of diabetes on early and midterm outcome of patients undergoing coronary artery bypass grafting surgery	CARDI	CABG	N	2010	2000	R	audit	Iran, Islamic Rep.	AH	U	Y	30D	69	1269	0.054
829	Sokouti, M.	A comprehensive study of mediastinal goiters	ENT	GOITRE	N	2010	2005	R	audit	Iran, Islamic Rep.	AH	U	N	IP	0	59	0.000
830	Solmaz, I.	Traumatic Brain Injury due to gunshot wounds: a single institution's experience with 442 consecutive patients	NEURO	CGSW	N	2009	1995	R	audit	Turkey	AH	U	N	IP	47	442	0.106
831	Sonshine, D.B.	The Implementation of a Pilot Femur Fracture Registry at Komfo Anokye Teaching Hospital: An Analysis of Data Quality and Barriers to Collaborative Capacity Building	ORTHO	FEMFRAC	N	2012	2011	P	nonrandomized cohort	Ghana	AH	R	N	IP	0	96	0.000
832	Sorbye, I.K.	Caesarean section among referred and self-referred birthing women: a cohort study from a tertiary hospital, northeastern Tanzania.	OBGYN	CAES	N	2011	2003	P	audit	Tanzania	DH	U	Y	IP	7	6161	0.001
833	Sorour, M.A.	Conservative approach in the management of isolated penetrating liver trauma.	TRAUM	LIVTRAUM	N	2013	2008	P	audit	Egypt, Arab Rep.	AH	U	N	IP	2	5	0.400
834	Souza, JP.	Caesarean section without medical indications is associated with an increased risk of adverse short- term maternal outcomes: the 2004-2008 WHO Global Survey on Maternal and Perinatal Health	OBGYN	CAES	N	2010	2006	R	audit	Multiple	Mixed	Mixed	Y	7D	112	73718	0.002
835	Srinath, S.	Risk prediction of morbidity and mortality in emergency laparotomy by possum equation	GENSX	LAPAR	N	2013	2011	P	nonrandomized cohort	India	AH	U	Y	30D	9	72	0.125
836	Sriussadaporn, S.	Lessons learned from 100 personal consecutive cases of Pancreaticoduodenectomy at a University Hospital in Thailand	HEPAT	WHIP	N	2013	2002	R	audit	Thailand	AH	U	Y	30D	2	100	0.020
837	Stanzani, F	Morbidity, mortality, and categorization of the risk of perioperative complications in lung cancer patients	THORA	LUNGRES	N	2014	2005	R	audit	Brazil	AH	U	Y	30D	8	145	0.055
838	Suciu B	Factors affecting early morbidity and mortality in non-small cell lung cancer surgery, the experience of surgical clinic no. 1TG. Mures	THORA	LUNGRES	N	2011	2007	R	audit	Romania	AH	U	Y	IP_30D	8	197	0.041
839	Suksompong, S.	Thoracic surgery mortality and morbidity in a university hospital	THORA	LUNGRES	N	2012	2008	R	audit	Thailand	AH	U	Y	30D	5	558	0.009
840	Sule, A.	Adult large bowel obstruction: A review of clinical experience	GENSX	COLRES	N	2011	1996	P	audit	Nigeria	AH	U	Y	IP	3	48	0.063
841	Sumer, A.	Outcome of surgical treatment of intestinal perforation in typhoid fever	GENSX	TIP	N	2010	2002	R	audit	Turkey	AH	U	N	IP	1	22	0.045
842	Sun, H.	An effective treatment for cerebral hemorrhage: minimally invasive craniopuncture combined with urokinase infusion therapy	NEURO	ICH	N	2010	2004	P	RCT	China	Mixed	Mixed	Y	14D	42	304	0.138
843	Surapaneni, S.	The Perforation-Operation time Interval; an Important Mortality Indicator in Peptic Ulcer Perforation	GENSX	PERF	N	2013	2009	R	audit	India	AH	U	N	IP	15	150	0.100

Reference	First Author	Article Title	Specialty	Procedure or Diagnosis	High-risk population?	Year Published	Data midpoint year	Retrospective or prospective	Study design	Country	Type of Facilities Included	Urban/ Rural/ Mix	Was POMR clearly defined?	Timeframe of POMR	POMR numerator	POMR denomiator	Overall POMR
844	Suwannarurk, K.	Silent abnormal placentation linkage to peripartum hysterectomy: Thammasat University Hospital 6-year study	OBGYN	EPH	N	2014	2009	R	audit	Thailand	AH	U	N	IP	3	37	0.081
845	Swende, TZ	Minilaparotomy Female Sterilisation At A Nigerian Tertiary Health Centre	OBGYN	BTL	N	2015	2005	R	audit	Nigeria	Mixed	Mixed	N	IP	0	78	0.000
846	Talpur, A.A.	Evaluation & management of patients with liver trauma	TRAUM	LIVTRAUM	N	2013	2010	P	audit	Pakistan	AH	U	N	IP	7	103	0.068
847	Talpur, N	Maternal and fetal morbidity with abruptio placentae	OBGYN	CAES	Y	2011	2008	R	audit	Pakistan	AH	U	N	IP	0	10	0.000
848	Talukder, M.M.H.	Surgery for primary intracerebral haemorrhage: is it safe and effective?	NEURO	ICH	N	2012	2006	P	nonrandomized cohort	Bangladesh	AH	U	Y	IP	13	30	0.433
849	Talwar, S.	Anomalous branch of pulmonary artery from the aorta and tetralogy of Fallot: morphology, surgical techniques and results	CARDI	CCHD	N	2014	2003	R	audit	India	AH	U	N	30D	2	11	0.182
850	Tamdee, D.	Factors related to 24-hour perioperative cardiac arrest in geriatric patients in a thai university hospital	MULTI	MULTI	N	2009	2005	P	audit	Thailand	AH	U	Y	24H	28	8905	0.003
851	Tamiru, T.	An alternative method of management of pediatric airway foreign bodies in the absence of rigid bronchoscopy	PAEDS	FORBOD	N	2012	2010	R	audit	Ethiopia	Other	R	N	IP	0	7	0.000
852	Tan, J.	Short-Term Outcomes from a Multicenter Retrospective Study in China Comparing Laparoscopic and Open Surgery for the Treatment of Infected Pancreatic Necrosis	HEPAT	PANCRE	N	2012	2009	R	nonrandomized cohort	China	AH	U	N	IP	4	76	0.053
853	Tan, W.M.	Treatment Outcome of Superficial Cerebral Abscess: An Analysis of Two Surgical Methods	NEURO	BABS	N	2010	2005	R	audit	Malaysia	Mixed	U	Y	IP	3	51	0.059
854	Tannuri, A.C.A.	Evolution of critically ill patients with gastroschisis from three tertiary centers	PAEDS	GASTROSCH	N	2011	2006	R	audit	Brazil	AH	U	Y	IP	23	163	0.141
855	Tannuri, A. C. A.	Does administering albumin to postoperative gastroschisis patients improve outcome?	PAEDS	GASTROSCH	N	2012	2005	P	nonrandomized cohort	Brazil	AH	U	N	IP	14	136	0.103
856	Tapisiz, O. L.	Emergency peripartum hysterectomy in a tertiary hospital in Ankara, Turkey: a 5-year review	OBGYN	EPH	N	2012	2008	R	audit	Turkey	AH	U	N	IP	2	30	0.067
857	Tarca, E.	Gastroschisis Treatment: What are the Causes of High Morbidity and Mortality Rates?	PAEDS	GASTROSCH	N	2013	2005	R	audit	Romania	AH	U	N	IP	34	54	0.630
858	Tariq, M	Clinical Profile and Outcome of Infective Endocarditis at the Aga Khan University Hospital	CARDI	ENDOCARD	N	2009	1994	R	audit	Pakistan	AH	U	Y	IP	3	18	0.167
859	Taslak Sengul, A.	Early diagnosis saves lives in esophageal perforations	THORA	ESPERF	N	2013	2005	R	case-control	Turkey	AH	U	N	IP	1	15	0.067
860	Tasoglu, I.	Primary Cardiac Myxomas: Clinical Experience and Surgical Results in 67 Patients	CARDI	CARDIACMYX	N	2009	1998	R	audit	Turkey	AH	U	Y	IP	3	67	0.045
861	Tavcar, I.	A 60-year experience in the treatment of pancreatic insulinoma in the Military Medical Academy, Belgrade, Serbia	HEPAT	PANC_RES	N	2014	1981	R	audit	Serbia	AH	U	N	IP	1	38	0.026
862	Tayyab, M.	Fournier's gangrene: A review of 15 cases	UROLO	FOURN	N	2010	2005	R	nonrandomized cohort	Pakistan	AH	U	N	IP	3	15	0.200
863	Tebeu, P. M.	Emergency obstetric hysterectomy at University Hospital, Yaounde, Cameroon	OBGYN	EPH	N	2013	2003	R	audit	Cameroon	AH	U	N	IP	1	20	0.050
864	Tedde, M. L.	The search for stability: bar displacement in three series of pectus excavatum patients treated with the Nuss technique	PAEDS	PECTUS	N	2011	2006	R	audit	Brazil	AH	U	N	30D	0	122	0.000
865	Teerapong, S.	Major complications of gynecological laparoscopy in Police General Hospital: a 4-year experience	OBGYN	MIS	N	2012	2008	R	audit	Thailand	Other	U	N	30D	0	423	0.000
866	Teicher, C.L.	Medecins Sans Frontieres Experience in Orthopedic Surgery in Postearthquake Haiti in 2010	ORTHO	OTRAUM	N	2014	2010	R	audit	Haiti	MSF	U	Y	IP	6	248	0.024
867	Teixeira, M.J.	Stereotactic biopsies of brain lesions	NEURO	ICBX	N	2009	1996	R	audit	Brazil	AH	U	N	IP	1	176	0.006
868	Tekumit, H.	Cusp Shaving for Concomitant Mild to Moderate Rheumatic Aortic Insufficiency	CARDI	CARD	Y	2010	2006	R	audit	Turkey	DH	U	Y	IP	2	43	0.047
869	Tennant, I. A.	Anaesthetic morbidity at the University Hospital of the West Indies	MULTI	MULTI	N	2009	2004	P	nonrandomized cohort	Jamaica	AH	U	N	IP	14	3185	0.004
870	Tenório, E. M.	Experience with the posterior leaflet extension technique for correction of rheumatic mitral insufficiency in children	CARDI	PCARD	N	2009	2004	R	audit	Brazil	AH	U	N	IP	1	30	0.033
871	Terra, R.	Surgical treatment of congenital tracheal stenoses*	THORA	TRACHSTEN	N	2009	2004	R	audit	Brazil	AH	U	N	IP	2	7	0.286
872	Terra, R.M.	Does videothoracoscopy improve clinical outcomes when implemented as part of a pleural empyema treatment algorithm?	THORA	DECORT	N	2012	2005	R	case-control	Brazil	AH	U	Y	60D	31	206	0.150
873	Terzi, A.	A case series of 46 appendectomies during pregnancy	GENSX	APPY	Y	2010	2007	R	audit	Turkey	AH	U	N	IP	0	46	0.000
874	Thakur, B.	Minimally invasive esophagectomy/gastroesophagectomy for cancer. is it safe in nepalese context?	THORA	ESOCA	N	2012	2011	P	nonrandomized cohort	Nepal	AH	U	Y	IP	2	34	0.059
875	Thakur, B	Results of management of esophageal and GE junction malignancies in Nepalese context	THORA	ESOCA	N	2013	2007	R	audit	Nepal	AH	U	N	IP	19	327	0.058
876	Thapa, A.	Surgical interventions in intracranial arteriovenous malformations: Indications and outcome analysis in a changing scenario	NEURO	ANEUR	N	2009	2004	R	audit	India	AH	U	Y	IP	6	111	0.054
877	Thomas, G.	A Pilot Program of Mass Surgery Weeks for Treatment of Hydrocele Due to Lymphatic Filariasis in Central Nigeria	GENSX	INGHERN	N	2009	2003	Mixed R/P	audit	Nigeria	DH	R	N	IP	1	373	0.003
878	Tian, Y.	Laparoscopic Subtotal Cholecystectomy as an Alternative Procedure Designed to Prevent Bile Duct Injury: Experience of a Hospital in Northern China	GENSX	CHOLE	Y	2009	2006	R	audit	China	AH	U	N	IP	0	48	0.000
879	Tian, Z. Q.	A 10-year experience with hepatic trauma in a Chinese level one trauma center	TRAUM	LIVTRAUM	N	2014	2008	R	nonrandomized cohort	China	AH	U	N	IP	19	119	0.160
880	Tinoco, R.C	Laparoscopic Gastrectomy for Gastric Cancer	GENSX	GASTCA	N	2009	2000	R	audit	Brazil	AH	U	N	IP	5	92	0.054
881	Tomiyoshi, S. D. T.	Effectiveness of the ligation of intersphincteric fistula tract (LIFT) in the treatment of anal fistula: initial results	GENSX	FIA	N	2014	2012	P	audit	Brazil	AH	U	N	30D	0	8	0.000
882	Topaloglu, U.	Extended lymphadenectomy for gastric cancer: a single center experience in Istanbul	GENSX	GASTCA	N	2009	2002	R	nonrandomized cohort	Turkey	AH	U	Y	IP	0	56	0.000

Reference	First Author	Article Title	Specialty	Procedure or Diagnosis	High-risk population?	Year Published	Data midpoint year	Retrospective or prospective	Study design	Country	Type of Facilities Included	Urban/ Rural/ Mix	Was POMR clearly defined?	Timeframe of POMR	POMR numerator	POMR denominator	Overall POMR
883	Torina, A. G.	The effects of modified ultrafiltration on pulmonary function and transfusion requirements in patients underwent coronary artery bypass graft surgery	CARDI	CABG	N	2010	2008	P	RCT	Brazil	AH	U	Y	IP	0	37	0.000
884	Traoré, D.	Acute sigmoid volvulus: Results of surgical treatment in the teaching hospitals of Bamako	GENSX	VOLV	N	2014	2003	R	case-control	Mali	AH	U	Y	IP	17	417	0.041
885	Traore, A.	Adult intussusception: Diagnostic pitfalls, morbidity and mortality in a developing country	GENSX	INTUSS	N	2012	1993	R	audit	Mali	AH	U	N	IP	3	41	0.073
886	Travancas, P.R.	Comparison of mechanical and biological prostheses when used to replace heart valves in children and adolescents with rheumatic fever	CARDI	VALVE	N	2009	2000	R	audit	Brazil	DH	U	Y	IP	4	73	0.055
887	Trivedi, N.P.	Optimizing multimodality treatment for head and neck cancer in rural India	ENT	HNC	N	2012	2007	R	audit	India	CH	R	N	IP	2	184	0.011
888	Tudorache, S	Prenatal diagnosis and perinatal outcome in congenital diaphragmatic hernia. Single tertiary center report	PAEDS	CDH	N	2014	2011	R	audit	Romania	AH	U	Y	30D	6	14	0.429
889	Tufegdzic, M	The weekend effect in patients hospitalized for upper gastrointestinal bleeding: a single-center 10-year experience	GENSX	UGIB	N	2014	2007	R	audit	Serbia	AH	U	Y	IP	12	64	0.188
890	Tumusiime, G.	Surgical Mortality at a Mission Hospital in Western Uganda	MULTI	MULTI	N	2010	2008	R	audit	Uganda	Other	R	N	IP	23	1818	0.013
891	Tunçalp, O.	Assessment of maternal near-miss and quality of care in a hospital-based study in Accra, Ghana	OBGYN	OBGYNE	N	2013	2010	P	audit	Ghana	AH	U	Y	6W	10	1212	0.008
892	Tyson, A.F.	Survival after burn in a sub-Saharan burn unit: Challenges and opportunities	TRAUM	BURN	N	2013	2012	R	audit	Malawi	AH	U	Y	IP	23	163	0.141
893	Tyson, A.F.	Delivery of operative pediatric surgical care by physicians and non-physician clinicians in Malawi	PAEDS	PAED	N	2014	2012	R	case-control	Malawi	DH	U	Y	IP	12	506	0.024
894	Uche, E. O.	Determinants and outcomes of ventriculoperitoneal shunt infections in Enugu, Nigeria	NEURO	HYDRO	N	2014	2010	R	audit	Nigeria	AH	U	N	30D	8	198	0.040
895	Uday, SK	SILACIG: A novel technique of single-incision laparoscopic appendicectomy based on institutional experience of 29 cases	GENSX	APPY	N	2013	2011	P	audit	India	AH	U	N	30D	0	29	0.000
896	Ugochukwu AI	Acute perforated peptic ulcer: On clinical experience in an urban tertiary hospital in south east Nigeria	GENSX	PERF	N	2013	2008	R	audit	Nigeria	AH	U	N	IP	16	76	0.211
897	Ugochukwu, A.I.	Ileal perforation due to typhoid fever - review of operative management and outcome in an urban centre in Nigeria.	GENSX	TIP	N	2013	2008	R	audit	Nigeria	AH	U	N	IP	16	86	0.186
898	Ugwu, EOV.	A Five-year Survey of Caesarean Delivery at a Nigerian Tertiary Hospital	OBGYN	CAES	N	2011	2007	R	audit	Nigeria	AH	U	N	IP	7	980	0.007
899	Ugwu, R. O.	Pattern, outcome and challenges of neonatal surgical cases in a tertiary teaching hospital	PAEDS	PAED	N	2013	2006	R	audit	Nigeria	AH	U	N	IP	80	166	0.482
900	Ugwumba, F.	Fournier's gangrene – analysis of management and outcome in south-eastern Nigeria	UROLO	FOURN	N	2012	2001	R	audit	Nigeria	AH	U	N	IP	1	28	0.036
901	Ugwumba, F. O.	Transvesical prostatectomy in the management of benign prostatic hyperplasia in a developing country	UROLO	PROST	N	2014	2003	R	audit	Nigeria	AH	U	N	30D	3	297	0.010
902	Uz Zaman, A.	Penetrating abdominal injury: A tertiary care hospital experience	TRAUM	LAPAR	N	2014	2012	P	nonrandomized cohort	Pakistan	AH	U	N	IP	8	79	0.101
903	Uzair, M.	Frequency Of Urethrocutaneous Fistula Following Snodgrass Hypospadias Repair In Children	UROLO	HYPOS	N	2013	2009	P	audit	Pakistan	AH	U	Y	30D	0	52	0.000
904	Valle, F.H.	Morbidity and Mortality in Patients Aged over 75 Years Undergoing Surgery for Aortic Valve Replacement	CARDI	VALVE	N	2010	2004	R	audit	Brazil	AH	U	Y	IP	32	230	0.139
905	Varcus, F.	Laparoscopic treatment of perforated duodenal ulcer - a multicentric study	GENSX	PERF	N	2013	2000	R	audit	Romania	DH	Mixed	Y	30D	0	186	0.000
906	Vaziri, M.	Mediastinal masses: Review of 105 cases	THORA	MEDMASS	N	2009	2001	R	nonrandomized cohort	Iran, Islamic Rep.	AH	U	N	IP	17	105	0.162
907	Veena, P.	A review of 93 cases of ruptured uterus over a period of 2 years in a tertiary care hospital in South India	OBGYN	UTRUP	N	2012	2009	R	audit	India	AH	U	N	IP	0	93	0.000
908	Velicki L	Cardiac myxoma: clinical presentation, surgical treatment and outcome	CARDI	CARDIACMYX	N	2010	2007	R	audit	Serbia	DH	U	Y	30D	0	17	0.000
909	Venter, J.A.	An audit of paediatric intussusception radiological reduction at the Bloemfontein Academic Hospital Complex, Free State, South Africa	PAEDS	INTUSS	N	2013	2007	R	audit	South Africa	AH	U	N	IP	0	33	0.000
910	Verma, S	Evaluation of the Utility of the Fournier's Gangrene Severity Index in the Management of Fournier's Gangrene in North India: A Multicentre Retrospective Study	UROLO	FOURN	N	2012	2010	R	audit	India	AH	U	N	IP	26	95	0.274
911	Vidotto, M.C.	Implications of extubation failure and prolonged mechanical ventilation in the postoperative period following elective intracranial surgery	NEURO	NEURO	N	2011	2007	P	audit	Brazil	AH	U	Y	IP	18	317	0.057
912	Vijarnsorn, C.	Surveillance of Pediatric Cardiac Surgical Outcome Using Risk Stratifications at a Tertiary Care Center in Thailand	CARDI	PCARD	N	2011	2005	R	audit	Thailand	AH	U	Y	IP	14	230	0.061
913	Vukovic, G.	Diagnostics and treatment of liver injuries in polytrauma	TRAUM	LIVTRAUM	N	2012	2008	R	audit	Serbia	AH	U	Y	IP	23	224	0.103
914	Vukovic, M.	Total colectomy in older patients with acute malignant obstruction of the left-sided colons	GENSX	COLRES	N	2013	2010	R	audit	Serbia	AH	U	Y	30D	5	81	0.062
915	Waldron, NR	Acid violence in Cambodia: The human, medical, and surgical implications	TRAUM	BURN	N	2014	2006	R	audit	Cambodia	AH	U	N	IP	2	130	0.015
916	Walker, I.	Paediatric surgery and anaesthesia in south-western Uganda: a cross-sectional survey	PAEDS	PAED	N	2010	2008	R	audit	Uganda	Mixed	Mixed	Y	OT	4	5188	0.001
917	Wang, C.	Comparison of six risk scores for in-hospital mortality in chinese patients undergoing heart valve surgery	CARDI	VALVE	N	2013	2008	R	audit	China	AH	U	Y	IP	112	3479	0.032
918	Wang C	Validation of the European System for Cardiac Operative Risk Evaluation (EuroSCORE) in Chinese Heart Valve Surgery Patients	CARDI	VALVE	N	2010	2005	R	audit	China	AH	U	Y	IP	77	1726	0.045
919	Wang, E.	Treatment of osteoporotic vertebral compression fractures with percutaneous kyphoplasty: a report of 196 cases	NEURO	SPINE	N	2013	2007	P	audit	China	AH	U	N	IP	0	196	0.000

Reference	First Author	Article Title	Specialty	Procedure or Diagnosis	High-risk population?	Year Published	Data midpoint year	Retrospective or prospective	Study design	Country	Type of Facilities Included	Urban/ Rural/ Mix	Was POMR clearly defined?	Timeframe of POMR	POMR numerator	POMR denominator	Overall POMR
920	Wang, H	Liver Resection in Hepatitis B-Related Hepatocellular Carcinoma: Clinical Outcomes and Safety in Overweight and Obese Patients	HEPAT	LIVRES	N	2014	2011	R	audit	China	AH	U	Y	30D	23	1543	0.015
921	Wang, L.	Reduction of alkaline reflux gastritis and marginal ulcer by modified Braun enteroenterostomy in gastroenterologic reconstruction after pancreaticoduodenectomy	HEPAT	WHIP	N	2014	2009	P	RCT	China	AH	U	Y	IP	0	62	0.000
922	Wang, Q. Y.	Video-assisted mediastinoscopic resection compared with video-assisted thoracoscopic surgery in patients with esophageal cancer	THORA	ESOCA	N	2014	2008	R	audit	China	AH	U	N	30D	1	167	0.006
923	Wang, W	Clinical features of acute aortic dissection from the Registry of Aortic Dissection in China	CARDI	TAD	N	2014	2012	R	audit	China	AH	U	Y	IP	14	251	0.056
924	Wang, X.	A comparison before and after aprotinin was suspended in cardiac surgery: different results in the real world from a single cardiac center in China.	CARDI	CARD	N	2009	2007	R	case-control	China	AH	U	Y	IP	32	3924	0.008
925	Wang, Y	Comprehensive treatment for gas gangrene of the limbs in earthquakes	ORTHO	NECFASC	N	2013	2008	R	audit	China	AH	U	N	IP	1	19	0.053
926	Wani, I.	Intestinal Ascariasis in Children	PAEDS	ASCAR	N	2010	2007	P	audit	India	AH	U	N	IP	1	79	0.013
927	Warf, B.	Five-year survival and outcome of treatment for postinfectious hydrocephalus in Ugandan infants	NEURO	HYDRO	N	2011	2003	R	nonrandomized cohort	Uganda	AH	U	Y	30D	4	149	0.027
928	Wasay, M.	Preoperative antifungal therapymay improve survival in patients with Aspergillus brain abscess	NEURO	BABS	N	2009	2001	R	audit	Pakistan	AH	U	N	IP	10	25	0.400
929	Kayal, A (Wei, M)	A Comprehensive Prospective Clinical Study of Hydatid Disease	GENSX	HYDAT	N	2014	2010	P	audit	India	AH	U	N	IP	0	14	0.000
930	Wei, Q.	Peripartum hysterectomy in 38 hospitals in China: a population-based study	OBGYN	EPH	N	2014	2011	P	audit	China	Mixed	Mixed	N	IP	0	43	0.000
931	Weiser, T.G.	Effect of A 19-Item Surgical Safety Checklist During Urgent Operations in A Global Patient Population	MULTI	MULTI	N	2010	2009	P	nonrandomized cohort	Multiple	Mixed	Mixed	Y	IP_30D	44	1750	0.025
932	Wella, H.L.	Causes and clinical outcomes in neonates with acute abdomen requiring surgery at Muhimbili National Hospital Dar es Salaam	PAEDS	AABDO	N	2013	2009	P	audit	Tanzania	AH	U	N	IP	15	43	0.349
933	Williams, O.M.	Intestinal Atresia: A Four-Year Review of Cases in Ikeja-Lagos	PAEDS	IATRES	N	2012	2006	R	audit	Nigeria	AH	U	N	IP	9	22	0.409
934	Winkler, A.S.	The Pattern of Neurosurgical Disorders in Rural Northern Tanzania: A Prospective Hospital-Based Study	NEURO	NEURO	N	2010	2003	P	nonrandomized cohort	Tanzania	Other	R	N	IP	4	24	0.167
935	Wong, E. G.	Operative Procedures in the Elderly in Low-Resource Settings: A Review of Medecins Sans Frontieres Facilities	MULTI	MULTI	N	2014	2010	R	audit	Multiple	MSF	Mixed	Y	OT	152	60800	0.003
936	Wu, A	Long-Term Outcome of A Large Series of Gastric Cancer Patients in China	GENSX	GASTCA	N	2010	2000	Mixed R/P	audit	China	Other	U	Y	30D_IP	79	1882	0.042
937	Wu, X.	Clinical characteristics and long term post-operative outcome of cardiac Myxoma	CARDI	CARDIACMYX	N	2012	2004	R	audit	China	AH	U	Y	IP	0	112	0.000
938	Wu, Z.	Foramen magnum meningiomas: experiences in 114 patients at a single institute over 15 years	NEURO	RIM	N	2009	2000	R	audit	China	AH	U	N	30D	3	114	0.026
939	Xu, K.	A Selective Clipping Microsurgical Treatment for Multiple Intracranial Anterior Circulation Aneurysms	NEURO	ANEUR	N	2014	2005	R	audit	China	Mixed	Mixed	Y	IP	3	146	0.021
940	Xu, Z.	Long-term follow-up with Ross procedure at a single institution in China	CARDI	VALVE	N	2014	2001	R	audit	China	AH	U	Y	30D	2	58	0.034
941	Yadav, D.	Spectrum of Perforation Peritonitis in Delhi: 77 Cases Experience	GENSX	PERF	N	2013	2011	P	audit	India	AH	U	N	IP	10	77	0.130
942	Yadav, K.	Evaluation of POSSUM and P-POSSUM as a tool for prediction of surgical outcomes in the Indian population	GENSX	GENS	N	2011	2009	P	audit	India	AH	U	Y	30D	6	100	0.060
943	Yalinkaya, A.	Emergency peripartum hysterectomy: 16-year experience of a medical hospital	OBGYN	EPH	N	2010	2000	R	audit	Turkey	AH	U	N	IP	13	140	0.093
944	Yamasmit, W.	Risk factors for cesarean hysterectomy in tertiary center in Thailand: a case-control study	OBGYN	EPH	N	2009	2004	R	case-control	Thailand	AH	U	N	IP	0	28	0.000
945	Yan, Z.	Surgical treatment of familial adenomatous polyposis: Experience from a single institution in China	GENSX	COLRES	N	2012	1998	R	audit	China	AH	U	N	30D	0	42	0.000
946	Yang, Z.	Clinicopathologic Characteristics and Outcomes of Patients with obstructive colorectal cancer	GENSX	COLRES	N	2011	2001	R	nonrandomized cohort	China	AH	U	Y	30D	85	1672	0.051
947	Yavangi, M.	Effect of Iranian Ministry of Health Protocols on Cesarean Section Rate: A Quasi-Experimental Study	OBGYN	CAES	N	2013	2008	P	RCT	Iran, Islamic Rep.	AH	U	N	IP	0	630	0.000
948	Yeap, B.H.	Neonatal tumours in Malaysia: a call for heightened awareness	PAEDS	ONCOL	N	2010	2004	Mixed R/P	audit	Malaysia	AH	U	N	IP	2	28	0.071
949	Yildirim IO	Prospective evaluation of the factors effective on morbidity and mortality of patients undergoing liver resection surgeries	HEPAT	LIVRES	N	2012	2008	P	audit	Turkey	AH	U	Y	IP	3	34	0.088
950	Yin, L.	Medullary hemangioblastoma: 34 patients at a single institution	NEURO	RIM	N	2013	2008	R	audit	China	AH	U	N	30D	2	34	0.059
951	Ying, F.	Lessons Learnt after 12 Years Experience in Laparoscopic Cholecystectomy at a Single Center	GENSX	CHOLE	N	2010	2003	R	audit	China	AH	U	N	IP	1	2400	0.000
952	Younes, R.N.	Surgical resection of lung metastases:results from 529 patients	THORA	LUNGRES	N	2009	1998	R	audit	Brazil	Other	U	Y	30D	2	776	0.003
953	Yousuf, K.M.	Management of chronic traumatic arteriovenous fistula of the lower extremities	TRAUM	PVI	N	2013	2007	R	audit	Pakistan	AH	U	Y	30D	0	29	0.000
954	Yu, D	Video-assisted thoracic bronchial sleeve lobectomy with bronchoplasty for treatment of lung cancer confined to a single lung lobe: a case series of Chinese patients	THORA	LUNGRES	N	2014	2010	R	audit	China	AH	U	Y	30D	0	9	0.000
955	Yu, J.	Valve replacement in pediatric patients: a single center experience	CARDI	VALVE	N	2011	1999	R	audit	China	AH	U	Y	30D_IP	3	35	0.086
956	Yu, L.	Off-pump versus on-pump coronary artery bypass surgery in patients with triple vessel disease and enlarged ventricles	CARDI	CABG	Y	2014	2009	P	RCT	China	AH	U	Y	IP	10	102	0.098
957	Yu L.	Surgery for chronic total occlusion of the left main coronary artery	CARDI	CABG	Y	2012	2004	R	audit	China	AH	U	N	IP	0	8	0.000

Reference	First Author	Article Title	Specialty	Procedure or Diagnosis	High-risk population?	Year Published	Data midpoint year	Retrospective or prospective	Study design	Country	Type of Facilities Included	Urban/ Rural/ Mix	Was POMR clearly defined?	Timeframe of POMR	POMR numerator	POMR denominator	Overall POMR
958	Yuan, S.	The implications of serum enzymes and coagulation activities in postinfarction myocardial rupture	CARDI	CRUPT	N	2011	2006	R	audit	China	AH	U	N	IP	6	19	0.316
959	Yusoff, A.R.	Survival analysis of cholangiocarcinoma: A 10-year experience in Malaysia	HEPAT	BILD	N	2012	2002	R	nonrandomized cohort	Malaysia	AH	U	Y	30D	1	12	0.083
960	Zafar, H.	Laparoscopic splenectomy for haematological disorder: our experience	GENSX	SPLEEN	N	2012	2011	R	audit	Pakistan	AH	U	N	IP	1	7	0.143
961	Zaman, B.S.	Indications and complication of emergency peripartum hysterectomy in Bahawal Victoria Hospital Bahawalpur	OBGYN	EPH	N	2013	2007	R	audit	Pakistan	AH	U	N	IP	3	24	0.125
962	Zanati, S. G.	Profile of cardiovascular risk factors and mortality in patients with symptomatic peripheral arterial disease	VASCU	BYPASS	N	2009	2002	P	audit	Brazil	AH	U	Y	IP	2	75	0.027
963	Zargar, M.	Liver Trauma: Operative and Non-operative Management	TRAUM	LIVTRAUM	N	2010	1999	R	audit	Iran, Islamic Rep.	Other	U	N	IP	13	54	0.241
964	Zehir, S.	Red cell distribution width and mortality in patients with hip fracture treated with partial prosthesis	ORTHO	HIPFRAC	N	2014	2009	R	audit	Turkey	AH	U	N	30D	34	316	0.108
965	Zhang, C	A retrospective study on the aetiology, management, and outcome of brain abscess in an 11-year, single-centre study from China	NEURO	BABS	N	2014	2006	R	audit	China	AH	U	Y	IP	10	60	0.167
966	Zhang H	Influence of diabetes mellitus on long-term clinical and economic outcomes after coronary artery bypass grafting.	CARDI	CABG	N	2014	2003	P	nonrandomized cohort	China	AH	U	Y	IP	94	9240	0.010
967	Zhang, M.	Pre-hospital statin therapy may not reduce incidence of all-cause mortality and overall MACCE during hospital stay after coronary artery bypass graft surgery.	CARDI	CABG	N	2009	2004	R	audit	China	AH	U	Y	IP	42	2013	0.021
968	Zhang, P.	Completion pneumonectomy for lung cancer treatment: early and long term outcomes	THORA	LUNGRES	N	2012	1997	R	audit	China	AH	U	Y	30D_IP	9	92	0.098
969	Zhang, P.	Surgial treatment of bronchiectasis: A retrospective analysis of 790 patients.	THORA	LUNGRES	N	2010	1998	R	audit	China	AH	U	Y	30D_IP	9	790	0.011
970	Zhang, W.B.	Risk factors of mortality in non-trauma exsanguinating patients that require damage control laparotomyan	GENSX	DCL	N	2010	2004	R	audit	China	AH	U	Y	30D_IP	7	26	0.269
971	Zhang, Z.	Experience in treating congenital esophageal atresia in China	PAEDS	EATEF	N	2010	2007	R	audit	China	AH	U	Y	30D	4	46	0.087
972	Zhang, Z. L.	Radical cystectomy for bladder cancer: Oncologic outcome in 271 Chinese patients	UROLO	RADCYS	N	2014	2005	R	audit	China	AH	U	Y	30D	4	271	0.015
973	Zhang, Z. L.	Complications of radical nephrectomy for renal cell carcinoma: A retrospective study comparing transperitoneal and retroperitoneal approaches using a standardized reporting methodology in two Chinese centers	UROLO	NEPHREC	N	2013	2002	R	audit	China	AH	U	N	IP	1	558	0.002
974	Zheng, Z.	Mortality Risk Model for Heart Valve Surgery in China	CARDI	VALVE	N	2013	2007	P	audit	China	Mixed	Mixed	Y	IP	260	13353	0.019
975	Zheng, Z.	Totally thoracoscopic versus open surgery for closure of atrial septal defect: Propensity-score matched comparison	CARDI	MCHD	N	2014	2011	R	nonrandomized cohort	China	AH	Mixed	Y	IP	0	508	0.000
976	Zheng, Z.	Risk factors and in-hospital mortality in Chinese patients undergoing coronary artery bypass grafting: analysis of a large multi-institutional Chinese database.	CARDI	CABG	N	2012	2007	R	audit	China	Mixed	Mixed	Y	IP	246	9838	0.025
977	Zhong, J. H.	Hepatic resection is safe and effective for patients with hepatocellular carcinoma and portal hypertension	HEPAT	LIVRES	N	2014	2008	R	case-control	China	AH	U	Y	30D	22	1738	0.013
978	Zhong, Y.	The results of surgery for colorectal hepatic metastases following expansion of the indications in 2005	HEPAT	LIVRES	N	2013	2005	R	audit	China	AH	U	Y	30D	6	530	0.011
979	Zhou, X.	Comparison of the posterior approach and anterior approach for a Kugel repair of treatment of inguinal hernias	GENSX	INGHERN	N	2013	2005	R	nonrandomized cohort	China	AH	U	N	IP	0	2381	0.000
980	Zhou, Y. M.	Distal pancreatectomy with en bloc celiac axis resection for pancreatic body-tail cancer: Is it justified?	HEPAT	PANC_RES	N	2014	2009	R	audit	China	AH	Mixed	N	30D	0	12	0.000
981	Zhou, Z	Effect and cost of perioperative use of antibiotics in coronary artery bypass grafting: a randomized controlled study	CARDI	CABG	N	2011	2006	P	RCT	China	AH	U	N	IP	2	614	0.003
982	Zhu, H.	Keyhole Endoscopic Hematoma Evacuation in Patients	NEURO	ICH	N	2012	2008	Mixed R/P	RCT	China	AH	U	Y	30D	5	30	0.167
983	Zhuang, Y.	Early outcomes of isolated coronary artery bypass grafting in Chinese aged patients with diabetes mellitus	CARDI	CABG	N	2009	2004	R	audit	China	AH	U	Y	30D_IP	29	593	0.049
984	Zilberstein, B.	Simplified technique for reconstruction of the digestive tract after total and subtotal gastrectomy for gastric cancer	GENSX	GASTCA	N	2014	2011	R	audit	Brazil	AH	U	N	IP	0	77	0.000
985	Zoumenou, E.	Pediatric anesthesia in developing countries: experience in the two main university hospitals of Benin in West Africa	PAEDS	PAED	N	2010	2007	R	audit	Benin	AH	U	Y	OT	5	512	0.010
High-Income Country studies																	
1	Billeter, A.	Mortality after Elective Colon Resection: The Search for Outcomes that Define Quality in Surgical Practice	GENSX	COLRES	N	2012	2008	P	audit	USA	Mixed	Mixed	Y	IP	1334	85260	0.016
2	Bregendahl, S.	Risk of complications and 30-day mortality after laparoscopic and open appendectomy in a Danish region, 1998-2007; a population-based study of 18,426 patients	GENSX	APPY	N	2013	2002	P	nonrandomized cohort	Denmark	Mixed	Mixed	Y	30D	58	18426	0.003
3	Fair, B.A.	The impact of operative timing on outcomes of appendicitis: a National Surgical Quality Improvement Project analysis	GENSX	APPY	N	2015	2009	P	nonrandomized cohort	USA	Mixed	Mixed	Y	30D	75	69926	0.001
4	Flood, K.M.	Changing trends in peripartum hysterectomy over the last 4 decades	OBGYN	EPH	N	2009	1985	R	nonrandomized cohort	Ireland	AH	U	N	IP	4	358	0.011
5	Ingraham, A.M.	Comparison of Hospital Performance in Nonemergency Versus Emergency Colorectal Operations at 142 Hospitals	GENSX	COLRES	N	2010	2006	P	nonrandomized cohort	USA	Mixed	Mixed	Y	30D	1272	30793	0.041
6	Jakobbson	Emergency peripartum hysterectomy: results from the prospective Nordic Obstetric Surveillance System	OBGYN	EPH	N	2015	2011	P	audit	Multiple	Mixed	Mixed	N	IP	1	211	0.005
7	Knight, M.	Cesarean Delivery and Peripartum Hysterectomy	OBGYN	EPH	N	2008	2005	P	case-control	UK	Mixed	Mixed	N	IP	2	318	0.006

Reference	First Author	Article Title	Specialty	Procedure or Diagnosis	High-risk population?	Year Published	Data midpoint year	Retrospective or prospective	Study design	Country	Type of Facilities Included	Urban/ Rural/ Mix	Was POMR clearly defined?	Timeframe of POMR	POMR numerator	POMR denomiator	Overall POMR
8	Mamidanna, R	Reduced Risk of Medical Morbidity and Mortality in Patients Selected for Laparoscopic Colorectal Resection in England	GENSX	COLRES	N	2012	2004	P	nonrandomized cohort	UK	Mixed	Mixed	Y	IP_30D	4515	138735	0.033
9	Moroz, L.A.	Hospital variation in maternal complications following caesarean delivery in the United States: 2006–2012	OBGYN	CAES	N	2015	2009	P	audit	USA	Mixed	Mixed	Y	IP	197	1339397	0.000
10	New Zealand POMRC	Perioperative Mortality in New Zealand: Sixth report of the Perioperative Mortality Review Committee	GENSX	COLRES	N	2017	2012	P	audit	New Zealand	Mixed	Mixed	Y	30D	782	21196	0.037
11	Schuitemaker, N	Maternal mortality after caesarean section in the Netherlands	OBGYN	CAES	N	1997	1987	P	audit	Netherlands	Mixed	Mixed	Y	6W	57	108587	0.001

Reference Number	Full reference
1	Abalos E, Addo V, Brocklehurst P, El Sheikh M, Farrell B, Gray S, et al. Caesarean section surgical techniques (CORONIS): a fractional, factorial, unmasked, randomised controlled trial. Lancet. 2013;382(9888):234-48.
2	Abantanga FA, Nimako B, Amoah M. The range of abdominal surgical emergencies in children older than 1 year at the Komfo Anokye Teaching Hospital, Kumasi, Ghana. Ann Afr Med. 2009;8(4):236-42.
3	Abasiattai AM, Umoiyoho AJ, Utuk NM, Inyang-Etoh EC, Asuquo OP. Emergency peripartum hysterectomy in a tertiary hospital in southern Nigeria. Pan Afr Med J. 2013;15:60.
4	Abbaszadeh M. The impact of carotid artery disease on outcomes of patients undergoing coronary artery bypass grafting: O impacto da doença da artéria carótida nos resultados de pacientes submetidos à revascularização miocárdica. Rev bras cir cardiovasc. 2011;26(2):258-63.
5	Abd El Maksoud W, Abd El Salam M, Ahmed HH. Comparative study between Lichtenstein procedure and modified darn repair in treating primary inguinal hernia: a prospective randomized controlled trial. Hernia. 2014;18(2):231-6.
6	Abdel Wahab M, Lawal AR, E ELH, Salah T, Hamdy E, Sultan AM. Caudate lobe resection: an Egyptian center experience. Langenbecks Arch Surg. 2009;394(6):1057-63.
7	Abdel-Aleem H, Alhusaini TK, Abdel-Aleem MA, Menoufy M, Gulmezoglu AM. Effectiveness of tranexamic acid on blood loss in patients undergoing elective cesarean section: randomized clinical trial. J Matern Fetal Neonatal Med. 2013;26(17):1705-9.
8	Abdelmolaa AS, Khalil NH, Negm NE, Elkhatib EA, Mohamed KE. Assessment of endoscopic assisted microsurgery in treatment of cerebellopontine angle lesions. Egyptian Journal of Neurology, Psychiatry and Neurosurgery. 2012;49(2):93-8.
9	Abdel-Wahab M, El-Husseiny TS, El Hanafy E, El Shobary M, Hamdy E. Prognostic factors affecting survival and recurrence after hepatic resection for hepatocellular carcinoma in cirrhotic liver. Langenbecks Arch Surg. 2010;395(6):625-32.
10	Abdulrahman RI, Parvizi R. Outcome of coronary artery bypass grafts: comparison between on pump and off pump. Acta Med Iran. 2010;48(3):158-63.
11	Abdur-Rahman LO, Adeniran JO, Taiwo JO, Nasir AA, Odi T. Bowel resection in Nigerian children. Afr J Paediatr Surg. 2009;6(2):85-7.
12	Abdur-Rahman LO, Kolawole IK, Adeniran JO, Nasir AA, Taiwo JO, Odi T. Pediatric day case surgery: experience from a tertiary health institution in Nigeria. Ann Afr Med. 2009;8(3):163-7.
13	Abdur-Rahman LO, Nasir AA, Adeniran JO. Circumcision: perspective in a Nigerian teaching hospital. Afr J Paediatr Surg. 2013;10(3):271-4.
14	Abid D, Elloumi A, Abid L, Mallek S, Aloulou H, Chabchoub I, et al. Congenital heart disease in 37,294 births in Tunisia: birth prevalence and mortality rate. Cardiol Young. 2014;24(5):866-71.
15	Abid O, Hakim A, Akram N. Complications after surgical tracheostomy. Journal of Medical Sciences (Peshawar). 2013;21(4):165-7.
16	Abrão FC, Waisberg DR, Fernandez A, Bernardo WM, Pêgo-Fernandes PM, Jatene FB. Desmoid tumors of the chest wall: surgical challenges and possible risk factors. Clinics. 2011;66(4):705-8.
17	Abubakar AM, Bello MA, Chinda JY, Danladi K, Umar IM. Challenges in the management of early versus late presenting congenital diaphragmatic hernia in a poor resource setting. Afr J Paediatr Surg. 2011;8(1):29-33.
18	Adademir T, Tuncer EY, Tas S, Donmez AA, Polat EB, Tuncer A. Surgical treatment of aortic valve endocarditis: a 26-year experience: Tratamento cirúrgico da endocardite da válvula aórtica: 26 anos de experiência. Rev bras cir cardiovasc. 2014;29(1):16-24.
19	Ademuyiwa AOB, Chris O.; Adesanya, Opeoluwa A.; Elebute, Olumide A. Non-Trauma Related Paediatric Abdominal Surgical Emergencies in Lagos, Nigeria: Epidemiology and Indicators of Survival. Niger Med J. 2012;53(2).
20	Ademuyiwa AOS, O. A.; Ijaduola, T. K.; Adejuyigbe, O. Determinants of Mortality in Neonatal Intestinal Obstruction in Ile Ife, Nigeria. Afr J Paediatr Surg. 2009;6(1).
21	Adenekan AT, Faponle AF, Oginni FO. Perioperative adverse airway events in cleft lip and palate repair. Southern African Journal of Anaesthesia and Analgesia. 2011;17(6):370-3.
22	Adeoye PO, Adebola SO, Adesiyun OA, Braimoh KT. Peripheral vascular surgical procedures in Ilorin, Nigeria: indications and outcome. Afr Health Sci. 2011;11(3):433-7.
23	Adhikari S, Hossein MZ, Das A, Mitra N, Ray U. Etiology and outcome of acute intestinal obstruction: a review of 367 patients in Eastern India. Saudi J Gastroenterol. 2010;16(4):285-7.
24	Adigun TA, Adeolu AA. Factors influencing the immediate postoperative outcome in operated head injured patients in Ibadan, Nigeria. Afr J Med Med Sci. 2012;41(3):301-5.
25	Adisa AO, Alatise OI, Arowolo OA, Lawal OO. Laparoscopic appendectomy in a Nigerian teaching hospital. JSLS. 2012;16(4):576-80.
26	Adisa AO, Lawal OO, Alatise OI, Adesunkanmi AR. An audit of laparoscopic surgeries in Ile-Ife, Nigeria. West Afr J Med. 2011;30(4):273-6.
27	Adisa AO, Lawal OO, Arowolo OA, Akinola DO. Laparoscopic cholecystectomy in Ile-Ife, Nigeria. Afr J Med Med Sci. 2011;40(3):221-4.
28	Adisa AO, Lawal OO, Arowolo OA, Alatise OI. Local adaptations aid establishment of laparoscopic surgery in a semiurban Nigerian hospital. Surgical Endoscopy and Other Interventional Techniques. 2013;27(2):390-3.
29	Adoga AA, Ma'an ND. Indications and outcome of pediatric tracheostomy: results from a Nigerian tertiary hospital. BMC Surg. 2010;10:2.
30	Afolayan JM, Nwachukwu CE, Esangbedo ES, Omu PO, Amadasun FE, Fadare JO. Evolving pattern of spinal anaesthesia in stable eclamptic patients undergoing caesarean section at University of Benin Teaching Hospital, Benin, Nigeria. Niger J Med. 2014;23(4):288-95.
31	Afuwape OO, Akute OO, Adebanjo AT. Preliminary experience with laparoscopic cholecystectomy in a nigerian teaching hospital. West Afr J Med. 2012;31(2):120-3.
32	Agrawal D, Joshua SP, Gupta D, Sinha S, Satyarthee GD. Can glasgow score at discharge represent final outcome in severe head injury. Journal of Emergencies, Trauma and Shock. 2012;5(3):217-9.
33	Agu K, Nzegwu M, Obi E. Prevalence, morbidity, and mortality patterns of typhoid ileal perforation as seen at the University of Nigeria Teaching Hospital Enugu Nigeria: an 8-year review. World J Surg. 2014;38(10):2514-8.
34	Aguiar PHPd, Zicarelli CAM, Isolan G, Antunes Á, Aires R, Georgeto SM, et al. Brainstem cavernomas: a surgical challenge: Cavernomas de tronco: desafio cirúrgico. Einstein (São Paulo). 2012;10(1).
35	Aguilar-Nascimento JEd, Bicudo-Salomão A, Caporossi C, Silva RdM, Cardoso EA, Santos TP, et al. Abordagem multimodal em cirurgia colorretal sem preparo mecânico de cólon: Multimodal approach in colorrectal surgery without mechanical bowel cleansing. Rev Col Bras Cir. 2009;36(3):204-9.
36	Ahmad M, Amer S, Alam S, Ullah A, Sadiq MU, Khan MA. Safety of single layer continuous extra mucosal gut anastomosis in emergency. Journal of Postgraduate Medical Institute. 2013;27(1):69-73.
37	Ahmad N, Khan SA, Abid KJ. Management of strangulated inguinal hernia in adults. Pakistan Journal of Medical and Health Sciences. 2014;8(1):34-6.
38	Ahmad N, Khan SA, Abid KJ. Outcome of splenic injuries in adult trauma population. Pakistan Journal of Medical and Health Sciences. 2013;7(4):878-80.
39	Ahmad T, Ahmed SW, Hussain N, Khan MI. Role of Urgent Thoracotomy in improving the survival of patients with severe chest trauma. J Coll Physicians Surg Pak. 2009;19(9):575-8.
40	Ahmed A, Ali M, Khan M, Khan F. Perioperative cardiac arrests in children at a university teaching hospital of a developing country over 15 years. Paediatr Anaesth. 2009;19(6):581-6.
41	Ahmed A, Dauda M, Garba S, Ukwenya Y. Emergency abdominal surgery in Zaria, Nigeria. S Afr J Surg. 2010;48(2):59-62.
42	Ahmed A, Ukwenya AY, Makama JG, Mohammad I. Management and outcome of gastric carcinoma in Zaria, Nigeria. Afr Health Sci. 2011;11(3):353-61.
43	Ahmed A. Trends in emergency surgical admissions in a tertiary health centre in Nigeria. West Afr J Med. 2009;28(2):106-9.
44	Ahmed AZ, Zohdi AM, Zaghloul MS, Elsamman AK. Endovascular coiling versus surgical clipping in the treatment of ruptured anterior communicating artery aneurysm in Cairo University Hospitals. Egyptian Journal of Radiology and Nuclear Medicine. 2013;44(3):523-30.
45	Aikawa P, Cintra ARS, Leite CA, Marques RH, Silva CTMd, Afonso MdS, et al. Impact of coronary artery bypass grafting in elderly patients: Impacto da cirurgia de revascularização do miocárdio em pacientes idosos. Rev bras cir cardiovasc. 2013;28(1):22-8.

Reference Number	Full reference	
46	Aisuodionoe-Shadracha OI, Akporiaye LE. Outcome of the TURP-TUVP sandwich procedure for minimally invasive surgical treatment of benign prostatic hyperplasia with volume larger than 40cc over a 4-year period in Nigeria. African Journal of Urology. 2013;19(1):22-5.	
47	Ajami GH, Amirghofran AA, Borzouee M, Navvabi MA, Amoozgar H, Sarikhani S. Coronary artery origin anomalies with especial emphasis on delayed diagnosis of anomalous origin of the left coronary artery from pulmonary artery, a report from south of Iran. Iranian Cardiovascular Research Journal. 2009;3(1):43-8.	
48	Akaba GO, Onafowokan O, Offiong RA, Omonua K, Ekele BA. Uterine rupture: trends and feto-maternal outcome in a Nigerian teaching hospital. Niger J Med. 2013;22(4):304-8.	
49	Akar AR, Kurtcephe M, Sener E, Alhan C, Durdu S, Kunt AG, et al. Validation of the EuroSCORE risk models in Turkish adult cardiac surgical population. Eur J Cardiothorac Surg. 2011;40(3):730-5.	
50	Akhtar RP, Abid AR, Naqshband MS, Mohydin BS, Khan JS. Outcome of double vs. single valve replacement for rheumatic heart disease. J Coll Physicians Surg Pak. 2011;21(1):9-14.	
51	Akhtar RP, Abid AR, Nuqshband MS, Khan JS. Clinical outcome of double valve repalcement with or without small aortic prosthesis. J Coll Physicians Surg Pak. 2012;22(10):617-21.	NB this study has an overlapping, but non-identical population to Akhtar (2011). Excluding this study does not influence POMR estimates.
52	Akinci M, Ergul Z, Kulah B, Yilmaz KB, Kulacoglu H. Risk factors related with unfavorable outcomes in groin hernia repairs. Hernia. 2010;14(5):489-93.	
53	Akinci M, Yilmaz KB, Kulah B, Seker GE, Ugurlu C, Kulacoglu H. Association of ventral incisional hernias with comorbid diseases. Chirurgia (Bucur). 2013;108(6):807-11.	
54	Alaei F, Moghadam MYA, Mortaezaian H, Alaei M, Bakhshandeh H. Balloon angioplasty versus surgical repair of coarctation of aorta in infants. Journal of Tehran University Heart Center. 2011;6(3):134-7.	
55	Alatise OI, Lawal OO, Adesunkanmi AK, Osasan SA. Surgical outcome of abdominoperineal resection for low rectal cancer in a Nigerian tertiary institution. World J Surg. 2009;33(2):233-9.	
56	Albsoul NM, BaniHani MN, Al-Muqbel K, AlWaqfi NR, Al Manasra AR, Hamdan FF, et al. Hurthle cell carcinoma:Expanded view. Jordan Medical Journal. 2009;43(4):333-40.	
57	Alemayehu W, Ballard K, Wright J. Primary repair of obstetric uterine rupture can be safely undertaken by non-specialist clinicians in rural Ethiopia: A case series of 386 women. BJOG. 2013;120(4):505-8.	
58	Alexander PV, Rajkumar D. The pattern of hydatid disease-a retrospective study from himachal pradesh, India. Indian J Surg. 2010;72(4):331-5.	
59	Ali AM, Sayd HA, Hamza HM, Salem MA. Role of surgery in stages II and III pediatric abdominal Non-Hodgkin lymphoma: A 5-years experience. Cancers (Basel). 2011;3(2):1593-604.	
60	Ali M, Khan A, Khan H, Khwanzada K. Short-term complications of ventriculoperitoeal shunt in children suffering from hydrocephalus. J Pediatr Neurol. 2009;7(2):165-9.	
61	Ali N, Aliyu S. Appendicitis and its surgical management experience at the University of Maiduguri Teaching Hospital Nigeria. Niger J Med. 2012;21(2):223-6.	
62	Ali N, Gali BM. Causes and treatment outcome of perforation peritonitis in north eastern Nigeria. Surgical Practice. 2010;14(3):92-6.	
63	Ali N, Madziga AG, Dogo D, Gali BM, Gadzama AA. Outcome of surgery for toxic goitres in Maiduguri: a single teaching hospital's perspective. Niger J Clin Pract. 2012;15(3):280-4.	
64	Ali R, Ahmed S, Qadir M, Maheshwari P, Khan R. Pneumothoraces in a neonatal tertiary care unit: case series. Oman Med J. 2013;28(1):67-9.	
65	Aliev AR. Strategic aspects of stomach cancer surgery. Georgian Med News. 2011(192):12-7.	
66	Allam IS, Gomaa IA, Fathi HM, Sukkar GFM. Incidence of emergency peripartum hysterectomy in Ain-shams University Maternity Hospital, Egypt: a retrospective study. Arch Gynecol Obstet. 2014.	
67	Almeida RMS. Remodelamento reverso cirúrgico do ventrículo esquerdo: seguimento de 111 meses: Surgical reverse remodelling of the left ventricle: 111 months of follow-up. Rev bras cir cardiovasc. 2009;24(4):470-7.	
68	Al-Mogheer B, Ammar W, Bakoum S, Elarousy W, Rizk H. Predictors of inhospital mortality in patients with infective endocarditis. Egyptian Heart Journal. 2013;65(3):159-62.	
69	Al-Qubati FAA, Damag A, Noman T. Incidence and outcome of pulmonary complications after open cardiac surgery, Thowra Hospital, Cardiac center, Sana'a, Yemen. Egyptian Journal of Chest Diseases and Tuberculosis. 2013;62(4):775-80.	
70	Alsmady MM, Ennab RM, Hassuneh SS, Massad IM, Bustami BB, AbuAbeeleh MA. Early and mid-term evaluation of mechanical heart valve replacement. Kuwait Med J. 2010;42(1):55-9.	
71	Alves AC, Queiroz FLd, Oliveira TANd, Franca-Neto PR, Lamounier PCdC, Lacerda-Filho A. Implementation of a colorectal laparoscopic surgical program - short-term outcomes and conversion rates: Implementacao de programa de cirurgia colorretal laparoscopica - resultados de curto prazo e taxas de conversao. J coloproctol (Rio J, Impr). 2013;33(4):210-5.	
72	Alves AP, de Oliveira PG, de Oliveira JM, de Mesquita DM, Dos Santos JH. Long-term results of the Modified Thal procedure in patients with chagasic megaesophagus. World J Surg. 2014;38(6):1425-30.	
73	Alvi AR, Jawad A, Fazal F, Sayyed R. Fulminant amoebic colitis: A rare fierce presentation of a common pathology. Trop Doct. 2013;43(2):80-2.	
74	Alvi AR, Sheikh GM, Kazim SF. Delayed surgical therapy reduces mortality in patients with acute necrotizing pancreatitis. J Pak Med Assoc. 2011;61(10):973-7.	
75	Alvi AR. Impact of rural medical care in remote mountainous region of pakistan: Challenges and opportunities. Pakistan Journal of Medical Sciences. 2009;25(1):131-6.	
76	AlWaqfi N, Khader Y, Ibrahim K. Coronary artery bypass: predictors of 30-day operative mortality in Jordanians. Asian Cardiovasc Thorac Ann. 2012;20(3):245-51.	
77	Amaral FTV, Rodrigues AJ, Manso PH, Schmidt A, Kolachinski MF, Carbone C, et al. Adult congenital heart disease: experience with the surgical approach: Cardiopatia congênita no adulto: experiência com a abordagem cirúrgica. Rev bras cir cardiovasc. 2013;28(3):371-9.	
78	Ameh EA, Mshelbwala PM. Challenges of managing paediatric abdominal trauma in a Nigerian setting. Niger J Clin Pract. 2009;12(2):192-5.	
79	Aminmansour B, Rezvany M, Sharifi D, Shemshaki H. Effect of decompressive hemicraniectomy on mortality of malignant middle cerebral artery infarction. J Res Med Sci. 2010;15(6):344-7.	
80	Amir D, Amin N. Frequency of complications due to laparoscopic cholecystectomy in hamedan hospitals. J Pak Med Assoc. 2012;62(1).	
81	Amponsah G. Challenges of anaesthesia in the management of the surgical neonates in Africa. Afr J Paediatr Surg. 2010;7(3):134-9.	
82	Amudhan A, Rajendran S, Raj VV, Rajarathinam G, Jyotibasu D, Ravichandran P, et al. Management of esophageal perforation: experience from a tertiary center in India. Dig Surg. 2009;26(4):322-8.	
83	Andrade IN, Moraes Neto FR, Andrade TG. Use of EuroSCORE as a predictor of morbidity after cardiac surgery. Rev Bras Cir Cardiovasc. 2014;29(1):9-15.	
84	Andrade IN, Moraes Neto FR, Oliveira JP, Silva IT, Andrade TG, Moraes CR. Assesment of the EuroSCORE as a predictor for mortality in valve cardiac surgery at the Heart Institute of Pernambuco. Rev Bras Cir Cardiovasc. 2010;25(1):11-8.	
85	Andreollo NA, Lopes LR, Coelho Neto JdS, Silveira HJV. Roux-en-Y gastroenteroanastomosis in the treatment of stenosing and advanced gastric adenocarcinoma: Gastroenteroanastomose em Y de Roux no tratamento do adenocarcinoma gástrico avançado e estenosante. ABCD arq bras cir dig. 2010;23(2):90-3.	
86	Andreollo NA, Tercioti V, Lopes LR, Coelho-Neto JS. Neoadjuvant chemoradiotherapy and surgery compared with surgery alone in squamous cell carcinoma of the esophagus. Arq Gastroenterol. 2013;50(2):101-6.	
87	Andrews NB. Neurosurgical procedures in Jehovah's Witnesses: the Tema experience. West Afr J Med. 2009;28(3):148-50.	
88	Ansar A, Malik T, Shuja S, Khan S. Hysterectomy as a management option for morbidly adherent placenta. J Coll Physicians Surg Pak. 2014;24(5):318-22.	
89	Anwar ul H, Ubaidullah, Akhter N, Samiullah, Javeria, Jan IA, et al. Factors affecting survival in patients with oesophageal atresia and tracheo-oesophageal fistula. J Ayub Med Coll Abbottabad. 2009;21(4):129-33.	

Reference Number	Full reference
90	Arantes Junior AA, Silva Junior GAd, Malheiros JA, Goncalves FF, Magaldi M, Santiago E, et al. A new expansive two-open-doors laminoplasty for multilevel cervical spondylotic myelopathy: technical report and follow-up results: Nova tecnica de laminoplastia expansiva para tratamento de mielopatia espondilotica cervical: descricao tecnica e resultados de follow-up. Arq neuropsiquiatr. 2014;72(1):49-54.
91	Aras M, Altas M, Yilmaz A, Serarslan Y, Yilmaz N, Yengil E, et al. Being a neighbor to Syria: a retrospective analysis of patients brought to our clinic for cranial gunshot wounds in the Syrian civil war. Clin Neurol Neurosurg. 2014;125:222-8.
92	Araujo SEA, Seid VE, Dumarco RB, Nahas CSR, Nahas SC, Cecconello I. Surgical outcomes after preceptored laparoscopic colorectal surgery: Results of a Brazilian preceptorship program. Hepatogastroenterology. 2009;56(96):1651-5.
93	Archana KB, S.P. A clinical review of emergency obstetric hysterectomy. J Obstet Gynaecol India. 2009;59:427-31.
94	Ardeshiri M, Faritous Z, Ojaghi Haghighi Z, Hosseini S, Baghaei R. Effect of obesity on mortality and morbidity after coronary artery bypass grafting surgery in Iranian patients. Anesth Pain Med. 2014;4(2):e18884.
95	Arinc S, Kosif A, Ertugrul M, Arpag H, Alpay L, Unal O, et al. Evaluation of pulmonary hydatid cyst cases. Int J Surg. 2009;7(3):192-5.
96	Arnold M, Moore SW, Sidler D, Kirsten GF. Long-term outcome of surgically managed necrotizing enterocolitis in a developing country. Pediatr Surg Int. 2010;26(4):355-60.
97	Arveen S, Jagdish S, Kadambari D. Perforated peptic ulcer in South India: an institutional perspective. World J Surg. 2009;33(8):1600-4.
98	Asadi-Pooya AA, Malekmohamadi Z, Kamgarpour A, Rakei SM, Taghipour M, Ashjazadeh N, et al. Corpus callosotomy is a valuable therapeutic option for patients with Lennox-Gastaut syndrome and medically refractory seizures. Epilepsy and Behavior. 2013;29(2):285-8.
99	Asanin B. Traumatic epidural hematomas in posterior cranial fossa. Acta Clin Croat. 2009;48(1):27-30.
100	Asefa Z, A Ge. Perforated peptic ulcer disease in Zewditu Hospital. Ethiop Med J. 2012;50(2):145-51.
101	Ashfaq A, Zia HA, Amanullah MM. Is early correction of congenital ventricular septal defect a better option in a developing country? J Pak Med Assoc. 2010;60(4):324-7.
102	Ashimi AO, Omole-Ohonsi A, Ugwa AE, Amole TG. A prospective surveillance of ruptured uterus in a rural tertiary health facility in northwest Nigeria. J Matern Fetal Neonatal Med. 2014;27(16):1684-7.
103	Asicioglu O, Gungorduk K, Yildirim G, Asicioglu BB, Gungorduk OC, Ark C, et al. Second-stage vs first-stage caesarean delivery: Comparison of maternal and perinatal outcomes. J Obstet Gynaecol. 2014;34(7):598-604.
104	Asicioglu O, Sahbaz A, Gungorduk K, Yildirim G, Asicioglu BB, Ulker V. Maternal and perinatal outcomes in women with placenta praevia and accreta in teaching hospitals in Western Turkey. J Obstet Gynaecol. 2014;34(6):462-6.
105	Aslam V, Bilal A, Khan A, Bilal M, Ahmed M, Abidin Z. Transhiatal oesophagectomy. Journal of Postgraduate Medical Institute. 2009;23(2):164-9.
106	Aslar AK, Ozdemir S, Mahmoudi H, Kuzu MA. Analysis of 230 cases of emergent surgery for obstructing colon cancer--lessons learned. J Gastrointest Surg. 2011;15(1):110-9.
107	Atik FA, Garcia MFMA, Santos LM, Chaves RB, Faber CN, Corso RB, et al. Resultados da implementação de modelo organizacional de um serviço de cirurgia cardiovascular: Results of the establishment of an organizational model in a cardiovascular surgery service. Rev bras cir cardiovasc. 2009;24(2):116-25.
108	Avila WS, Gouveia AM, Pomerantzeff P, Bortolotto MR, Grinberg M, Stolf N, et al. Maternal-fetal outcome and prognosis of cardiac surgery during pregnancy. Arq Bras Cardiol. 2009;93(1):9-14.
109	Awan MS, Talpur AA, Jabeen F, Awan AH, Khaskheli NM. Minicholecystectomy versus laproscopic cholecystectomy. Medical Channel. 2011;17(2):68-71.
110	Aydin E, Arslan A, Ozkokeli M. Comparison of superior septal approach with left atriotomy in mitral valve surgery: Comparação da abordagem septal superior com atriotomia esquerda em cirurgia valvar mitral. Rev bras cir cardiovasc. 2014;29(3):367-73.
111	Azarfarin R, Pourafkari L, Parvizi R, Alizadehasl A, Mahmoodian R. Off-pump coronary artery bypass surgery in severe left ventricular dysfunction. Asian Cardiovasc Thorac Ann. 2010;18(1):44-8.
112	Azeredo LG, Veronese ET, Santiago JAD, Brandão CMdA, Pomerantzeff PMA, Jatene FB. Late outcome analysis of the Braile Biomédica® pericardial valve in the aortic position: Avaliação dos resultados tardios da bioprótese de pericárdio bovino Braile Biomédica® em posição aórtica. Rev bras cir cardiovasc. 2014;29(3):316-21.
113	Badejoko OO, Adeyemi AB, Kuti O, Ijarotimi AO, Loto OM, Awowole IO, et al. Operative gynecologic laparoscopy in Ile-Ife, Nigeria: Preliminary experience. J Gynecol Surg. 2013;29(4):186-9.
114	Badejoko OO, Awowole IO, Ijarotimi AO, Badejoko BO, Loto OM, Ogunniyi SO. Obstetric hysterectomy: Trend and outcome in Ile-Ife, Nigeria. J Obstet Gynaecol. 2013;33(6):581-4.
115	Badrinath R, Kakembo N, Kisa P, Langer M, Ozgediz D, Sekabira J. Outcomes and unmet need for neonatal surgery in a resource-limited environment: Estimates of global health disparities from Kampala, Uganda. J Pediatr Surg. 2014;49(12):1825-30.
116	Bagheri J, Jebelli M, Valeshabad AK, Bagheri A, Mandegar MH. Impacts of non-dialysis-dependent renal insufficiency on the early surgical outcomes after isolated coronary artery bypass graft surgery. Turk Gogus Kalp Damar Cerrahisi Dergisi-Turkish Journal of Thoracic and Cardiovascular Surgery. 2013;21(4):911-7.
117	Bagheri J, Rezakhanloo F, Valeshabad AK, Bagheri A. Effects of body mass index on the early surgical outcomes after coronary artery bypass grafting. Turk Gogus Kalp Damar Cerrahisi Dergisi-Turkish Journal of Thoracic and Cardiovascular Surgery. 2014;22(2):253-9.
118	Bagheri J, Sarzaeem MR, Valeshabad AK, Bagheri A, Mandegar MH. Effect of sex on early surgical outcomes of isolated coronary artery bypass grafting. Turk Gogus Kalp Damar Cerrahisi Dergisi-Turkish Journal of Thoracic and Cardiovascular Surgery. 2014;22(3):534-9.
119	Bagheri R, Afghani R, Haghi SZ, Fattahi Masoum SH, Sadrizadeh A. Outcome of repair of bronchial injury in 10 patients with blunt chest trauma. Asian Cardiovasc Thorac Ann. 2014.
120	Bagheri R, Haghi SZ, Amini M, Fattahi AS. Pulmonary Hydatid cyst: Analysis of 1024 cases. Tehran University Medical Journal. 2010;68(3):188-93.
121	Bagheri R, Haghi SZ, Fattahi Masoum SH, Bahadorzadeh L. Surgical management of bronchiectasis: analysis of 277 patients. Thorac Cardiovasc Surg. 2010;58(5):291-4.
122	Bahar I, Akgul A, Demirbag AE, Altinay L, Thompson LO, Boran M, et al. Open heart surgery in patients with end-stage renal failure: fifteen-year experience. J Card Surg. 2009;24(1):24-9.
123	Bahebeck J, Sobgui E, Loic F, Nonga BN, Mbanya JC, Sosso M. Limb-threatening and life-threatening diabetic extremities: clinical patterns and outcomes in 56 patients. J Foot Ankle Surg. 2010;49(1):43-6.
124	Baig A, Ishaq M, Kumar A, Ishaq Sheikh M. Pyogenic liver abscess: A five year retrospective study in slums of Karachi. Journal of the Liaquat University of Medical and Health Sciences. 2012;11(1):19-23.
125	Balci AE, Balci TA, Ozyurtan MO. Current surgical therapy for bronchiectasis: surgical results and predictive factors in 86 patients. Ann Thorac Surg. 2014;97(1):211-7.
126	Balderrabano-Saucedo N, Vizcaino-Alarcon A, Sandoval-Serrano E, Segura-Stanford B, Arevalo-Salas LA, de la Cruz LR, et al. Pentalogy of Cantrell: Forty-two Years of Experience in the Hospital Infantil de Mexico Federico Gomez. World J Pediatr Congenit Heart Surg. 2011;2(2):211-8.
127	Bali RS, Verma S, Agarwal PN, Singh R, Talwar N. Perforation peritonitis and the developing world. ISRN Surg. 2014;2014:105492.
128	Bandre E, Kabore RA, Ouedraogo I, Sore O, Tapsoba T, Bambara C, et al. Hirschsprung's disease: management problem in a developing country. Afr J Paediatr Surg. 2010;7(3):166-8.
129	Bani Hani MN, AlWaqfi NR, Heis HA, Bani-Hani KE, Hijazi EA, Rashdan YH, et al. Jejunal disorders: potentially lethal causes of acute abdomen are still overlooked. Surg Laparosc Endosc Percutan Tech. 2009;19(1):39-42.
130	Banieghbal B. Minimally invasive surgery for children with HIV/AIDS. Journal of Laparoendoscopic and Advanced Surgical Techniques. 2009;19(1):97-101.
131	Banu T, Chowdhury TK, Kabir M, Talukder R, Lakhoo K. Bringing Surgery to Rural Children: Chittagong, Bangladesh Experience. World J Surg. 2013;37(4):730-6.
132	Barati M, Talebi-Taher M, Nojomi M, Kerami F. Ten-year experience of rhinocerebral zygomycosis in a teaching hospital in Tehran. Iranian Journal of Clinical Infectious Diseases. 2010;5(3):117-20.

Reference Number	Full reference
133	Barnard BJLR, P. J.; Wyk, H. W. J. van. Mitral Valve Replacement at Tygerberg Hospital: a 5 Year Follow-Up. SA Heart Journal. 2010;7(1).
134	Bartos A, Bartos D, Dunca F, Mocanu L, Zaharie F, Iancu M, et al. Multi-organ resections for colorectal cancer: analysis of potential factors with role in the occurrence of postoperative complications and deaths. Chirurgia (Bucur). 2012;107(4):476-82.
135	Batajoo H, Hazra NK. Laparoscopic versus open appendectomy in acute appendicitis. J Nepal Health Res Counc. 2012;10(22):239-42.
136	Beard JH, Oresanya LB, Akoko L, Mwanga A, Mkony CA, Dicker RA. Surgical task-shifting in a low-resource setting: outcomes after major surgery performed by nonphysician clinicians in Tanzania. World J Surg. 2014;38(6):1398-404.
137	Beerdawood KA, Alhmoud FD, Al Namoura AA, Al Briezat AHS. Primary colorectal anastomosis, no preparation, no stoma needed. Journal of the Bahrain Medical Society. 2014;25(2):93-7.
138	Bekele S, Biluts H. Laparascopic cholecstectomy at Myungsung Christian Medical Center, Ethiopia: a five-years experience. Ethiop Med J. 2012;50(3):251-7.
139	Benjacholmas V, Namchaisiri J, Lertsarpcharoen P, Punnahitananda S, Thaithumyanon P. Short-term outcome of PDA ligation in the preterm infants at King Chulalongkorn Memorial Hospital, Thailand. J Med Assoc Thai. 2009;92(7):909-13.
140	Benzagmout M, Boujraf S, Chakour K, Chaoui M. Potts disease in children. Surg Neurol Int. 2011;2(1).
141	Beudeker N, Broadis E, Borgstein E, Heij HA. The hidden mortality of imperforate anus. Afr J Paediatr Surg. 2013;10(4):302-6.
142	Bezircioglu I, Baloglu A, Cetinkaya B, Pirim B. Do clinical and laboratory parameters effect maternal and fetal outcomes in pregnancies complicated with hemolysis, elevated liver enzymes, and low platelet count syndrome? Journal of the Turkish German Gynecology Association. 2012;13(1):1-7.
143	Bhaijee F, Locketz ML, Krige JEJ. Fibrolamellar hepatocellular carcinoma at a tertiary centre in South Africa A case series. S Afr J Surg. 2009;47(4):108-11.
144	Bhandarwar AH, Bakhshi GD, Tayade MB, Borisa AD, Thadeshwar NR, Gandhi SS. Surgical response to the 2008 Mumbai terror attack. Br J Surg. 2012;99(3):368-72.
145	Bhatti K, Ali Lashari A, Shaikh F. A clinic-pathologic correlation of elective abdominal hysterectomy at teaching hospital Khairpur, Pakistan. Rawal Medical Journal. 2013;38(2):143-6.
146	Bi YM, Chen XZ, Jing CK, Zhou RB, Gao YF, Yang LB, et al. Safety and Survival Benefit of Surgical Management for Elderly Gastric Cancer Patients. Hepatogastroenterology. 2014;61(134):1801-5.
147	Biccard BM. Factors associated with mortality when chronic betablocker therapy is withdrawn in the peri-operative period in vascular surgical patients: a matched case-control study. Cardiovasc J Afr. 2010;21(2):97-102.
148	Biluts H, Bekele A, Kottiso B, Enqueselassie F, Munie T. In-patient surgical mortality in Tikur Anbessa Hospital: a five-year review. Ethiop Med J. 2009;47(2):135-42.
149	Biro G, Szeberin Z, Nemes A, Acsady G. Cryopreserved homograft and autologous deep vein replacement for infrarenal aorto and iliaco-femoral graft infection: early and late results. J Cardiovasc Surg (Torino). 2011;52(2):169-76.
150	Borracci RA, Rubio M, Celano L, Ingino CA, Allende NG, Guerrero RAA. Prospective validation of EuroSCORE II in patients undergoing cardiac surgery in Argentinean centres. Interact Cardiovasc Thorac Surg. 2014;18(5):539-43.
151	Botianu PV, Gliga M, Moldovan SC, Botianu AM, Bacarea A, Botianu AM, et al. Anatomo-clinical analysis of 14 consecutive cases of primary cystic mesenterico-epiploic tumors. Chirurgia (Bucur). 2014;109(5):644-8.
152	Bouassida M, Feidi B, Mroua B, Chtourou MF, Sassi S, Chebbi F, et al. Histopathologic characteristics and short-term outcomes of colorectal cancer in young Tunisian patients: one center's experience. Pan Afr Med J. 2012;12:10.
153	Breda JR, Ragognette RG, Breda ASCR, Gurian DB, Horiuti L, Machado LN, et al. Avaliação inicial da ablação operatória biatrial por radiofrequência de fibrilação atrial: Surgical biatrial ablation of atrial fibrillation: initial results. Rev bras cir cardiovasc. 2010;25(1):45-50.
154	Briand V, Dumont A, Abrahamowicz M, Sow A, Traore M, Rozenberg P, et al. Maternal and perinatal outcomes by mode of delivery in senegal and mali: a cross-sectional epidemiological survey. PLoS One. 2012;7(10):e47352.
155	Bukar M, Audu BM, Massa AA. Caesarean delivery at the Federal Medical Centre Gombe: a 3-year experience. Niger J Med. 2009;18(2):179-83.
156	Bunchungmongkol N, Punjasawadwong Y, Chumpathong S, Somboonviboon W, Suraseranivongse S, Vasinanukorn M, et al. Anesthesia-related cardiac arrest in children: the Thai Anesthesia Incidents Study (THAI Study). J Med Assoc Thai. 2009;92(4):523-30.
157	Cai XJ, Wang YF, Liang YL, Yu H, Liang X. Laparoscopic left hemihepatectomy: a safety and feasibility study of 19 cases. Surg Endosc. 2009;23(11):2556-62.
158	Caliskan ACA, E.; Aytan, H.; Demirturk, F. Emergency peripartum hysterectomy: a 4-year review. Nobel Medicus. 2010;6:20-3.
159	Camille A, Evelyne AK, Martial AE, Denise K, Marie-Josee TA, Emmanuel K. Advantages of early management of facial clefts in Africa. Int J Pediatr Otorhinolaryngol. 2014;78(3):504-6.
160	Campos FG, Calijuri-Hamra MC, Imperiale AR, Kiss DR, Nahas SC, Cecconello I. Locally advanced colorectal cancer: results of surgical treatment and prognostic factors: Câncer colorretal localmente avançado: resultados do tratamento cirúrgico e fatores prognósticos. Arq gastroenterol. 2011;48(4):270-5.
161	Campos FG, Perez RO, Imperiale AR, Seid VE, Nahas SC, Cecconello I. Surgical treatment of familial adenomatous polyposis: ileoretal anastomosis or restorative proctolectomy?: Tratamento cirúrgico da polipose adenomatosa familiar: anastomose íleo-retal ou bolsa ileal? Arq gastroenterol. 2009;46(4):294-9.
162	Campos FG, Valarini R. Evolution of laparoscopic colorectal surgery in Brazil: results of 4744 patients from the national registry. Surg Laparosc Endosc Percutan Tech. 2009;19(3):249-54.
163	Caputti GM, Palma JH, Gaia DF, Buffolo E. Off-pump coronary artery bypass surgery in selected patients is superior to the conventional approach for patients with severely depressed left ventricular function. Clinics. 2011;66(12):2049-53.
164	Caronna R, Boukari AK, Zaongo D, Hessou T, Gayito RC, Ahononga C, et al. Comparative analysis of primary repair vs resection and anastomosis, with laparostomy, in management of typhoid intestinal perforation: results of a rural hospital in northwestern Benin. BMC Gastroenterol. 2013;13:102.
165	Carosella VC, Navia JL, Al-Ruzzeh S, Grancelli H, Rodriguez W, Cardenas C, et al. The first Latin-American risk stratification system for cardiac surgery: can be used as a graphic pocket-card score. Interact Cardiovasc Thorac Surg. 2009;9(2):203-8.
166	Casarim ALM, Tincani AJ, Del Negro A, Aguiar CG, Fanni RV, Martins AS. Carotid body tumor: retrospective analysis on 22 patients: Tumor de corpo carotídeo: análise retrospectiva de 22 pacientes. São Paulo med j. 2014;132(3):133-9.
167	Cawich SO, Mohanty SK, Simpson LK, Bonadie KO. Is emergent laparoscopic cholecystectomy for acute cholecystitis safe in a low volume resource poor setting? International Journal of Surgery. 2014;12(8):798-802.
168	Caylak H, Genc O, Kavakli K, Gurkok S, Gozubuyuk A, Yucel O, et al. Surgical management of bronchiectasis: a collective review of 339 patients with long-term follow-up. Thorac Cardiovasc Surg. 2011;59(8):479-83.
169	Celiku E, Dracini X, Dibra A, Di Matteo FM, Catania A. Rectal cancer surgery. A ten years experience. G Chir. 2010;31(11-12):507-10.
170	Cetin G, Gursoy M, Ugurlucan M, Uzunhasan I, Hatemi AC, Tireli E, et al. Single-institutional 22 years experience on cardiac myxomas. Angiology. 2010;61(5):504-9.
171	Chagas JFS, Aquino JLBd, Pascoal MBN, Teixeira AS, Ferro MMN, Gambaro MCO, et al. Multicentricidade no carcinoma diferenciado da tireóide: Multicentricity in the thyroid differentiated carcinoma. Rev bras otorrinolaringol. 2009;75(1):97-100.
172	Chalya PL, Gilyoma JM, Kanumba ES, Mawala B, Masalu N, Kahima KJ, et al. Dermatological malignancies at a university teaching hospital in north-western tanzania: A retrospective review of 154 cases. Tanzania Journal of Health Research. 2012;14(1).
173	Chalya PL, Kanumba ES, McHembe M. Etiological spectrum and treatment outcome of Obstructive jaundice at a University teaching Hospital in northwestern Tanzania: A diagnostic and therapeutic challenges. BMC Res Notes. 2011;4:147.
174	Chalya PL, Mabula JB, Dass RM, Ngayomela IH, Chandika AB, Mbelenge N, et al. Major limb amputations: a tertiary hospital experience in northwestern Tanzania. J Orthop Surg Res. 2012;7:18.
175	Chalya PL, Mabula JB, Giiti G, Chandika AB, Dass RM, McHembe MD, et al. Splenic injuries at Bugando Medical Centre in northwestern Tanzania: a tertiary hospital experience. BMC Res Notes. 2012;5:59.
176	Chalya PL, Mabula JB, Koy M, Kataraihya JB, Jaka H, Mshana SE, et al. Typhoid intestinal perforations at a University teaching hospital in Northwestern Tanzania: A surgical experience of 104 cases in a resource-limited setting. World J Emerg Surg. 2012;7:4.

Reference Number	Full reference
177	Chalya PL, Mabula JB, Koy M, McHembe MD, Jaka HM, Kabangila R, et al. Clinical profile and outcome of surgical treatment of perforated peptic ulcers in Northwestern Tanzania: A tertiary hospital experience. World J Emerg Surg. 2011;6:31.
178	Chalya PL, McHembe MD, Mshana SE, Rambau PF, Jaka H, Mabula JB. Clinicopathological profile and surgical treatment of abdominal tuberculosis: a single centre experience in northwestern Tanzania. BMC Infect Dis. 2013;13:270.
179	Chalya PLM, M.; Gilyoma, J. M.; Mabula, J. B.; Mawala, B.; Mona, L. Enterocutaneous Fistula: a Tanzanian Experience in a Tertiary Care Hospital. East and Central African Journal of Surgery. 2010;15(2).
180	Chamisa I. Pattern of civilian gunshot wounds in Durban, South Africa. Eur J Trauma Emerg Surg. 2011;37(1):37-40.
181	Chan RPC, Galas FRBG, Hajjar LA, Bello CN, Piccioni MA, Auler Jr JOC. Intensive perioperative glucose control does not improve outcomes of patients submitted to open-heart surgery: a randomized controlled trial. Clinics. 2009;64(1):51-60.
182	Chander J, Mangla V, Vindal A, Lal P, Ramteke VK. Laparoscopic choledochoduodenostomy for biliary stone disease: a single-center 10-year experience. J Laparoendosc Adv Surg Tech A. 2012;22(1):81-4.
183	Chandra D, Gupta A, Nath RK, Kazmi A, Grover V, Gupta VK. Surgical management of anomalous pulmonary venous connection to the superior vena cava - Early results. Indian Heart J. 2013;65(5):561-5.
184	Chao TE, Patel PB, Kikubaire M, Niescierenko M, Hagander L, Meara JG. Surgical Care in Liberia and Implications for Capacity Building. World J Surg. 2014.
185	Chaudhary RK, Bhaduri D, Bhatia M, Hatti S, Ba R, Meva J. Influence of comorbidity in cancer surgery on treatment decisions, postoperative course and oncological outcome. Asia Pac J Clin Oncol. 2013;9(1):47-52.
186	Chau-in W, Hintong T, Rodanant O, Lekprasert V, Punjasawadwong Y, Charuluxananan S, et al. Anesthesia-related complications of caesarean delivery in Thailand: 16,697 cases from the Thai Anaesthesia Incidents Study. J Med Assoc Thai. 2010;93(11):1274-83.
187	Chen B, Zhang B, Zhu C, Ye Z, Wang C, Ma D, et al. Modified McKeown minimally invasive esophagectomy for esophageal cancer: a 5-year retrospective study of 142 patients in a single institution. PLoS One. 2013;8(12):e82428.
188	Chen M, Zhang L, Wei Q, Fu X, Gao Q, Liu X. Peripartum hysterectomy between 2009 and 2010 in Sichuan, China. Int J Gynaecol Obstet. 2013;120(2):183-6.
189	Chen QK, Jiang GN, Ding JA. Surgical treatment for pulmonary aspergilloma: a 35-year experience in the Chinese population. Interact Cardiovasc Thorac Surg. 2012;15(1):77-80.
190	Chen X, Huang F, Xu M, Wang L, Jiang Y, Xiao L, et al. The stented elephant trunk procedure combined total arch replacement for Debakey I aortic dissection: operative result and follow-up. Interact Cardiovasc Thorac Surg. 2010;11(5):594-8.
191	Chen XZ, Hu JK, Liu J, Yang K, Zhou ZG, Wang LL, et al. Comparison of short-term outcomes and perioperative systemic immunity of laparoscopy-assisted and open radical gastrectomy for gastric cancer. J Evid Based Med. 2011;4(4):225-31.
192	Chen ZY, Chen LL, Wu LM. Transcatheter Amplatzer Occlusion and Surgical Closure of Patent Ductus Arteriosus: Comparison of Effectiveness and Costs in a Low-Income Country. Pediatr Cardiol. 2009;30(6):781-5.
193	Chichom Mefire A, Weledji PE, Verla VS, Lidwine NM. Diagnostic and therapeutic challenges of isolated small bowel perforations after blunt abdominal injury in low income settings: analysis of twenty three new cases. Injury. 2014;45(1):141-5.
194	Chigbu B, Onwere S, Kamanu C, Aluka C, Okoro O, Feyi-Waboso P, et al. Lessons learned from the outcome of bloodless emergency laparotomies on Jehovah's Witness women presenting in the extremis with ruptured uterus. Arch Gynecol Obstet. 2009;279(4):469-72.
195	Chinnery GE, Krige JEJ, Kotze UK, Navsaria P, Nicol A. Surgical management and outcome of civilian gunshot injuries to the pancreas. Br J Surg. 2012;99:140-8.
196	Chirdan LB, Uba AF, Pam SD, Edino ST, Mandong BM, Chirdan OO. Sacrococcygeal teratoma: Clinical characteristics and long-term outcome in Nigerian children. Ann Afr Med. 2009;8(2):105-9.
197	Chongsuvivatwong V, Bachtiar H, Chowdhury ME, Fernando S, Suwanrath C, Kor-anantakul O, et al. Maternal and fetal mortality and complications associated with cesarean section deliveries in teaching hospitals in Asia. J Obstet Gynaecol Res. 2010;36(1):45-51.
198	Chowdhary SK, Kandpal D. Minimal access surgery in children:a 5 year study. Indian Pediatr. 2012;49(12):971-4.
199	Chowdhury FH, Haque MR, Sarker MH. Intracranial epidermoid tumor; microneurosurgical management: An experience of 23 cases. Asian J Neurosurg. 2013;8(1):21-8.
200	Chu K, Cortier H, Maldonado F, Mashant T, Ford N, Trelles M. Cesarean section rates and indications in sub-Saharan Africa: a multi-country study from Medecins sans Frontieres. PLoS One. 2012;7(9):e44484.
201	Chu K, Havet P, Ford N, Trelles M. Surgical care for the direct and indirect victims of violence in the eastern Democratic Republic of Congo. Confl Health. 2010;4:6.
202	Chu KM, Ford N, Trelles M. Operative mortality in resource-limited settings: the experience of Medecins Sans Frontieres in 13 countries. Arch Surg. 2010;145(8):721-5.
203	Chu KM, Ford NP, Trelles M. Providing surgical care in Somalia: A model of task shifting. Confl Health. 2011;5:12.
204	Claria RS, Ardiles V, Palavecino ME, Mazza OM, Salceda JA, Bregante ML, et al. Laparoscopic resection for liver tumors: initial experience in a single center. Surg Laparosc Endosc Percutan Tech. 2009;19(5):388-91.
205	Clarke DL, Quazi MA, Reddy K, Thomson SR. Emergency operation for penetrating thoracic trauma in a metropolitan surgical service in South Africa. J Thorac Cardiovasc Surg. 2011;142(3):563-8.
206	Coelho JCU, Valle CL, Ribas BM, Andriguetto LD, Claus CMP. Surgical treatment of cystic neoplasms of the pancreas. Arq Gastroenterol. 2010;47(2):135-40.
207	Colafranceschi AS, Monteiro AJdO, Botelho ESL, Canale LS, Rabischoffsky A, Costa IP, et al. Cirurgia vídeo-assistida para a ablação da fibrilação atrial isolada por radiofrequência bipolar: Videothoracoscopy for isolated atrial fibrillation ablation through bipolar radiofrequency. Arq bras cardiol. 2009;93(4):334-42.
208	Colli BO, Carlotti Junior CG, Assirati Junior JA, Borba LAB, Coelho Junior VdPM, Neder L. Foramen magnum meningiomas: surgical treatment in a single public institution in a developing country: Meningiomas do forame magno: tratamento cirúrgico em uma instituição pública em um país em desenvolvimento. Arq neuropsiquiatr. 2014;72(7):528-37.
209	Conterno LO, Toni SMD, Konkiewitz RG, Guedes ES, Barros RTd, Tiveron MG. Impact of hospital infections on patients outcomes undergoing cardiac surgery at Santa Casa de Misericórdia de Marília: Impacto das infecções hospitalares na evolução de pacientes submetidos à cirurgia cardíaca na Santa Casa de Misericórdia de Marília. Rev bras cir cardiovasc. 2014;29(2):167-76.
210	Corciova FC, Corciova C, Georgescu CA, Enache M, Anghel D, Bartos O, et al. Echocardiographic predictors of adverse short-term outcomes after heart surgery in patients with mitral regurgitation and pulmonary hypertension. Heart Surg Forum. 2012;15(3):E127-32.
211	Corrêa Neto IJF, Sia ON, Rolim AS, Souza RFL, Watté HH, Robles L. Clinical outcomes of Fournier's gangrene from a tertiary hospital. J coloproctol (Rio J, Impr). 2012;32(4):407-10.
212	Costa SRP, Henriques AC, Horta SHC, Waisberg J, Speranzini MB. En-bloc pancreatoduodenectomy and right hemicolectomy for treating locally advanced right colon cancer (T4): a series of five patients: Duodenopancreatectomia e hemicolectomia em monobloco para o tratamento do câncer de cólon direito localmente avançado (T4): uma série de cinco doentes. Arq gastroenterol. 2009;46(2):151-3.
213	Cui HW, Liu YR, Zhang BA. Clinical features and treatment outcomes of moyamoya disease in 125 patients of Henan, China. Journal of Neurological Sciences. 2013;30(1):48-58.
214	Cunnigaiper ND, Raj P, Ganeshram P, Venkatesan V. Does Ochsner-Sherren regimen still hold true in the management of appendicular mass? Ulus Travma Acil Cerrahi Derg. 2010;16(1):43-6.
215	Curi-Curi P, Ramírez-Marroquín S, Cervantes-Salazar J, Soulé M, Erdmenger J, Calderón-Colmenero J. Surgical repair of congenital mitral valve malformations: Reparación quirúrgica de las malformaciones congénitas de la válvula mitral^ies. Arch cardiol Méx. 2010;80(2):87-94.
216	Dabdoub CF, Dabdoub CB, Villavicencio R, Quevedo G. How I do it: Myelomeningocele in bolivia. Surg Neurol Int. 2014;5:S39-S48.
217	Dakubo JC, Naaeder SB, Clegg-Lamptey JN. Gastro-duodenal peptic ulcer perforation. East Afr Med J. 2009;86(3):100-9.

Reference Number	Full reference
218	Dalcin RP, Abaid CA, Almeida PM, Adaime SB, Londero TM, Gai LV. Ten-years comparative study after surgical treatment of perforated peptic ulcer according to ulcer relapse between H. Pylori positive, after eradication, and negative patients: Estudo comparativo quanto à recidiva ulcerosa do tratamento cirúrgico de úlcera péptica perfurada entre pacientes H. Pylori positivos, após erradicação, e negativos. ABCD arq bras cir dig. 2009;22(1):15-8.
219	Dan RG, Cretu OM, Mazilu O, Sima LV, Iliescu D, Blidisel A, et al. Postoperative morbidity and mortality after liver resection. Retrospective study on 133 patients. Chirurgia (Bucur). 2012;107(6):737-41.
220	Danisman N, Baser E, Togrul C, Kaymak O, Tandogan M, Gungor T. Emergency peripartum hysterectomy: Experience of a major referral hospital in Ankara, Turkey. J Obstet Gynaecol. 2014:1-3.
221	Dar MI, Dar AH, Almani K, Atta ul M, Khan AQ, Rizwani GH, et al. Coronary artery bypass surgery in old age group: is age itself a barrier? J Pak Med Assoc. 2009;59(9):587-9.
222	Davidov M, Pajic B, Palanacki V, Popov D, Ognjenovic A. Early results of laparoscopic resection of the stomach. Hepatogastroenterology. 2012;59(116):1135-7.
223	Davidovic L, Markovic M, Ilic N, Koncar I, Kostic D, Simic D, et al. Repair of abdominal aortic aneurysms in the presence of the horseshoe kidney. Int Angiol. 2011;30(6):534-40.
224	Davoodi S, Karimi A, Ahmadi SH, Marzban M, Movahhedi N, Abbasi K, et al. Short- and mid-term results of triple-valve surgery with an evaluation of postoperative quality of life. Tex Heart Inst J. 2009;36(2):125-30.
225	Davoodi S, Sheikhvatan M, Karimi A, Ahmadi SH, Goodarzynejad H, Fathollahi MS. Outcomes and long-term quality of life of patients with severe left ventricular dysfunction who underwent coronary artery bypass surgery. Gen Thorac Cardiovasc Surg. 2012;60(4):202-12.
226	De Aguiar PHP, Zicarelli CAM, Aires R, Santiago NM, Tahara A, Simm R, et al. Posterior communicating artery aneurysms: Technical pitfalls. Neurosurg Q. 2010;20(2):74-81.
227	de Almeida AG, Nunes ML, Palmini ALF, da Costa JC. Incidence of SUDEP in a cohort of patients with refractory epilepsy The role of surgery and lesion localization. Arq Neuropsiquiatr. 2010;68(6):898-902.
228	de Araujo Silva DO, Matis GK, Costa LF, Kitamura MA, de Carvalho Junior EV, de Moura Silva M, et al. Chronic subdural hematomas and the elderly: Surgical results from a series of 125 cases: Old "horses" are not to be shot! Surg Neurol Int. 2012;3:150.
229	de Silva W, Ubayasiri R, Weerasinghe C, Wijeyaratne S. Challenges in the management of extremity vascular injuries: A wartime experience from a tertiary centre in Sri Lanka. World J Emerg Surg. 2011;6:24.
230	Deboutte D, O'Dempsey T, Mann G, Faragher B. Cost-effectiveness of caesarean sections in a post-conflict environment: a case study of Bunia, Democratic Republic of the Congo. Disasters. 2013;37:S105-S20.
231	Dehaki MG, Ghavidel AA, Givtaj N, Omrani G, Salehi S. Recurrence rate of different techniques for repair of coarctation of aorta: A 10 years experience. Ann Pediatr Cardiol. 2010;3(2):123-6.
232	Demirci O, Tugrul AS, Yilmaz E, Tosun O, Demirci E, Eren YS. Emergency peripartum hysterectomy in a tertiary obstetric center: nine years evaluation. J Obstet Gynaecol Res. 2011;37(8):1054-60.
233	Dennison MA, James; Abdulla, Mirgani. Caesarean Sections at Juba Teaching Hospital 2008-2009. South Sudan Medical Journal. 2010;3(1).
234	Diaconescu MR, Glod M, Costea I, Grigorovici M, Covic A, Diaconescu S. Surgical management of renal hyperparathyroidism: a preliminary series report. Chirurgia (Bucur). 2011;106(1):51-7.
235	Dias RR, Fernandes F, Ramires FJA, Mady C, Albuquerque CP, Jatene FB. Mortality and Embolic Potential of Cardiac Tumors: Mortalidade e Potencial Embólico dos Tumores Cardíacos. Arq bras cardiol. 2014;103(1):13-8.
236	Dias RR, Mejia OAV, Fernandes F, Ramires FJA, Mady C, Stolf NAG, et al. Mortality Impact of Thoracic Aortic Disease in Sao Paulo State from 1998 to 2007. Arq Bras Cardiol. 2013;101(6):528-34.
237	Dias RR, Mejia OV, Carvalho Jr EV, Lage DOC, Dias AR, Pomerantzeff PMA, et al. Aortic root reconstruction through valve-sparing operation: critical analysis of 11 years of follow-up: Análise crítica da reconstrução da raiz da aorta com a preservação da valva aórtica: 11 anos de seguimento. Rev bras cir cardiovasc. 2010;25(1):66-72.
238	Dinc B, Ay N, Ciyiltepe H. Comparing methods of ileostomy closure constructed in colorectal surgery in Turkey. Prz Gastroenterol. 2014;9(5):291-6.
239	Ding YB, Xia TS, Wu JD, Chen GY, Wang S, Xia JG. Surgical outcomes for gastric cancer of a single institute in southeast China. Am J Surg. 2012;203(2):217-21.
240	Dinkhuysen JJ, Andrade AJPd, Leme J, Silva C, Medina CS, Pereira CC, et al. Clinical evaluation of the Spiral Pump® after improvements to the original project in patients submitted to cardiac surgeries with cardiopulmonary bypass: Avaliação clínica da bomba espiral (Spiral Pump®) após aperfeiçoamentos introduzidos ao projeto original em pacientes submetidos a cirurgias cardíacas com circulação extracorpórea. Rev bras cir cardiovasc. 2014;29(3):330-7.
241	Diom ES, Cisse Z, Tall A, Ndiaye M, Pegbessou E, Ndiaye IC, et al. Management of acquired cholesteatoma in children: A 15 year review in ENT service of CHNU de FANN Dakar. Int J Pediatr Otorhinolaryngol. 2013;77(12):1998-2003.
242	Dongo AE, Kesieme EB, Irabor DO, Ladipo JK. A review of posttraumatic bowel injuries in ibadan. ISRN Surg. 2011;2011:478042.
243	Doumi EBAM, Mohammed Ibrahim. Acute Abdomen at El Obeid Hospital, Western Sudan. Sudan Journal of Medical Science. 2009;4(2).
244	Dracini X, Celiku E, Dibra A, Burazeri G, Kellici S. Surgical treatment of gastric cancer in Albania. Macedonian Journal of Medical Sciences. 2012;5(1):90-3.
245	Du Y, Xu J, Wang G, Shi J, Yang L, Shi S, et al. Comparison of two tranexamic acid dose regimens in patients undergoing cardiac valve surgery. J Cardiothorac Vasc Anesth. 2014;28(5):1233-7.
246	Duan SJ, Liu HS, Niu J, Wang CX, Chen SH, Wang MH. Classifications of clinical and bowel morphological changes and their relationship with characteristics of patients with incarcerated groin hernias. Med Sci Monit. 2014;20:214-8.
247	Duci SB, Arifi HM, Selmani ME, Mekaj AY, Gashi MM, Buja ZA, et al. Surgical Treatment of 55 Patients with Pressure Ulcers at the Department of Plastic and Reconstructive Surgery Kosovo during the Period 2000-2010: A Retrospective Study. Plast Surg Int. 2013;2013:129692.
248	Duishanbai S, Geng D, Liu C, Guo HR, Hao YJ, Liu B, et al. Treatment of intracranial hydatid cysts sailike duishanbai. Chin Med J. 2011;124(18):2954-8.
249	Duttaroy DD, Jitendra J, Duttaroy B, Bansal U, Dhameja P, Patel G, et al. Management strategy for dirty abdominal incisions: Primary or delayed primary closure? A randomized trial. Surg Infect (Larchmt). 2009;10(2):129-36.
250	Edaigbini SA, Delia IZ, Aminu MB, Bosan IB, Ibrahim A, Anumenechi N. Vascular surgeries in West Africa: Challenges and prospects. Asian Cardiovasc Thorac Ann. 2014.
251	Edwin F, Aniteye E, Tettey MM, Tamatey M, Frimpong-Boateng K. Outcome of left heart mechanical valve replacement in West African children--a 15-year retrospective study. J Cardiothorac Surg. 2011;6:57.
252	Edwin F, Sereboe LA, Tettey MM, Aniteye EA, Kotei DA, Tamatey MM, et al. Experience from a single centre concerning the surgical spectrum and outcome of adolescents and adults with congenitally malformed hearts in West Africa. Cardiol Young. 2010;20(2):159-64.
253	Efetie ER, Abubakar JS, Habeeb SA. Audit of gynaecological laparoscopies in National Hospital Abuja, Nigeria. Niger J Clin Pract. 2009;12(2):149-52.
254	Eke AC, Alabi-Isama LI, Akabuike JC. Management options for vulvar carcinoma in a low resource setting. World J Surg Oncol. 2010;8.
255	Ekenze SO, Anyanwu PA, Ezomike UO, Oguonu T. Profile of pediatric abdominal surgical emergencies in a developing country. Int Surg. 2010;95(4):319-24.
256	Ekenze SO, Mgbor SO, Okwesili OR. Routine surgical intervention for childhood intussusception in a developing country. Ann Afr Med. 2010;9(1):27-30.
257	Ekwunife CN, Nwobe O. First 100 Laparoscopic Surgeries in a Predominantly Rural Nigerian Population: A Template for Future Growth. World J Surg. 2014;38(11):2813-7.
258	Ekwunife OH, Oguejiofor IC, Modekwe VI, Osuigwe AN. Jejuno-ileal atresia: a 2-year preliminary study on presentation and outcome. Niger J Clin Pract. 2012;15(3):354-7.
259	El-Baradie M, Khorshid O, Touny A. Adjuvant postoperative radiochemotherapy for patients with gastric carcinoma: A single institution experience. Chinese-German Journal of Clinical Oncology. 2012;11(5):249-56.

Reference Number	Full reference
260	Elias N, Tarasoutchi F, Spina GS, Sampaio RO, Pomerantzeff PMA, Laurindo FR, et al. Fibrose miocárdica e remodelamento ventricular na insuficiência aórtica crônica importante: Fibrosis miocárdica y remodelación ventricular en la insuficiencia aórtica crónica severa^ies: Myocardial fibrosis and ventricular remodeling in severe chronic aortic regurgitation. Arq bras cardiol. 2009;92(1):63-7.
261	Eltayeb AA, Mostafa MM, Ibrahim NH, Eltayeb AA. The role of surgery in management of necrotizing enterocolitis. Int J Surg. 2010;8(6):458-61.
262	Elusoji SO, Iribhogbe PE, Osime OC. Thyroidectomy under ketamine anaesthesia in a semi urban hospital in Nigeria. Pakistan Journal of Medical Sciences. 2009;25(4):695-7.
263	Emmiler M, Yaveri A, Kocogullari CU, Ela Y, Sahin DA, Sivaci RG, et al. Gastrointestinal ischemia related mortality in patients undergoing off- or on-pump coronary artery bypass grafting. Heart Surg Forum. 2009;12(2):E79-84.
264	Engin O, Calik S, Calik B, Yildirim M, Coskun G. Parasitic appendicitis from past to present in Turkey. Iran J Parasitol. 2010;5(3):57-63.
265	Ercan M, Bostanci EB, Dalgic T, Karaman K, Ozogul YB, Ozer I, et al. Surgical outcome of patients with perforation after endoscopic retrograde cholangiopancreatography. Journal of Laparoendoscopic and Advanced Surgical Techniques. 2012;22(4):371-7.
266	Erdogan D, Karaman I, Aslan MK, Karaman A, Cavusoglu YH. Analysis of 3,776 pediatric inguinal hernia and hydrocele cases in a tertiary center. J Pediatr Surg. 2013;48(8):1767-72.
267	Erek E, Yilmaz B, Kaya M, Onan IS, Sen O, Oz K, et al. Analysis of results according to the Aristotle scoring system in congenital heart surgery. Turk Gogus Kalp Damar Cerrahisi Dergisi-Turkish Journal of Thoracic and Cardiovascular Surgery. 2014;22(3):509-16.
268	Eroglu A, Turkyilmaz A, Aydin Y, Yekeler E, Karaoglanoglu N. Current management of esophageal perforation: 20 years experience. Dis Esophagus. 2009;22(4):374-80.
269	Escarain MC, Bozovich GE, Salvatori C, Favaloro RR. The ross procedure: A fifteen-year experience. Rev Argent Cardiol. 2012;80(5):347-53.
270	Esmat M, Abdelaal A, Mostafa D. Application of Yang-Monti Principle in Ileal Ureter Substitution: Is It a beneficial Modification? Int braz j urol. 2012;38(6):779-87.
271	Etonyeaku AC, Agbakwuru EA, Akinkuolie AA, Omotola CA, Talabi AO, Onyia CU, et al. A review of the management of perforated duodenal ulcers at a tertiary hospital in south western Nigeria. Afr Health Sci. 2013;13(4):907-13.
272	Evans CL, Kim YM, Yari K, Ansari N, Tappis H. Using direct clinical observation to assess the quality of cesarean delivery in Afghanistan: An exploratory study. BMC Pregnancy Childbirth. 2014;14(1).
273	Evsen MS, Sak ME, Soydinc HE, Caca FN, Obut M, Gul T. Retrospective analysis of placenta accreta: management strategies - evaluation of 41 cases. Ginekol Pol. 2012;83(7):501-4.
274	Eze JN, Ibekwe PC. Uterine rupture at a secondary hospital in Afikpo, Southeast Nigeria. Singapore Med J. 2010;51(6):506-11.
275	Ezomike UO, Ekenze SO, Amah CC. Outcomes of surgical management of intestinal atresias. Niger J Clin Pract. 2014;17(4):479-83.
276	Ezomike UO, Ituen MA, Ekpemo CS. Indications and outcome of childhood preventable bowel resections in a developing country. Afr J Paediatr Surg. 2014;11(2):97-100.
277	Falase B, Sanusi M, Majekodunmi A, Animasahun B, Ajose I, Idowu A, et al. Open heart surgery in Nigeria; a work in progress. J Cardiothorac Surg. 2013;8:9.
278	Fan HG, Zheng Z, Feng W, Yuan X, Wang W, Hu SS. Repair of left ventricular aneurysm: ten-year experience in Chinese patients. Chin Med J (Engl). 2009;122(17):1963-8.
279	Fantini FA, Gontijo B, Martins C, Lopes RM, Vrandecic EC, Goulart E, et al. Operação de Fontan: uma técnica em evolução: Fontan operation: a technique in evolution. Rev bras cir cardiovasc. 2009;24(4):463-9.
280	Faouzi N, Yosra BA, Said J, Soufiane G, Aouatef C, Rachid K, et al. Intestinal volvulus: aetiology, morbidity and mortality in Tunisian children. Afr J Paediatr Surg. 2011;8(2):147-50.
281	Faria MSM, Caporossi C, Aguilar-Nascimento JEd. Colorectal resection without mechanical colon cleansing: experience with 54 patients. J coloproctol (Rio J, Impr). 2012;32(2):123-6.
282	Fatimi SH, Hanif HM, Ahmed A, Shamsi G, Muzaffar M. Outcomes of surgical management of tracheobronchial injuries---a case series from a developing country. Chin J Traumatol. 2011;14(3):161-4.
283	Fatimi SH, Hanif HM, Awais A, Shamsi G, Muzaffar M. Major thoracic vessels and cardiac trauma: case series from a center in a developing country. Ulus Travma Acil Cerrahi Derg. 2012;18(6):490-4.
284	Fattahi E, Somi MH, Moosapour MR, Fouladi RF. Independent predictors of in-hospital re-bleeding, need of operation and mortality in acute upper gastrointestinal bleeding. Pak J Biol Sci. 2011;14(17):849-53.
285	Fawole AO, Shah A, Fabanwo AO, Adegbola O, Adewunmi AA, Eniayewun AB, et al. Predictors of maternal mortality in institutional deliveries in Nigeria. Afr Health Sci. 2012;12(1):32-40.
286	Fedakar A, Sasmazel A, Bugra O, Sarikaya S, Buyukbayrak F, Erdem H, et al. Results of mitral valve repair in rheumatic mitral lesions. Heart Surg Forum. 2010;13(2):E86-90.
287	Feng B, Liu Y, Hu S, Shen X, Wang X, Wang H, et al. Arterial switch for transposition of the great vessels and Taussig-Bing anomaly after six months of age. Ann Thorac Surg. 2009;88(6):1948-51.
288	Feng JF, Zhou XM, Mao WM. Prognostic analysis of carcinoma of the ampulla of vater: Pancreaticoduodenectomy versus local resection. Hippokratia. 2012;16(1):23-8.
289	Fente BG. Experience working with Nurse Anesthetists' as Non-Physician Anesthesia Providers in a temporary Semi-Urban Niger Delta University Teaching Hospital, Okolobiri and review of the literature. Journal of Dental and Medical Sciences. 2013;3(6):36-40.
290	Fernandes EO, Teixeira C, Silva LCCd. Thoracic surgery: risk factors for postoperative complications of lung resection: Cirurgia torácica: fatores de risco para complicações pós-operatórias na ressecção pulmonar. Rev Assoc Med Bras (1992). 2011;57(3):292-8.
291	Fernandes RHO, Rossi TA. Transanal rectopexy - twelve case studies. J coloproctol (Rio J, Impr). 2012;32(2):132-5.
292	Fernandes RL, Lynch JC, Welling L, Gonçalves M, Tragante R, Temponi V, et al. Complete removal of the spinal nerve sheath tumors. Surgical technics and results from a series of 30 patients: Remoção completa dos tumores da bainha dos nervos raquianos. Técnica cirúrgica e resultados de uma série de 30 pacientes. Arq neuropsiquiatr. 2014;72(4):312-7.
293	Fesseha N, Getachew A, Hiluf M, Gebrehiwot Y, Bailey P. A national review of cesarean delivery in Ethiopia. Int J Gynaecol Obstet. 2011;115(1):106-11.
294	Fofie C, Baffoe P. A two-year review of uterine rupture in a regional hospital. Ghana Med J. 2010;44(3):98-102.
295	Fontana R, Hermam P, Pugliese V, Perini MV, Velho FF, Cecconello I. SURGICAL OUTCOMES AND PROGNOSTIC FACTORS IN PATIENTS WITH SYNCHRONOUS COLORECTAL LIVER METASTASES: Resultados do tratamento cirúrgico e de estudo dos fatores prognósticos em pacientes com metástases hepáticas sincrônicas do câncer colorretal. Arq gastroenterol. 2014;51(1):4-9.
296	Fontes PRO, Waechter FL, Nectoux M, Sampaio JA, Teixeira UF, Pereira-Lima L. LOW MORTALITY RATE IN 97 CONSECUTIVE PANCREATICODUODENECTOMIES: the experience of a group: Baixa mortalidade em 97 duodenopancreatectomias consecutivas: a experiência de um grupo. Arq gastroenterol. 2014;51(1):29-33.
297	Forny DN, Ferrante SMR, Silveira VGd, Siviero I, Chagas VLA, Méio IB. Choledochal cyst in childhood: review of 30 cases: Cisto de colédoco na infância: revisão de 30 casos. Rev Col Bras Cir. 2014;41(5):331-5.
298	Furlanetto G, Furlanetto BHS, Henriques SS, Kapins CEB, Lopes LM, Olmos MCC, et al. Nova técnica: operação de Norwood com perfusão regional cerebral e coronariana: New technique: Norwood operation with regional cerebral and coronary perfusion. Rev bras cir cardiovasc. 2009;24(4):447-52.
299	Gadelhak N, Shehta A, Hamed H. Diagnosis and management of choledochal cyst: 20 years of single center experience. World J Gastroenterol. 2014;20(22):7061-6.
300	Gajin P, Radak D, Tanaskovic S, Babic S, Nenezic D. Urgent carotid endarterectomy in patients with acute neurological ischemic events within six hours after symptoms onset. Vascular. 2014;22(3):167-73.
301	Gala T, Alvi AR, Sheikh GM, Habib HY, Ghafoor Z, Mir TA, et al. Experience of managing complicated diverticulitis of colon: a retrospective case series from South Asian country. J Pak Med Assoc. 2014;64(4):409-14.

Reference Number	Full reference
302	Galukande M, Sekavuga DB, Duffy K, Wooding N, Rackara S, Nakaggwa F, et al. Mass safe male circumcision: Early lessons from a Ugandan urban site - A case study. Pan Afr Med J. 2012;13:88.
303	Ganie FA, Lone H, Wani ML, Wani N, Ahangar AG, Ganie SA. The increasing rate of secondary amputation in popliteal arterial injury associated with multi-organ injuries and hypotension. International Cardiovascular Research Journal. 2012;6(4):124-7.
304	Ganigara M, Prabhu A, Varghese R, Pavithran S, Valliatu J, Nair RS. Extracardiac Fontan operation after late bidirectional Glenn shunt. Asian Cardiovasc Thorac Ann. 2010;18(3):253-9.
305	Gao JS, Wang ZJ, Zhao B, Ma SZ, Pang GY, Na DM, et al. Inguinal hernia repair with tension-free hernioplasty under local anesthesia. Saudi Med J. 2009;30(4):534-6.
306	Gao X, Zhang R, Mao Y, Wang Y. Childhood and juvenile meningiomas. Childs Nerv Syst. 2009;25(12):1571-80.
307	Garofallo SB, Machado DP, Rodrigues CG, Bordim Jr O, Kalil RAK, Portal VL. Applicability of Two International Risk Scores in Cardiac Surgery in a Reference Center in Brazil: Aplicabilidade de Dois Escores de Risco Internacionais em Cirurgia Cardíaca em Centro de Referência Brasileiro. Arq bras cardiol. 2014;102(6):539-48.
308	Gathura E, Poenaru D, Bransford R, Albright AL. Outcomes of ventriculoperitoneal shunt insertion in Sub-Saharan Africa. J Neurosurg Pediatr. 2010;6(4):329-35.
309	Gerges SS, Seleem MI, Ahmed AE, Eldin SS, El-Atrebi KA, Baky AMA, et al. Laparoscopic cholecystectomy in liver cirrhosis patients: An Egyptian experience. Surgical Practice. 2012;16(1):12-6.
310	Gessessew A, Barnabas GA, Prata N, Weidert K. Task shifting and sharing in Tigray, Ethiopia, to achieve comprehensive emergency obstetric care. Int J Gynaecol Obstet. 2011;113(1):28-31.
311	Ghandehari K, Ahmadi F, Afzalnia A. Results of surgical and nonsurgical treatment of aneurysms in a developing country. Stroke Res Treat. 2011;2011:560831.
312	Gharsallah H, Trabelsi W, Hajjej Z, Nasri M, Lebbi A, Jebali MA, et al. Cardiac surgery in patients on hemodialysis: eight years experience of the Tunisian military hospital. Saudi J Kidney Dis Transpl. 2010;21(6):1157-64.
313	Ghezzi TL, Moschetti L, Corleta OC, De Abreu GP, De Abreu LP. Analysis of the videolaparoscopy potentiality in the surgical treatment of the bowel obstruction. Arq Gastroenterol. 2010;47(2):148-51.
314	Ghodsi M, Mortazavi A, Shahjouei S, Hanaei S, Esmaeili A, Nejat F, et al. Exophytic glioma of the medulla: presentation, management and outcome. Pediatr Neurosurg. 2013;49(4):195-201.
315	Ghosh S, Sharma S, Gadpayle AK, Gupta HK, Mahajan RK, Sahoo R, et al. Clinical, laboratory, and management profile in patients of liver abscess from northern India. J Trop Med. 2014;2014:142382.
316	Ghritlaharey RK, Budhwani KS, Shrivastava DK. Exploratory laparotomy for acute intestinal conditions in children: a review of 10 years of experience with 334 cases. Afr J Paediatr Surg. 2011;8(1):62-9.
317	Gil J, Rodriguez JM, Gil E, Balsalobre MD, Hernandez Q, Gonzalez FM, et al. Surgical treatment of endemic goiter in a nonhospital setting without general anesthesia in Africa. World J Surg. 2014;38(9):2212-6.
318	Gilyoma JM, Balumuka DD, Chalya PL. Ten-year experiences with Tracheostomy at a University teaching hospital in Northwestern Tanzania: A retrospective review of 214 cases. World J Emerg Surg. 2011;6(1):38.
319	Gilyoma JM, Hauli KA, Chalya PL. Cut throat injuries at a university teaching hospital in northwestern Tanzania: a review of 98 cases. BMC Emerg Med. 2014;14:1.
320	Gnanappa GK, Ganigara M, Prabhu A, Varma SK, Murmu U, Varghese R, et al. Outcome of complex adult congenital heart surgery in the developing world. Congenit Heart Dis. 2011;6(1):2-8.
321	Gomes WJ, Saavedra RE, Garanhão DM, Carvalho AR, Alves FA. The renewed concept of the Batista operation for ischemic cardiomyopathy: maximum ventricular reduction: O conceito renovado da operação de Batista na cardiomiopatia isquêmica: máxima redução ventricular. Rev bras cir cardiovasc. 2011;26(4):544-51.
322	Gong J, Shi DB, Li XX, Cai SJ, Guan ZQ, Xu Y. Short-term outcomes of laparoscopic total mesorectal excision compared to open surgery. World J Gastroenterol. 2012;18(48):7308-13.
323	Gonullu D, Koksoy FN, Ilgun S, Demiray O, Yucel O, Yucel T. Treatment of penetrating hepatic injuries: a retrospective analysis of 50 patients. Eur Surg Res. 2009;42(3):174-80.
324	Gonzales GF, Tapia VL, Fort AL, Betran AP. Pregnancy outcomes associated with Cesarean deliveries in Peruvian public health facilities. Int J Womens Health. 2013;5:637-45.
325	Gonzalez QH, Rodriguez-Zentner HA, Moreno-Berber JM, Vergara-Fernandez O, Tapia-Cid de Leon H, Jonguitud LA, et al. Laparoscopic versus open total mesorectal excision: a nonrandomized comparative prospective trial in a tertiary center in Mexico City. Am Surg. 2009;75(1):33-8.
326	Govender M, Madiba TE. Current management of large bowel injuries and factors influencing outcome. Injury. 2010;41(1):58-63.
327	Grinberg M, Jonke VM, Sampaio RO, Spina GS, Tarasoutchi F. Validation of a new surgical risk score for heart valve surgery: VMCP. Arq Bras Cardiol. 2009;92(4):320-5.
328	Guedes MAV, Pomerantzeff PMA, Brandão CMdA, Vieira MLC, Grinberg M, Stolf NAG. Mitral valve surgery using right anterolateral thoracotomy: is the aortic cannulation a safety procedure?: Cirurgia valvar mitral via toracotomia ântero-lateral direita: a canulação aórtica é segura? Rev bras cir cardiovasc. 2010;25(3):322-5.
329	Guler A, Sahin MA, Cingoz F, Ozal E, Demirkilic U, Arslan M. Can cardiac surgery be performed safely on patients with haematological malignancies. Cardiovasc J Afr. 2012;23(4):194-6.
330	Gunawansa N, Goonerathne T, Cassim R, Wijeyaratne M. Open repair of infra renal abdominal aortic aneurysms: a single center experience from the developing world. Ann Vasc Dis. 2011;4(4):313-8.
331	Gungorduk K, Yildirim G, Dugan N, Polat I, Sudolmus S, Ark C. Peripartum hysterectomy in Turkey: a case-control study. J Obstet Gynaecol. 2009;29(8):722-8.
332	Guo FH, Ma SL, Yang S, Dong YQ, Luo F, Wang ZM. Surgical strategy for gastric cancer patients with liver cirrhosis: A retrospective cohort study. International Journal of Surgery. 2014;12(8):810-4.
333	Guo Q, Wu Y. Surgical treatment of pancreatic islet cell tumor: report of 44 cases. Hepatogastroenterology. 2013;60(128):2099-102.
334	Gupta AR, Gupta R, Jadhav V, Sanghvi B, Shah HS, Parelkar SV. Minimal access surgery in children: an initial experience of 28 months. Afr J Paediatr Surg. 2009;6(2):93-7.
335	Gursoy S, Ucvet A, Ozturk AA, Erbaycu AE, Basok O, Yucel N. Seven years experience of bronchogenic cysts. Saudi Med J. 2009;30(2):238-42.
336	Gursoy S, Ucvet A, Tozum H, Erbaycu AE, Kul C, Basok O. Primary Intrathoracic Extrapulmonary Hydatid Cysts Analysis of 14 Patients with a Rare Clinical Entity. Tex Heart Inst J. 2009;36(3):230-3.
337	Gurtani FM, Fadaei B, Akbari M. Emergency peripartum hysterectomy in Isfahan; maternal mortality and morbidity rates among the women who underwent peripartum hysterectomy. Adv Biomed Res. 2013;2:20.
338	Gwely NN, Mowafy A, Khalaf S, Amer S, Hamza U, El-Saeed M. Management of stab wounds of the heart: analysis of 73 cases in 10 years. Thorac Cardiovasc Surg. 2010;58(4):210-4.
339	Haddad R, Fagundes WV. Aortoplastia redutora com contenção externa associada à troca valvar aórtica em pacientes de alto risco: Reduction aortoplasty with external wrapping associated with aortic valve replacement in high-risk patients. Rev bras cir cardiovasc. 2009;24(2):194-9.
340	Hadi A, Aman Z, Khan SA, Zafar H, Khan M, Afridi SK, et al. Surgical management of bile duct injuries following open or laparoscopic cholecystectomy. J Pak Med Assoc. 2013;63(8):1008-12.
341	Hadzi-Djokic J, Pejcic TP, Acimovic M. Vesico-vaginal fistula: report of 220 cases. Int Urol Nephrol. 2009;41(2):299-302.
342	Hagander L, Kabir M, Chowdhury MZ, Gunnarsdottir A, Habib MG, Banu T. Major Neonatal Surgery Under Local Anesthesia: A Cohort Study from Bangladesh. World J Surg. 2014.
343	Haider S, Shaheen R. To find out maternal and fetal outcome of cardiac disease in pregnancy. Pakistan Journal of Medical and Health Sciences. 2014;8(3):559-61.
344	Hajjar LA, Vincent JL, Galas F, Nakamura RE, Silva CMP, Santos MH, et al. Transfusion Requirements After Cardiac Surgery The TRACS Randomized Controlled Trial. Jama-Journal of the American Medical Association. 2010;304(14):1559-67.

Reference Number	Full reference
345	Halesha B, Raju VS, Chandrashekar K, Sahana KP. Emergency peripartum hysterectomy: A retrospective study at a tertiary care hospital in Karnataka. Indian Journal of Public Health Research and Development. 2013;4(3):103-6.
346	Hamid R, Baba AA, Shera AH, Wani SA, Altaf T, Kant MH. Late-presenting congenital diaphragmatic hernia. Afr J Paediatr Surg. 2014;11(2):119-23.
347	Hannan MJ, Hoque MM. Intestinal obstruction in children due to segmental enteritis: experience in Chittagong, Bangladesh. Pediatr Surg Int. 2012;28(3):277-80.
348	Hasbahceci M, Uludag M, Erol C, Ozdemir A. Laparoscopic cholecystectomy in a single, non-teaching hospital: an analysis of 1557 patients. J Laparoendosc Adv Surg Tech A. 2012;22(6):527-32.
349	Hashemi SMR, Golchin N, Nejad EA, Noormohamadi S. Timing of surgery for aneurysmal subarachnoid hemorrhage. Acta Med Iran. 2011;49(7):420-4.
350	Hashemzadeh S, Hashemzadeh K, Kakaei F, Aligholipour R, Ghabili K. Surgical treatment of postintubation tracheal stenosis: Iranian experience of effect of previous tracheostomy. Int J Gen Med. 2012;5:93-8.
351	Hassan N, Sirichand P, Zaheen Z, Shaikh F. Uterine rupture at LUMHS: A review of 85 cases. Journal of the Liaquat University of Medical and Health Sciences. 2009;8(2):165-8.
352	Haynes AB, Regenbogen SE, Weiser TG, Lipsitz SR, Dziekan G, Berry WR, et al. Surgical outcome measurement for a global patient population: validation of the Surgical Apgar Score in 8 countries. Surgery. 2011;149(4):519-24.
353	Hellar AMM, N. A. A. The Pattern and Surgical Management of Diabetic Foot at Muhimbili National Hospital, Dar-es-Salaam, Tanzania. East and Central African Journal of Surgery. 2011;16(1).
354	Hiep PN, Vu PA, Thien HH. Laparoscopic surgery in rectal cancer: a retrospective analysis. Int J Colorectal Dis. 2009;24(12):1465-9.
355	Hovnanian AL, Soeiro AdM, Serrano Júnior CV, Oliveira SAd, Jatene FB, Stolf NAG, et al. Surgical myocardial revascularization of patients with ischemic cardiomyopathy and severe left ventricular disfunction. Clinics. 2010;65(1):3-8.
356	Hu G, Jin B, Zheng H, Lai C, Ouyang C, Xia Y, et al. Analysis of 287 patients with aortic dissection: general characteristics, outcomes and risk factors in a single center. J Huazhong Univ Sci Technolog Med Sci. 2011;31(1):107-13.
357	Huang L, Li J, Yan JJ, Liu CF, Wu MC, Yan YQ. Prealbumin is predictive for postoperative liver insufficiency in patients undergoing liver resection. World J Gastroenterol. 2012;18(47):7021-5.
358	Huang ZQ, Xu LN, Yang T, Zhang WZ, Huang XQ, Cai SW, et al. Hepatic resection: an analysis of the impact of operative and perioperative factors on morbidity and mortality rates in 2008 consecutive hepatectomy cases. Chin Med J. 2009;122(19):2268-77.
359	Hussain D, Sarfraz K. Outcome of extra-mucosal small gut anastomosis in a peripheral hospital. Journal of Postgraduate Medical Institute. 2009;23(2):170-3.
360	Hyginus E, Eric NI, Lawrence I, Sylvester N. Morbidity and mortality following high order caesarean section in a developing country. J Pak Med Assoc. 2012;62(10):1016-9.
361	Ibrahim AG, Ali N, Aliyu S, Bakari AA. One-stage urethroplasty for strictures in maiduguri, north eastern Nigeria. ISRN Urol. 2012;2012:847870.
362	Iddriss A, Padayatchi N, Reddy D, Reddi A. Pulmonary resection for extensively drug resistant tuberculosis in Kwazulu-Natal, South Africa. Ann Thorac Surg. 2012;94(2):381-6.
363	Idowu OE, Falope LO, Idowu AT. Outcome of endoscopic third ventriculostomy and Chhabra shunt system in noncommunicating non-tumor childhood hydrocephalus. J Pediatr Neurosci. 2009;4(2):66-9.
364	Igberase GO, Ebeigbe PN, Andrew BO. HIGH CAESAREAN SECTION RATE: A TEN YEAR EXPERIENCE IN A TERTIARY HOSPITAL IN THE NIGER DELTA, NIGERIA. Niger J Clin Pract. 2009;12(3):294-7.
365	Iglezias JCR, Dallan LAO, Lourenção Júnior A, Celullare AL, Pereira R, Stolf NAG. Degree of risk related to procedures performed in conjunction with surgical myocardial revascularization in octogenarians. Clinics. 2009;64(5):387-92.
366	Igwegbe AO, Eleje GU, Udegbunam OI. Risk factors and perinatal outcome of uterine rupture in a low-resource setting. Niger Med J. 2013;54(6):415-9.
367	Ikeoka DT, Fernandes VA, Gebara O, Garcia JC, Barros e Silva PG, Rodrigues MJ, et al. Evaluation of the Society of Thoracic Surgeons score system for isolated coronary bypass graft surgery in a Brazilian population. Rev Bras Cir Cardiovasc. 2014;29(1):51-8.
368	Ilori IUI, A.M.; Eyo, C.S. Factors associated with mortality in neonatal surgical emergencies in a developing tertiary hospital in Nigeria. Open J Pediatri. 2013;3:231-5.
369	Islam J, Clarke DL, Thomson SR. Lessons from emergency laparotomy for abdominal tuberculosis in the HIV/AIDS era. S Afr J Surg. 2014;52(1):10-2.
370	Islam MT, Yoshimura Y. Rate of cesarean delivery at hospitals providing emergency obstetric care in Bangladesh. International Journal of Gynecology and Obstetrics. 2015;128(1):40-3.
371	Iyem H. Evaluation of the reliability of the EuroSCORE risk analysis prediction in high-risk older patients undergoing CABG. Cardiovasc J Afr. 2009;20(6):340-3.
372	Jaha L, Andreevska T, Rudari H, Ademi B, Ismaili-Jaha V. A decade of civilian vascular trauma in Kosovo. World J Emerg Surg. 2012;7(1):24.
373	Jain BK, Garg PK, Kumar A, Mishra K, Mohanty D, Agrawal V. Colonic perforation with peritonitis in amoebiasis: a tropical disease with high mortality. Trop Gastroenterol. 2013;34(2):83-6.
374	Jaipuria J, Sagar S, Singhal M, Bagdia A, Gupta A, Kumar S, et al. Paediatric extremity vascular injuries - Experience from a large urban trauma centre in India. Injury-International Journal of the Care of the Injured. 2014;45(1):176-82.
375	Jakab F, Teknos D, Baranyai Z, Mersich T. Transverse hepatectomy: a 14-years experience. Hepatogastroenterology. 2012;59(115):844-6.
376	Jan WA, Rehman ZU, Khan SM, Ali G, Qayyum A, Mumtaz N. Outcome of open versus laproscopic appendicectomy in department of surgery, lady reading hospital, Peshawar. Journal of Postgraduate Medical Institute. 2011;25(3):245-51.
377	Janati M, Bolandparvaz S, Salaminia S, Johari HG, Sabet B, Kojuri J. Outcome of penetrating cardiac injuries in southern Iran, Shiraz. Chin J Traumatol. 2013;16(2):89-93.
378	Janjua AM, Saleem K, Khan I, Rashid A, Khan AA, Hussain A. Double flap patch closure of VSD with elevated pulmonary vascular resistance: An experience at AFIC/NIHD. Journal of the College of Physicians and Surgeons Pakistan. 2011;21(4):197-201.
379	Jat N, Ghauri AQ, Khokhar PB, Memon IA, Iftikhar R. Laparoscopic cholecystectomy - 5 years experience at SOM Fauji foundation hospital Karachi. Medical Channel. 2011;17(2):18-20.
380	Javaid SY, T.; Rafique, S.; Malik, S. Postpartum and emergency caesarean hysterectomy. Pak J Med Health Sci 2011;5:239-42.
381	Jehangir S, John J, Rajkumar S, Mani B, Srinivasan R, Kang G. Intussusception in southern India: Comparison of retrospective analysis and active surveillance. Vaccine. 2014;32:A99-A103.
382	Ji Q, Mei Y, Wang X, Feng J, Cai J, Ding W. Risk factors for pulmonary complications following cardiac surgery with cardiopulmonary bypass. Int J Med Sci. 2013;10(11):1578-83.
383	Jiang T, Jia G, Ma Z, Luo S, Zhang Y. The diagnosis and treatment of subependymal giant cell astrocytoma combined with tuberous sclerosis. Childs Nerv Syst. 2011;27(1):55-62.
384	Joaquim AF, Ghizoni E, Anderle DV, Oliveira Ed, Tedeschi H. Axis instrumentation: surgical results: Instrumentação do áxis: resultados cirúrgicos. Arq neuropsiquiatr. 2012;70(11):857-63.
385	Kacila M, K KT, Granov N, Omerbasic E, Straus S. Assessment of the Initial and Modified Parsonnet score in mortality prediction of the patients operated in the Sarajevo Heart center. Bosn J Basic Med Sci. 2010;10(2):165-8.
386	Kadowa I. Ruptured uterus in rural Uganda: prevalence, predisposing factors and outcomes. Singapore Med J. 2010;51(1):35-8.
387	Kakar BK, Khan S, Babar H. Surgical experience of chronic constrictive pericarditis at Quetta, Pakistan. Pakistan Journal of Medical and Health Sciences. 2012;6(1):63-5.
388	Kamani F, Moghimi M, Marashi SA, Peyrovi H, Sheikhvatan M. Perforated peptic ulcer disease: mid-term outcome among Iranian population. Turk J Gastroenterol. 2010;21(2):125-8.
389	Kamilya G, Seal SL, Mukherji J, Bhattacharyya SK, Hazra A. Maternal mortality and cesarean delivery: an analytical observational study. J Obstet Gynaecol Res. 2010;36(2):248-53.

Reference Number	Full reference
390	Kandakure PR, Dharmapuram AK, Ramadoss N, Babu V, Rao IM, Murthy KS. Sternotomy approach for modified Blalock-Taussig shunt: is it a safe option? Asian Cardiovasc Thorac Ann. 2010;18(4):368-72.
391	Kandasamy T, Merialdi M, Guidotti RJ, Betran AP, Harris-Requejo J, Hakimi F, et al. Cesarean delivery surveillance system at a maternity hospital in Kabul, Afghanistan. Int J Gynaecol Obstet. 2009;104(1):14-7.
392	Kara M, Toz E, Yilmaz E, Oge T, Avci I, Eminli I, et al. Analysis of uterine rupture cases in Agri: A five-year experience. Clin Exp Obstet Gynecol. 2010;37(3):221-3.
393	Kara M. Emergency peripartum hysterectomy cases in Agri: a 6-year review. Clin Exp Obstet Gynecol. 2012;39(2):202-4.
394	Karapandzic VM, Matic MD, Pesko PM, Rankovic VI, Milicic BR. Risk assessment in coronary patients undergoing abdominal nonvascular surgery. Central European Journal of Medicine. 2009;4(4):459-66.
395	Karayalcin R, Ozcan S, Ozyer S, Mollamahmutoglu L, Danisman N. Emergency peripartum hysterectomy. Arch Gynecol Obstet. 2011;283(4):723-7.
396	Kargar S, Mirshamsi MH, Zare M, Arefanian S, Shadman Yazdi E, Aref A. Laparoscopic versus open appendectomy; which method to choose? A prospective randomized comparison. Acta Med Iran. 2011;49(6):352-6.
397	Karkee R, Lee AH, Khanal V, Pokharel PK, Binns CW. Obstetric complications and cesarean delivery in Nepal. International Journal of Gynecology and Obstetrics. 2014;125(1):33-6.
398	Karpelowsky JS, Leva E, Kelley B, Numanoglu A, Rode H, Millar AJ. Outcomes of human immunodeficiency virus-infected and -exposed children undergoing surgery--a prospective study. J Pediatr Surg. 2009;44(4):681-7.
399	Karpelowsky JS, Millar AJ, van der Graaf N, van Bogerijen G, Zar HJ. Comparison of in-hospital morbidity and mortality in HIV-infected and uninfected children after surgery. Pediatr Surg Int. 2012;28(10):1007-14.
400	Karpelowsky JS, Zar HJ, van Bogerijen G, van der Graaf N, Millar AJ. Predictors of postoperative complications in HIV-infected children undergoing surgery. J Pediatr Surg. 2011;46(4):674-8.
401	Kassi AB, Lebeau R, Yenon KS, Katche E, Diane B, Kouassi JC. Morbidity and mortality of Hartmann's procedure for sigmoid volvulus at the University Hospital of Cocody, Abidjan. West Afr J Med. 2011;30(3):169-72.
402	Kaushish LCR, Unni BMK, Luthra MGM. Beating heart versus conventional coronary bypass surgery: Our experience. Medical Journal Armed Forces India. 2010;66(4):357-61.
403	Kaya B, Sana B, Eris C, Kutanis R. Immediate appendectomy for appendiceal mass. Ulus Travma Acil Cerrahi Derg. 2012;18(1):71-4.
404	Kazim SF, Pal KM. Appendicitis in pregnancy: experience of thirty-eight patients diagnosed and managed at a tertiary care hospital in Karachi. Int J Surg. 2009;7(4):365-7.
405	Kendig CE, Samuel JC, Varela C, Msiska N, Kiser MM, McLean SE, et al. Pediatric surgical care in Lilongwe, Malawi: outcomes and opportunities for improvement. J Trop Pediatr. 2014;60(5):352-7.
406	Kertai MD, Tiszai-Szucs T, Varga KS, Hermann C, Acsady G, Gal J. Intraoperative use of packed red blood cell transfusion and mortality in patients undergoing abdominal or thoracoabdominal aortic aneurysm surgery. J Cardiovasc Surg (Torino). 2009;50(4):501-8.
407	Khalaf I, Salih E, El-Mallah E, Farghal S, Abdel-Raouf A. The outcome of open renal stone surgery calls for limitation of its use: A single institution experience. African Journal of Urology. 2013;19(2):58-65.
408	Khaleghnejad Tabari A, Mirshemirani A, Rouzrokh M, Mohajerzadeh L, Khaleghnejad Tabari N, Ghaffari P. Acute mediastinitis in children: A nine-year experience. Tanaffos. 2013;12(2):48-52.
409	Khalil J, Muqim R, Rafique M, Khan M. Laparoscopic versus open appendectomy: a comparison of primary outcome measures. Saudi J Gastroenterol. 2011;17(4):236-40.
410	Khamechian T, Alizargar J, Farzanegan M. Pattern of splenectomy indications in kashan shahid-beheshti hospital: a 5-year study. Arch Trauma Res. 2013;1(4):180-3.
411	Khan A, Ghani T, Rahim A, Rahman MM. Changing trends in incidence and indications of caesarean section. Mymensingh Med J. 2014;23(1):52-5.
412	Khan B, Khan B, Sultana R, Bashir R, Deeba F. A ten year review of emergency peripartum hysterectomy in a tertiary care hospital. J Ayub Med Coll Abbottabad. 2012;24(1):14-7.
413	Khan IM, Aurangzeb M, Mujeeb ur R, Tayyab M. Palliative surgery for pancreatic carcinoma. Journal of the College of Physicians and Surgeons Pakistan. 2010;20(11):719-22.
414	Khan K, Khan MY, Mamoon M, Manan F, Uzair M, Fayaz M, et al. Modified posterior sagittal ano-rectoplasty: A new approach for the management of ano-rectal malformations in children. JPMI - Journal of Postgraduate Medical Institute. 2012;26(2):183-90.
415	Khan KI, Mahmood S, Akmal M, Waqas A. Comparison of rate of surgical wound infection, length of hospital stay and patient convenience in complicated appendicitis between primary closure and delayed primary closure. J Pak Med Assoc. 2012;62(6):596-8.
416	Khan MA, Dar AM, Kawoosa NU, Ahangar AG, Lone GN, Bashir G, et al. Clinical profile and surgical outcome for pulmonary aspergilloma: nine year retrospective observational study in a tertiary care hospital. Int J Surg. 2011;9(3):267-71.
417	Khan MA, Godil SS, Tabani H, Panju S, Enam SA. Clinical review of pediatric pilocytic astrocytomas treated at a tertiary care hospital in Pakistan. Surg Neurol Int. 2012;3(1).
418	Khan MB, Kumar R, Irfan FB, Irfan AB, Bari ME. Civilian craniocerebral gunshot injuries in a developing country: presentation, injury characteristics, prognostic indicators, and complications. World Neurosurg. 2014;82(1-2):14-9.
419	Khan MR, Bari H, Raza SA. Early postoperative outcome after curative colorectal cancer surgery. Singapore Med J. 2011;52(3):195-200.
420	Khan MR, Bari H, Zafar SN, Raza SA. Impact of age on outcome after colorectal cancer surgery in the elderly - a developing country perspective. BMC Surg. 2011;11:17.
421	Khan MR, Kassi M, Janjua SA. Abdominal wall hernia repair in cirrhotic patients: outcomes seen at a tertiary care hospital in a developing country. Trop Doct. 2010;40(1):5-8.
422	Khan MS, Sanki PK, Hossain MZ, Charles A, Bhattacharya S, Sarkar UN. Cardiac myxoma: A surgical experience of 38 patients over 9 years, at SSKM hospital Kolkata, India. South Asian J Cancer. 2013;2(2):83-6.
423	Khan MZ, Perveen S, Ansari JA, Sami SA, Furnaz S, Fatimi SH. Outcome and factors associated with hospital mortality in patients with impaired left ventricular function undergoing coronary artery bypass grafting: Where do we stand? Pakistan Journal of Medical Sciences. 2009;25(4):526-32.
424	Khan TR, Rawat J, Maletha M, Singh S, Rashid KA, Wakhlu A, et al. Traumatic diaphragmatic injuries in children: do they really mark the severity of injury? Our experience. Pediatr Surg Int. 2009;25(7):595-9.
425	Khan Z, Sharafat S, Ali M. Early complications of simple anterior cervical discectomy. JPMI - Journal of Postgraduate Medical Institute. 2012;26(4):444-8.
426	Khanna AK, Tiwary SK, Kumar P, Khanna R, Khanna A. A case series describing 118 patients with lower limb necrotizing fasciitis. Int J Low Extrem Wounds. 2009;8(2):112-6.
427	Khanum F, Sadaf R, Meher un N, Zahid M. Emergency peripartum hysterectomy in a tertiary care hospital. Journal of Medical Sciences (Peshawar). 2013;21(2):58-61.
428	Khemakhem R, Haggui B, Rahay H, Nouira F, Charieg A, Ghorbel S, et al. Congenital diaphragmatic hernia in neonate: a retrospective study about 28 observations. Afr J Paediatr Surg. 2012;9(3):217-22.
429	Khorram-Manesh A, Pourseidi B. Management of traumatic liver injuries without a valid trauma system. Prehosp Disaster Med. 2009;24(4):349-55.
430	Khumjui C, Doung-Ngern P, Sermgew T, Smitsuwan P, Jiraphongsa C. Incidence of intussusception among children 0-5 years of age in Thailand, 2001-2006. Vaccine. 2009;27:F116-F9.
431	Kiboi JG, Kitunguu PK, Angwenyi PO, Sagina LS. Outcome after acute traumatic subdural haematoma in Kenya: A single-centre experience. African Journal of Neurological Sciences. 2010;29(1).
432	Kim YM, Tappis H, Zainullah P, Ansari N, Evans C, Bartlett L, et al. Quality of caesarean delivery services and documentation in first-line referral facilities in Afghanistan: a chart review. BMC Pregnancy Childbirth. 2012;12:14.

Reference Number	Full reference
433	Kishore GS, Gupta V, Doley RP, Kudari A, Kalra N, Yadav TD, et al. Traumatic diaphragmatic hernia: tertiary centre experience. Hernia. 2010;14(2):159-64.
434	Kitara DLK, I.; Mugisa, B. D.; Obol, J. H. The Postoperative Complications Prediction in Mulago Hospital using POSSUM Scoring System. East and Central African Journal of Surgery. 2010;15(2).
435	Kithikii KPG, K. J. Risk Factors Related to Hospital Mortality in Kenyan Patients with Traumatic Intracranial Haematomas. East and Central African Journal of Surgery. 2011;16(1).
436	Koh KH, Tan C, Hii L, Ong TK, Jong YH. Outcome of coronary artery bypass grafting in end stage renal disease patients. Med J Malaysia. 2012;67(2):173-6.
437	Kong VY, Bulajic B, Allorto NL, Handley J, Clarke DL. Acute appendicitis in a developing country. World J Surg. 2012;36(9):2068-73.
438	Kong VY, van de Linde S, Aldous C, Handley JJ, Clarke DL. Quantifying the disparity in outcome between urban and rural patients with acute appendicitis in South Africa. S Afr Med J. 2013;103(10):742-5.
439	Korejo R, Nasir A, Yasmin H, Bhutta S. Emergency obstetric hysterectomy. J Pak Med Assoc. 2012;62(12):1322-5.
440	Koszta G, Kacska Z, Szatmari K, Szerafin T, Fulesdi B. Lower whole blood selenium level is associated with higher operative risk and mortality following cardiac surgery. J Anesth. 2012;26(6):812-21.
441	Koszta G, Sira G, Szatmari K, Farkas E, Szerafin T, Fulesdi B. Performance of EuroSCORE II in Hungary: A Single-centre Validation Study. Heart Lung Circ. 2014;23(11):1041-50.
442	Kotb S, Elsheemy MS, Morsi HA, Zakaria T, Salah M, Eissa MA. Renal recoverability in infants with obstructive calcular anuria: Is it better than in older children? J Pediatr Urol. 2013;9(6 PART B):1178-82.
443	Kothari KC, Nair CK, George PS, Patel MH, Gatti RC, Gurjar GC. Comparison of esophagectomy with and without thoracotomy in a low-resource tertiary care center in a developing country. Dis Esophagus. 2011;24(8):583-9.
444	Kotze PG, Abou-Rejaile VR, Barcelos IFd, Martins JF, Miranda EF, Rocha JG, et al. Complications after intestinal resection in Crohn's disease: laparoscopic versus conventional approach: Complicacoes apos ressecoes intestinais na doenca de Crohn: ha diferencas entre as vias convencional e videolaparoscopica? J coloproctol (Rio J, Impr). 2013;33(3):139-44.
445	Kritayakirana K, Sriussadaporn S, Pak-Art R, Prichayudh S, Samorn P, Sriussadaporn S. Cardiac trauma: has survival improved? A university hospital experience in Bangkok, Thailand. J Med Assoc Thai. 2013;96(2):196-202.
446	Kucukarslan N, Tatar T, Uzun M, Yavuz I, Ozal E, Tatar H. Coronary artery bypass surgery in patients with malignancy: a single-center study with comparison to patients without malignancy. J Card Surg. 2009;24(2):151-5.
447	Kwari DY, Chinda JY, Olasoji HO, Adeosun OO. Cleft lip and palate surgery in children: anaesthetic considerations. Afr J Paediatr Surg. 2010;7(3):174-7.
448	Kwari YD, Bello MR, Eni UE. Pattern of perioperative cardiac arrests at University of Maiduguri Teaching Hospital. Niger J Med. 2010;19(2):173-6.
449	Lacerda CF, Bertulucci PA, Oliveira ATTd. Totally laparoscopic liver resection: new brazilian experience: Ressecção hepática totalmente laparoscópica: nova experiência brasileira. ABCD arq bras cir dig. 2014;27(3):191-5.
450	Laiq N, Khan RA, Mailk A, Ahmad H. Intravenous magnesium prevents atrial fibrillation after valvular heart surgery. Journal of Medical Sciences (Peshawar). 2013;21(2):77-80.
451	Lakhey PJ, Bhandari RS, Ghimire B, Khakurel M. Perioperative outcomes of pancreaticoduodenectomy: Nepalese experience. World J Surg. 2010;34(8):1916-21.
452	Lal P, Kumar R, Leekha N, Chander J, Kar P, Ramteke VK. Laparoscopic Nissen fundoplication is an excellent modality for GERD: early experience from a tertiary care hospital in India. J Laparoendosc Adv Surg Tech A. 2010;20(5):441-6.
453	Landry E, Pett C, Fiorentino R, Ruminjo J, Mattison C. Assessing the quality of record keeping for cesarean deliveries: results from a multicenter retrospective record review in five low-income countries. BMC Pregnancy Childbirth. 2014;14:139.
454	Laohawiriyakamol S, Sangkhathat S, Chiengkriwate P, Patrapinyokul S. Surgery in management of snake envenomation in children. World J Pediatr. 2011;7(4):361-4.
455	Lashkarizadeh MR, Ashrafganguie M, Ashrafganguie M. Surgical management of femoral artery pseudoaneurysms secondary to drug abuse. Journal of the College of Physicians and Surgeons Pakistan. 2011;21(11):672-5.
456	Latipov R, Khudoyorov R, Flem E. Childhood intussusception in Uzbekistan: Analysis of retrospective surveillance data. BMC Pediatr. 2011;11.
457	Leake PA, Hamilton-Johnson TN, Harry M, Gordon-Strachan GM, Plummer JM, Newnham MS. Open abdominal aortic aneurysm repair in the era of endovascular repair: Reparación abierta de la aorta abdominal en la era de la reparación endovascular^ies. West Indian med j. 2011;60(6):636-40.
458	Leake PA, Plummer JM, Rhoden A, Frankson MA, Gordon-Strachan G, Powell LP, et al. Colorectal anastomotic leakage at the university hospital of the west indies: an analysis of risk factors. West Indian Med J. 2013;62(9):711-5.
459	Lei Z, Zheng Z, Li J, Li Q. The diagnosis and treatment for hilar cholangiocarcinoma: A report of 136 cases. Chinese-German Journal of Clinical Oncology. 2012;11(1):11-4.
460	Lenzi AW, Miyague NI, Ferreira WS, Sallum FS. Mortalidade hospitalar na cirurgia de reconstrução da via de saída do ventrículo direito com homeonxerto pulmonar: Hospital mortality in surgery for reconstruction outflow right ventricle with pulmonary homograft. Rev bras cir cardiovasc. 2010;25(1):25-31.
461	Leon-Wyss J, Lo Rito M, Barnoya J, Castaneda AR. Persistent institutional difficulties in surgery for transposition of the great arteries in guatemala: analysis with the aristotle basic and comprehensive scores. World J Pediatr Congenit Heart Surg. 2011;2(3):346-50.
462	Leon-Wyss JR, Veshti A, Veras O, Gaitan GA, O'Connell M, Mack RA, et al. Pediatric cardiac surgery: a challenge and outcome analysis of the Guatemala effort. Semin Thorac Cardiovasc Surg Pediatr Card Surg Annu. 2009:8-11.
463	Letchumanan VP, Lim KF, Mohamad AB. Diagnosis and management of ruptured hepatoma: single center experience over 10 years. Med J Malaysia. 2013;68(5):405-9.
464	Lewis A, Kaufman MR, Wolter CE, Phillips SE, Maggi D, Condry L, et al. Genitourinary Fistula Experience in Sierra Leone: Review of 505 Cases. J Urol. 2009;181(4):1725-31.
465	Li BZ, Guo QQ, Yang HJ, Guan JY, Liv ZQ, Li SM, et al. Left Thoracoabdominal Approach in Surgical Treatment of Adenocarcinoma of the Esophagogastric Junction in the Northern Henan Province of China. Dig Surg. 2011;28(1):2-8.
466	Li C, Yang WH, Zhou J, Wu Y, Li YS, Wen SH, et al. Risk factors for predicting postoperative complications after open infrarenal abdominal aortic aneurysm repair: results from a single vascular center in China. J Clin Anesth. 2013;25(5):371-8.
467	Li J, Mukherjee R, Lan Z, Liu Y, He M. Microneurosurgical management of anterior choroidal artery aneurysms: a 16-year institutional experience of 102 patients. Neurol Res. 2012;34(3):272-80.
468	Li J, Wang X, Feng X, Gu Y, Tang R. Comparison of open and laparoscopic preperitoneal repair of groin hernia. Surg Endosc. 2013;27(12):4702-10.
469	Li M, Ni J, Li Y, Fang X, Gu S, Zhang Z, et al. Single-staged anterior and posterior spinal fusion: A safe and effective alternative for severe and rigid adolescent idiopathic scoliosis in China. J Paediatr Child Health. 2009;45(5):246-53.
470	Li Q, Yang CH, Xu JG, Chen J, You C. Cross-sectional study of craniocerebral trauma in a tertiary hospital after 2008 sichuan earthquake: A brief report of 242 cases and experiences from West China Hospital. Journal of Trauma - Injury, Infection and Critical Care. 2011;70(6):E108-E12.
471	Li Y, Zheng Z, Hu S. The Chinese coronary artery bypass grafting registry study: Analysis of the national multicentre database of 9248 patients. Heart. 2009;95(14):1140-4.
472	Li Z, Li B, Fan X, Su J, Zhang J, He Y, et al. Surgical treatment of interrupted aortic arch associated with ventricular septal defect and patent ductus arteriosus in patients over one year of age. Chin Med J. 2014;127(9):1684-90.
473	Liang CY, Wen HS, Guo YQ, Shi B, Tian YC, Song ZY, et al. Severe intraoperative complications during VATS Lobectomy compared with thoracotomy lobectomy for early stage non-small cell lung cancer. J Thorac Dis. 2013;5(4):513-7.
474	Liang X, Hou S, Liu H, Li Y, Jiang B, Bai W, et al. Effectiveness and safety of laparoscopic resection versus open surgery in patients with rectal cancer: A randomized, controlled trial from China. Journal of Laparoendoscopic and Advanced Surgical Techniques. 2011;21(5):381-5.

Reference Number	Full reference
475	Limpastan K, Norasetthada T, Watcharasaksilp W, Vaniyapong T. Factors influencing the outcome of decompressive craniectomy used in the treatment of severe traumatic brain injury. J Med Assoc Thai. 2013;96(6):678-82.
476	Lin JX, Huang CM, Zheng CH, Li P, Xie JW, Wang JB, et al. Laparoscopy-assisted gastrectomy with D2 lymph node dissection for advanced gastric cancer without serosa invasion: a matched cohort study from South China. World J Surg Oncol. 2013;11:4.
477	Lisboa LAF, Moreira LFP, Mejia OV, Dallan LAO, Pomerantzeff PMA, Costa R, et al. Evolution of cardiovascular surgery at the Instituto do Coracao: Analysis of 71,305 surgeries. Arq Bras Cardiol. 2010;94(2):164-71+2-8+74-81.
478	Litorp H, Kidanto HL, Ronullonullst M, Abeid M, Nystronullm L, Essenulln B. Maternal near-miss and death and their association with caesarean section complications: A cross-sectional study at a university hospital and a regional hospital in Tanzania. BMC Pregnancy Childbirth. 2014;14(1).
479	Liu H, Zen Y, Li J, Wang X, Li H, Xu J, et al. Optimal treatment determination on the basis of haematoma volume and intra-cerebral haemorrhage score in patients with hypertensive putaminal haemorrhages: a retrospective analysis of 310 patients. BMC Neurol. 2014;14:141.
480	Liu SX, Xu XD, Ding XY, Liu GZ, Zhao ZZ, Zhao XX, et al. Comparison of immediate results and mid-term follow-up of surgical and percutaneous closure of ruptured sinus of Valsalva aneurysm. J Cardiol. 2014;63(3-4):239-43.
481	Liu YL, Hu SS, Shen XD, Li SJ, Wang X, Yan J, et al. Safety and efficacy of arterial switch operation in previously inoperable patients. J Card Surg. 2010;25(4):400-5.
482	Lobão CAF, Nogueira J, Souto AADd, Oliveira JAd. Cerebral biopsy: comparison between frame-based stereotaxy and neuronavigation in an oncology center: Biópsia cerebral: comparação entre estereotaxia com arco e neuronavegação em um centro de oncologia. Arq neuropsiquiatr. 2009;67(3b):876-81.
483	Locali RF, Matsuoka PK, Cherbo T, Gabriel EA, Buffolo E. Tumores renais e adrenais com invasão cardíaca: resultados cirúrgicos imediatos em 14 pacientes: Renal and adrenal tumors with cardiac invasion: immediate surgical results in 14 patients: Tumores renales y adrenales con invasión cardiaca: resultados quirúrgicos inmediatos en 14 pacientes^ies. Arq bras cardiol. 2009;92(3):178-82.
484	Lofvenmark I, Norrbrink C, Nilsson-Wikmar L, Hultling C, Chakandinakira S, Hasselberg M. Traumatic spinal cord injury in Botswana: characteristics, aetiology and mortality. Spinal Cord. 2014.
485	Lohsiriwat V, Anubhonganant W, Prapasrivorakul S, Iramaneerat C, Riansuwan W, Boonnuch W, et al. Outcomes of local excision for early rectal cancer: a 6-year experience from the largest university hospital in Thailand. Asian Pac J Cancer Prev. 2013;14(9):5141-4.
486	Lohsiriwat V, Pongsanguansuk W, Lertakyamanee N, Lohsiriwat D. Impact of metabolic syndrome on the short-term outcomes of colorectal cancer surgery. Dis Colon Rectum. 2010;53(2):186-91.
487	Lohsiriwat V. Enhanced recovery after surgery vs conventional care in emergency colorectal surgery. World J Gastroenterol. 2014;20(38):13950-5.
488	Lone YA, Dar AM, Sharma ML, Robbani I, Sarmast AH, Mushtaq E, et al. Outcome of the surgical treatment of bullous lung disease: A prospective study. Tanaffos. 2012;11(2):27-33.
489	Long TC, Bac NH, Thuan ND, Dat le T, Viet DQ, Chuong le CH. Laparoscopic liver resection: 5-year experience at a single center. Surg Endosc. 2014;28(3):796-802.
490	Lopes-Junior AG, Belebecha V, Jacob CE. Hepatectomy: a critical analysis on expansion of the indications: Hepatectomia: uma analise critica da ampliacao das indicacoes. ABCD arq bras cir dig. 2014;27(1):47-52.
491	Lopez-Basave HN, Morales-Vasquez F, Herrera-Gomez A, Rosciano AP, Meneses-Garcia A, Ruiz-Molina JM. Pelvic exenteration for colorectal cancer: oncologic outcome in 59 patients at a single institution. Cancer Manag Res. 2012;4:351-6.
492	Lotfy M, El Said A, Sakr S. Decompressive craniotomy after traumatic brain injury: Post operative clinical outcome. Egyptian Journal of Neurology, Psychiatry and Neurosurgery. 2010;47(2):255-9.
493	Lourenço IS, Franco AM, Bassetto S, Rodrigues AJ. Pressure support-ventilation versus spontaneous breathing with "T-Tube" for interrupting the ventilation after cardiac operations: Pressão de suporte ventilatório versus respiração espontânea em "Tubo-T" para a interrupção da ventilação após as operações cardíacas. Rev bras cir cardiovasc. 2013;28(4):455-61.
494	Loveland JA, Krog F, Beale P. A review of paediatric liver resections in Johannesburg: experiences and preferred technique. S Afr Med J. 2012;102(11 Pt 2):881-3.
495	Lucumay EM, Gilyoma JM, Rambau PF, Chalya PL. Paediatric neck masses at a University teaching hospital in northwestern Tanzania: a prospective analysis of 148 cases. BMC Res Notes. 2014;7:772.
496	Lukong CS, Jabo BA, Mfuh AY. Colostomy in neonates under local anaesthesia: indications, technique and outcome. Afr J Paediatr Surg. 2012;9(2):176-80.
497	Lumbiganon P, Laopaiboon M, Gulmezoglu AM, Souza JP, Taneepanichskul S, Ruyan P, et al. Method of delivery and pregnancy outcomes in Asia: the WHO global survey on maternal and perinatal health 2007-08. Lancet. 2010;375(9713):490-9.
498	Luo GH, Ma WG, Sun HS, Pan SW, Huang ZX, Wang HY, et al. Surgical treatment for primary mitral valve tumor: a 25-year single-center experience. Cardiology. 2011;119(2):81-7.
499	Luqman Z, Ansari J, Siddiqui FJ, Sami SA. Is urgent coronary artery bypass grafting a safe option in octogenarians? A developing country perspective. Interact Cardiovasc Thorac Surg. 2009;9(3):441-5.
500	Lynch JC, Corrêa J, Pereira C. Diagnosis of, surgical technique for and treatment results from medullary lipomas associated with spinal dysraphism: experience with 38 patients: Diagnóstico, técnica cirúrgica e resultados nos lipomas medulares associados ao disrafismo vertebral: experiência com 38 pacientes. Arq neuropsiquiatr. 2011;69(4):676-81.
501	Lynch JC, Schiavini H, Bomfim C, Fonseca JF, Pereira C. Microsurgical ressection for parasagittal meningiomas with preservation of the parasagittal sinus and excelent neurovascular control: Ressecção microcirúrgica dos meningiomas parasagitais com excelente controle vascular e preservação do seio longitudinal superior. Arq neuropsiquiatr. 2013;71(5):301-6.
502	Ma DY, Liu LZ, Yao H, Hu YJ, Ji T, Liu XC, et al. A retrospective study in management of carotid body tumour. Br J Oral Maxillofac Surg. 2009;47(6):461-5.
503	Ma LK, Liu N, Bian XM, Teng LR, Qi H, Gong XM, et al. Pregnancy outcomes of repeat cesarean section in Peking Union Medical College Hospital. Chin Med Sci J. 2009;24(3):147-50.
504	Ma LL, Bi H, Hou XF, Huang Y, Wang GL, Zhao L, et al. Laparoendoscopic single-site radical cystectomy and urinary diversion: Initial experience in China using a homemade single-port device. J Endourol. 2012;26(4):355-9.
505	Maaloe N, Bygbjerg IC, Onesmo R, Secher NJ, Sorensen BL. Disclosing doubtful indications for emergency cesarean sections in rural hospitals in Tanzania: a retrospective criterion-based audit. Acta Obstet Gynecol Scand. 2012;91(9):1069-76.
506	Mabula JB, Chalya PL, McHembe MD, Kihunrwa A, Massinde A, Chandika AB, et al. Bowel perforation secondary to illegally induced abortion: a tertiary hospital experience in Tanzania. World J Emerg Surg. 2012;7(1):29.
507	Mabula JB, Chalya PL. Surgical management of inguinal hernias at Bugando Medical Centre in northwestern Tanzania: our experiences in a resource-limited setting. BMC Res Notes. 2012;5:585.
508	Mabula JB, Gilyoma JM, McHembe MD, Jaka H, Kamugisha E, Kidenya B, et al. Predictors of outcome among patients with obstructive jaundice at Bugando medical centre in north-western Tanzania. Tanzania Journal of Health Research. 2013;15(4).
509	Mabula JB, Kayange NM, Manyama M, Chandika AB, Rambau PF, Chalya PL. Hirschsprung's disease in children: a five year experience at a university teaching hospital in northwestern Tanzania. BMC Res Notes. 2014;7:410.
510	Mabula JB, McHembe MD, Koy M, Chalya PL, Massaga F, Rambau PF, et al. Gastric cancer at a university teaching hospital in northwestern Tanzania: a retrospective review of 232 cases. World J Surg Oncol. 2012;10:257.
511	Machado MAC, Surjan RCT, Goldman SM, Ardengh JC, Makdissi FF. LAPAROSCOPIC PANCREATIC RESECTION. FROM ENUCLEATION TO PANCREATODUODENECTOMY. 11-YEAR EXPERIENCE: Pancreatectomia laparoscopica. Da enucleacao a duodenopancreatectomia. 11 anos de experiencia. Arq gastroenterol. 2013;50(3):214-8.
512	Machado MdN, Miranda RC, Takakura IT, Palmegiani E, Santos CAd, Oliveira MA, et al. Lesão renal aguda após revascularização do miocárdio com circulação extracorpórea: Lesión renal aguda post-revascularización del miocardio con circulación extracorpórea Acute kidney injury after on-pump coronary artery bypass graft surgery. Arq bras cardiol. 2009;93(3):247-52.

Reference Number	Full reference
513	Machado MN, Nakazone MA, Murad-Júnior JA, Maia LN. Surgical treatment for infective endocarditis and hospital mortality in a Brazilian single-center: Tratamento cirúrgico para endocardite infecciosa e mortalidade hospitalar em centro único brasileiro. Rev bras cir cardiovasc. 2013;28(1):29-35.
514	Madureira FAV, Madureira FAV, Loss AB, Madureira D. Qualidade de vida após a cardiomiotomia à Heller-Dor: Quality of life after Heller-Dor's cardiomiotomy. Rev Col Bras Cir. 2009;36(3):193-8.
515	Mahmoudi S, Elikaee S, Keshavarz H, Pourakbari B, Mamishi S. Pediatric hydatidosis in Iranian referral pediatrics center. Iranian Journal of Parasitology. 2012;7(2):87-91.
516	Malagon Reyes RM, Reyes Mendoza LE, Angeles Vazquez MJ, Mendieta Zeron H. Experience of the MALA bag in the open abdomen management in an Obstetrical Intensive Care Unit. Acta Med Port. 2013;26(6):699-704.
517	Malekpour-Afshar R, Karamoozian S, Shafei H. Post traumatic meningitis in neurosurgery department. Am J Infect Dis. 2009;5(1):21-5.
518	Malhotra A, Pawar SR, Srivastava A, Yadav BS, Kaushal R, Sharma P, et al. Clinical and hemodynamic study of tilting disc heart valve: Single-center study. Asian Cardiovascular and Thoracic Annals. 2014;22(5):519-25.
519	Malik A, Shah SMA, Khan MA, Amin M, Waseem N, Noor A, et al. Atrial myxoma: An experience in a tertiary care center. Journal of Postgraduate Medical Institute. 2011;25(2):147-51.
520	Malik AA, Bari SU, Amin R, Jan M. Surgical management of complicated hydatid cysts of the liver. World J Gastrointest Surg. 2010;2(3):78-84.
521	Malik AA, Bari SU, Rouf KA, Wani KA. Pyogenic liver abscess: Changing patterns in approach. World J Gastrointest Surg. 2010;2(12):395-401.
522	Malik AM, Khan A, Talpur KA, Laghari AA. Factors influencing morbidity and mortality in elderly population undergoing inguinal hernia surgery. J Pak Med Assoc. 2010;60(1):45-7.
523	Malik AM, Talpur AH, Laghari AA. Video-assisted laparoscopic extracorporeal appendectomy versus open appendectomy. J Laparoendosc Adv Surg Tech A. 2009;19(3):355-9.
524	Malik M, Chauhan S, Malik V, Gharde P, Kiran U, Pandey RM. Is EuroSCORE applicable to Indian patients undergoing cardiac surgery? Ann Card Anaesth. 2010;13(3):241-5.
525	Maluf MA, Carvalho AC, Carvalho WB. One and a half ventricular repair as an alternative for hypoplastic right ventricle: Operação de um ventrículo e meio como uma alternativa para o ventrículo direito hipoplásico. Rev bras cir cardiovasc. 2010;25(4):466-73.
526	Manning RG, Aziz AQ. Should Laparoscopic Cholecystectomy be Practiced in the Developing World? The Experience of the First Training Program in Afghanistan. Ann Surg. 2009;249(5):794-8.
527	Mansour-Ghanaei F, Joukar F, Soati F, Javadi M. Clinical features of hydatid disease in Guilan (the North Province of Iran): A ten-year study. Archives of Clinical Infectious Diseases. 2012;7(4):119-23.
528	Marin FA, Lamonica-Garcia VC, Henry MA, Burini RC. Grade of esophageal cancer and nutritional status impact on postsurgery outcomes. Arq Gastroenterol. 2010;47(4):348-53.
529	Maroof SA, Khan MJ, Khan Y, Uzair M. Surgical management of omphalomesenteric duct remnants in children. JPMI - Journal of Postgraduate Medical Institute. 2009;23(2):179-83.
530	Mataraci I, Polat A, Toker ME, Tezcan O, Erkin A, Kirali K. Postoperative revision surgery for bleeding in a tertiary heart center. Asian Cardiovasc Thorac Ann. 2010;18(3):266-71.
531	Mbamara SU, Obiechina N, Eleje GU. An analysis of uterine rupture at the Nnamdi Azikiwe University Teaching Hospital Nnewi, Southeast Nigeria. Niger J Clin Pract. 2012;15(4):448-52.
532	Medeiros BA, Iezzi LE, Feitosa MR, Parra RS, Almeida ALNRd, Carvalho RGd, et al. Perineal rectosigmoidectomy on treatment of rectal procidentia: analysis of 48 cases. J coloproctol (Rio J, Impr). 2012;32(3):208-13.
533	Medeiros BA, Iezzi LE, Feitosa MR, Parra RS, Almeida ALNRd, Carvalho RGd, et al. Proctocolectomy and ileal J-pouch anal anastomosis on the surgical treatment of familial adenomatous polyposis and ulcerative colitis: analysis of 49 cases. J coloproctol (Rio J, Impr). 2012;32(3):260-4.
534	Mehrabi Bahar M, Jangjoo A, Amouzeshi A, Kavianifar K. Wound infection incidence in patients with simple and gangrenous or perforated appendicitis. Arch Iran Med. 2010;13(1):13-6.
535	Memon AA, Siddiqui FG, Abro AH, Agha AH, Lubna S, Memon AS. An audit of secondary peritonitis at a tertiary care university hospital of Sindh, Pakistan. World J Emerg Surg. 2012;7:6.
536	Memon AA, Siddiqui FG, Abro AH, Agha AH, Lubna S, Memon AS. Management of recurrent inguinal hernia at a tertiary care hospital of southern Sindh, Pakistan. World J Surg. 2013;37(3):510-5.
537	Memon AA, Zafar H, Raza R, Murtaz G. Traumatic pancreatic injury - An elusive diagnosis: Experience from a developing country urban trauma referral centre. J Pak Med Assoc. 2013;63(4):440-4.
538	Memon AS, Siddiqui FG, Memon HA, Ali SA. Management of ruptured amoebic liver abscess: 22-years experience. J Ayub Med Coll Abbottabad. 2010;22(2):96-9.
539	Memon MR, Sanghi AG, Abbasi SA, Memon AA. Role of laparoscopy in blunt abdominal trauma. Rawal Medical Journal. 2013;38(1):40-3.
540	Memon MR, Shaikh AA, Memon SR, Jamro B. Results of stoppa's sublay mesh repair in incisional & ventral hernias. J Pak Med Assoc. 2010;60(10):798-801.
541	Memon S, Qazi RA, Pushpa, Khushk IA. Pattern of obstructed labour at a public sector university hospital of Sindh, Pakistan. Journal of the Liaquat University of Medical and Health Sciences. 2009;8(1):60-4.
542	Menezes FH, Gomes de Souza VM. Physiologic component of the estimation of physiologic ability and surgical stress scoring system as a predictor of immediate outcome after elective open abdominal aortic aneurysm repair. Ann Vasc Surg. 2011;25(4):485-95.
543	Meng G, Xing Q, Yuan Q, Du Z, Wang Y, Meng H. Internal compared with external drainage of pancreatic duct during pancreaticoduodenectomy: a retrospective study. Chin J Cancer Res. 2014;26(3):277-84.
544	Mesbah Y, Fialla E, Barakat R, Badawy A, Ragab A. Emergency peripartum hysterectomy: The experience of a tertiary referral hospital. Middle East Fertility Society Journal. 2013;18(2):89-93.
545	Mezue WC, Ndubuisi CA, Erechukwu UA, Ohaegbulam SC. Chest injuries associated with head injury. Niger J Surg. 2012;18(1):8-12.
546	Mezue WC, Ohaegbulam SC, Chikani MC, Achebe DN. Management of Giant Pituitary Tumors Affecting Vision in Nigeria. World Neurosurg. 2012;77(5-6):606-9.
547	Mezue WC, Ohaegbulam SC, Ndubuisi CA, Chikani MC, Achebe DS. Management of intracranial meningiomas in Enugu, Nigeria. Surg Neurol Int. 2012;3:110.
548	Miah ZI, Bhuiyan ZH. Outcome of 500 cases of transurethral resection of prostate (TURP) in district level teaching hospital. Bangladesh Journal of Medical Science. 2013;12(2):186-91.
549	Min CM, Sah BK, Xin YX, Meng YQ, Chen L, Ming X, et al. Why the postoperative mortality rate of gastric cancer is lower in our center? Hepatogastroenterology. 2010;57(97):178-84.
550	Mir IS, Mohsin M, Kirmani O, Cheachek BA, Alam I, Wani M. Is laparoscospic orchidectomy the treatment of choice in adults with impalpable testis in rural hospitals in the developing world? Trop Doct. 2009;39(1):12-5.
551	Mir MR, Rajabpour MV, Delarestaghi MM, Hadji M, Harirchi I, Mir P, et al. Short- and long-term survival of esophageal cancer patients treated at the Cancer Institute of Iran. Dig Surg. 2013;30(4-6):331-6.
552	Mirhosseini SJ, Forouzannia SK, Ali-Hassan-Sayegh S, Ravan HV, Abdollahi MH, Mozayan MR. Preoperative C-reactive protein can predict early clinical outcomes following elective off-pump CABG surgery in patients with severe left ventricle dysfunction. Saudi J Anaesth. 2012;6(4):327-31.
553	Mirhosseini SJ, Sayegh SA. Effect of preoperative anemia on short term clinical outcomes in diabetic patients after elective off-pump CABG surgery. Acta Med Iran. 2012;50(9):615-8.
554	Mirshemirani A, Khaleghnejad A, Kouranloo J, Sadeghian N, Rouzrokh M, Hasas-Yeganeh S. Liver Hydatid Cyst in Children (A 14-year Review). Iran J Pediatr. 2011;21(3):385-9.
555	Mirshemirani AR, Razavi S, Sadeghian S. Surgical treatment of pulmonary hydatid cyst in 72 children. Tanaffos. 2009;8(1):56-61.

Reference Number	Full reference
556	Mirza B, Ijaz L, Saleem M, Sheikh A. Surgical aspects of intestinal tuberculosis in children: our experience. Afr J Paediatr Surg. 2011;8(2):185-9.
557	Mocumbi AO, Lameira E, Yaksh A, Paul L, Ferreira MB, Sidi D. Challenges on the management of congenital heart disease in developing countries. Int J Cardiol. 2011;148(3):285-8.
558	Mohamed AA, Mahran KM, Zaazou MM. Blunt abdominal trauma requiring laparotomy in poly-traumatized patients. Saudi Med J. 2010;31(1):43-8.
559	Mohamed AA, Mahran KM. Laparoscopic appendectomy in complicated appendicitis: Is it safe? J Minim Access Surg. 2013;9(2):55-8.
560	Mohanty NK, Kumar A, Vasudeva P, Jain M, Prakash S, Arora RP. Analysis of the perioperative and five-year oncological outcome of two hundred cases of open radical cystectomy: a single center experience. Indian J Cancer. 2012;49(1):96-101.
561	Moiyadi AV, Shetty PM. Perioperative outcomes following surgery for brain tumors: Objective assessment and risk factor evaluation. J Neurosci Rural Pract. 2012;3(1):28-35.
562	Molina G, Funk LM, Rodriguez V, Lipsitz SR, Gawande A. Evaluation of surgical care in El Salvador using the WHO surgical vital statistics. World J Surg. 2013;37(6):1227-35.
563	Monsalve GA, Martínez CM, Gallo T, González MV, Arango G, Upegui A, et al. Paciente embarazada con enfermedad cardiaca: manejo periparto basado en la estratificación del riesgo: serie de casos 2005-2009^ies: Pregnant patient with cardiac diseases: risk based peripartum management: case series 2005-2009. Rev colomb anestesiol. 2010;38(3):348-60.
564	Montufar-Rueda C, Rodriguez L, Jarquin JD, Barboza A, Bustillo MC, Marin F, et al. Severe postpartum hemorrhage from uterine atony: a multicentric study. J Pregnancy. 2013;2013:525914.
565	Moodley J, Khedun SM, Devjee J. Breech presentation at a district level hospital in South Africa. South African Family Practice. 2010;52(1):64-8.
566	Morales C, Barrera L, Moreno M, Villegas M, Correa J, Sucerquia L, et al. Efficacy and safety of non-operative management of blunt liver trauma. Eur J Trauma Emerg Surg. 2011;37(6):591-6.
567	Morrison BF, Coard K, Strachan G, Miller R, Aiken W, Mayhew R. Radical prostatectomy outcomes at the University Hospital of the West Indies: 2000-2007. West Indian Med J. 2011;60(1):68-72.
568	Mosaddeghi KS, Heris HK, Bayat A, Mosaddeghi KZ. Capsulorrhaphy in the management of liver hydatid cyst. Ann Hepatol. 2014;13(3):378-83.
569	Mozafar M, Sobhiyeh MR, Fattahi F, Kish LF, Zirakzadah H. Trans-thoracic versus trans-hiatal esophagectomy complications and outcomes in patients with esophageal cancer in Shohada-E-Tajrish Hospital, Tehran, Iran; 2000-2008. Iranian Red Crescent Medical Journal. 2010;12(4):476-9.
570	Mpabalwani EM, Chitambala P, Chibumbya JN, Matapo B, Mutambo H, Mwenda JM, et al. Intussusception incidence rates in 9 Zambian hospitals, 2007-2011: Prerotavirus vaccine introduction. Pediatr Infect Dis J. 2014;33(SUPPL. 1):S94-S8.
571	Mukasa PK, Kabakyenga J, Senkungu JK, Ngonzi J, Kyalimpa M, Roosmalen VJ. Uterine rupture in a teaching hospital in Mbarara, western Uganda, unmatched case-control study. Reprod Health. 2013;10:29.
572	Mulaudzi TV, Robbs JV, Paruk N, Pillay B, Madiba TE, Govindsamy V. The influence of diabetes on short-term outcome following a prosthetic above-the-knee femoro-popliteal bypass. Cardiovasc J Afr. 2009;20(3):170-2.
573	Mumtaz K, Kamani L, Chawla T, Hamid S, Jafri W. Hepatic cystic echinococcosis: clinical characteristics and outcomes in Pakistan. Trop Doct. 2009;39(4):215-7.
574	Murtaza B, Saeed S, Sharif MA. Postoperative complications in emergency versus elective laparotomies at a peripheral hospital. J Ayub Med Coll Abbottabad. 2010;22(3):42-7.
575	Mushtaque M, Mir MF, Bhat M, Parray FQ, Khanday SA, Dar RA, et al. Pellet gunfire injuries among agitated mobs in Kashmir. Ulusal Travma ve Acil Cerrahi Dergisi. 2012;18(3):255-9.
576	Mutyaba AK, Balkaran S, Cloete R, du Plessis N, Badri M, Brink J, et al. Constrictive pericarditis requiring pericardiectomy at Groote Schuur Hospital, Cape Town, South Africa: Causes and perioperative outcomes in the HIV era (1990-2012). J Thorac Cardiovasc Surg. 2014.
577	Muyembe-Tamfum JJ, Veyi J, Kaswa M, Lunguya O, Verhaegen J, Boelaert M. An outbreak of peritonitis caused by multidrug-resistant Salmonella Typhi in Kinshasa, Democratic Republic of Congo. Travel Med Infect Dis. 2009;7(1):40-3.
578	Najam RB, P.; Sharma, R.; Agarwal, D. Emergency obstetric hysterectomy: a retrospective study at a tertiary care hospital. J Clin Diagn Res. 2010;4:2864-8.
579	Narasimman S, Nallusamy M, Hassan S. Review of oesophageal atresia and tracheoesophageal fistula in hospital sultanah bahiyah, alor star. Malaysia from january 2000 to december 2009. Med J Malaysia. 2013;68(1):48-51.
580	Nasio NA. Perforated Peptic Ulcer Disease at Kenyatta National Hospital, Nairobi. East and Central African Journal of Surgery. 2009;14(1):13-7.
581	Nasir AA, Abdur-Rahman LO, Adeniran JO. Outcomes of surgical treatment of malrotation in children. Afr J Paediatr Surg. 2011;8(1):8-11.
582	Nasir AA, Abdur-Rahman LO, Adeniran JO. Predictor of mortality in children with typhoid intestinal perforation in a Tertiary Hospital in Nigeria. Pediatr Surg Int. 2011;27(12):1317-21.
583	Nassar OAH. Modified Pseudocontinent Perineal Colostomy: A Special Technique. Dis Colon Rectum. 2011;54(6):718-28.
584	Nazeem WM, El-Beltagy M, Kamal HM. Intracranial meningiomas in children: Surgical experience. Egyptian Journal of Neurology, Psychiatry and Neurosurgery. 2012;49(3):219-24.
585	Nazem M, Hosseinpour M. Evaluation of early and late complications in patients with congenital lobar emphysema: a 12 year experience. Afr J Paediatr Surg. 2010;7(3):144-6.
586	Nega B. Pattern of acute abdomen and variables associated with adverse outcome in a rural primary hospital setting. Ethiop Med J. 2009;47(2):143-51.
587	Nega B. Truncal Vagotomy for Peptic Pyloric Stenosis and Assessment of Completeness by Acid Tests. East and Central African Journal of Surgery. 2010;15(1):40-7.
588	Neumann CR, Brasil AV, Albers F. Risk factors for mortality in traumatic cervical spinal cord injury: Brazilian data. J Trauma. 2009;67(1):67-70.
589	Nguyen HS, Vu TD, Nguyen TQ. A modified carpentier's technique for Ebstein's anomaly repair. J Card Surg. 2014;29(4):554-60.
590	Nikibakhsh AA, Mahmoodzadeh H, Vali M, Enashaei A, Asem A, Yekta Z. Outcome of immediate use of the permanent peritoneal dialysis catheter in children with acute and chronic renal failure. Iran J Pediatr. 2013;23(2):171-6.
591	Niramis R, Anuntkosol M, Tongsin A, Mahatharadol V. Influence of Down's syndrome on management and outcome of patients with congenital intrinsic duodenal obstruction. J Pediatr Surg. 2010;45(7):1467-72.
592	Nisar N, Sohoo NA. Emergency peripartum hysterectomy: frequency, indications and maternal outcome. J Ayub Med Coll Abbottabad. 2009;21(1):48-51.
593	Nizam K, Haider G, Memon N, Haider A. Cesarean section rate: Much room for reduction. Rawal Medical Journal. 2010;35(1):19-22.
594	Nogueira L, Reis RBd, Machado RD, Tobias-Machado M, Carvalhal G, Freitas Jr C, et al. Cutaneous ureterostomy with definitive ureteral stent as urinary diversion option in unfit patients after radical cystectomy: Ureterostomia cutânea como opção de derivação urinária em pacientes em condições clínicas após cistectomia radical. Acta cir bras. 2013;28(supl.1):43-7.
595	Nooraei N, Hashemian SMR, Golfam A, Saghebi SR, Radmand G. Preoperative assessment of mechanical ventilation requirement after surgical treatment of esophageal cancer. Tanaffos. 2010;9(1):34-41.
596	Nooren M, Nawal R. Obstetric hysterectomy: a life saving emergency. Indian J Med Sci. 2013;67(5-6):99-102.
597	Novaes FR, Navarro TP, Bernardes RdC, Pinto FARR, Lima LCM, Monteiro ELdS, et al. Results of Castro Bernardes intraluminal ring in surgery for ascending aortic aneurysms and dissections: Resultados do anel intraluminal de Castro Bernardes nas cirurgias de aneurismas e dissecções da aorta ascendente. Rev bras cir cardiovasc. 2013;28(2):176-82.
598	Nuhu A, Dahwa S, Hamza A. Operative management of typhoid ileal perforation in children. Afr J Paediatr Surg. 2010;7(1):9-13.
599	Nuhu A, Jah A. Acute sigmoid volvulus in a West African population. Ann Afr Med. 2010;9(2):86-90.
600	Nuhu A, Madziga AG, Gali BM. Acute perforated duodenal ulcer in Maiduguri: experience with simple closure and Helicobacter pylori eradication. West Afr J Med. 2009;28(6):384-7.
601	Nuhu A, Samateh A. DAY CASE HAEMORRHOIDECTOMY IN A DEVELOPING COUNTRY. Niger J Clin Pract. 2009;12(1):51-3.

Reference Number	Full reference
602	Nunes JC, Braz JRC, Oliveira TS, De Carvalho LR, Castiglia YMM, Braz LG. Intraoperative and anesthesia-related cardiac arrest and its mortality in older patients: A 15-year survey in a tertiary teaching hospital. PLoS One. 2014;9(8).
603	Nwafor MI, Aniebue UU, Nwankwo TO, Onyeka TC, Okafor VU. Perinatal outcome of preterm cesarean section in a resource-limited centre: a comparison between general anaesthesia and subarachnoid block. Niger J Clin Pract. 2014;17(5):613-8.
604	Nwankwo OE, Uche EO. Epidemiological and treatment profiles of spinal cord injury in southeast Nigeria. Spinal Cord. 2013;51(6):448-52.
605	Nwobodo E, Nnadi D. Emergency obstetric hysterectomy in a tertiary hospital in sokoto, Nigeria. Ann Med Health Sci Res. 2012;2(1):37-40.
606	Nwobodo EI, Isah AY, Panti A. Elective caesarean section in a tertiary hospital in Sokoto, north western Nigeria. Niger Med J. 2011;52(4):263-5.
607	Nyamtema AS, Pemba SK, Mbaruku G, Rutasha FD, van Roosmalen J. Tanzanian lessons in using non-physician clinicians to scale up comprehensive emergency obstetric care in remote and rural areas. Human Resources for Health. 2011;9:8.
608	Nyengidiki TK, Allagoa DO. Rupture of the gravid uterus in a tertiary health facility in the Niger delta region of Nigeria: A 5-year review. Niger Med J. 2011;52(4):230-4.
609	Obalum DC, Okeke GC. Lower limb amputations at a Nigerian private tertiary hospital. West Afr J Med. 2009;28(1):24-7.
610	Obeidat B, Zayed F, Amarin Z, Obeidat N, El-Jallad MF. Tubal ectopic pregnancy in the north of Jordan: Presentation and management. Clin Exp Obstet Gynecol. 2010;37(2):138-40.
611	Obiechina NJ, Eleje GU, Ezebialu IU, Okeke CA, Mbamara SU. Emergency peripartum hysterectomy in Nnewi, Nigeria: a 10-year review. Niger J Clin Pract. 2012;15(2):168-71.
612	Obiechina NJ, Ugboaja JO, Onyegbule OA, Eleje GU. Vaginal hysterectomy in a Nigerian tertiary health facility. Niger J Med. 2010;19(3):324-5.
613	Ogelle O, Okafor C, Eke AC, Obiechina N, Mbamara S. Current trends in hysterectomies at a Nigerian tertiary center. J Gynecol Surg. 2010;26(1):7-13.
614	Ogundoyin OO, Afolabi AO, Ogunlana DI, Lawal TA, Yifieyeh AC. Pattern and outcome of childhood intestinal obstruction at a tertiary hospital in Nigeria. Afr Health Sci. 2009;9(3):170-3.
615	Ohene-Yeboah M, Dakubo JC, Boakye F, Naeeder SB. Penetrating abdominal injuries in adults seen at two teaching hospitals in ghana. Ghana Med J. 2010;44(3):103-8.
616	Okafor UV, Azike J. A review of anaesthesia for emergency laparotomy in paediatric intestinal obstruction in Enugu, Nigeria. Pan Afr Med J. 2009;3:7.
617	Okafor UV, Efetie ER, Nwoke O, Okezie O, Umeh U. Anaesthetic and Obstetric challenges of morbid obesity in caesarean deliveries-a study in South-eastern Nigeria. Afr Health Sci. 2012;12(1):54-7.
618	Okafor UV, Ezegwui HU, Ekwazi K. Trends of different forms of anaesthesia for caesarean section in South-eastern Nigeria. J Obstet Gynaecol. 2009;29(5):392-5.
619	Okafor UVE, H.U.; Okezie, O. Anaesthetic challenges in emergency peripartum hysterectomy in West Africa: a Nigerian perspective. South Afr J Anaesth Analgesia 2010;16:8-11.
620	Okonta K, Anbarasu M, Kanagarajan K. INTRA-AORTIC BALLOON PUMP IN CORONARY ARTERY BYPASS GRAFT - FACTORS AFFECTING OUTCOME. J West Afr Coll Surg. 2011;1(4):28-40.
621	Okorie CO, Pisters LL. Modifying and increasing day-case procedures to solve local problems: Experience of a urology unit. Niger Med J. 2012;53(1):26-30.
622	Okoro PE, Igwe P, Opara PI. Pattern and survival of biliary atresia patients; experience in southern Nigeria. Niger J Surg. 2013;19(1):4-6.
623	Oladapo OT, Akinsanya AF. Relative morbidity of abdominal myomectomy for very large uterine fibroids in a developing country hospital. Arch Gynecol Obstet. 2011;283(4):825-30.
624	Olamijulo JA, Abiara OE, Olaleye OO, Ogedengbe OK, Giwa-Osagie F, Oluwole OO. Emergency obstetric hysterectomy in a Nigerian teaching hospital: a ten-year review. Nig Q J Hosp Med. 2013;23(1):69-74.
625	Olavarrieta JRL, Coronel P. Expectativas y satisfacción en el tratamiento del neumotórax espontáneo primario recurrente tratado por toracotomía o cirugía torácica video-asistida^ies: Expectations and patient satisfaction related to the use of thoracotomy and video-assisted thoracoscopic surgery for treating recurrence of spontaneous primary pneumothorax. J bras pneumol. 2009;35(2):122-8.
626	Olayiwola B, Fadeyibi IO, Jewo PI, Sanyaolu NO, Bankole MA. Day-case herniotomy surgery for children with inhalational anaesthesia in Lagos, Nigeria. Macedonian Journal of Medical Sciences. 2011;4(2):163-6.
627	Oliveira Júnior JdL, Fiorelli AI, Santos RHB, Pomerantzeff PAM, Dallan LAdO, Stolf NAG. A doença coronária aumenta a mortalidade hospitalar de portadores de estenose aórtica submetidos à substituição valvar?: Does the coronary disease increase the hospital mortality in patients with aortic stenosis undergoing valve replacement? Rev bras cir cardiovasc. 2009;24(4):453-62.
628	Oliveira MFd, Saad F, Reis RC, Rotta JM, Pinto FCG. Programmable valve represents an efficient and safe tool in the treatment of idiopathic normal-pressure hydrocephalus patients: Válvula de pressão programável representa uma ferramenta eficaz e segura no tratamento de pacientes com hidrocefalia de pressão normal idiopática. Arq neuropsiquiatr. 2013;71(4):229-36.
629	Oliveira TM, Oliveira GM, Klein CH, Souza ESNA, Godoy PH. Mortality and complications of coronary artery bypass grafting in Rio de Janeiro, from 1999 to 2003. Arq Bras Cardiol. 2010;95(3):303-12.
630	Omisanjo OA, Bioku MJ, Ikuerowo SO, Sule GA, Esho JO. Clinical characteristics and outcome of management of Fournier's gangrene at the Lagos State University Teaching Hospital, Ikeja, Lagos, Nigeria. Ann Afr Med. 2014;13(4):174-8.
631	Omole-Ohonsi A, Ashimi OA. Non-emergency hysterectomy: why the aversion? Arch Gynecol Obstet. 2009;280(6):953-9.
632	Omole-Ohonsi A, Belga F. Surgical management of uterine fibroids at aminu kano teaching hospital. Obstet Gynecol Int. 2012;2012:702325.
633	Omole-Ohonsi A, Olayinka HT. Emergency peripartum hysterectomy in a developing country. J Obstet Gynaecol Can. 2012;34(10):954-60.
634	Onan B, Demirhan R, Oz K, Onan IS. Cardiac and great vessel injuries after chest trauma: our 10-year experience. Ulus Travma Acil Cerrahi Derg. 2011;17(5):423-9.
635	Onan IS, Erek E, Haydin S, Onan B, Kocyigit OI, Topuz U, et al. Clinical outcome of patients in a start-up congenital heart surgery program in Turkey. Artif Organs. 2013;37(1):E18-23.
636	Onat S, Ulku R, Avci A, Ates G, Ozcelik C. Urgent thoracotomy for penetrating chest trauma: analysis of 158 patients of a single center. Injury. 2011;42(9):900-4.
637	Onder A, Gumus M, Kapan M, Boyuk A, Arikanoglu Z, Girgin S. Associated organ injuries in pancreatic injuries, morbidity, and mortality. Turkish Journal of Medical Sciences. 2012;42(4):559-66.
638	Oneko O, Petru E, Masenga G, Ulrich D, Obure J, Zeck W. Management of the placenta in advanced abdominal pregnancies at an East African tertiary referral center. J Womens Health. 2010;19(7):1369-75.
639	Onyebuchi AK, Lawani LO, Nkwo PO, Iyoke CA, Onoh RC, Ajah LO. Determinants of decision-to-intervention time in the management and therapeutic outcome of emergency gynecological surgeries in south east Nigeria. Ther Clin Risk Manag. 2014;10(1):577-82.
640	Opara PIU, A.S.; Okoro, P.E. Surgical admissions in a newborn unit in a low resource setting, challenges in management and outcomes. J Neonatal Biol. 2014;3(2):132.
641	Oral A, Yigiter M, Yildiz A, Yalcin O, Dikmen T, Eren S, et al. Diagnosis and management of hydatid liver disease in children: a report of 156 patients with hydatid disease. J Pediatr Surg. 2012;47(3):528-34.
642	Ordonez CA, Badiel M, Pino LF, Salamea JC, Loaiza JH, Parra MW, et al. Damage control resuscitation: Early decision strategies in abdominal gunshot wounds using an easy "aBCD" mnemonic. Journal of Trauma and Acute Care Surgery. 2012;73(5):1074-8.
643	Oribabor FO, Adebayo BO, Aladesanmi T, Akinola DO. Perforated duodenal ulcer; management in a resource poor, semi-urban nigerian hospital. Niger J Surg. 2013;19(1):13-5.
644	Osaikhuwuomwan JA, Ande AB. Reappraisal of ruptured uterus in an urban tertiary center in the Niger-delta region of Nigeria. Journal of Maternal-Fetal and Neonatal Medicine. 2011;24(4):559-63.
645	Osifo OD, Efobi AC. Challenges of giant ventral hernia repair in children in an African tertiary care center with limited resources. Hernia. 2009;13(2):143-7.

Reference Number	Full reference
646	Osifo OD, Evbuomwan I. Primary perineal surgeries for the low and intermediate anorectal anomalies: 5-year results in a developing country. Surgical Practice. 2009;13(3):64-8.
647	Osifo OD, Mene AO. Hypospadias repair in a resource-poor region: coping with the challenges in 5 years. J Pediatr Urol. 2010;6(1):60-5.
648	Osifo OD, Ogiemwonyi SO. Appendicitis in children: An increasing health scourge in a developing country. Pakistan Journal of Medical Sciences. 2009;25(3):490-5.
649	Osifo OD, Ogiemwonyi SO. Peritonitis in children: our experience in Benin City, Nigeria. Surg Infect (Larchmt). 2011;12(2):127-30.
650	Osifo OD, Okolo JC. Neonatal intestinal obstruction in Benin, Nigeria. Afr J Paediatr Surg. 2009;6(2):98-101.
651	Osifo OD, Ovueni ME, Evbuomwan I. Omphalocele management using goal-oriented classification in African centre with limited resources. J Trop Pediatr. 2011;57(4):286-8.
652	Osifo ODO, M. E. The Predictors, Prevalence and Outcome of Burst Abdomen in Emergency Paediatric Surgical Centre. East and Central African Journal of Surgery. 2010;15(2).
653	Osifo ODO, M.E. Duodenal obstruction: etiology, morbidity and mortality among edo state children, Nigeria. Afr J Biomed Res. 2009;12(3):193–7.
654	Oucheng N, Lauwers F, Gollogly J, Draper L, Joly B, Roux FE. Frontoethmoidal meningoencephalocele: appraisal of 200 operated cases. J Neurosurg Pediatr. 2010;6(6):541-9.
655	Ozdemir AC, Emrecan B, Baltalarli A. Bileaflet versus posterior-leaflet-only preservation in mitral valve replacement. Tex Heart Inst J. 2014;41(2):165-9.
656	Ozer I, Bostanci EB, Koc U, Karaman K, Ercan M, Ulas M, et al. Surgical treatment for gastric cancer in Turkish patients over age 70: early postoperative results and risk factors for mortality. Langenbecks Arch Surg. 2010;395(8):1101-6.
657	Ozogul YB, Ulas M, Ozer I, Ercan M, Gomceli I, Birol Bostanci E, et al. Short-term outcomes after surgery for colorectal cancer in Turkish patients aged 70 and above. Turk J Gastroenterol. 2010;21(3):257-61.
658	Ozyurtkan MO, Balci AE. Surgical treatment of intrathoracic hydatid disease: a 5-year experience in an endemic region. Surg Today. 2010;40(1):31-7.
659	Pais-Costa SR, Araujo SLM, Lima OAT, Teixeira ACP. Hepatectomia laparoscópica: indicações e resultados em 18 casos ressecados: Laparoscopic hepatectomy: indications and results from 18 resectable cases. Einstein (São Paulo). 2011;9(3).
660	Paiva-Neto MAd, Tella-Júnior OId. Supra-orbital keyhole removal of anterior fossa and parasellar meningiomas: Minicraniotomia supra-orbitária superciliar no tratamento de meningiomas na fossa craniana anterior e para-selares. Arq neuropsiquiatr. 2010;68(3):418-23.
661	Pal KM, Bari H, Nasim S. Pancreaticoduodenectomy: a developing country perspective. J Pak Med Assoc. 2011;61(3):232-5.
662	Pan XF, Tantai JC, Lin L, Cao KJ, Zhao H. Comparison of short and long-term results between sleeve resection and pneumonectomy in lung cancer patients over 70 years old: 10 years experience from a single institution in China. Thoracic Cancer. 2014;5(6):494-9.
663	Pande S, Agarwal SK, Gupta D, Mohanty S, Kapoor A, Tewari S, et al. Early and mid-term results of minimally invasive coronary artery bypass grafting. Indian Heart J. 2014;66(2):193-6.
664	Pardhan A, Hameed A, Zafar H, Mazahir S, Murtaza G. Outcomes of Splenectomy for Idiopathic Thrombocytopenic Purpura in adults: A Developing Country Perspective. J Pak Med Assoc. 2014;64(11):1240-3.
665	Parsak CK, Tuncer U, Kesiktas E, Akcam T, Sakman G, Ozdemir S, et al. Reconstruction of cervical esophagus defects by free jejunal flap after proximal esophageal carcinoma resections. Advances in Clinical and Experimental Medicine. 2011;20(6):729-36.
666	Parveen Z, Qureshi AN, Akbar M, Zafar A, Subhani A. Palliative surgery for intestinal obstruction due to recurrent ovarian cancer. J Ayub Med Coll Abbottabad. 2009;21(1):135-6.
667	Paulista MD, Paulista PHD, Guerra ALP, Paulista PP. Tratamento cirúrgico da conexão anômala parcial das veias pulmonares em veia cava superior: Surgical treatment of partial anomalous pulmonary venous connection to the superior vena cava. Rev bras cir cardiovasc. 2009;24(2):133-7.
668	Peer SM, Devaraddeppa PM, Buggi S. Traumatic diaphragmatic hernia-our experience. Int J Surg. 2009;7(6):547-9.
669	Pei G, Zhou S, Han Y, Liu Z, Xu S. Risk factors for postoperative complications after lung resection for non-small cell lung cancer in elderly patients at a single institution in China. J Thorac Dis. 2014;6(9):1230-8.
670	Pembe AB, Othman MK. Pregnancy outcome after one previous caesarean section at a tertiary university teaching hospital in Tanzania. Tanzania Journal of Health Research. 2010;12(3):188-94.
671	Pembe ABW, Peter J. T. ; Massawe, Siriel N. Emergency Peripartum Hysterectomies at Muhimbili National Hospital, Tanzania: A Review of Cases from 2003 to 2007. Tanzania Journal of Health Research. 2012;14(1).
672	Pereira C, Mbaruku G, Nzabuhakwa C, Bergstrom S, McCord C. Emergency obstetric surgery by non-physician clinicians in Tanzania. International Journal of Gynecology and Obstetrics. 2011;114(2):180-3.
673	Pereira LC, Oliveira KM, L'Abbate GL, Sugai R, Ferreira JA, da Motta LA. Outcome of fully awake craniotomy for lesions near the eloquent cortex: analysis of a prospective surgical series of 79 supratentorial primary brain tumors with long follow-up. Acta Neurochir (Wien). 2009;151(10):1215-30.
674	Pereira SR, Puts MT, Portela MC, Sayeg MA. The impact of prefracture and hip fracture characteristics on mortality in older persons in Brazil. Clin Orthop Relat Res. 2010;468(7):1869-83.
675	Perveen F, Memon GU, Rabia S. Use of bilateral internal iliac artery ligation for controlling severe obstetric haemorrhage. Pakistan Journal of Medical Sciences. 2011;27(1):94-7.
676	Phadungkiatwattana P, Tongsakul N. Analyzing the impact of private service on the cesarean section rate in public hospital Thailand. Arch Gynecol Obstet. 2011;284(6):1375-9.
677	Piegas LS, Bittar OJNV, Haddad N. Cirurgia de revascularização miocárdica: resultados do Sistema Único de Saúde: Myocardial revascularization surgery (MRS): results from National Health System (SUS): Cirugía de revascularización miocárdica: resultados del Sistema Único de Salud^ies. Arq bras cardiol. 2009;93(5):513-60.
678	Pires de Aguiar PH, Tahara A, Agner C, Calfat Maldaun MV, Theodoros Panagopoulos A, Matsushige T, et al. Intraventricular meningiomas in adults: clinical series and review of the literature. Rev chil neurocir. 2011;37:23-8.
679	Pires LV, Uberti EMH, Fajardo MD, da Cunha AGV, Rosa MW, Ayub ACK, et al. Role of Hysterectomy in the Management of Patients with Gestational Trophoblastic Neoplasia Importance of Receiving Treatment in Reference Centers. J Reprod Med. 2012;57(7-8):359-68.
680	Pitcher GJ, Davies MR, Bowley DM, Numanoglu A, Rode H. Fetal extraperitoneal rectal perforation: a rare neonatal emergency. J Pediatr Surg. 2009;44(7):1405-9.
681	Pivatto Júnior F, Kalil RAK, Costa AR, Pereira EMC, Santos EZ, Valle FH, et al. Morbimortalidade em octogenários submetidos à cirurgia de revascularização miocárdica: Morbimortality in octogenarian patients submitted to coronary artery bypass graft surgery. Arq bras cardiol. 2010;95(1):41-6.
682	Pokharel N, Sapkota P, Kc B, Rimal S, Thapa S, Shakya R. Acute appendicitis in elderly patients: a challenge for surgeons. Nepal Med Coll J. 2011;13(4):285-8.
683	Poletti GB, Toro IFC, Alves TF, Miranda ECM, Seabra JCT, Mussi RK. Descriptive analysis of and overall survival after surgical treatment of lung metastases: Analise descritiva e sobrevida global do tratamento cirurgico das metastases pulmonares. J bras pneumol. 2013;39(6):650-8.
684	Pomerantzeff PMA, Brandão CMdA, Leite Filho OA, Guedes MAV, Silva MFd, Grinberg M, et al. Plástica da valva mitral em pacientes com insuficiência mitral reumática: técnicas e resultados de 20 anos: Mitral valve repair in rheumatic patients with mitral insuficiency: twenty years of techniques and results. Rev bras cir cardiovasc. 2009;24(4):485-9.
685	Pourzand A, Fakhri BA, Azhough R, Hassanzadeh MA, Hashemzadeh S, Bayat AM. Management of high-risk popliteal vascular blunt trauma: Clinical experience with 62 cases. Vascular Health and Risk Management. 2010;6(1):613-8.
686	Pradhan GB, Shrestha D, Shrestha S, Bhattachan CL. Inguinal herniotomy in children: a one year survey at Nepal Medical College Teaching Hospital. Nepal Med Coll J. 2011;13(4):301-2.
687	Pradhan M, Shao Y. Emergency Peripartum Hysterectomy as Postpartum Hemorrhage Treatment: Incidence, Risk factors, and Complications. JNMA J Nepal Med Assoc. 2014;52(193):668-76.
688	Qadir I, Perveen S, Furnaz S, Shahabuddin S, Sharif H. Risk stratification analysis of operative mortality in isolated coronary artery bypass graft patients in Pakistan: comparison between additive and logistic EuroSCORE models. Interact Cardiovasc Thorac Surg. 2011;13(2):137-41.

Reference Number	Full reference
689	Qadir I, Salick MM, Perveen S, Sharif H. Mortality from isolated coronary bypass surgery: a comparison of the Society of Thoracic Surgeons and the EuroSCORE risk prediction algorithms. Interact Cardiovasc Thorac Surg. 2012;14(3):258-62.
690	Qamarunisa, Memon H, Ali M. Frequency, maternal and fetal outcome of abruptio placenta in a rural medical college hospital, Mirpurkhas Sindh. Pakistan Journal of Medical Sciences. 2010;26(3):663-6.
691	Qazi Q, Akhtar Z, Khan K. Clinical presetations and complications associated with tubal rupture in patients with tubal ectopic pregnancy. JPMI - Journal of Postgraduate Medical Institute. 2010;24(4):312-7.
692	Qiao G, Li L, Li S, Tang S, Wang B, Xi H, et al. Laparoscopic cyst excision and Roux-Y hepaticojejunostomy for children with choledochal cysts in China: a multicenter study. Surg Endosc. 2014.
693	Qiao QL, Zhang TP, Guo JC, Zhan HX, Zhao JX, Liu YC, et al. Prognostic factors after pancreatoduodenectomy for distal bile duct cancer. Am Surg. 2011;77(11):1445-8.
694	Han, Q-q, Song Z-g, Zou L-j, Han L, Lu F-l, Lang X-l, et al. Reinforced aortic root reconstruction for acute type A aortic dissection involving the aortic root: Reconstrução da raiz da aorta reforçada para dissecção aguda da aorta tipo A envolvendo a raiz da aórtica. Rev bras cir cardiovasc. 2013;28(2):190-9.
695	Queiroz AB, Schneidwind K, Mulatti GC, Santo F, Neto PS, Torres IO, et al. Repair of ruptured abdominal aortic aneurysms with bifurcated endografts: a single-center study. Clinics. 2014;69(6):420-5.
696	Raagab AE, Mesbah YH, Brakat RI, Zayed AA, Alsaammani MA. Re-laparotomy after cesarean section: risk, indications and management options. Med Arch. 2014;68(1):41-3.
697	Rabiu KA, Adewunmi AA, Akinola OI, Eti AE, Tayo AO. Comparison of maternal and neonatal outcomes following caesarean section in second versus first stage of labour in a Tertiary Hospital in Nigeria. Niger Postgrad Med J. 2011;18(3):165-71.
698	Raboi A, Al-Motarreb A, Al-Kanadi A, Abdulmughni AA, Kadi A. Mechanical valve dysfunction in yemen. Heart Views. 2010;11(2):47-51.
699	Rachid S, Didier LJ, Bade MA, Sani CM, Habibou A. Laparoscopic cholecystectomy in sickle cell patients in Niger. Pan Afr Med J. 2009;3:19.
700	Radak D, Milojevic P, Babic S, Matic P, Tanaskovic S, Vukotic V, et al. Renal tumor with tumor thrombus in inferior vena cava and right atrium: The report of five cases with long-term follow-up. Int Urol Nephrol. 2011;43(4):1033-8.
701	Radinovic KS, Markovic-Denic L, Dubljanin-Raspopovic E, Marinkovic J, Jovanovic LB, Bumbasirevic V. Effect of the overlap syndrome of depressive symptoms and delirium on outcomes in elderly adults with hip fracture: a prospective cohort study. J Am Geriatr Soc. 2014;62(9):1640-8.
702	Rahman GA. Possible risk factors for respiratory complications after thyroidectomy: An observational study. Ent-Ear Nose & Throat Journal. 2009;88(4):890-2.
703	Rahman GA. Rectal cancer: pattern and outcome of management in University of Ilorin Teaching Hospital, Ilorin, Nigeria. Ann Afr Med. 2010;9(3):164-9.
704	Rahmanian A, Jamali M, Razmkon A, Kivelev J, Romani R, Alibai EA, et al. Benefits of early aneurysm surgery: Southern Iran experience. Surg Neurol Int. 2012;3:156.
705	Ramku EM, Ramku RM, Behramaj AM, Heta AM. Considerations for children's tracheostomy report of 30 patients seen in a 4 year period. Niger J Med. 2009;18(1):59-62.
706	Ramli RR, Hassan S, Adil ARS, Abdullah B. Outcome of neck dissections in a rural tertiary University Hospital in Malaysia. Pakistan Journal of Medical Sciences. 2011;27(1):233-5.
707	Ramos Júnior F, Marques JAP, Albuquerque LAFd, Santos FdP, Almeida JPCd, Lucena JMPd. Surgical management of intracranial aneurysms in the "coiling age". Arq bras neurocir. 2009;28(1):14-8.
708	Raso JL, Darwich RZ, Lucca Júnior Fd, Santana RV, Tanure MT, Cariri GA, et al. Bridge-therapy with enoxaparin in the preoperative period of endarterectomy: Terapia-ponte com enoxaparina no período pré-operatório da endarterectomia. Arq neuropsiquiatr. 2010;68(5):775-7.
709	Rathi PK, Shaikh AR, Kella N, Behan RB. Laparoscopic cholecystectomy without the use of drain in selected cases. Journal of the Liaquat University of Medical and Health Sciences. 2011;10(3):117-20.
710	Rathod JB, Shah DK, Yagnik BD, Yagnik VD. Upper gastrointestinal bleeding: audit of a single center experience in Western India. Clin Pract. 2011;1(4):e132.
711	Raza MS, Jaffery SAY, Khan FA. Flexor zone 5 cut injuries: Emergency management and outcome. Journal of the College of Physicians and Surgeons Pakistan. 2014;24(3):194-7.
712	Reddy D, Muckart DJ. Holes in the heart: an atlas of intracardiac injuries following penetrating trauma. Interact Cardiovasc Thorac Surg. 2014;19(1):56-63.
713	Redman LA, Naidoo P, Biccard BM. HIV, vascular surgery and cardiovascular outcomes: a South African cohort study. Anaesthesia. 2014;69(3):208-13.
714	Redwan AA. Complex post-cholecystectomy biliary injuries: Management with 10 years' experience in a major referral center. Journal of Laparoendoscopic and Advanced Surgical Techniques. 2012;22(6):539-49.
715	Rehman L, Khan HAQ, A.S MH. Outcome of cloward technique in cervical disc prolapse. Journal of the College of Physicians and Surgeons Pakistan. 2010;20(11):733-7.
716	Rehman ZU, Alvi AR, Khan S. Intussusceptions in adults: Clinical features and operative procedures. Journal of the College of Physicians and Surgeons Pakistan. 2010;20(12):790-3.
717	Rekik S, Trabelsi I, Maaloul I, Hentati M, Hammami A, Frikha I, et al. Short- and long-term outcomes of surgery for active infective endocarditis: a Tunisian experience. Interact Cardiovasc Thorac Surg. 2009;9(2):241-5.
718	Rekik S, Trabelsi I, Znazen A, Maaloul I, Hentati M, Frikha I, et al. Prosthetic valve endocarditis: management strategies and prognosis A ten-year analysis in a tertiary care centre in Tunisia. Neth Heart J. 2009;17(2):56-60.
719	Ren L, Upadhyay AM, Wang L, Li L, Lu J, Fu W. Mortality rate prediction by Physiological and Operative Severity Score for the Enumeration of Mortality and Morbidity (POSSUM), Portsmouth POSSUM and Colorectal POSSUM and the development of new scoring systems in Chinese colorectal cancer patients. Am J Surg. 2009;198(1):31-8.
720	Ren M, Meng Q, Zhou W, Kong F, Yang B, Yuan J, et al. Comparison of short-term effect of thoracoscopic segmentectomy and thoracoscopic lobectomy for the solitary pulmonary nodule and early-stage lung cancer. Onco Targets Ther. 2014;7:1343-7.
721	Restrepo CA, Buitrago CA, Holguin C. Implantation of peritoneal catheters by laparotomy: nephrologists obtained similar results to general surgeons. Int J Nephrol Renovasc Dis. 2014;7:383-90.
722	Rezende KF, Ferraz MB, Malerbi DA, Melo NH, Nunes MP, Pedrosa HC, et al. Direct costs and outcomes for inpatients with diabetes mellitus and foot ulcers in a developing country: The experience of the public health system of Brazil. Diabetes and Metabolic Syndrome: Clinical Research and Reviews. 2009;3(4):228-32.
723	Ribeiro GS, Tartof SY, Oliveira DW, Guedes AC, Reis MG, Riley LW, et al. Surgery for valvular heart disease: a population-based study in a Brazilian urban center. PLoS One. 2012;7(5):e37855.
724	Ribeiro TA, Premaor MO, Larangeira JA, Brito LG, Luft M, Guterres LW, et al. Predictors of hip fracture mortality at a general hospital in South Brazil: an unacceptable surgical delay. Clinics (Sao Paulo). 2014;69(4):253-8.
725	Rivetti LA, Campagnucci VP. Tratamento cirúrgico das taquiarritmias: qual o paciente ideal?: [revisão]. RELAMPA, Rev Lat-Am Marcapasso Arritm. 2011;24(1):18-21.
726	Robbs JV, Paruk N. Management of HIV Vasculopathy - A South African Experience. Eur J Vasc Endovasc Surg. 2010;39(SUPPL. 1):S25-S31.
727	Rodrigues AJ, Evora PRB, Bassetto S, Alves Júnior L, Scorzoni Filho A, Araújo WF, et al. Fatores de risco para lesão renal aguda após cirurgia cardíaca: Risk factors for acute renal failure after heart surgery. Rev bras cir cardiovasc. 2009;24(4):441-6.
728	Rodrigues AJ, Évora PRB, Bassetto S, Alves Júnior L, Scorzoni Filho A, Vicente WVA. Substituição valvar isolada com próteses metálicas St. Jude Medical em posição aórtica ou mitral: seguimento de médio prazo: Sustitución valvular aislada con prótesis metálicas St. Jude Medical en posición aórtica o mitral: seguimiento de medio plazo^ies: Isolated mitral and aortic valve replacement with the St. Jude Medical valve: a midterm follow-up. Arq bras cardiol. 2009;93(3):290-8.
729	Rodrigues LCL, Bortoletto A, Matsumoto MH. Effectiveness of the surgical intervention in the quality of life and survival of patients with metastatic lesions in the spine: Efetividade da intervenção cirúrgica na qualidade de vida e na sobrevida dos pacientes com lesões metastáticas na coluna vertebral: Efectividad de la intervención quirúrgica en la calidad de vida y en la sobrevida de los pacientes con lesiones metastásicas en la columna vertebral^ies. Coluna/Columna. 2014;13(1):57-62.

Reference Number	Full reference
730	Roodpeyma S, Hekmat M, Dordkhar M, Rafieyian S, Hashemi A. A prospective observational study of paediatric cardiac surgery outcomes in a postoperative intensive care unit in Iran. J Pak Med Assoc. 2013;63(1):55-9.
731	Rooh ul M, Kamran K, Khalil J, Gul T, Farid S. Laparoscopic treatment of hepatic hydatid cyst. Journal of the College of Physicians and Surgeons Pakistan. 2011;21(8):468-71.
732	Rouf S, Sharmin S, Dewan F, Akhter S. Relaparotomy after cesarean section: Experience from a tertiary referral and teaching hospital of Bangladesh. Bangladesh Journal of Obstetrics and Gynecology. 2009;24(1):3-9.
733	Rukewe A, Fatiregun A, Adebayo K. Anaesthesia for caesarean deliveries and maternal complications in a Nigerian teaching hospital. Afr J Med Med Sci. 2014;43(1):5-10.
734	Sa MP, Sa MV, Albuquerque AC, Silva BB, Siqueira JW, Brito PR, et al. GuaragnaSCORE satisfactorily predicts outcomes in heart valve surgery in a Brazilian hospital. Rev Bras Cir Cardiovasc. 2012;27(1):1-6.
735	Sá MPBdO, Nogueira JRC, Ferraz PE, Figueiredo OJ, Cavalcante WCP, Cavalcante TCP, et al. Risk factors for low cardiac output syndrome after coronary artery bypass grafting surgery: Fatores de risco para síndrome de baixo débito cardíaco após cirurgia de revascularização miocárdica. Rev bras cir cardiovasc. 2012;27(2):217-23.
736	Saaiq M, Niaz ud D, Zubair M, Shah SA. Presentation and outcome of surgically managed liver trauma: experience at a tertiary care teaching hospital. J Pak Med Assoc. 2013;63(4):436-9.
737	Saaiq M, Shah SA, Zubair M. Abdominal tuberculosis: epidemiologic profile and management experience of 233 cases. J Pak Med Assoc. 2012;62(7):704-7.
738	Sabzi F, Moradi GR, Dadkhah H, Poormotaabed A, Dabiri S. Low dose aprotinin increases mortality and morbidity in coronary artery bypass surgery. J Res Med Sci. 2012;17(1):74-82.
739	Sadaf N, Haq G, Shukar-ud-Din S. Maternal and foetal outcome in HELLP syndrome at tertiary care hospital. J Pak Med Assoc. 2013;63(12):1500-3.
740	Sadaka M, ElSharkawy E, Soliman M, A.N EL-D, El-Hay MAA. Study of infective endocarditis in Alexandria main university hospitals. Egyptian Heart Journal. 2013;65(4):307-17.
741	Sadeck LSR, Leone CR, Procianoy RS, Guinsburg R, Marba STM, Martinez FE, et al. Effects of therapeutic approach on the neonatal evolution of very low birth weight infants with patent ductus arteriosus : Efeitos da abordagem terapeutica da persistencia de canal arterial sobre a evoluciao neonatal de recem-nascidos de extremo baixo peso J Pediatr (Rio J). 2014;90(6):616-23.
742	Sadeghi MM, Arasteh M, Gharipour M, Nilfroush P, Shamsolketabi H, Etesampour A, et al. Evaluation of accuracy of Euroscore risk model in prediction of perioperative mortality after Coronary Bypass Graft Surgery in Isfahan, Iran. J Res Med Sci. 2011;16(6):787-92.
743	Sadrizadeh A, Haghi S, Masuom SH, Bagheri R, Dalouee M. Evaluation of the effect of pulmonary hydatid cyst location on the surgical technique approaches. Lung India. 2014;31(4):361-5.
744	Saeed F, Khalid R, Khan A, Masheer S, Rizvi JH. Peripartum hysterectomy: A ten-year experience at a tertiary care hospital in a developing country. Trop Doct. 2010;40(1):18-21.
745	Safari S, Najafi I, Hosseini M, Sanadgol H, Sharifi A, Moghadam MA, et al. Outcomes of fasciotomy in patients with crush-induced acute kidney injury after Bam earthquake. Iran J Kidney Dis. 2011;5(1):25-8.
746	Sah BK, Chen MM, Yan M, Zhu ZG. Reoperation for early postoperative complications after gastric cancer surgery in a Chinese hospital. World J Gastroenterol. 2010;16(1):98-103.
747	Saha L, Chowdhury SB. Study on primary cesarean section. Mymensingh Med J. 2011;20(2):292-7.
748	Saha N, Saha DK, Rahman MA, Islam MK, Aziz MA. Comparison of post operative morbidity between laparoscopic and open appendectomy in children. Mymensingh Med J. 2010;19(3):348-52.
749	Sahin S, Guzin K, Eroglu M, Kayabasoglu F, Yasartekin MS. Emergency peripartum hysterectomy: our 12-year experience. Arch Gynecol Obstet. 2014;289(5):953-8.
750	Sai K, Mou YG, Zeng J, Lv YC, Xi SY, Guan S, et al. Neurosurgical interventions for patients with nasopharyngeal carcinoma: A single institution experience. World J Surg Oncol. 2013;11.
751	Saidi H, Mutisto BK. Motorcycle injuries at a tertiary referral hospital in Kenya: injury patterns and outcome. Eur J Trauma Emerg Surg. 2013;39(5):481-5.
752	Salahuddin O, Malik MAN, Sajid MA, Azhar M, Dilawar O, Salahuddin A. Acute appendicitis in the elderly; Pakistan Ordnance Factories hospital, Wah Cantt. experience. J Pak Med Assoc. 2012;62(9):946-9.
753	Salama IA, Shoreem HA, Saleh SM, Hegazy O, Housseni M, Abbasy M, et al. Iatrogenic Biliary Injuries: Multidisciplinary Management in a Major Tertiary Referral Center. HPB Surg. 2014;2014:575136.
754	Salehi R, Parvizi R, Ansarin K, Imani S, Goldust M. Surgical embolectomy in treating acute massive pulmonary embolism. J Pak Med Assoc. 2013;63(8):969-72.
755	Salehi SH, Fatemi MJ, Asadi K, Shoar S, Ghazarian AD, Samimi R. Electrical injury in construction workers: A special focus on injury with electrical power. Burns. 2014;40(2):300-4.
756	Saluja SS, Nayeem M, Sharma BC, Bora G, Mishra PK. Management of choledochal cysts and their complications. Am Surg. 2012;78(3):284-90.
757	Samuel JC, Akinkuotu A, Msiska N, Cairns BA, Muyco AP, Charles AG. Re-examining treatment strategies for sigmoid volvulus: An analysis of treatment and outcomes in Lilongwe, Malawi. Glob j Surg. 2010;1(2):149-53.
758	Samuel JC, Qureshi JS, Mulima G, Shores CG, Cairns BA, Charles AG. An Observational Study of the Etiology, clinical presentation and outcomes associated with peritonitis in Lilongwe, Malawi. World J Emerg Surg. 2011;6(1):37.
759	Sangkomkamhang U, Pattanittum P, Laopaiboon M, Lumbiganon P. Mode of delivery and outcomes in preterm births. J Med Assoc Thai. 2011;94(4):415-20.
760	Sani R, Nameoua B, Yahaya A, Hassane I, Adamou R, Hsia RY, et al. The impact of launching surgery at the district level in Niger. World J Surg. 2009;33(10):2063-8.
761	Santo MA, Domene CE, Riccioppo D, Barreira L, Takeda FR, Pinotti HW. Common bile duct stones: analysis of the videolaparoscopic surgical treatment: Coledocolitíase: análise do tratamento videolaparoscópico. Arq gastroenterol. 2012;49(1):41-51.
762	Santos AAd, Sousa AG, Thomé HOdS, Machado RL, Piotto RF. Impact on early and late mortality after blood transfusion in coronary artery bypass graft surgery: Impacto na mortalidade precoce e tardia após transfusão de hemácias em cirurgia de revascularização miocárdica. Rev bras cir cardiovasc. 2013;28(1):1-9.
763	Santos ACd, Martins LLT, Brasil AMS, Pinto SA, Gabriel Neto S, Oliveira ECd. Emergency surgery for complicated colorectal cancer in central Brazil: Cirurgia de emergência para o câncer colorretal complicado no Brasil central. J coloproctol (Rio J, Impr). 2014;34(2):104-8.
764	Santos HNd, Magedanz EH, Guaragna JCVdC, Santos NNd, Albuquerque LC, Goldani MA, et al. Predictors of stroke in patients undergoing cardiac surgery: Preditores de acidente vascular cerebral em pacientes submetidos à cirurgia cardíaca. Rev bras cir cardiovasc. 2014;29(2):140-7.
765	Santos TdS, Melo AR, Moraes HHAd, Almeida Júnior P, Dourado E. Impacted foreign bodies in orbital region: review of nine cases: Corpo estranho impactado na região orbital: revisão de nove casos. Arq bras oftalmol. 2010;73(5):438-42.
766	Hayashi LY, Gazzotti MR, Vidotto MC, Jardim JR. Incidence, indication and complications of postoperative reintubation after elective intracranial surgery: Incidencia, indicacao e complicacoes da reintubacao no pos-operatorio de cirurgia eletiva intracraniana. São Paulo med j. 2013;131(3):158-65.
767	Saramma PP, Girish Menon R, Srivastava A, Sarma P. Hyponatremia after aneurysmal subarachnoid hemorrhage: Implications and outcomes. J Neurosci Rural Pract. 2013;4(1):24-8.
768	Saramma PP, Krishnakumar K, Sarma PS. Alcohol-based hand rub and surgical site infection after elective neurosurgery: An intervention. Neurol India. 2011;59(1):12-7.
769	Sardenberg RAdS, Figueiredo LPd, Haddad FJ, Gross JL, Younes RN. Pulmonary metastasectomy from soft tissue sarcomas. Clinics. 2010;65(9):871-6.
770	Sarioglu T, Yalcinbas YK, Erek E, Sarioglu A. Challenges in the Management of Patients With Functionally Univentricular Heart in Turkey. World Journal for Pediatric and Congenital Hearth Surgery. 2012;3(3):344-9.
771	Satomi E, Sitta MdC, Machado AN, Leme LEG. Identification and treatment of osteoporosis among elderly patients with hip fractures. Clinics. 2009;64(12):1201-4.
772	Saula PW, Hadley GP. Hypertrophic pyloric stenosis in the Third World. Trop Doct. 2011;41(4):204-10.

Reference Number	Full reference
773	Savlovschi C, Serban D, Trotea T, Borcan R, Dumitrescu D. Post-surgery morbidity and mortality in colorectal cancer in elderly subjects. Chirurgia (Bucur). 2013;108(2):177-9.
774	Sayed S, Fischer S, Karck M, Hassouna A, Haverich A. Effect of different preoperative patient characteristics on coronary surgery outcome: a comparative study between a developing and a developed country. J Card Surg. 2009;24(3):275-80.
775	Sayyah-Melli M, Zonoozi GK, Hashemzadeh S, Esfahani A, Ouladehsahebmadarek E, Shobeiry MJ, et al. Comparison of platinum-based neoadjuvant chemotherapy and primary debulking surgery in patients with advanced ovarian cancer. Journal of Obstetrics and Gynecology of India. 2013;63(6):405-9.
776	Scott C, Antoine C, Scarlett M, Irvine R. The provision of surgical care for children with cardiac disease: The Jamaican experience - An 18 year review. West Indian Med J. 2012;61(4).
777	Seal SL, Kamilya G, Mukherji J, Bhattacharyya SK, De A, Hazra A. Outcome in second- versus first-stage cesarean delivery in a teaching institution in eastern India. Am J Perinatol. 2010;27(6):507-12.
778	Secchi MA, Pettinari R, Mercapide C, Bracco R, Castilla C, Cassone E, et al. Surgical management of liver hydatidosis: a multicentre series of 1412 patients. Liver Int. 2010;30(1):85-93.
779	Sehitogullari A, Bilici S, Sayir F, Cobanoglu U, Kahraman A. A long-term study assessing the factors influencing survival and morbidity in the surgical management of bronchiectasis. J Cardiothorac Surg. 2011;6:161.
780	Sekabira J, Hadley GP. Gastroschisis: a third world perspective. Pediatr Surg Int. 2009;25(4):327-9.
781	Sekirime WK, Lule JC. Outcome of cesarean section in asymptomatic HIV-1 infection in Kampala, Uganda. J Obstet Gynaecol Res. 2009;35(4):679-88.
782	Sen Gupta TK, Jha JK, Biswas RS, Chattopadhyay SD, Gupta NK, Kumar S, et al. Emergency management of sigmoid volvulus-institutional experience over four years. J Indian Med Assoc. 2011;109(10):714-6.
783	Sen I, Stephen E, Agarwal S. Clinical profile of aortoiliac occlusive disease and outcomes of aortobifemoral bypass in India. J Vasc Surg. 2013;57(2 Suppl):20s-5s.
784	Sengun IS, Sanli A, Ozalevli S, Onen A, Itil BO, Tasdogen A, et al. Results of diaphragm pacing application in amyotrophic lateral sclerosis patients. First Turkish experience. Journal of Neurological Sciences. 2013;30(2):305-13.
785	Serrano Júnior CV, Ramires JAF, Soeiro AdM, César LAM, Hueb WA, Dallan LA, et al. Efficacy of aneurysmectomy in patients with severe left ventricular dysfunction: favorable short-and long-term results in ischemic cardiomyopathy. Clinics. 2010;65(10):947-52.
786	Shahabuddin S, Ansari JA, Siddiqui FJ, Amanullah M, Sami SA. Redo coronary artery surgery; early and intermediate outcomes from a tertiary care hospital in a developing country. J Pak Med Assoc. 2011;61(1):31-5.
787	Shaikh AR, Muneer A, Laghari ZH. Changing practice of rectal cancer surgery in Pakistan. Pakistan Journal of Medical Sciences. 2010;26(3):601-6.
788	Shaikh AR, Muneer A, Shaikh AA. Laparoscopic surgery for rectal cancer - an early experience from Pakistan. Medical Channel. 2014;20(1):50-3.
789	Shaikh AR, Rao AM, Muneer A. Inguinal mesh hernioplasty under local anaesthesia. J Pak Med Assoc. 2012;62(6):566-9.
790	Shaikh AR, Sangrasi AK, Shaikh GA. Clinical outcomes of laparoscopic versus open appendectomy. JSLS. 2009;13(4):574-80.
791	Shaikh GS, Shaikh S, Baloch I. Traumatic duodenal injuries & surgical management at tertiary care hospital chandka medical college hospital Larkana. Medical Channel. 2011;17(1):19-23.
792	Shaikh NB, Shaikh S, Shaikh JM. Morbidity and mortality associated with obstetric hysterectomy. J Ayub Med Coll Abbottabad. 2010;22(2):100-4.
793	Shamim MS, Ali SF, Enam SA. Non-operative management is superior to surgical stabilization in spine injury patients with complete neurological deficits: A perspective study from a developing world country, Pakistan. Surg Neurol Int. 2011;2:166.
794	Sheikh MR, Khan MS, Saeed Z, Furnaz S, Sharif H. Outcome of coronary artery bypass grafting in a tertiary-care center in Pakistan. Asian Cardiovasc Thorac Ann. 2014.
795	Sheng QF, Lv ZB, Xiao XM. Re-operation for Hirschsprung's disease: experience in 24 patients from China. Pediatr Surg Int. 2012;28(5):501-6.
796	Shetty GS, Bodhankar YD, Ingle S, Thakkar RG, Goel M, Shukla PJ, et al. Complications as indicators of quality assurance after 401 consecutive colorectal cancer resections: the importance of surgeon volume in developing colorectal cancer units in India. World J Surg Oncol. 2012;10:15.
797	Shetty VH, Gowda S, Muralidhar L. Role of Ultrasonography in Diagnosis of Ectopic Pregnancy with Clinical Analysis and Management in Tertiary Care Hospital. The Journal of Obstetrics and Gynecology of India. 2014.
798	Shi JH, Meng X, Han J, Li Y, Wang JG, Zhang HB, et al. A mortality risk assessment model for cardiac valve replacement surgery and its application in the use of prophylactic extracorporeal membrane oxygenation. Int Surg. 2010;95(3):227-31.
799	Shi Y, Su Z, Li L, Liu H, Jing C. Comparing the effects of Bassini versus tension-free hernioplasty: 3 years' follow-up. Front Med China. 2010;4(4):463-8.
800	Shiekh KA, Baba AA, Ahmad SM, Shera AH, Patnaik R, Sherwani AY. Mechanical small bowel obstruction in children at a tertiary care centre in Kashmir. Afr J Paediatr Surg. 2010;7(2):81-5.
801	Shrestha A, Joshi RM, Thapa A, Devkota UP, Gongal DN. Outcome of head injury patients undergoing surgical management: a tertiary level experience. Kathmandu Univ Med J (KUMJ). 2011;9(36):283-5.
802	Shrikhande SV, Barreto SG, Somashekar BA, Suradkar K, Shetty GS, Talole S, et al. Evolution of pancreatoduodenectomy in a tertiary cancer center in India: improved results from service reconfiguration. Pancreatology. 2013;13(1):63-71.
803	Shu Q, Shi Z, Xu WZ, Li JH, Zhang ZW, Lin R, et al. Experience in minimally invasive Nuss operation for 406 children with pectus excavatum. World J Pediatr. 2011;7(3):257-61.
804	Shu'aibu S, Liman H, Akpayak I, Ofoha C, Ramyil V, Dakum N. PRELIMINARY EXPERIENCE WITH RADICAL CYSTECTOMY AND W-ILEAL POUCH FOR MUSCLE INVASIVE TRANSITIONAL CELL BLADDER CARCINOMA. J West Afr Coll Surg. 2012;2(1):25-37.
805	Shukla PJ, Barreto SG, Bedi M, Bheerappa N, Chaudhary A, Gandhi M, et al. Peri-operative outcomes for pancreatoduodenectomy in India: a multi-centric study. HPB (Oxford). 2009;11(8):638-44.
806	Shyam DC, Rapsang AG. Inguinal hernias in patients of 50 years and above. Pattern and outcome: Avaliação do tratamento da hérnia inguinal em pacientes com mais de 50 anos de idade. Rev Col Bras Cir. 2013;40(5):374-9.
807	Sibanda M, Sibanda E, Jonsson K. A prospective evaluation of lower extremity ulcers in a Zimbabwean population. Int Wound J. 2009;6(5):361-6.
808	Siddappa S, Ramprasad K, Muddegowda MK. Xanthogranulomatous pyelonephritis: A retrospective review of 16 cases. Korean Journal of Urology. 2011;52(6):421-4.
809	Siddiqui MM, Paras I, Jalal A. Risk factors of prolonged mechanical ventilation following open heart surgery: what has changed over the last decade? Cardiovasc Diagn Ther. 2012;2(3):192-9.
810	Siddiqui MT, Hasan A, Mohsin S, Hamid M, Amanullah MM. Contegra valved conduit in the paediatric population: An exciting prospect for right ventricle to pulmonary artery reconstruction; experience and outcomes at Aga Khan University. J Pak Med Assoc. 2012;62(10):1113-7.
811	Sie Essoh JB, Kodo M, Dje Bi Dje V, Lambin Y. Limb amputations in adults in an Ivorian teaching hospital. Niger J Clin Pract. 2009;12(3):245-7.
812	Silva ARe, Herdy GVH, Vieira AA, Simões LC. Plastia mitral cirúrgica em crianças com febre reumática: Plastia mitral quirúrgica en niños con fiebre reumática^ies: Surgical mitral valve repair in children with rheumatic fever. Arq bras cardiol. 2009;92(6):433-8.
813	Silva KP, Rocha LA, Leslie AT, Guinsburg R, Silva CM, Nardozza LM, et al. Newborns with congenital heart diseases: epidemiological data from a single reference center in Brazil. J Prenat Med. 2014;8(1-2):11-6.
814	Silva LdFd, Silva JPd, Turquetto ALR, Franchi SM, Cascudo CM, Castro RM, et al. Horizontal right axillary minithoracotomy: aesthetic and effective option for atrial and ventricular septal defect repair in infants and toddlers: Minitoracotomia axilar direita horizontal: opção estética e eficaz para correção de comunicação interventricular e interatrial em lactentes e crianças. Rev bras cir cardiovasc. 2014;29(2):123-30.
815	Silveira Filho LM, Petrucci O, Vilarinho KAdS, Baker RS, Garcia F, Oliveira PPMd, et al. A bovine pericardium rigid prosthesis for left ventricle restoration: 12 years of follow-up: Prótese rígida de pericárdio bovino para remodelamento ventricular esquerdo: 12 anos de seguimento. Rev bras cir cardiovasc. 2011;26(2):164-72.
816	Sincos IR, Aun R, Belczak SQ, Nascimento LD, Mioto Netto B, Casella I, et al. Endovascular and open repair for blunt aortic injury, treated in one clinical institution in Brazil: a case series. Clinics. 2011;66(2):267-74.

Reference Number	Full reference
817	Singh GB, Rai AK, Singh S, Sahu R, Arora R. Management of otogenic lateral sinus thrombosis. Auris Nasus Larynx. 2014;41(2):143-7.
818	Singh H, Mishra A, Sharma D, Somashekar U. A simple prognostic scoring system for typhoid ileal perforation peritonitis. Trop Doct. 2010;40(4):203-7.
819	Singh R, Kumar N, Bhattacharya A, Vajifdar H. Preoperative predictors of mortality in adult patients with perforation peritonitis. Indian J Crit Care Med. 2011;15(3):157-63.
820	Singh SP, Singh U, Singh CP, Chaturvedi J, Shekhar C. Laparoscopic cholecystectomy v/s open cholecystectomy: A comparative study at LLRM Medical College & Hospital, Meerut. Indian Journal of Public Health Research and Development. 2012;3(1):72-4.
821	Singh TG, Ghalige HS, Karthik K, Abhilash S, Ranita Devi S, Singh M, et al. Primary supratentorial haemorrhage null Surgery or no surgery in an Indian setup. Journal of Clinical and Diagnostic Research. 2014;8(9):NC01-NC3.
822	Singh V, Sinha RJ, Mehrotra S, Sankhwar SN, Bhatt S. Repair of vesicovaginal fistula by the transabdominal route: Outcome at a north Indian tertiary hospital. Int Urogynecol J Pelvic Floor Dysfunct. 2012;23(4):411-6.
823	Siqueira Júnior TM, Mitre AI, Duarte RJ, Nascimento H, Barreto F, Falcao E, et al. Transperitoneal versus extraperitoneal laparoscopic radical prostatectomy during the learning curve: does the surgical approach affect the complication rate? Int braz j urol. 2010;36(4):450-7.
824	Smaniotto B, Bahten LCV, Nogueira Filho DC, Tano AL, Thomaz Junior L, Fayad O. Trauma hepático: análise do tratamento com balão intra-hepático em um hospital universitário de Curitiba: Hepatic trauma: analysis of the treatment with intrahepatic balloon in a university hospital of Curitiba. Rev Col Bras Cir. 2009;36(3):217-22.
825	Soares RR, Ferber L, Lorentz MN, Soldati MT. Reposição volêmica intraoperatória: cristaloides versus coloides em revascularização cirúrgica do miocárdio sem circulação extracorpórea: Reposición volémica intraoperatoria: cristaloides versus coloides en revascularización quirúrgica del miocardio sin circulación extracorpórea^ies. Rev bras anestesiol. 2009;59(4):439-51.
826	Sodhi HBS, Savardekar AR, Mohindra S, Chhabra R, Gupta V, Gupta SK. The clinical profile, management, and overall outcome of aneurysmal subarachnoid hemorrhage at the neurosurgical unit of a tertiary care center in India. J Neurosci Rural Pract. 2014;5(2):118-26.
827	Sohrab N, Alireza Y, Ata M, Mahmoud S, Bahram Q, Azad R, et al. Prevalence and risk factors of mortality after surgery for congenital heart disease in Tabriz, Iran: A five year retrospective analysis. Pakistan Journal of Medical Sciences. 2010;26(2):267-70.
828	Sohrabi B, Yaghoubi AR, Ghaffari S. The impact of diabetes on early and midterm outcome of patients undergoing coronary artery bypass grafting surgery. Iranian Cardiovascular Research Journal. 2010;4(2):66-9.
829	Sokouti M, Montazeri V. A comprehensive study of mediastinal goiters. Tanaffos. 2010;9(1):15-20.
830	Solmaz I, Kural C, Temiz C, Secer HI, Duz B, Gonul E, et al. Traumatic brain injury due to gunshot wounds: a single institution's experience with 442 consecutive patients. Turk Neurosurg. 2009;19(3):216-23.
831	Sonshine DB, Shantz J, Kumah-Ametepey R, Coughlin RR, Gosselin RA. The Implementation of a Pilot Femur Fracture Registry at Komfo Anokye Teaching Hospital: An Analysis of Data Quality and Barriers to Collaborative Capacity-Building. World J Surg. 2013;37(7):1506-12.
832	Sorbye IK, Vangen S, Oneko O, Sundby J, Bergsjo P. Caesarean section among referred and self-referred birthing women: a cohort study from a tertiary hospital, northeastern Tanzania. BMC Pregnancy Childbirth. 2011;11:55.
833	Sorour MA, Kassem MI, Ghazal AH, Azzam A, El-Khashab ESI, Shehata GM. Conservative approach in the management of isolated penetrating liver trauma. Alexandria Journal of Medicine. 2013;49(2):125-32.
834	Souza JP, Gulmezoglu A, Lumbiganon P, Laopaiboon M, Carroli G, Fawole B, et al. Caesarean section without medical indications is associated with an increased risk of adverse short-term maternal outcomes: the 2004-2008 WHO Global Survey on Maternal and Perinatal Health. BMC Med. 2010;8:71.
835	Srinath S, Naveen HS, Manjunath. Risk prediction of morbidity and mortality in emergency laparotomy by possum equation. Research Journal of Pharmaceutical, Biological and Chemical Sciences. 2013;4(4):206-16.
836	Sriussadaporn S, Sriussadaporn S, Pak-Art R, Kritayakirana K, Prichayudh S, Samorn P. Lessons learned from 100 personal consecutive cases of pancreaticoduodenectomy at a university hospital in Thailand. J Med Assoc Thai. 2013;96(9):1147-58.
837	Stanzani F, Paisani DdM, Oliveira Ad, Souza RCd, Perfeito JAJ, Faresin SM. Morbidity, mortality, and categorization of the risk of perioperative complications in lung cancer patients: Mortalidade, morbidade e categorização de risco para complicações perioperatórias em pacientes com câncer de pulmão. J bras pneumol. 2014;40(1):21-9.
838	Suciu B, Bud V, Copotoiu C, Branzaniuc K, Copotoiu R, Fodor D, et al. Factors affecting early morbidity and mortality in non-small cell lung cancer surgery, the experience of Surgical Clinic No. 1, Tg. Mures. Rev Med Chir Soc Med Nat Iasi. 2011;115(1):116-26.
839	Suksompong S, Thamtanavit S, von Bormann B, Thongcharoen P. Thoracic surgery mortality and morbidity in a university hospital. Asian Cardiovasc Thorac Ann. 2012;20(2):182-7.
840	Sule AZ, Ajibade A. Adult large bowel obstruction: a review of clinical experience. Ann Afr Med. 2011;10(1):45-50.
841	Sumer A, Kemik O, Dulger AC, Olmez A, Hasirci I, Kisli E, et al. Outcome of surgical treatment of intestinal perforation in typhoid fever. World J Gastroenterol. 2010;16(33):4164-8.
842	Sun H, Liu H, Li D, Liu L, Yang J, Wang W. An effective treatment for cerebral hemorrhage: Minimally invasive craniopuncture combined with urokinase infusion therapy. Neurol Res. 2010;32(4):371-7.
843	Surapaneni S, S R, Reddy AV. The Perforation-Operation time Interval; An Important Mortality Indicator in Peptic Ulcer Perforation. J Clin Diagn Res. 2013;7(5):880-2.
844	Suwannarurk K, Thaweekul Y, Mairaing K, Poomtavorn Y, Bhamarapravatana K. Silent abnormal placentation linkage to peripartum hysterectomy: Thammasat University Hospital 6-year study. J Med Assoc Thai. 2014;97(5):473-7.
845	Swende TZ, Akinbuwa BA. Minilaparotomy female sterilisation at a Nigerian tertiary health centre. Internet Journal of Gynecology and Obstetrics. 2010;13(1).
846	Talpur AA, Awan MS, Hashmi F, Jamal A. Evaluation & management of patients with liver trauma. Medical Channel. 2013;19(3):39-43.
847	Talpur NN, Memon SR, Jamro B, Korejo R. Maternal and fetal morbidity with abruptio placentae. Rawal Medical Journal. 2011;36(4).
848	Talukder MM, Islam KM, Hossain M, Jahan MU, Mahmood E, Hossain SS. Surgery for primary intracerebral haemorrhage: is it safe and effective? Bangladesh Med Res Counc Bull. 2012;38(3):74-8.
849	Talwar S, Meena A, Choudhary SK, Kothari SS, Gupta SK, Saxena A, et al. Anomalous branch of pulmonary artery from the aorta and tetralogy of Fallot: morphology, surgical techniques and results. Eur J Cardiothorac Surg. 2014;46(2):291-6.
850	Tamdee D, Charuluxananan S, Punjasawadwong Y, Tawichasri C, Kyokong O, Patumanond J, et al. Factors related to 24-hour perioperative cardiac arrest in geriatric patients in a Thai university hospital. J Med Assoc Thai. 2009;92(2):198-207.
851	Tamiru T, Gray PE, Pollock JD. An alternative method of management of pediatric airway foreign bodies in the absence of rigid bronchoscopy. Int J Pediatr Otorhinolaryngol. 2013;77(4):480-2.
852	Tan J, Tan H, Hu B, Ke C, Ding X, Chen F, et al. Short-term outcomes from a multicenter retrospective study in China comparing laparoscopic and open surgery for the treatment of infected pancreatic necrosis. J Laparoendosc Adv Surg Tech A. 2012;22(1):27-33.
853	Tan WM, Adnan JS, Mohamad Haspani MS. Treatment outcome of superficial cerebral abscess: An analysis of two surgical methods. Malays J Med Sci. 2010;17(1):23-9.
854	Tannuri ACA, Sbragia L, Tannuri U, Silva LM, Leal AJG, Schmidt AFS, et al. Evolution of critically ill patients with gastroschisis from three tertiary centers. Clinics. 2011;66(1):17-20.
855	Tannuri ACA, Silva LM, Leal AJG, Moraes ACFd, Tannuri U. Does administering albumin to postoperative gastroschisis patients improve outcome? Clinics. 2012;67(2):107-11.
856	Tapisiz OL, Altinbas SK, Yirci B, Cenksoy P, Kaya AE, Dede S, et al. Emergency peripartum hysterectomy in a tertiary hospital in Ankara, Turkey: a 5-year review. Arch Gynecol Obstet. 2012;286(5):1131-4.

Reference Number	Full reference
857	Tarca E, Aprodu SG. Gastroschisis treatment: what are the causes of high morbidity and mortality rates? Chirurgia (Bucur). 2013;108(4):516-20.
858	Tariq M, Siddiqui BK, Jadoon A, Alam M, Khan SA, Atiq M, et al. Clinical profile and outcome of infective endocarditis at the Aga Khan University hospital. International Journal of Collaborative Research on Internal Medicine and Public Health. 2009;1(3):84-99.
859	Taslak Sengul A, Bilgin Buyukkarabacak Y, Durgun Yetim T, Pirzirenli MG, Celik B, Basoglu A. Early diagnosis saves lives in esophageal perforations. Turkish Journal of Medical Sciences. 2013;43(6):939-45.
860	Tasoglu I, Tutun U, Lafci G, Hijaazi A, Yener U, Ulus AYT, et al. Primary Cardiac Myxomas: Clinical Experience and Surgical Results in 67 Patients. J Card Surg. 2009;24(3):256-9.
861	Tavcar I, Kikovic S, Bezmarevic M, Rusovic S, Perisic N, Mirkovic D, et al. A 60-year expirience in the treatment of pancreatic insulinoma in the Military Medical Academy, Belgrade, Serbia. Vojnosanit Pregl. 2014;71(3):293-7.
862	Tayyab M, Aurangzeb M, Ahmad N, Saeed Q. Fournier's gangrene: A review of 15 cases. Journal of Postgraduate Medical Institute. 2010;24(2):138-41.
863	Tebeu PM, Ndive PE, Ako WT, Biyaga PT, Fomulu JN, Doh AS. Emergency obstetric hysterectomy at University Hospital, Yaounde, Cameroon. Int J Gynaecol Obstet. 2013;120(1):91-2.
864	Tedde ML, Campos JRMd, Das-Neves-Pereira J-C, Abrao FC, Jatene FB. The search for stability: bar displacement in three series of pectus excavatum patients treated with the Nuss technique. Clinics. 2011;66(10):1743-6.
865	Teerapong S, Rungaramsin P, Tanprasertkul C, Bhamarapravatana K, Suwannarurk K. Major complication of gynaecological laparoscopy in Police General Hospital: a 4-year experience. J Med Assoc Thai. 2012;95(11):1378-83.
866	Teicher CL, Alberti K, Porten K, Elder G, Baron E, Herard P. Medecins sans frontieres experience in orthopedic surgery in postearthquake Haiti in 2010. Prehosp Disaster Med. 2014;29(1):21-6.
867	Teixeira MJ, Fonoff ET, Mandel M, Alves HL, Rosemberg S. Stereotactic biopsies of brain lesions: Biópsia estereotáxica de lesões encefálicas. Arq neuropsiquiatr. 2009;67(1):74-7.
868	Tekumit H, Cenal AR, Tataroglu C, Uzun K, Polat A, Akinci E. Cusp shaving for concomitant mild to moderate rheumatic aortic insufficiency. J Card Surg. 2010;25(1):16-22.
869	Tennant IA, Augier R, Crawford-Sykes A, Hambleton IR, Tha M, Harding H. Anaesthetic morbidity at the University Hospital of the West Indies: Morbilidad anestésica en el Hospital Universitario de West Indies: un estudio transversal prospectivo^ies. West Indian med j. 2009;58(5):452-9.
870	Tenório EM, Moraes Neto F, Chauvaud S, Moraes CRRd. Experiência com a técnica de ampliação do folheto posterior para correção da insuficiência mitral reumática na infância: Experience with the posterior leaflet extension technique for correction of rheumatic mitral insufficiency in children. Rev bras cir cardiovasc. 2009;24(4):567-9.
871	Terra RM, Minamoto H, Mariano LC, Fernandez A, Otoch JP, Jatene FB. Surgical treatment of congenital tracheal stenoses. J Bras Pneumol. 2009;35(6):515-20.
872	Terra RM, Waisberg DR, Almeida JLJd, Devido MS, Pêgo-Fernandes PM, Jatene FB. Does videothoracoscopy improve clinical outcomes when implemented as part of a pleural empyema treatment algorithm? Clinics. 2012;67(6):557-64.
873	Terzi A, Yildiz F, Vural M, Coban S, Cece H, Kaya M. A case series of 46 appendectomies during pregnancy. Wien Klin Wochenschr. 2010;122(23-24):686-90.
874	Thakur B, Hui L, Devkota M, Xin C, Lama R. Minimally invasive esophagectomy/gastroesophagectomy for cancer. is it safe in nepalese context? Journal of Cancer Science and Therapy. 2012;4(4):102-5.
875	Thakur B, Li H, Devkota M. Results of management of esophageal and GE junction malignancies in Nepalese context. J Thorac Dis. 2013;5(2):123-8.
876	Thapa A, Chandra PS, Sinha S, Gupta A, Singh M, Suri A, et al. Surgical interventions in intracranial arteriovenous malformations: Indications and outcome analysis in a changing scenario. Neurol India. 2009;57(6):749-55.
877	Thomas G, Richards FO, Jr., Eigege A, Dakum NK, Azzuwut MP, Sarki J, et al. A pilot program of mass surgery weeks for treatment of hydrocele due to lymphatic filariasis in central Nigeria. Am J Trop Med Hyg. 2009;80(3):447-51.
878	Tian Y, Wu SD, Su Y, Kong J, Yu H, Fan Y. Laparoscopic subtotal cholecystectomy as an alternative procedure designed to prevent bile duct injury: experience of a hospital in northern China. Surg Today. 2009;39(6):510-3.
879	Tian ZQ, Su XF, Yu F, Liu H, Fang Z, Luo KL. A 10-year experience with hepatic trauma in a Chinese level one trauma center. Saudi Med J. 2014;35(9):1150-3.
880	Tinoco RC, Tinoco AC, El-Kadre LJ, Sueth DM, Conde LM. Laparoscopic gastrectomy for gastric cancer. Surg Laparosc Endosc Percutan Tech. 2009;19(5):384-7.
881	Tomiyoshi SDT, Santos CHMDOS. Effectiveness of the ligation of intersphincteric fistula tract (LIFT) in the treatment of anal fistula: initial results: Eficácia da técnica de ligadura interesfincteriana do trato fistuloso (LIFT) no tratamento da fístula anal: resultados iniciais. ABCD arq bras cir dig. 2014;27(2):101-3.
882	Topaloglu U, Dulundu E, Ozkan E, Kayahan M, Ozel Y. Extended lymphadenectomy for gastric cancer: a single center experience in Istanbul. Hepatogastroenterology. 2009;56(89):266-9.
883	Torina AG, Petrucci O, Oliveira PPMd, Severino ESBdO, Vilarinho KAdS, Lavagnoli CFR, et al. Efeitos da ultrafiltração modificada na função pulmonar e necessidade de hemotransfusão em pacientes submetidos à revascularização do miocárdio: The effects of modified ultrafiltration on pulmonary function and transfusion requirements in patients underwent coronary artery bypass graft surgery. Rev bras cir cardiovasc. 2010;25(1):59-65.
884	Traore D, Sanogo ZZ, Bengaly B, Sissoko F, Coulibaly B, Togola B, et al. Acute sigmoid volvulus: results of surgical treatment in the teaching hospitals of Bamako. J Visc Surg. 2014;151(2):97-101.
885	Traore D, Sissoko F, Ongoiba N, Traore I, Traore AK, Koumare AK. Adult intussusception: diagnostic pitfalls, morbidity and mortality in a developing country. J Visc Surg. 2012;149(3):e211-4.
886	Travancas PR, Dorigo AH, Simoes LC, Fonseca SC, Bloch KV, Herdy GV. Comparison of mechanical and biological prostheses when used to replace heart valves in children and adolescents with rheumatic fever. Cardiol Young. 2009;19(2):192-7.
887	Trivedi NP, Trivedi P, Trivedi H, Trivedi S, Trivedi N. Optimizing multimodality treatment for head and neck cancer in rural India. Indian J Cancer. 2012;49(2):225-9.
888	Tudorache S, Chiutu LC, Iliescu DG, Georgescu R, Stoica GA, Simionescu CE, et al. Prenatal diagnosis and perinatal outcome in congenital diaphragmatic hernia. Single tertiary center report. Rom J Morphol Embryol. 2014;55(3):823-33.
889	Tufegdzic M, Panic N, Boccia S, Malerba S, Bulajic M, La Vecchia C, et al. The weekend effect in patients hospitalized for upper gastrointestinal bleeding: a single-center 10-year experience. Eur J Gastroenterol Hepatol. 2014;26(7):715-20.
890	Tumusiime G. Surgical Mortality at a Mission Hospital in Western Uganda. East and Central African Journal of Surgery. 2010;15(2).
891	Tuncalp O, Hindin MJ, Adu-Bonsaffoh K, Adanu RM. Assessment of maternal near-miss and quality of care in a hospital-based study in Accra, Ghana. International Journal of Gynecology and Obstetrics. 2013;123(1):58-63.
892	Tyson AF, Boschini LP, Kiser MM, Samuel JC, Mjuweni SN, Cairns BA, et al. Survival after burn in a sub-Saharan burn unit: challenges and opportunities. Burns. 2013;39(8):1619-25.
893	Tyson AF, Msiska N, Kiser M, Samuel JC, McLean S, Varela C, et al. Delivery of operative pediatric surgical care by physicians and non-physician clinicians in Malawi. Int J Surg. 2014;12(5):509-15.
894	Uche EO, Onyia E, Mezue UC, Okorie E, Ozor II, Chikani MC. Determinants and outcomes of ventriculoperitoneal shunt infections in Enugu, Nigeria. Pediatr Neurosurg. 2014;49(2):75-80.
895	Uday SK, Bhargav PR. SILACIG: A novel technique of single-incision laparoscopic appendicectomy based on institutional experience of 29 cases. J Minim Access Surg. 2013;9(2):76-9.
896	Ugochukwu AI, Amu OC, Nzegwu MA, Dilibe UC. Acute perforated peptic ulcer: on clinical experience in an urban tertiary hospital in south east Nigeria. Int J Surg. 2013;11(3):223-7.
897	Ugochukwu AI, Amu OC, Nzegwu MA. Ileal perforation due to typhoid fever - review of operative management and outcome in an urban centre in Nigeria. Int J Surg. 2013;11(3):218-22.
898	Ugwu EO, Obioha KC, Okezie OA, Ugwu AO. A five-year survey of caesarean delivery at a Nigerian tertiary hospital. Ann Med Health Sci Res. 2011;1(1):77-83.

Reference Number	Full reference
899	Ugwu RO, Okoro PE. Pattern, outcome and challenges of neonatal surgical cases in a tertiary teaching hospital. Afr J Paediatr Surg. 2013;10(3):226-30.
900	Ugwumba FO, Nnabugwu, II, Ozoemena OFN. Fournier's gangrene - analysis of management and outcome in south-eastern Nigeria. S Afr J Surg. 2012;50(1):16-9.
901	Ugwumba FO, Ozoemena OF, Okoh AD, Echetabu KN, Mbadiwe OM. Transvesical prostatectomy in the management of benign prostatic hyperplasia in a developing country. Niger J Clin Pract. 2014;17(6):797-801.
902	Uz Zaman A, Iqbal M, Zaheer F, Abbas Khan R, Ahsan Malik K. Penetrating abdominal injury: A tertiary care hospital experience. Rawal Medical Journal. 2014;39(1):68-71.
903	Uzair M, Ahmad M, Hussain M, Younus M, Khan K. Frequency of urethrocutaneous fistula following snodgrass hypospadias repair in children. Journal of Postgraduate Medical Institute. 2013;27(1):74-7.
904	Valle FH, Costa AR, Pereira EM, Santos EZ, Pivatto Junior F, Bender LP, et al. [Morbidity and mortality in patients aged over 75 years undergoing surgery for aortic valve replacement]. Arq Bras Cardiol. 2010;94(6):720-5.
905	Varcus F, Lazar F, Beuran M, Lica I, Turculet C, Nicolau E, et al. Laparoscopic treatment of perforated duodenal ulcer -- a multicenter study. Chirurgia (Bucur). 2013;108(2):172-6.
906	Vaziri M, Pazooki A, Zahedi-Shoolami L. Mediastinal masses: Review of 105 cases. Acta Med Iran. 2009;47(4):297-300.
907	Veena P, Habeebullah S, Chaturvedula L. A review of 93 cases of ruptured uterus over a period of 2 years in a tertiary care hospital in South India. J Obstet Gynaecol. 2012;32(3):260-3.
908	Velicki L, Nicin S, Mihajlovic B, Kovacevic P, Susak S, Fabri M. Cardiac myxoma: clinical presentation, surgical treatment and outcome. J BUON. 2010;15(1):51-5.
909	Venter JA, le Grange SM, Otto SF, Joubert G. An audit of paediatric intussusception radiological reduction at the Bloemfontein Academic Hospital Complex, Free State, South Africa. SAJCH South African Journal of Child Health. 2013;7(2):60-4.
910	Verma S, Sayana A, Kala S, Rai S. Evaluation of the Utility of the Fournier's Gangrene Severity Index in the Management of Fournier's Gangrene in North India: A Multicentre Retrospective Study. J Cutan Aesthet Surg. 2012;5(4):273-6.
911	Vidotto MC, Sogame LC, Gazzotti MR, Prandini M, Jardim JR. Implications of extubation failure and prolonged mechanical ventilation in the postoperative period following elective intracranial surgery. Braz j med biol res. 2011;44(12):1291-8.
912	Vijarnsorn C, Laohaprasitiporn D, Durongpisitkul K, Chantong P, Soongswang J, Cheungsomprasong P, et al. Surveillance of pediatric cardiac surgical outcome using risk stratifications at a tertiary care center in Thailand. Cardiol Res Pract. 2011;1(1).
913	Vukovic G, Lausevic Z. Diagnostics and treatment of liver injuries in polytrauma. HealthMED. 2012;6(8):2796-801.
914	Vukovic M, Moljevic N, Crnogorac S. Total colectomy in older patients with acute malignant obstruction of the left-sided colons. Journal of Acute Disease. 2013;2(1):44-7.
915	Waldron NR, Kennifer D, Bourgois E, Vanna K, Noor S, Gollogly J. Acid violence in Cambodia: The human, medical and surgical implications. Burns. 2014.
916	Walker IA, Obua AD, Mouton F, Ttendo S, Wilson IH. Paediatric surgery and anaesthesia in south-western Uganda: a cross-sectional survey. Bull World Health Organ. 2010;88(12):897-906.
917	Wang C, Li X, Lu FL, Xu JB, Tang H, Han L, et al. Comparison of six risk scores for in-hospital mortality in Chinese patients undergoing heart valve surgery. Heart Lung Circ. 2013;22(8):612-7.
918	Wang C, Yao F, Han L, Zhu J, Xu ZY. Validation of the European system for cardiac operative risk evaluation (EuroSCORE) in Chinese heart valve surgery patients. J Heart Valve Dis. 2010;19(1):21-7.
919	Wang E, Yi H, Wang M, Huang C. Treatment of osteoporotic vertebral compression fractures with percutaneous kyphoplasty: A report of 196 cases. European Journal of Orthopaedic Surgery and Traumatology. 2013;23(SUPPL. 1):S71-S5.
920	Wang H, Yang J, Zhang X, Yan L, Yang J. Liver resection in hepatitis B-related hepatocellular carcinoma: clinical outcomes and safety in overweight and obese patients. PLoS One. 2014;9(6):e99281.
921	Wang L, Su AP, Zhang Y, Yang M, Yue PJ, Tian BL. Reduction of alkaline reflux gastritis and marginal ulcer by modified Braun enteroenterostomy in gastroenterologic reconstruction after pancreaticoduodenectomy. J Surg Res. 2014;189(1):41-7.
922	Wang QY, Tan LJ, Feng MX, Zhang XY, Zhang L, Jiang NQ, et al. Video-assisted mediastinoscopic resection compared with video-assisted thoracoscopic surgery in patients with esophageal cancer. J Thorac Dis. 2014;6(6):663-7.
923	Wang W, Duan W, Xue Y, Wang L, Liu J, Yu S, et al. Clinical features of acute aortic dissection from the Registry of Aortic Dissection in China. J Thorac Cardiovasc Surg. 2014;148(6):2995-3000.
924	Wang X, Zheng Z, Ao H, Zhang S, Wang Y, Zhang H, et al. A comparison before and after aprotinin was suspended in cardiac surgery: different results in the real world from a single cardiac center in China. J Thorac Cardiovasc Surg. 2009;138(4):897-903.
925	Wang Y, Lu B, Hao P, Yan MN, Dai KR. Comprehensive treatment for gas gangrene of the limbs in earthquakes. Chin Med J. 2013;126(20):3833-9.
926	Wani I, Rather M, Naikoo G, Amin A, Mushtaq S, Nazir M. Intestinal Ascariasis in Children. World J Surg. 2010:1-6.
927	Warf BC, Dagi AR, Kaaya BN, Schiff SJ. Five-year survival and outcome of treatment for postinfectious hydrocephalus in Ugandan infants. J Neurosurg Pediatr. 2011;8(5):502-8.
928	Wasay M, Patel J, Azam I, Khan MA, Smego Jr RA. Preoperative antifungal therapy may improve survival in patients with Aspergillus brain abscess. Clin Neurol Neurosurg. 2009;111(7):565-7.
929	Kayal A, Hussain A. A comprehensive prospective clinical study of hydatid disease. ISRN Gastroenterol. 2014;2014:514757
930	Wei Q, Zhang W, Chen M, Zhang L, He G, Liu X. Peripartum hysterectomy in 38 hospitals in China: a population-based study. Arch Gynecol Obstet. 2014;289(3):549-53.
931	Weiser TG, Haynes AB, Dziekan G, Berry WR, Lipsitz SR, Gawande AA. Effect of a 19-item surgical safety checklist during urgent operations in a global patient population. Ann Surg. 2010;251(5):976-80.
932	Wella HL. Causes and clinical outcomes in neonates with acute abdomen requiring surgery at Muhimbili National Hospital Dar es Salaam. Tanz Med J 2013;26(1):5-7.
933	Williams OM, Osuoji RI, Ajai OT, Olayiwola B, Bankole MA. Intestinal atresia: A four-year review of cases in Ikeja-Lagos. Journal of Nepal Paediatric Society. 2012;32(1):28-32.
934	Winkler AS, Tluway A, Slottje D, Schmutzhard E, Hrtl R. The pattern of neurosurgical disorders in rural northern Tanzania: A prospective hospital-based study. World Neurosurg. 2010;73(4):264-9.
935	Wong EG, Trelles M, Dominguez L, Mupenda Mwania J, Kasonga Tshibangu C, Haq Saqeb S, et al. Operative Procedures in the Elderly in Low-Resource Settings: A Review of Medecins Sans Frontieres Facilities. World J Surg. 2014.
936	Wu AW, Ji JF, Yang H, Li YN, Li SX, Zhang LH, et al. Long-term outcome of a large series of gastric cancer patients in China. Chinese Journal of Cancer Research. 2010;22(3):167-75.
937	Wu X, Yang D, Yang Z, Li J, Zhao Y, Wang K, et al. Clinical characteristics and long term post-operative outcome of cardiac Myxoma. EXCLI Journal. 2012;11:240-9.
938	Wu Z, Hao S, Zhang J, Zhang L, Jia G, Tang J, et al. Foramen magnum meningiomas: experiences in 114 patients at a single institute over 15 years. Surg Neurol. 2009;72(4):376-82.
939	Xu K, Luo Q, Chen X, Yu JL. A selective clipping microsurgical treatment for multiple intracranial anterior circulation aneurysms. Kuwait Med J. 2014;46(1):21-7.
940	Xu Z, Li W, Xu X, Zhou Z, Song S, Ma J, et al. Long-term follow-up with ross procedure at a single institution in China. Thorac Cardiovasc Surg. 2014;62(3):216-21.
941	Yadav D, Garg PK. Spectrum of perforation peritonitis in delhi: 77 cases experience. Indian J Surg. 2013;75(2):133-7.
942	Yadav K, Singh M, Griwan M, Mishra T, Kumar N, Kumar H. Evaluation of POSSUM and P-POSSUM as a tool for prediction of surgical outcomes in the Indian population. Australas Med J. 2011;4(7):366-73.
943	Yalinkaya A, Guzel AI, Kangal K. Emergency peripartum hysterectomy: 16-year experience of a medical hospital. J Chin Med Assoc. 2010;73(7):360-3.

Reference Number	Full reference
944	Yamasmit W, Chaithongwongwatthana S. Risk factors for cesarean hysterectomy in tertiary center in Thailand: a case-control study. J Obstet Gynaecol Res. 2009;35(1):60-5.
945	Yan Z, Liao G, Pei H. Surgical treatment of familial adenomatous polyposis: Experience from a single institution in China. Asia Pac J Clin Oncol. 2012;8(3):e23-e8.
946	Yang Z, Wang L, Kang L, Xiang J, Peng J, Cui J, et al. Clinicopathologic characteristics and outcomes of patients with obstructive colorectal cancer. J Gastrointest Surg. 2011;15(7):1213-22.
947	Yavangi M, Sohrabi MR, Tabriz AA. Effect of Iranian ministry of health protocols on Cesarean section rate: A quasi-experimental study. Journal of Research in Health Sciences. 2013;13(1):48-52.
948	Yeap BH, Zahari Z. Neonatal tumours in Malaysia: a call for heightened awareness. Pediatr Surg Int. 2010;26(2):207-12.
949	Yildirim IO, Salihoglu Z, Bolayirli MI, Colakoglu N, Yuceyar L. Prospective evaluation of the factors effective on morbidity and mortality of the patients having liver resection surgeries. Hepatogastroenterology. 2012;59(118):1928-32.
950	Yin L, Zhang L, Hao S, Zhang J, Wu Z. Medullary hemangioblastoma: 34 patients at a single institution. J Clin Neurosci. 2014;21(2):250-5.
951	Ying F, Shuodong W, Hong Y, Yang S, Jing K, Yu T, et al. Lessons learnt after 12 years experience in laparoscopic cholecystectomy at a single center. Hepatogastroenterology. 2010;57(98):202-6.
952	Younes RN, Gross JL, Taira AM, Martins AAC, Neves GS. Surgical resection of lung metastases: results from 529 patients. Clinics. 2009;64(6):535-41.
953	Yousuf KM, Bhagwani AR, Bilal N. Management of chronic traumatic arteriovenous fistula of the lower extremities. Eur J Trauma Emerg Surg. 2013;39(4):393-6.
954	Yu D, Han Y, Zhou S, Song X, Li Y, Xiao N, et al. Video-assisted thoracic bronchial sleeve lobectomy with bronchoplasty for treatment of lung cancer confined to a single lung lobe: a case series of Chinese patients. J Cardiothorac Surg. 2014;9:67.
955	Yu JH, Guo HW, Zhang G, Wu SM, Song GM, Sun WY. Valve replacement in pediatric patients: a single center experience. Chin Med J (Engl). 2011;124(2):218-22.
956	Yu L, Gu T, Shi E, Wang C, Fang Q, Yu Y, et al. Off-pump versus on-pump coronary artery bypass surgery in patients with triple-vessel disease and enlarged ventricles. Ann Saudi Med. 2014;34(3):222-8.
957	Yu L, Gu TX, Shi EY, Jiang CL. Surgery for chronic total occlusion of the left main coronary artery. Ann Saudi Med. 2012;32(2):156-61.
958	Yuan S-M, Jing H, Lavee J. The implications of serum enzymes and coagulation activities in postinfarction myocardial: As implicações de enzimas séricas e atividades de coagulação em ruptura cardíaca pós-infarto do miocárdio. Rev bras cir cardiovasc. 2011;26(1):7-14.
959	Yusoff AR, Razak MMA, Yoong BK, Vijeyasingam R, Siti ZM. Survival analysis of cholangiocarcinoma: A 10-year experience in Malaysia. World J Gastroenterol. 2012;18(5):458-65.
960	Zafar H, Hameed A, Pardhan A, Murtaza G, Mazahir S. Laparoscopic splenectomy for haematological disorder: our experience. J Pak Med Assoc. 2012;62(10):1096-9.
961	Zaman BSS, J.; Bhatti, S.Z.; Shamas, N. Indications and complication of emergency peripartum hysterectomy in Bahawal Victoria Hospital Bahawalpur. Pak J Med Health Sci. 2013;7:726-9.
962	Zanati SG, Mouraria GG, Matsubara LS, Giannini M, Matsubara BB. Profile of cardiovascular risk factors and mortality in patients with symptomatic peripheral arterial disease. Clinics (Sao Paulo). 2009;64(4):323-6.
963	Zargar M, Laal M. Liver trauma: Operative and non-operative management. International Journal of Collaborative Research on Internal Medicine and Public Health. 2010;2(4):96-107.
964	Zehir S, Sipahioglu S, Ozdemir G, Sahin E, Yar U, Akgul T. Red cell distribution width and mortality in patients with hip fracture treated with partial prosthesis. Acta Orthop Traumatol Turc. 2014;48(2):141-6.
965	Zhang C, Hu L, Wu X, Hu G, Ding X, Lu Y. A retrospective study on the aetiology, management, and outcome of brain abscess in an 11-year, single-centre study from China. BMC Infect Dis. 2014;14:311.
966	Zhang H, Yuan X, Osnabrugge RL, Meng D, Gao H, Zhang S, et al. Influence of diabetes mellitus on long-term clinical and economic outcomes after coronary artery bypass grafting. Ann Thorac Surg. 2014;97(6):2073-9.
967	Zhang M, Kang JP, Nie SP, Lv Q, Liu XH, Ma CS. Pre-hospital statin therapy may not reduce incidence of all-cause mortality and overall MACCE during hospital stay after coronary artery bypass graft surgery. Acta Cardiol. 2009;64(2):253-7.
968	Zhang P, Jiang C, He WX, Song N, Zhou X, Jiang GN. Completion pneumonectomy for lung cancer treatment: early and long term outcomes. J Cardiothorac Surg. 2012;7:5.
969	Zhang P, Jiang G, Ding J, Zhou X, Gao W. Surgical treatment of bronchiectasis: a retrospective analysis of 790 patients. Ann Thorac Surg. 2010;90(1):246-50.
970	Zhang WB, Wang WY, Wang GF, Li N, Li JS. Risk factors of mortality in non-trauma exsanguinating patients that require damage control laparotomy. ANZ J Surg. 2010;80(4):258-64.
971	Zhang Z, Huang Y, Su P, Wang D, Wang L. Experience in treating congenital esophageal atresia in China. J Pediatr Surg. 2010;45(10):2009-14.
972	Zhang ZL, Dong P, Li YH, Liu ZW, Yao K, Han H, et al. Radical cystectomy for bladder cancer: Oncologic outcome in 271 Chinese patients. Chin J Cancer. 2014;33(3):165-71.
973	Zhang ZL, Li YH, Luo JH, Liu ZW, Yao K, Dong P, et al. Complications of radical nephrectomy for renal cell carcinoma: A retrospective study comparing transperitoneal and retroperitoneal approaches using a standardized reporting methodology in two Chinese centers. Chin J Cancer. 2013;32(8):461-8.
974	Zheng Z, Fan H, Gao H, Li X, Yuan X, Meng J, et al. Mortality risk model for heart valve surgery in China. J Heart Valve Dis. 2013;22(1):93-101.
975	Zheng Z, Kun H, Xuezeng X, Yunge C, Zengshan M, Huiming G, et al. Totally thoracoscopic versus open surgery for closure of atrial septal defect: Propensity-score matched comparison. Heart Surg Forum. 2014;17(4):E227-E31.
976	Zheng Z, Zhang L, Hu S, Li X, Yuan X, Gao H. Risk factors and in-hospital mortality in Chinese patients undergoing coronary artery bypass grafting: analysis of a large multi-institutional Chinese database. J Thorac Cardiovasc Surg. 2012;144(2):355-9, 9.e1.
977	Zhong JH, Li H, Xiao N, Ye XP, Ke Y, Wang YY, et al. Hepatic resection is safe and effective for patients with hepatocellular carcinoma and portal hypertension. PLoS One. 2014;9(9).
978	Zhong Y, Zhu D, Liang L, Ye Q, Wei Y, Ren L, et al. The results of surgery for colorectal hepatic metastases following expansion of the indications in 2005. Colorectal Dis. 2013;15(8):e429-34.
979	Zhou X. Comparison of the posterior approach and anterior approach for a Kugel repair of treatment of inguinal hernias. Surg Today. 2013;43(4):403-7.
980	Zhou YM, Zhang XF, Li XD, Liu XB, Wu LP, Li B. Distal pancreatectomy with en bloc celiac axis resection for pancreatic body-tail cancer: Is it justified? Med Sci Monit. 2014;20:1-5.
981	Zhou ZJ, Zheng YL, Hu YH. Effect and cost of perioperative use of antibiotics in coronary artery bypass grafting: a randomized controlled study. Chin Med J. 2011;124(5):699-703.
982	Zhu HW, Wang ZX, Shi W. Keyhole Endoscopic Hematoma Evacuation in Patients. Turk Neurosurg. 2012;22(3):294-9.
983	Zhuang Y, Xiao MD, Yuan ZX, Lu CB, Lin L, Yu M, et al. Early outcomes of isolated coronary artery bypass grafting in Chinese aged patients with diabetes mellitus. Saudi Med J. 2009;30(9):1202-7.
984	Zilberstein B, Jacob CE, Barchi LC, Yagi OK, Ribeiro-Jr U, Coimbra BGMM, et al. Simplified technique for reconstruction of the digestive tract after total and subtotal gastrectomy for gastric cancer: Técnica simplificada de reconstrução do trato digestivo após gastrectomia para câncer gástrico. ABCD arq bras cir dig. 2014;27(2):133-7.
985	Zoumenou E, Gbenou S, Assouto P, Ouro Bang'na Maman AF, Lokossou T, Hounnou G, et al. Pediatric anesthesia in developing countries: experience in the two main university hospitals of Benin in West Africa. Paediatr Anaesth. 2010;20(8):741-7.

High-income country studies

Reference Number	Full reference
1	Billeter AT, Polk HC, Jr., Hohmann SF, Qadan M, Fry DE, Jorden JR, et al. Mortality after elective colon resection: the search for outcomes that define quality in surgical practice. J Am Coll Surg. 2012;214(4):436-43
2	Bregendahl S, Norgaard M, Laurberg S, Jepsen P. Risk of complications and 30-day mortality after laparoscopic and open appendectomy in a Danish region, 1998-2007; a population-based study of 18,426 patients. Pol Przegl Chir. 2013;85(7):395-400.
3	Fair BA, Kubasiak JC, Janssen I, Myers JA, Millikan KW, Deziel DJ, et al. The impact of operative timing on outcomes of appendicitis: a National Surgical Quality Improvement Project analysis. Am J Surg. 2015;209(3):498-502.
4	Flood KM, Said S, Geary M, Robson M, Fitzpatrick C, Malone FD. Changing trends in peripartum hysterectomy over the last 4 decades. Am J Obstet Gynecol. 2009;200(6):632 e1-6.
5	Ingraham AM, Cohen ME, Bilimoria KY, Feinglass JM, Richards KE, Hall BL, et al. Comparison of hospital performance in nonemergency versus emergency colorectal operations at 142 hospitals. J Am Coll Surg. 2010;210(2):155-65.
6	Jakobsson MT, A.M.; Colmorn, L.B.; Lindqvist, P.G.; Klungsoyr, K.; Krebs, L.; et al. . Emergency peripartum hysterectomy: results from the prospective Nordic Obstetric Surveillance Study (NOSS). Acta Obstetricia et Gynecologica Scandinavica. 2015;94:745-54.
7	Knight M, Kurinczuk JJ, Spark P, Brocklehurst P, United Kingdom Obstetric Surveillance System Steering C. Cesarean delivery and peripartum hysterectomy. Obstet Gynecol. 2008;111(1):97-105.
8	Mamidanna R, Burns EM, Bottle A, Aylin P, Stonell C, Hanna GB, et al. Reduced risk of medical morbidity and mortality in patients selected for laparoscopic colorectal resection in England: a population-based study. Arch Surg. 2012;147(3):219-27.
9	Moroz LA, Wright JD, Ananth CV, Friedman AM. Hospital variation in maternal complications following caesarean delivery in the United States: 2006-2012. BJOG. 2016;123(7):1115-20.
10	Perioperative Mortality Review Committee. Sixth Report of the Perioperative Mortality Review Committee Wellington, New Zealand: Health Quality and Safety Commission New Zealand; 2017 [Available from: https://www.hqsc.govt.nz/our-programmes/mrc/pomrc/.
11	Schuitemaker NvR, J.; Dekker, G.; van Dongen, P.; van Geijn, H.; Bennebroek Gravenhorst, J. Maternal mortality after cesarean section in the Netherlands. Acta Obstetricia et Gynecologica Scandinavica. 1997;75:332-4.
